# Comparative efficacy and acceptability of treatments for depressive symptoms in cognitive impairment: A systematic review and Bayesian network meta-analysis

**DOI:** 10.3389/fnagi.2022.1037414

**Published:** 2022-12-12

**Authors:** Boru Jin, Yunting Xv, Bixuan Zhang, Lei Qiao, Huayan Liu

**Affiliations:** ^1^Department of Neurology, First Affiliated Hospital of China Medical University, Shenyang, Liaoning, China; ^2^Department of Neurology, Zhongshan Hospital, Fudan University, Shanghai, China; ^3^Department of Rehabilitation, Fourth Affiliated Hospital of China Medical University, Shenyang, Liaoning, China; ^4^Department of General Surgery, Sheng Jing Hospital of China Medical University, Shenyang, Liaoning, China

**Keywords:** depressive symptoms, cognitive impairment, network meta-analysis, non-pharmacological (NON-Mesh), pharmacological

## Abstract

**Background:**

Depressive symptoms play an essential role in cognition decline, while the benefit and acceptability of treatments for depressive symptoms in cognitive impairment are still unknown.

**Objective:**

To comprehensively evaluate the comparative efficacy and acceptability of treatments for depressive symptoms in cognitive impairment based on the quantitative Bayesian network meta-analysis method (NMA).

**Method:**

We searched MEDLINE, Embase, the Cochrane Library, CINAHL, and PsycINFO from inception until August 2022 to identify randomized clinical trials (RCTs) evaluating treatments for depressive symptoms in cognitive impairment. Efficacy was evaluated by the Cornell Scale for Depression in Dementia (CSDD), the Hamilton Depression Rating Scale (HDRS), and the Geriatric Depression Scale (GDS) for depression; the Neuropsychiatric Inventory (NPI) and the Cohen–Mansfeld Agitation Inventory (CMAI) for behavior; and the Mini-Mental State Examination (MMSE) for cognition. Safety was evaluated by total adverse events (AEs), serious AEs, diarrhea, headache, and nausea.

**Results:**

In this study, 13,043 participants from 107 RCTs were included, involving 28 treatments and the discontinuation of antidepressants. On CSDD, aerobic exercise (MD −4.51, 95%CrI −8.60 to −0.37), aripiprazole (MD −1.85, 95%CrI −3.66 to −0.02), behavioral training (MD −1.14, 95%CrI −2.04 to −0.34), electrical current stimulation (MD −3.30, 95%CrI −5.94 to −0.73), massage (MD −12.67, 95%CrI −14.71 to −10.59), music therapy (MD −2.63, 95%CrI −4.72 to −0.58), and reminiscence therapy (MD −2.34, 95%CrI −3.51 to −1.25) significantly outperformed the placebo. On MMSE, cognitive stimulation therapy (MD 1.42, 95%CrI 0.49 to 2.39), electrical current stimulation (MD 4.08, 95%CrI 1.07 to 7.11), and reminiscence therapy (MD 1.31, 95%CrI 0.04 to 2.91) significantly outperformed the placebo. Additionally, no treatments showed a significantly higher risk than the placebo.

**Conclusion:**

Our NMAs indicated that non-pharmacological interventions were more efficacious and safe than pharmacological treatments for reducing depressive symptoms as well as improving cognitive impairment. Electrical current stimulation, aerobic exercise, and reminiscence therapy could be first recommended considering their beneficial performance on both depression and cognition. Hence, non-pharmacological treatments deserve more attention and extensive application and should at least be considered as an alternative or assistance in clinical settings.

**Systematic review registration:**

https://www.crd.york.ac.uk/prospero/display_record.php?ID=CRD42021239621, identifier: CRD42021239621.

## Introduction

Dementia is a common debilitating disorder affecting an estimated population of 55 million worldwide, and there are nearly 10 million new cases every year (World Health Organization, 2021). Apart from cognition decline, depressive symptoms are closely associated with faster progression of the disease and a huge burden on caregivers (Diniz et al., 2013; Vaughan et al., 2015), affecting up to 63% of the patients with cognitive impairment (Solfrizzi et al., 2007).

Depressive symptoms play an essential role in the occurrence and prognosis of cognition impairment. First, it may be an independent risk factor for cognitive impairment (Bennett and Thomas, 2014), which means that the risk of developing dementia is two-fold in elderly people with a history of depression (Saczynski et al., 2010; Byers and Yaffe, 2011). Second, depressive symptoms can accelerate the deterioration of cognitive impairment and behavioral disturbance, resulting in increased morbidity and mortality (Rapp et al., 2011a,b). Third, viewed as a prodrome of dementia, it could be a reaction or a psychological response to the disease (Kessing, 2012; Bennett and Thomas, 2014; Baruch et al., 2019). Moreover, depressed patients with cognitive impairment are more likely to experience recurrent depression compared to those with simple depression (Hall and Reynolds-Iii, 2014). Hence, alleviation of depressive symptoms is of paramount importance to delay the course of the disease and improve the quality of life of patients.

Pharmacological approaches for depression remain the mainstay of treating depressive symptoms in cognitive impairment (Kessing et al., 2007), though they may not be necessarily effective and tolerable (Bingham et al., 2019). Recent reviews provided conflicting results on the benefits of antidepressants and even claimed minimal or no effect on depression symptoms, cognitive function, or activities of daily living (Orgeta et al., 2017; Dudas et al., 2018). Some also indicated that patients on antidepressants were more likely to suffer from side effects (Farina et al., 2017; Dudas et al., 2018; Baruch et al., 2019). Meanwhile, a few meta-analyses claimed the beneficial effects of non-pharmacological treatments for depressive symptoms in cognitive impairment, though most of them only focused on limited interventions and were of unsatisfactory quality (Woods et al., 2018; Bennett et al., 2019; Li H. C. et al., 2019; Li X. et al., 2019; Zafra-Tanaka et al., 2019). There is still a lack of comprehensive assessment of both pharmacological and non-pharmacological treatments for depressive symptoms in cognitive impairment.

The traditional pairwise meta-analysis method can only compare two interventions at a time utilizing direct evidence, which would provide limited insights when there are no head-to-head clinical trials. Given the complexity of this targeted issue, network meta-analysis (NMA) is adopted to face this challenge, as it is capable of fully utilizing both direct and indirect evidence and presenting a comparative hierarchy of efficacy and acceptability. As a powerful and reliable method, NMA has been widely applied to explore the potential evidence (Mutz et al., 2019; Zhou et al., 2020).

Hence, we conducted this systematic review and NMA to evaluate all available treatments for depressive symptoms in cognitive impairment, aiming to provide comparative evidence and quantitative hierarchies on both efficacy and acceptability.

## Methods

We performed a series of NMAs using the Bayesian model, which strictly conformed to the principles of the Preferred Reporting Items for Systematic Reviews and Meta-Analyses (PRISMA) extension statement for reporting systematic reviews incorporating NMA of health interventions (Supplementary material 1) (Hutton et al., 2015). We registered our work in the PROSPERO database (https://www.crd.york.ac.uk/prospero/display_record.php?ID=CRD42021239621, identifier: CRD42021239621).

### Eligibility criteria

#### Participants

Participants were diagnosed with mild cognitive impairment (MCI) or various types of dementia according to corresponding criteria. According to the ADNI definition, cognitive impairment is found to be consistent with amnestic MCI (Petersen, 2004; Albert et al., 2011). Dementia was defined by the study authors on the basis of diagnostic criteria such as the Diagnostic and Statistical Manual of Mental Disorders, 5th edition (DSM-5) for dementia (American Psychiatric Association, 2013), the National Institute of Neurological and Communicative Disorders and Stroke and the Alzheimer's Disease and Related Disorders Association (NINCDS–ADRDA) criteria for Alzheimer's disease (AD) (McKhann et al., 1984), etc. There were no restrictions on sex, age, ethnicity, nationality, or duration of disease.

#### Interventions

All available treatments including both pharmacological and non-pharmacological treatments of depressive symptoms in cognitive impairment were carefully considered. Accordingly, we mainly searched several fields of pharmacological and non-pharmacological therapies, such as antidepressants, antipsychotics, N-Methyl-d-aspartate receptor antagonists (NMDA), analgesics, hormones, cognitive stimulation therapy, non-invasive brain stimulation, psychological treatments, multidomain interventions, and so on. Specific potential pharmacological and non-pharmacological treatments that we searched for are listed in Table 1.

**Table 1:** Potential pharmacological and non-pharmacological interventions.

**Pharmaceutical treatments**		
1	Selective serotonin reuptake inhibitors	Citalopram, dapoxetine, escitalopram, fluoxetine, fluvoxamine, indalpine, paroxetine, sertraline, vilazodone, zimelidine, venlafaxine, desvenlafaxine, duloxetine, milnacipran, levomilnacipran, sibutramine, bicifadine, etc.
2	Selective serotonin receptor agonists	Triptans, intranasal sumatriptan, almotriptan, eletriptan, frovatriptan, naratriptan, rizatriptan, sumatriptan, zolmitriptan, etc.
3	Tricyclic antidepressants	Amitriptyline, amoxapine, clomipramine, desipramine, dibenzepin, dothiepin, doxepin, imipramine, lofepramine, nortriptyline, opipramol, protriptyline, trimipramine, etc.
4	Serotonin antagonists	Pizotifen, sandomigran, etc.
5	Cholinesterase inhibitors (ChEIs)	Donepezil, galantamine, rivastigmine
6	N-Methyl-d-aspartate receptor antagonist	Memantine
7	Antipsychotics	Aripiprazole, chlorpromazine, clozapine, haloperidol, levomepromazine, perphenazine, prochlorperazine, olanzapine, quetiapine, risperidone, etc
8	Analgesics	Morphine, tramadol, meperidine, acetaminophen, lysine acetylsalicylic acid (L-ASA), etc.
9	Hormone	Progestin, estradiol, norethisterone, estrogen, etc.
10	others	Chinese medicine, lithium, methylphenidate, melatonin, EGb 761 (ginkgo), etc.
**Non-pharmaceutical treatments**		
1	Cognitive therapy	Mindfulness-based stress reduction, cognitive behavioral therapy, peaceful mind, individualized cognitive rehabilitation, etc.
2	Non-invasive brain stimulation	Transcranial magnetic stimulation (TMS), Transcranial direct current stimulation (tDCS), transcutaneous electrical nerve stimulation, etc.
3	Psychological treatments	Psychological sleep interventions, recognizable psychotherapeutics, reminiscence therapy
4	Physiotherapy approach	Manual therapy, soft-tissue techniques, strength and endurance training, yoga and tai chi, spinal manipulation, massage, oxygen therapy, etc.
5	Exercise	Aerobic-exercise, strength-exercise, etc.
6	Multidomain interventions	Multi-faceted intervention, collaborative care, multimodal rehabilitative intervention, comprehensive home-based care intervention, etc.
7	Others	Occupational therapy, music therapy, supplements, botanicals and diet alteration, mind-body therapy, robot-assisted therapy, animal-assisted therapy, ultrasound guided nerve pulsed radiofrequency, acupuncture, etc.

#### Comparators

Placebo, usual care or therapy, and any other corresponding pharmacological or non-pharmacological interventions were eligible.

#### Outcomes

After a comprehensive investigation of all the scales evaluating symptoms of cognition impairment, we finally selected the Cornell Scale for Depression in Dementia (CSDD) (Alexopoulos et al., 1988), the Hamilton Depression Rating Scale (HDRS) (Endicott et al., 1981), and the Geriatric Depression Scale (GDS) (Yesavage, 1988) to evaluate the alleviation of depressive symptoms; the Neuropsychiatric Inventory (NPI) (Cummings et al., 1994) and the Cohen–Mansfeld Agitation Inventory (CMAI) (Finkel et al., 1992) to appraise the psychiatric condition; and the Mini-Mental State Examination (MMSE) (Folstein et al., 1983) to access the change of cognition impairment. Among the overall adverse events (AEs), we selected the risk of total AEs, diarrhea, headaches, nausea, and severe AEs as secondary outcomes of acceptability because of their highest occurrence. The data that we extracted were the results of the intent-to-treat population using the last observation carried forward method, but some were unavailable.

#### Information source and literature search

The systematic literature search was performed utilizing databases of MEDLINE, Embase, Cochrane Central Register of Controlled Trials (CENTRAL), Cumulative Index to Nursing and Allied Health (CINAHL), and PsycINFO. The search strategy was characteristically designed for each of the five databases by combining free text, Medical Subject Heading, and EMTREE terms, among others (Supplementary material 2). The search covered English-language articles from inception until August 2022. Each database and registration platform would be retrieved again before completing the NMAs in case of omitting any newly published works. The unpublished studies were retrieved *via* conference proceedings, clinical trial registries, and author contact. Only potential studies for inclusion were scanned carefully from the reference lists of included studies and related reviews.

### Data collection and analysis

#### Study selection

We only included high-quality randomized controlled trials (RCTs) in English that appraised the efficacy or acceptability of any pharmacological or non-pharmacological intervention treating depressive symptoms in cognitive impairment. Following the eligibility criteria elucidated above, the evaluation and screening of articles were performed by two reviewers independently. When there is any controversy after elaborate discussion, a third reviewer then intervened to make the final decision. After deleting the duplicates, they screened the titles and abstracts of the left literature to select the ones that were worthy of being reviewed in full text. Based on such a rigorous and scientific review, the finally included RCTs were identified.

#### Data extraction and quality

Baseline characteristics of the included studies and potential effect modifiers were widely abstracted, including age, sex constituent ratio of patients, duration of treatment, the sample size of trials, the dosage of treatments, baseline scores of MMSE, CSDD, GDS, and NPI, and completed ratio of participants. Then, primary outcomes of efficacy and secondary outcomes of acceptability were extracted, carefully considering the methods of drug delivery, schedules of drug administration, the context of non-pharmacological treatment, etc.

#### Risk of bias assessment

The risk of bias of included RCTs was strictly evaluated according to the criteria outlined in the Cochrane Handbook for Systematic Reviews of Interventions (Higgins et al., 2019), which appraises six aspects including random sequence generation (selection bias), allocation concealment (selection bias), blinding of participants and personnel (performance bias), blinding of outcome assessment (detection bias), incomplete outcome data (attrition bias), selective reporting (reporting bias), and other bias. This evaluation was done by two reviewers independently to assign a level of risk of bias (high risk, unclear risk, and low risk) for each item, and when there was any controversy, a third reviewer would give the final assessment. Overall, RCTs included in our NMAs had a relatively acceptable low risk of biases across different parameters scored. Additionally, the non-pharmacological treatment could not realize the concealment of patients, thus causing minimal bias which is usually allowed.

#### Outcome measurement

The primary outcomes were described by the changed scores of the scales, which belong to continuous data and were computed as mean difference (MD) and 95% credibility intervals (CrI). The secondary outcomes of acceptability were described by the number of adverse events that occurred, which belong to discontinuous data, and were computed as the hazard ratio (HR) and 95% CrIs.

#### Data synthesis and statistical analysis

Our systematic review and NMAs were done across all types of dementia and MCI to derive the overall efficacy and acceptability of comprehensive therapies for depressive symptoms in cognitive impairment. Initially, we summarized and examined baseline data of characteristics of the involved RCTs and patients to access the clinical and methodological heterogeneity. Additionally, the geometry of the network of comparisons across trials was connected to make sure each included RCT would be involved in our NMAs. Then, traditional pairwise meta-analyses were done to anticipate the heterogeneity and publication bias among the RCTs before NMAs. The heterogeneity was assessed by *I*^2^ statistic, and the publication bias was judged by funnel plots.

Next, NMAs were conducted within a Bayesian hierarchical model framework to estimate all the included valuable treatments. We adopted the random-effect model in our NMAs because it could be the most appropriate and advisable methodology in consideration of between-study heterogeneities (Mills et al., 2013). Specifically, models were applied using four chains of Markov Chain Monte Carlo estimation running for 100,000 iterations with thinning of 10, and the first 20,000 iterations were discarded as burn-in after visual inspection of the mixing chains. The convergence was estimated by visually examining the iteration plot and the potential scale reduction factor. Overall, the process above was performed in R version 4.0.4.

Additionally, we carefully considered transitivity and similarity, based on which assumption was made by comparing the distribution of studies and baseline characteristics of participants and by examining potential effect modifiers such as age, the timing of exposure, and the risk of bias. Besides, since a large number of treatments may lead to unavailable cases, the common within-network between-study variance (τ2) across comparisons was presumed for all comparisons in the entire networks. As for consistency, the design-by-treatment interaction model was adapted to examine the consistency of NMAs. If the inconsistency was tracked without identifying any discrepancy to blame, we then appraised the local inconsistency of each network loop using a loop-specific method (Veroniki et al., 2013). Furthermore, additional analyses were done to enhance the scientificity and preciseness of this NMA, such as sensitivity analysis and subgroup analysis.

## Results

### Literature search and description of studies

**Figure 1:**
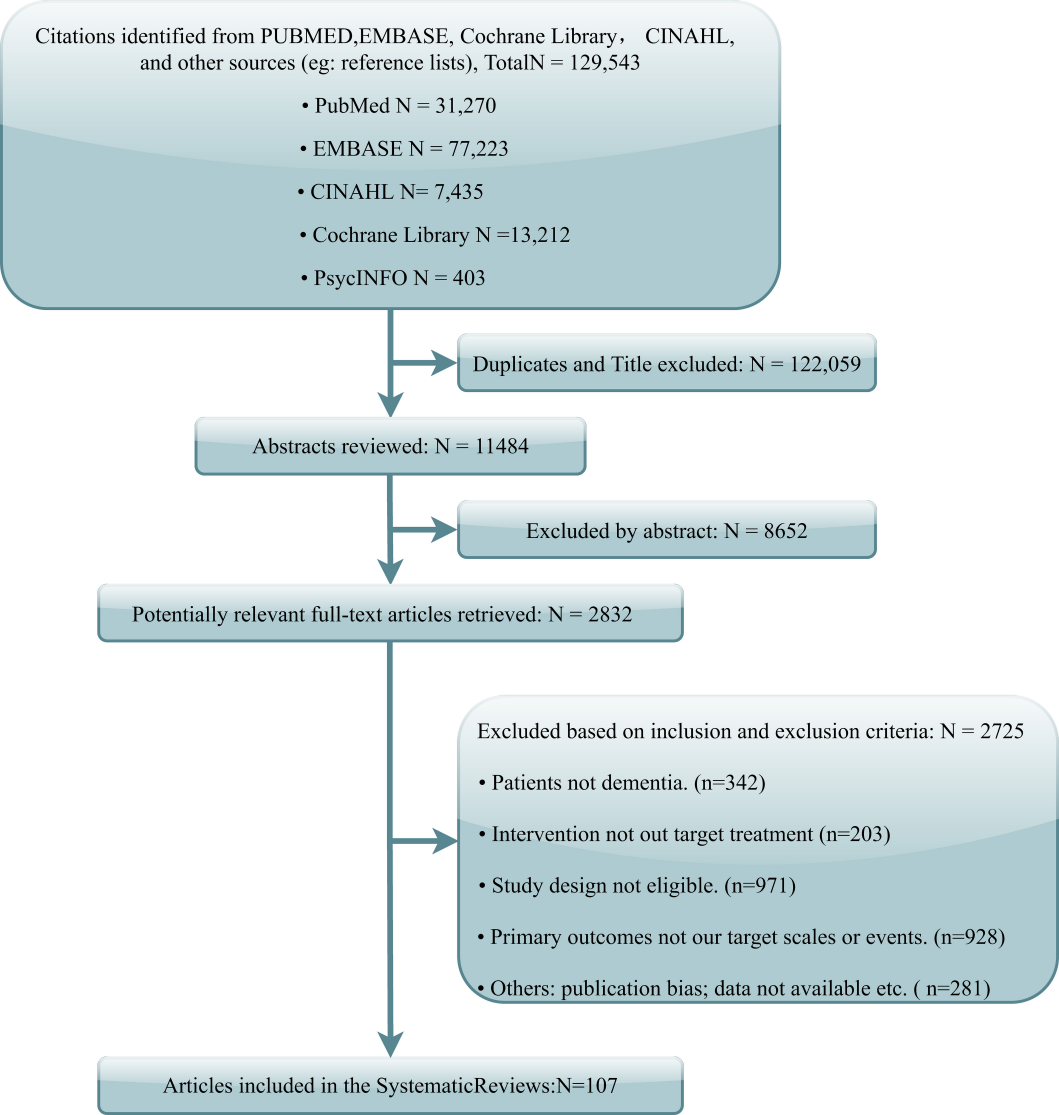
A flowchart of the screening process.

The literature search yielded 129,543 potentially relevant records. After the deletion of duplicates and titles, 11,484 abstracts were screened, and 2,832 articles were left for full-text review. Finally, 107 articles were identified referring to the inclusion criteria (Figure 1). Notably, one study fulfilled all criteria but was excluded from the NMA because of the apparent publication bias displayed in funnel plots and recognized by three reviewers (Rodriguez-Mansilla et al., 2015).

#### Characteristics of the studies

**Figure 2:**
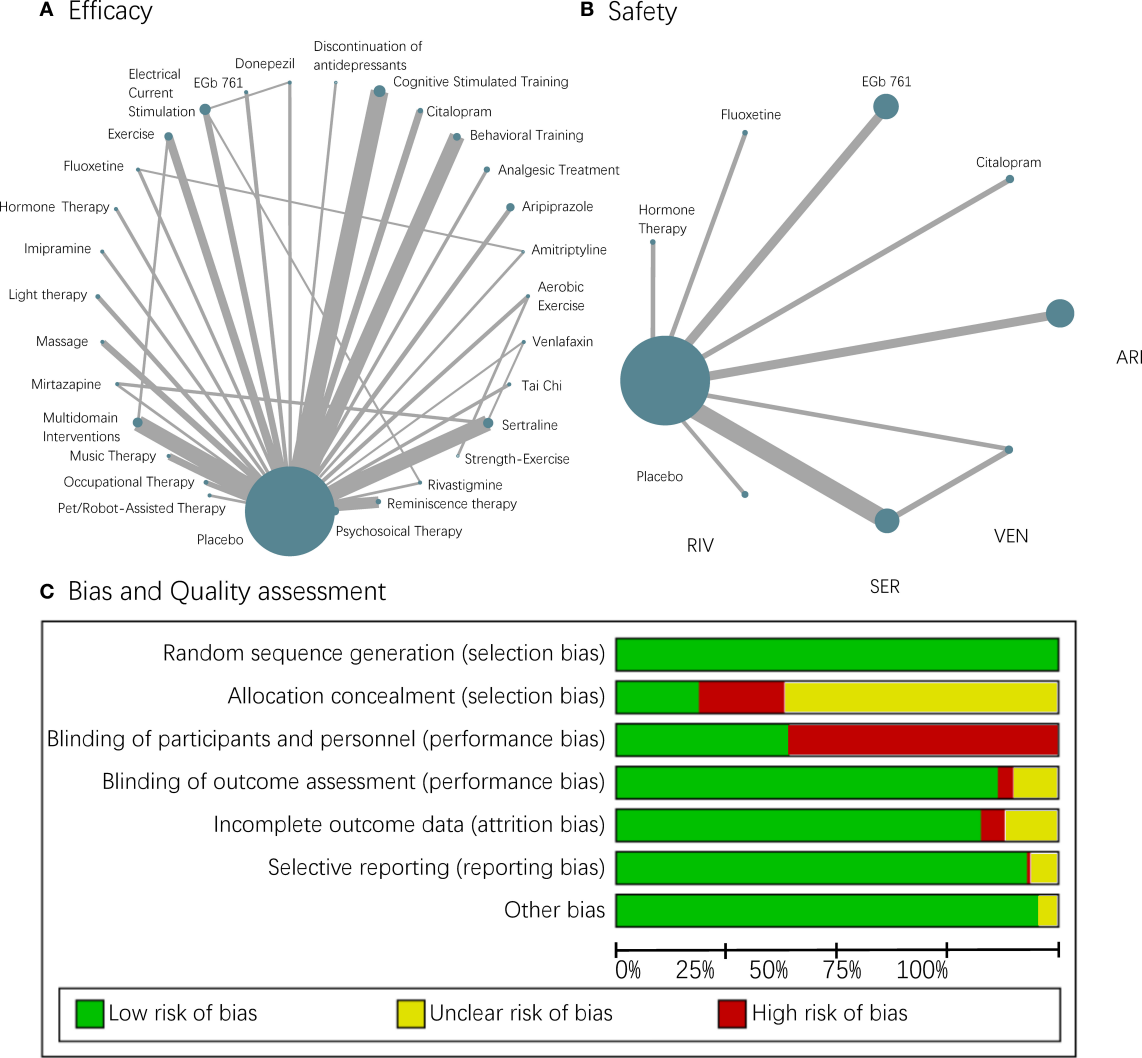
A network plot and risk of bias. **(A,B)** Each node in the network geometry represents a kind of intervention. Nodes will be linked by a line when the treatments are directly comparable with “head-to-head trails.” The size of the nodes corresponds to the number of participants receiving that intervention, and the width of the lines is proportional to the number of RCTs this comparison included. **(C)** Risk of bias chart of studies included in the quantitative analysis.

In this study, 13,043 participants from 107 RCTs were included, involving 13 pharmacological treatments, 15 non-pharmacological treatments, and the discontinuation of antidepressants. The weighted network plots of efficacy and safety are described in Figures 2A,B. The characteristics of these RCTs are displayed in Supplementary material 3.

#### Risk of bias

The risk of bias within each study included in our NMAs was acceptable (Figure 2C), though the blinding of participants and personnel in non-pharmacological interventions was impossible and appeared to cause a high risk. Funnel plots were also employed to evaluate the publication bias, and they were all visualized as being symmetric generally (Supplementary material 4). The heterogeneity described by *I*^2^ was small, and the overall stability of NMAs revealed from sensitivity analysis was quite well, except for a minimal hesitation on MMSE (Supplementary material 5). Therefore, we further did a subgroup analysis of AD to explore the potential effect of diagnosis and gained generally consistent results with the whole group (Supplementary materials 6–8).

#### Grading the evidence

We used the Grades of Recommendation, Assessment, Development, and Evaluation (GRADE) approach to grade the quality of underlying evidence and the strength of recommendations in this study. Our GRADE judgments focused on six aspects, including within-study bias, reporting bias, indirectness, imprecision, heterogeneity, and incoherence, and finally led to a good confidence rating. Additionally, we performed this evaluation using CINeMA, which is recommended for assessing confidence in the results of a network meta-analysis (Nikolakopoulou et al., 2020) (Supplementary material 9).

### Efficacy

#### Depression

**Figure 3:**
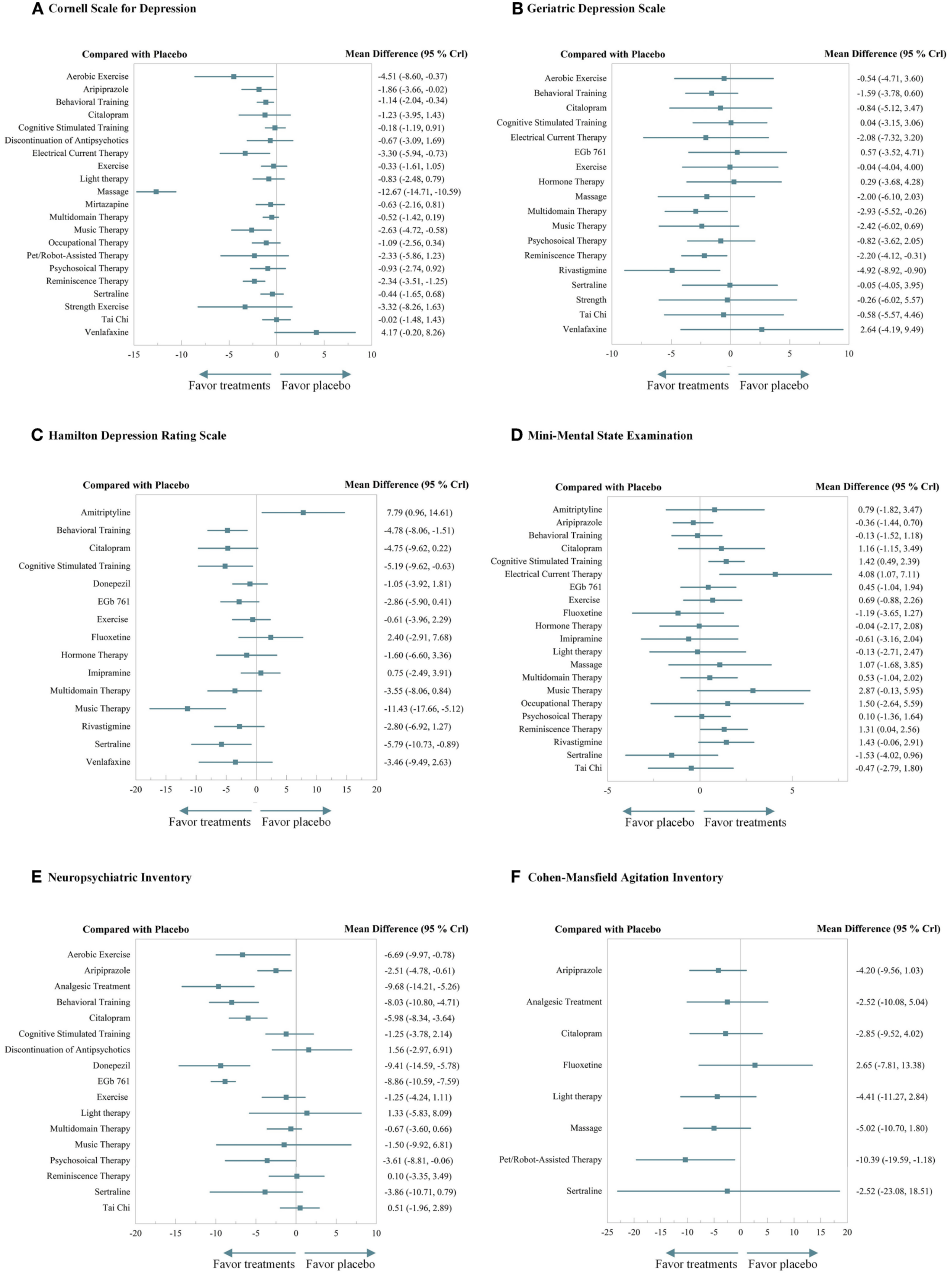
A forest plot of efficacy. **(A)** Cornell Scale for Depression. **(B)** Geriatric Depression Scale. **(C)** Hamilton Depression Rating Scale. **(D)** Mini-Mental State Examination. **(E)** Neuropsychiatric Inventory. **(F)** Cohen–Mansfeld Agitation Inventory.

The NMA on CSDD was performed across 20 treatments and the discontinuation of antidepressants, based on 46 RCTs with 5,143 patients. The results showed that seven treatments showed significant differences superior to placebo including aerobic exercise (MD −4.51, 95%CrI −8.60 to −0.37), aripiprazole (MD −1.86, 95%CrI −3.66 to −0.02), behavioral training (MD −1.14, 95%CrI −2.04 to −0.34), electrical current stimulation (MD −3.30, 95%CrI −5.94 to −0.73), massage (MD −12.67, 95%CrI −14.71 to −10.59), music therapy (MD −2.63, 95%CrI −4.72 to −0.58), and reminiscence therapy (MD −2.34, 95%CrI −3.51 to −1.25). Massage outperformed all the other 21 interventions. Interventions such as aerobic exercise, behavioral training, and electrical current stimulation also demonstrated significant efficacy, while the venlafaxine behaved quite unsatisfactorily. Antidepressants that were discontinued displayed no significant harm. Specific results of efficacy and elaborate rank of the hierarchy are shown in Figure 3A and Supplementary materials 10A, 11A.

The NMA on GDS was performed across 18 treatments based on 31 RCTs with 2,872 patients. The results demonstrated that multidomain interventions (MD −2.93, 95%CrI −5.52 to −0.26), reminiscence therapy (MD −2.20, 95%CrI −4.12 to −0.31), and rivastigmine (MD −4.92, 95%CrI −8.92 to −0.90) significantly outperformed the placebo, and other interventions also demonstrated an effective tendency over placebo, though without significance. Specific results of efficacy and elaborate rank of the hierarchy are shown in Figure 3B and Supplementary materials 10B, 11B.

The NMA on HDRS was performed across 15 treatments based on 19 RCTs with 1,591 patients. The results indicated that behavioral training (MD −4.78, 95%CrI −8.06 to −1.51), cognitive stimulation therapy (MD −5.19, 95%CrI −9.62 to −0.63), music therapy (MD −11.43, 95%CrI −17.66 to −5.12), and sertraline (MD −5.79, 95%CrI −10.73 to −0.89) significantly outperformed the placebo. Music therapy outperformed nine other treatments with significant differences, while amitriptyline performed disappointingly. Specific results of efficacy and elaborate rank of the hierarchy are shown in Figure 3C and Supplementary materials 10C, 11C.

#### Cognition

The NMA on MMSE was performed across 21 treatments based on 53 RCTs with 5,995 patients. The results indicated that cognitive stimulation therapy (MD 1.42, 95%CrI 0.49 to 2.39), electrical current stimulation (MD 4.08, 95%CrI 1.07 to 7.11), and reminiscence therapy (MD 1.31, 95%CrI 0.04 to 2.56) significantly outperformed the placebo. Especially, electrical current stimulation significantly outperformed the other 10 treatments, while sertraline performed inferior to the three treatments. Specific results of efficacy and elaborate rank of the hierarchy are shown in Figure 3D and Supplementary materials 10D, 11D.

#### Behavior

The NMA on NPI was performed across 17 treatments based on 33 RCTs with 6,524 patients. The results suggested that aerobic exercise (MD −6.69, 95%CrI −9.97 to −0.78), aripiprazole (MD −2.51, 95%CrI −4.78 to −0.61), analgesic treatment (MD −9.68, 95%CrI −14.21 to −5.26), behavioral training (MD −8.03, 95%CrI −10.80 to −4.71), citalopram (MD −5.98, 95%CrI −8.34 to −3.64), donepezil (MD −9.41, 95%CrI −14.59 to −5.78), EGb761 (MD −8.86, 95%CrI −10.59 to −7.59), and psychosocial therapy (MD −3.61, 95%CrI −8.81 to −0.06) significantly outperformed the placebo. Citalopram significantly outperformed five treatments. Analgesic treatment, behavioral training, and EGb761 also demonstrated beneficial significance over others. Besides, the discontinuation of antidepressants did not show significant harm. Specific results of efficacy and elaborate rank of the hierarchy are shown in Figure 3E and Supplementary materials 10E, 11E.

The NMA on CMAI was performed across 8 treatments based on 11 RCTs with 1,508 patients. The results demonstrated that pet/robot-assisted therapy (MD −10.39, 95%CrI −19.59 to −1.18) significantly outperformed the placebo. Specific results of efficacy and elaborate rank of the hierarchy are shown in Figure 3F and Supplementary materials 10F, 11F.

### Acceptability

**Figure 4:**
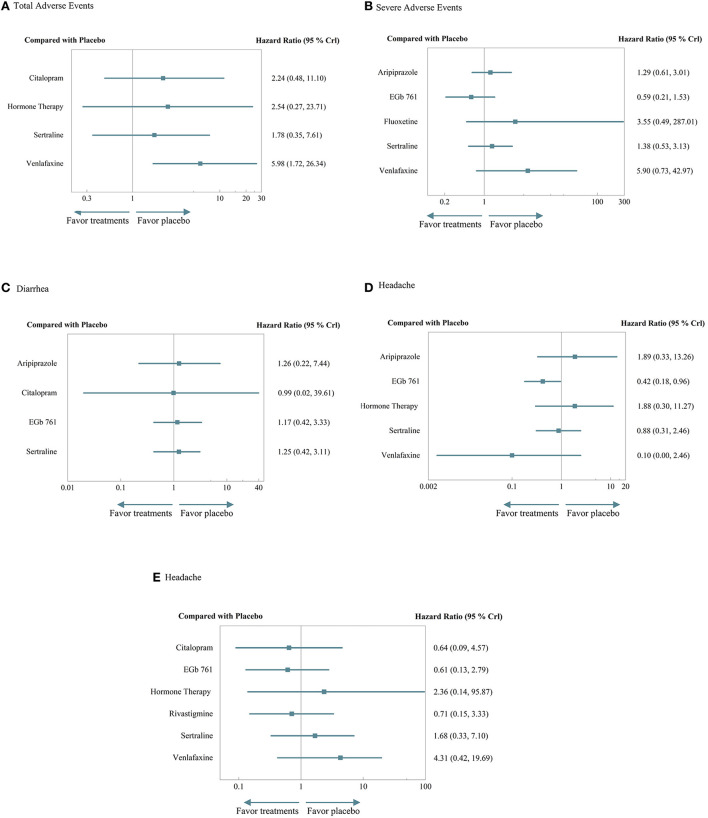
A forest plot of acceptability. **(A)** Total adverse events. **(B)** Severe adverse events. **(C)** Diarrhea. **(D)** Headache. **(E)** Nausea.

The reported data on adverse events from the included RCTs were carefully considered based on eight pharmacological treatments. NMAs were conducted over the incidence of total adverse events and the four most common and important adverse events, including headache, nausea, diarrhea, and serious adverse events. Overall, none of the treatments showed any significance compared to the placebo, while most of them had a riskier tendency than the placebo. In terms of total adverse events, severe AEs, and nausea, venlafaxine seemed to be the most likely to cause adverse events. EGB 761 and citalopram performed well usually, with relatively lower risk. Specific results of efficacy and elaborate rank of the hierarchy are shown in Figure 4 and Supplementary materials 12, 13.

## Discussion

Our NMAs are based on 107 RCTs, including 13,043 patients with depressive symptoms in cognitive impairment, who were randomly assigned to 13 pharmacological treatments, 15 non-pharmacological treatments, and the discontinuation of antidepressants. We evaluated all available and high-quality treatments for depressive symptoms in cognitive impairment concerning four aspects of depression, cognition, behavior, and acceptability, providing comparative evidence and quantitative hierarchies using the NMA method. Our objective was to quantitatively determine if all the most common 28 treatments helped patients with depressive symptoms in cognitive impairment and if the discontinuation of antidepressants did significantly harm patients.

Overall, our results suggested that non-pharmacological treatments could be beneficial in reducing symptoms of depression and may be even more efficacious than pharmacological ones. On depression, NMA on CSDD revealed that six treatments could provide more benefits than control care or placebo, including aerobic exercise, behavioral training, electrical current stimulation, massage, music therapy, and reminiscence therapy. However, no pharmacological treatments presented any statistical significance superior to a placebo on depression, some of which even had a tendency of aggravation, such as venlafaxine and amitriptyline. On cognition, non-pharmacological interventions such as cognitive stimulation therapy, electrical current stimulation, and reminiscence therapy outperformed the usual care and pharmacological ones. On behavior, non-pharmacological interventions also behaved quite well, especially for the pet/robot-assisted therapy on agitation and aerobic exercise on total neuropsychiatric symptoms. Moreover, NMAs of acceptability revealed that most pharmacological treatments were associated with a riskier tendency of adverse events, indicating potential poor acceptability. Hence, our analysis suggested that non-pharmacological treatments could provide more benefits and cause less risk, which should be applied more widely and at least considered as an alternative in clinical settings.

Specifically, among the non-pharmacological treatments, electrical current stimulation, aerobic exercise, and reminiscence therapy are quite recommended due to their satisfactory performance in both depression and cognition. When considering the behavioral problem, aerobic exercise may be the first choice due to its beneficial performance in all aspects. Although massage demonstrated significant efficacy over usual care, there should be hesitation in interpretation due to the limited data and asymmetric funnel plot. As for the pharmacological treatments, aripiprazole and citalopram may own the most possibility to have some benefits on depression, which also showed significant improvements on NPI and no harm to MMSE. We also noted that the efficacy of pharmacological treatments on the alleviation of neuropsychiatric symptoms according to NMA on NPI should be claimed, which reminds us that there may be a combined treatment when there are severe neuropsychiatric problems. Besides, no significant harm caused by the discontinuation of antidepressants was observed for both depressive and neuropsychiatric symptoms. A clinical treatment strategy should be designed concerning the specific situation of physical and mental health of patients to figure out the most beneficial simple or combined interventions. In addition, we called for more RCTs on combined interventions to help us better assess and design the clinical treatment strategy.

Pursuing the credibility of evidence, the bias of risk accessed by the Cochrane risk of bias tool implies relatively low bias, though some unclear bias may come from the blinding inadequacy due to the methodological shortcoming that non-pharmacological approaches are unable to realize double-blind. No important discrepancies across the direct comparisons in the distribution of study characteristics were observed after attempting to examine the potential effect modifiers on transitivity. Funnel plots were evaluated visually and believed to be symmetrical after excluding one; thus, publication bias is unlikely with our comprehensive search strategy. Subgroup analysis of AD was done to explore the potential heterogeneity from diagnosis and delivered consistent conclusions. Besides, we appraise the network inconsistency through node-split modeling.

It should be highlighted that we have several strengths. First, as there is a lack of evidence, this study may be the first attempt to quantitatively synthesize the efficacy and safety of treatments for depressive symptoms in cognitive impairment by the NMA method. The included RCTs were concerned with both pharmacological and non-pharmacological approaches, all of which strictly followed the inclusion/exclusion criteria with high quality and low bias. Second, based on the NMA method, not only the head-to-head studies but also the indirect comparisons were comprehensively analyzed, which gave rise to comparative evaluation and derived hierarchies. Third, we performed NMAs on three aspects of efficacy, including depression, cognition, and behavior, utilizing six professional scales, aiming at providing a more specific evaluation and description of the benefits and harms. In addition, acceptability was carefully assessed to rise some attention to clinical prescriptions. Fourth, our conclusions were based on a substantial number of patients and RCTs compared with the previous knowledge syntheses, giving rise to a great guarantee of precision and credibility.

Our analysis also has limitations. First, for some treatments, the paucity of reported RCTs may limit the comprehensiveness and power of this analysis. Second, to maintain the precision of conclusions, the review was restricted to high-quality trials of a single intervention, given that most combined ones were inconsistent and lacked enough data. Third, since the number of patients and comprehensive interventions are quite large, some biases may be inevitable, such as the discrepancies in duration and gender radio, though we have tried our best to appraise and avoid them. Fourth, since different scales may have incompatible abilities in evaluation owing to their intrinsic characteristics, the divergences of their results should be interpreted modestly.

## Conclusion

Our NMAs indicated that non-pharmacological interventions were more efficacious and safe than pharmacological treatments for treating depressive symptoms in cognitive impairment. Specifically, electrical current stimulation, aerobic exercise, and reminiscence therapy were quite recommended considering their beneficial performance on both depression and cognition. Additionally, since there were some limitations, we expect to update and revise our NMAs furthermore.

## Data availability statement

The original contributions presented in the study are included in the article/Supplementary material, further inquiries can be directed to the corresponding authors.

## Author contributions

HL, LQ, and BJ developed the concept and design of the study. BJ and BZ conducted systematic literature searches, extracted the data, and rated the risk of bias and methodological quality criteria of included studies, under the supervision of YX. LQ and BJ analyzed and interpreted the data. BJ wrote the first draft of the manuscript. All authors have contributed to the further revision and have approved the final manuscript.

## Supplementary material

Supplementary material 1 PRISMA NMA checklist.

Supplementary material 2 Searching strategies of the current network meta-analysis.

Supplementary material 3 Baseline characteristics of the included randomized controlled trails.

Supplementary material 4 Comparison-adjusted funnel plot. The red vertical line represents the null hypothesis that independent effect size estimates do not differ from the comparison-specific pooled estimates.

Supplementary material 5 Sensitivity and heterogeneity analysis. When the difference in DIC between the random-effect and fixed-effect models is close, the models could be viewed as stable and reliable. When the *I*^2^% is <28%, we can conclude that the heterogeneity is relatively minor.

Supplementary material 6 A subgroup analysis of Alzheimer's disease (forest plot of efficacy).

Supplementary material 7 A subgroup analysis of Alzheimer's disease (league tables of efficacy). The diagonal gives the different treatments examined in the network meta-analyses. The efficacy data are given as mean difference (MD) with a 95% credible interval (CrI), in which each cell indicates the values for a specific contrast between the treatments.

Supplementary material 8 A subgroup analysis of Alzheimer's disease (relative ranks of efficacy). The derived hierarchies (relative ranks) are described from the most to the least effective and the most effective to the least.

Supplementary material 9 Grades of Recommendation, Assessment, Development, and Evaluation (GRADE).

Supplementary material 10 League tables of efficacy. The diagonal gives the different treatments examined in the network meta-analyses. The efficacy data are given as mean difference (MD) with a 95% credible interval (CrI), in which each cell indicates the values for a specific contrast between the treatments.

Supplementary material 11 Relative ranks of efficacy. The derived hierarchies (relative ranks) are described from the most to the least effective and the most effective to the least.

Supplementary material 12 League tables of acceptability. The diagonal gives the different treatments examined in the network meta-analyses. The efficacy data are given as hazard ratio (HR) with a 95% credible interval (CrI), in which each cell indicates the values for a specific contrast between the treatments.

Supplementary material 13 Relative ranks of acceptability. The derived hierarchies (relative ranks) are described from the most to the least effective and the most acceptable to the least.
