# Supplementary material for: Comparative efficacy and acceptability of treatments for depressive symptoms in cognitive impairment: A systematic review and Bayesian network meta-analysis
Source: Front Aging Neurosci. 2022 Dec 12;14:1037414. doi: 10.3389/fnagi.2022.1037414 (PMC9790988; doi:10.3389/fnagi.2022.1037414)
Supplement: Supplementary material 1 — PRISMA NMA checklist. [file Data_Sheet_1.zip › Supplementary Material Presentation/Supplementary_10.pdf]

## A. Cornell Scale for Depression

| | | | | | | | | | | | | | | | | | | | | | |
|---|---|---|---|---|---|---|---|---|---|---|---|---|---|---|---|---|---|---|---|---|---|
| Aerobic Exercise | 2.62 (-1.93, 7.12) | 3.35 (-0.89, 7.46) | 3.21 (-1.65, 8.18) | 4.32 (0.06, 8.52) | 3.77 (-0.97, 8.53) | 1.18 (-3.68, 5.98) | 4.17 (-0.17, 8.42) | 3.67 (-0.85, 8.08) | -8.19 (-12.91, -3.58) | 3.87 (-0.39, 8.12) | 3.96 (-0.27, 8.09) | 1.80 (-2.79, 6.43) | 3.40 (-0.99, 7.68) | 4.51 (0.37, 8.60) | 2.15 (-3.34, 7.72) | 3.55 (-0.92, 8.04) | 2.13 (-2.15, 6.32) | 4.08 (-0.21, 8.23) | 1.14 (-1.47, 3.79) | 4.47 (0.15, 8.79) | 8.65 (2.85, 14.37) |
| -2.62 (-7.12, 1.93) | Aripiprazole | 0.71 (-1.36, 2.64) | 0.62 (-2.61, 3.88) | 1.67 (-0.37, 3.83) | 1.17 (-1.85, 4.13) | -1.44 (-4.67, 1.72) | 1.53 (-0.65, 3.81) | 1.03 (-1.50, 3.49) | -10.81 (-13.59, -8.06) | 1.22 (-1.17, 3.59) | 1.33 (-0.78, 3.24) | -0.77 (-3.53, 1.92) | 0.76 (-1.61, 3.06) | 1.85 (0.02, 3.66) | -0.45 (-4.48, 3.50) | 0.92 (-1.61, 3.51) | -0.48 (-2.70, 1.61) | 1.42 (-0.82, 3.60) | -1.46 (-6.73, 3.80) | 1.84 (-0.51, 4.13) | 6.06 (1.34, 10.56) |
| -3.35 (-7.46, 0.89) | -0.71 (-2.64, 1.36) | Behavioral Training | -0.08 (-2.90, 2.77) | 0.96 (-0.32, 2.43) | 0.49 (-2.06, 2.98) | -2.16 (-4.91, 0.60) | 0.82 (-0.66, 2.52) | 0.32 (-1.47, 2.19) | -11.53 (-13.72, -9.23) | 0.51 (-1.18, 2.20) | 0.62 (-0.56, 1.74) | -1.47 (-3.71, 0.73) | 0.07 (-1.58, 1.74) | 1.14 (0.34, 2.04) | -1.18 (-4.83, 2.48) | 0.23 (-1.73, 2.32) | -1.19 (-2.60, 0.18) | 0.70 (-0.72, 2.21) | -2.17 (-7.21, 2.84) | 1.12 (-0.49, 2.86) | 5.33 (0.90, 9.55) |
| -3.21 (-8.18, 1.65) | -0.62 (-3.88, 2.61) | 0.08 (-2.77, 2.90) | Citalopram | 1.06 (-1.80, 4.04) | 0.58 (-3.04, 4.15) | -2.05 (-5.85, 1.70) | 0.92 (-2.09, 3.98) | 0.42 (-2.79, 3.52) | -11.42 (-14.73, -8.04) | 0.61 (-2.41, 3.64) | 0.69 (-2.14, 3.48) | -1.39 (-4.79, 2.00) | 0.14 (-2.91, 3.27) | 1.23 (-1.43, 3.95) | -1.09 (-5.59, 3.47) | 0.33 (-2.94, 3.63) | -1.12 (-4.06, 1.85) | 0.80 (-2.09, 3.72) | -2.01 (-7.73, 3.55) | 1.21 (-1.79, 4.31) | 5.39 (0.37, 10.43) |
| -4.32 (-8.52, -0.06) | -1.67 (-3.83, 0.37) | -0.96 (-2.43, 0.32) | -1.06 (-4.04, 1.80) | Cognitive Stimulated Training | -0.49 (-3.22, 2.06) | -3.11 (-6.00, -0.39) | -0.13 (-1.36, 1.05) | -0.64 (-2.72, 1.29) | -12.49 (-14.84, -10.19) | -0.46 (-2.30, 1.35) | -0.34 (-1.79, 0.85) | -2.46 (-4.86, -0.10) | -0.89 (-2.81, 0.82) | 0.18 (-0.91, 1.19) | -2.16 (-5.95, 1.61) | -0.75 (-2.89, 1.36) | -2.15 (-3.80, -0.69) | -0.26 (-1.90, 1.31) | -3.14 (-8.20, 1.87) | 0.18 (-1.68, 1.90) | 4.38 (-0.11, 8.57) |
| -3.77 (-8.53, 0.97) | -1.17 (-4.13, 1.85) | -0.49 (-2.98, 2.06) | -0.58 (-4.15, 3.04) | 0.49 (-2.06, 3.22) | Discontinuation of Antipsychotics | -2.59 (-6.20, 0.89) | 0.35 (-2.32, 3.17) | -0.14 (-3.03, 2.76) | -11.98 (-15.14, -8.79) | 0.05 (-2.76, 2.86) | 0.12 (-2.37, 2.64) | -1.97 (-5.18, 1.30) | -0.40 (-3.19, 2.42) | 0.67 (-1.69, 3.09) | -1.63 (-5.82, 2.59) | -0.25 (-3.18, 2.81) | -1.67 (-4.32, 1.00) | 0.24 (-2.37, 2.97) | -2.60 (-8.27, 2.79) | 0.66 (-2.08, 3.43) | 4.87 (-0.05, 9.62) |
| -1.18 (-5.98, 3.68) | 1.44 (-1.72, 4.67) | 2.16 (-0.60, 4.91) | 2.05 (-1.70, 5.85) | 3.11 (0.39, 6.00) | 2.59 (-0.89, 6.20) | Electrical Current Therapy | 2.98 (0.13, 5.94) | 2.49 (-0.63, 5.57) | -9.37 (-12.73, -6.02) | 2.65 (-0.30, 5.68) | 2.75 (-0.04, 5.47) | 0.68 (-2.69, 3.97) | 2.23 (-0.79, 5.13) | 3.30 (0.73, 5.94) | 0.99 (-3.40, 5.45) | 2.35 (-0.83, 5.65) | 0.96 (-1.87, 3.83) | 2.86 (-0.01, 5.72) | -0.01 (-5.57, 5.54) | 3.28 (0.37, 6.25) | 7.49 (2.46, 12.36) |
| -4.17 (-8.42, 0.17) | -1.53 (-3.81, 0.65) | -0.82 (-2.52, 0.66) | -0.92 (-3.98, 2.09) | 0.13 (-1.05, 1.36) | -0.35 (-3.17, 2.32) | -2.98 (-5.94, -0.13) | Exercise | -0.50 (-2.69, 1.56) | -12.34 (-14.81, -9.91) | -0.31 (-2.37, 1.66) | -0.21 (-1.84, 1.18) | -2.32 (-4.80, 0.14) | -0.75 (-2.80, 1.09) | 0.33 (-1.05, 1.61) | -2.02 (-5.85, 1.84) | -0.61 (-2.86, 1.67) | -2.01 (-3.84, -0.35) | -0.11 (-1.96, 1.64) | -3.00 (-8.13, 2.17) | 0.32 (-1.72, 2.21) | 4.50 (-0.00, 8.80) |
| -3.67 (-8.08, 0.85) | -1.03 (-3.49, 1.50) | -0.32 (-2.19, 1.47) | -0.42 (-3.52, 2.79) | 0.64 (-1.29, 2.72) | 0.14 (-2.76, 3.03) | -2.49 (-5.57, 0.63) | 0.50 (-1.56, 2.69) | Light therapy | -11.84 (-14.45, -9.20) | 0.17 (-2.01, 2.38) | 0.29 (-1.57, 2.07) | -1.83 (-4.45, 0.86) | -0.26 (-2.44, 1.94) | 0.83 (-0.79, 2.48) | -1.49 (-5.39, 2.52) | -0.10 (-2.51, 2.39) | -1.52 (-3.52, 0.50) | 0.38 (-1.62, 2.43) | -2.48 (-7.80, 2.78) | 0.81 (-1.35, 3.06) | 5.01 (0.33, 9.44) |
| 8.19 (3.58, 12.91) | 10.81 (8.06, 13.59) | 11.53 (9.23, 13.72) | 11.42 (8.04, 14.73) | 12.49 (10.19, 14.84) | 11.98 (8.79, 15.14) | 9.37 (6.02, 12.73) | 12.34 (9.91, 14.81) | 11.84 (9.20, 14.45) | Massage | 12.05 (9.36, 14.55) | 12.14 (9.83, 14.26) | 10.03 (7.12, 12.91) | 11.61 (9.02, 14.01) | 12.67 (10.59, 14.71) | 10.35 (6.26, 14.47) | 11.72 (9.01, 14.53) | 10.33 (7.91, 12.67) | 12.23 (9.84, 14.56) | 9.37 (3.91, 14.77) | 12.65 (10.16, 15.12) | 16.87 (12.15, 21.41) |
| -3.87 (-8.12, 0.39) | -1.22 (-3.59, 1.17) | -0.51 (-2.20, 1.18) | -0.61 (-3.64, 2.41) | 0.46 (-1.35, 2.30) | -0.05 (-2.86, 2.76) | -2.65 (-5.68, 0.30) | 0.31 (-1.66, 2.37) | -0.17 (-2.38, 2.01) | -12.05 (-14.55, -9.36) | Mirtazapine | 0.10 (-1.57, 1.78) | -1.99 (-4.58, 0.59) | -0.45 (-2.49, 1.60) | 0.63 (-0.81, 2.16) | -1.68 (-5.53, 2.09) | -0.29 (-2.62, 2.15) | -1.70 (-3.58, 0.19) | 0.19 (-1.26, 1.72) | -2.68 (-7.87, 2.41) | 0.62 (-1.39, 2.68) | 4.84 (0.43, 8.98) |
| -3.96 (-8.09, 0.27) | -1.33 (-3.24, 0.78) | -0.62 (-1.74, 0.56) | -0.69 (-3.48, 2.14) | 0.34 (-0.85, 1.79) | -0.12 (-2.64, 2.37) | -2.75 (-5.47, 0.04) | 0.21 (-1.18, 1.84) | -0.29 (-2.07, 1.57) | -12.14 (-14.26, -9.83) | -0.10 (-1.78, 1.57) | Multidomain Therapy | -2.10 (-4.26, 0.15) | -0.55 (-2.16, 1.10) | 0.52 (-0.19, 1.42) | -1.80 (-5.34, 1.95) | -0.39 (-2.33, 1.74) | -1.81 (-3.16, -0.42) | 0.09 (-1.30, 1.57) | -2.78 (-7.81, 2.29) | 0.50 (-1.04, 2.27) | 4.74 (0.32, 8.88) |
| -1.80 (-6.43, 2.79) | 0.77 (-1.92, 3.53) | 1.47 (-0.73, 3.71) | 1.39 (-2.00, 4.79) | 2.46 (0.10, 4.86) | 1.97 (-1.30, 5.18) | -0.68 (-3.97, 2.69) | 2.32 (-0.14, 4.80) | 1.83 (-0.86, 4.45) | -10.03 (-12.91, -7.12) | 1.99 (-0.59, 4.58) | 2.10 (-0.15, 4.26) | Music Therapy | 1.54 (-0.98, 4.08) | 2.63 (0.58, 4.72) | 0.33 (-3.71, 4.38) | 1.69 (-1.00, 4.51) | 0.29 (-2.09, 2.65) | 2.20 (-0.23, 4.66) | -0.67 (-6.04, 4.65) | 2.62 (0.15, 5.18) | 6.82 (2.03, 11.28) |
| -3.40 (-7.68, 0.99) | -0.76 (-3.06, 1.61) | -0.07 (-1.74, 1.58) | -0.14 (-3.27, 2.91) | 0.89 (-0.82, 2.81) | 0.40 (-2.42, 3.19) | -2.23 (-5.13, 0.79) | 0.75 (-1.09, 2.80) | 0.26 (-1.94, 2.44) | -11.61 (-14.01, -9.02) | 0.45 (-1.60, 2.49) | 0.55 (-1.10, 2.16) | -1.54 (-4.08, 0.98) | Occupational Therapy | 1.09 (-0.33, 2.56) | -1.23 (-5.01, 2.60) | 0.16 (-2.16, 2.55) | -1.26 (-3.05, 0.55) | 0.64 (-1.22, 2.50) | -2.24 (-7.43, 2.91) | 1.07 (-0.89, 3.15) | 5.28 (0.74, 9.57) |
| -4.51 (-8.60, -0.37) | -1.85 (-3.66, -0.02) | -1.14 (-2.04, -0.34) | -1.23 (-3.95, 1.43) | -0.18 (-1.19, 0.91) | -0.67 (-3.09, 1.69) | -3.30 (-5.94, -0.73) | -0.33 (-1.61, 1.05) | -0.83 (-2.48, 0.79) | -12.67 (-14.71, -10.59) | -0.63 (-2.16, 0.81) | -0.52 (-1.42, 0.19) | -2.63 (-4.72, -0.58) | -1.09 (-2.56, 0.33) | Placebo | -2.33 (-5.86, 1.23) | -0.93 (-2.74, 0.92) | -2.34 (-3.51, -1.25) | -0.44 (-1.65, 0.78) | -3.32 (-8.26, 1.63) | -0.02 (-1.48, 1.43) | 4.17 (-0.20, 8.26) |
| -2.15 (-7.72, 3.34) | 0.45 (-3.50, 4.48) | 1.18 (-2.48, 4.83) | 1.09 (-3.47, 5.59) | 2.16 (-1.61, 5.95) | 1.63 (-2.59, 5.82) | -0.99 (-5.45, 3.40) | 2.02 (-1.84, 5.85) | 1.49 (-2.52, 5.39) | -10.35 (-14.47, -6.26) | 1.68 (-2.09, 5.53) | 1.80 (-1.95, 5.34) | -0.33 (-4.38, 3.71) | 1.23 (-2.60, 5.01) | 2.33 (-1.23, 5.86) | Pet/Robot-Assisted Therapy | 1.41 (-2.66, 5.46) | -0.02 (-3.80, 3.63) | 1.90 (-1.94, 5.60) | -0.99 (-7.10, 5.28) | 2.30 (-1.53, 6.14) | 6.51 (0.83, 11.93) |
| -3.55 (-8.04, 0.92) | -0.92 (-3.51, 1.61) | -0.23 (-2.32, 1.73) | -0.33 (-3.63, 2.94) | 0.75 (-1.36, 2.89) | 0.25 (-2.81, 3.18) | -2.35 (-5.65, 0.83) | 0.61 (-1.67, 2.86) | 0.10 (-2.39, 2.51) | -11.72 (-14.53, -9.01) | 0.29 (-2.15, 2.62) | 0.39 (-1.74, 2.33) | -1.69 (-4.51, 1.00) | -0.16 (-2.55, 2.16) | 0.93 (-0.92, 2.74) | -1.41 (-5.46, 2.66) | Psychosocial Therapy | -1.42 (-3.62, 0.70) | 0.47 (-1.77, 2.66) | -2.40 (-7.60, 2.90) | 0.91 (-1.44, 3.23) | 5.12 (0.47, 9.57) |
| -2.13 (-6.32, 2.15) | 0.48 (-1.61, 2.70) | 1.19 (-0.18, 2.60) | 1.12 (-1.85, 4.06) | 2.15 (0.69, 3.80) | 1.67 (-1.00, 4.32) | -0.96 (-3.83, 1.87) | 2.01 (0.35, 3.84) | 1.52 (-0.50, 3.52) | -10.33 (-12.67, -7.91) | 1.70 (-0.19, 3.58) | 1.81 (0.42, 3.16) | -0.29 (-2.65, 2.09) | 1.26 (-0.55, 3.05) | 2.34 (1.25, 3.51) | 0.02 (-3.63, 3.80) | 1.42 (-0.70, 3.62) | Reminiscence Therapy | 1.89 (0.28, 3.60) | -0.97 (-5.98, 4.14) | 2.32 (0.57, 4.18) | 6.52 (2.03, 10.78) |
| -4.08 (-8.23, 0.21) | -1.42 (-3.60, 0.82) | -0.70 (-2.21, 0.72) | -0.80 (-3.72, 2.09) | 0.26 (-1.31, 1.90) | -0.24 (-2.97, 2.37) | -2.86 (-5.72, 0.01) | 0.11 (-1.64, 1.96) | -0.38 (-2.43, 1.62) | -12.23 (-14.56, -9.84) | -0.19 (-1.72, 1.26) | -0.09 (-1.57, 1.30) | -2.20 (-4.66, 0.23) | -0.64 (-2.50, 1.22) | 0.44 (-0.78, 1.65) | -1.90 (-5.60, 1.94) | -0.47 (-2.66, 1.77) | -1.89 (-3.60, -0.28) | Sertraline | -2.88 (-7.94, 2.17) | 0.41 (-1.43, 2.30) | 4.63 (0.54, 8.49) |
| -1.14 (-3.79, 1.47) | 1.46 (-3.80, 6.73) | 2.17 (-2.84, 7.21) | 2.01 (-3.55, 7.73) | 3.14 (-1.87, 8.20) | 2.60 (-2.79, 8.27) | 0.01 (-5.54, 5.57) | 3.00 (-2.17, 8.13) | 2.48 (-2.78, 7.80) | -9.37 (-14.77, -3.91) | 2.68 (-2.41, 7.87) | 2.78 (-2.29, 7.81) | 0.67 (-4.65, 6.04) | 2.24 (-2.91, 7.43) | 3.32 (-1.63, 8.26) | 0.99 (-5.28, 7.10) | 2.40 (-2.90, 7.60) | 0.97 (-4.14, 5.98) | 2.88 (-2.17, 7.94) | Strength Exercise | 3.31 (-1.83, 8.41) | 7.46 (1.09, 13.97) |
| -4.47 (-8.79, -0.15) | -1.84 (-4.13, 0.51) | -1.12 (-2.86, 0.49) | -1.21 (-4.31, 1.79) | -0.18 (-1.90, 1.68) | -0.66 (-3.43, 2.08) | -3.28 (-6.25, -0.37) | -0.32 (-2.21, 1.72) | -0.81 (-3.06, 1.35) | -12.65 (-15.12, -10.16) | -0.62 (-2.68, 1.39) | -0.50 (-2.27, 1.04) | -2.62 (-5.18, -0.15) | -1.07 (-3.15, 0.89) | 0.02 (-1.43, 1.48) | -2.30 (-6.14, 1.53) | -0.91 (-3.23, 1.44) | -2.32 (-4.18, -0.57) | -0.41 (-2.30, 1.43) | -3.31 (-8.41, 1.83) | Tai Chi | 4.20 (-0.37, 8.54) |
| -8.65 (-14.37, -2.85) | -6.06 (-10.56, -1.34) | -5.33 (-9.55, -0.90) | -5.39 (-10.43, -0.37) | -4.38 (-8.57, 0.11) | -4.87 (-9.62, 0.05) | -7.49 (-12.36, -2.46) | -4.50 (-8.80, 0.00) | -5.01 (-9.44, -0.33) | -16.87 (-21.41, -12.15) | -4.84 (-8.98, -0.43) | -4.74 (-8.88, -0.32) | -6.82 (-11.28, -2.03) | -5.28 (-9.57, -0.74) | -4.17 (-8.26, 0.20) | -6.51 (-11.93, -0.83) | -5.12 (-9.57, -0.47) | -6.52 (-10.78, -2.03) | -4.63 (-8.49, -0.54) | -7.46 (-13.97, -1.09) | -4.20 (-8.54, 0.37) | Venlafaxine |

## B. Geriatric Depression Scale

| | | | | | | | | | | | | | | | | | | |
|---|---|---|---|---|---|---|---|---|---|---|---|---|---|---|---|---|---|---|
| Aerobic Exercise | -1.04 (-5.79, 3.61) | -0.32 (-6.31, 5.57) | 0.53 (-4.65, 5.75) | -1.57 (-8.30, 5.25) | 1.12 (-4.79, 7.04) | 0.51 (-5.25, 6.24) | 0.84 (-4.90, 6.68) | -1.48 (-7.30, 4.44) | -2.40 (-7.29, 2.58) | -1.88 (-7.52, 3.11) | 0.54 (-3.60, 4.71) | -0.27 (-5.37, 4.82) | -1.67 (-6.30, 2.91) | -4.39 (-10.26, 1.37) | 0.49 (-5.28, 6.20) | 0.26 (-3.75, 4.26) | -0.07 (-6.57, 6.44) | 3.17 (-4.99, 11.30) |
| 1.04 (-3.61, 5.79) | Behavioral Training | 0.74 (-4.08, 5.62) | 1.60 (-2.26, 5.49) | -0.48 (-6.22, 5.27) | 2.18 (-2.45, 6.79) | 1.56 (-3.07, 6.14) | 1.86 (-2.58, 6.46) | -0.40 (-5.02, 4.13) | -1.34 (-4.71, 2.11) | -0.83 (-5.09, 2.96) | 1.59 (-0.60, 3.78) | 0.77 (-2.86, 4.51) | -0.61 (-3.53, 2.30) | -3.33 (-7.92, 1.29) | 1.54 (-2.96, 6.14) | 1.32 (-4.81, 7.57) | 1.01 (-4.43, 6.52) | 4.26 (-3.02, 11.47) |
| 0.32 (-5.57, 6.31) | -0.74 (-5.62, 4.08) | Citalopram | 0.85 (-4.39, 6.11) | -1.21 (-8.15, 5.49) | 1.45 (-4.49, 7.32) | 0.79 (-4.99, 6.59) | 1.14 (-4.64, 6.99) | -1.15 (-7.08, 4.72) | -2.08 (-7.03, 2.94) | -1.55 (-7.29, 3.53) | 0.84 (-3.47, 5.12) | 0.05 (-5.20, 5.19) | -1.34 (-6.00, 3.27) | -4.07 (-10.03, 1.87) | 0.78 (-5.05, 6.63) | 0.58 (-6.50, 7.85) | 0.28 (-6.37, 6.86) | 3.52 (-4.68, 11.43) |
| -0.53 (-5.75, 4.65) | -1.60 (-5.49, 2.26) | -0.85 (-6.11, 4.39) | Cognitive Stimulated Training | -2.10 (-8.38, 4.15) | 0.60 (-4.69, 5.82) | -0.06 (-5.08, 5.05) | 0.27 (-4.74, 5.46) | -2.04 (-7.21, 3.14) | -2.94 (-7.01, 1.25) | -2.44 (-7.31, 1.87) | -0.04 (-3.20, 3.15) | -0.84 (-5.10, 3.42) | -2.21 (-5.93, 1.44) | -4.94 (-10.10, 0.16) | -0.07 (-5.19, 5.03) | -0.28 (-6.80, 6.33) | -0.59 (-6.53, 5.24) | 2.60 (-4.99, 10.07) |
| 1.57 (-5.25, 8.30) | 0.48 (-5.27, 6.22) | 1.21 (-5.49, 8.15) | 2.10 (-4.15, 8.38) | Electrical Current Therapy | 2.67 (-4.14, 9.39) | 2.05 (-4.61, 8.71) | 2.35 (-4.21, 9.08) | 0.12 (-6.55, 6.86) | -0.84 (-6.77, 5.06) | -0.35 (-6.94, 5.66) | 2.08 (-3.24, 7.38) | 1.25 (-4.84, 7.27) | -0.11 (-5.77, 5.55) | -2.85 (-9.50, 3.80) | 2.05 (-4.58, 8.59) | 1.83 (-6.01, 9.67) | 1.49 (-5.92, 8.88) | 4.71 (-4.08, 13.19) |
| -1.12 (-7.04, 4.79) | -2.18 (-6.79, 2.45) | -1.45 (-7.32, 4.49) | -0.60 (-5.82, 4.69) | -2.67 (-9.39, 4.14) | EGb 761 | -0.62 (-6.43, 4.98) | -0.27 (-6.04, 5.45) | -2.58 (-8.42, 3.19) | -3.52 (-8.31, 1.40) | -3.00 (-8.60, 2.07) | -0.57 (-4.71, 3.52) | -1.41 (-6.42, 3.58) | -2.78 (-7.36, 1.66) | -5.49 (-11.28, 0.31) | -0.65 (-6.37, 5.02) | -0.85 (-7.96, 6.37) | -1.17 (-7.69, 5.25) | 2.04 (-5.84, 10.05) |
| -0.51 (-6.24, 5.25) | -1.56 (-6.14, 3.07) | -0.79 (-6.59, 4.99) | 0.06 (-5.05, 5.08) | -2.05 (-8.71, 4.61) | 0.62 (-4.98, 6.43) | Exercise | 0.32 (-5.28, 6.04) | -1.96 (-7.60, 3.72) | -2.90 (-7.63, 1.96) | -2.36 (-7.94, 2.53) | 0.04 (-4.00, 4.04) | -0.76 (-5.76, 4.19) | -2.16 (-6.56, 2.20) | -4.88 (-10.67, 0.72) | -0.01 (-5.67, 5.71) | -0.22 (-7.29, 6.87) | -0.57 (-6.88, 5.93) | 2.68 (-5.30, 10.61) |
| -0.84 (-6.68, 4.90) | -1.86 (-6.46, 2.58) | -1.14 (-6.99, 4.64) | -0.27 (-5.46, 4.74) | -2.35 (-9.08, 4.21) | 0.27 (-5.45, 6.04) | -0.32 (-6.04, 5.28) | Hormone Therapy | -2.30 (-8.08, 3.37) | -3.20 (-7.95, 1.52) | -2.68 (-8.28, 2.12) | -0.29 (-4.28, 3.68) | -1.10 (-6.02, 3.73) | -2.49 (-6.94, 1.91) | -5.22 (-10.93, 0.44) | -0.35 (-6.06, 5.26) | -0.58 (-7.57, 6.39) | -0.88 (-7.26, 5.55) | 2.36 (-5.69, 10.19) |
| 1.48 (-4.44, 7.30) | 0.40 (-4.13, 5.02) | 1.15 (-4.72, 7.08) | 2.04 (-3.14, 7.21) | -0.12 (-6.86, 6.55) | 2.58 (-3.19, 8.42) | 1.96 (-3.72, 7.60) | 2.30 (-3.37, 8.08) | Psychosoical Therapy | -0.91 (-5.79, 3.96) | -0.39 (-5.95, 4.57) | 2.00 (-2.03, 6.10) | 1.19 (-3.77, 6.15) | -0.19 (-4.70, 4.21) | -2.91 (-8.63, 2.76) | 1.95 (-3.74, 7.71) | 1.74 (-5.21, 8.78) | 1.39 (-4.95, 7.84) | 4.62 (-3.24, 12.59) |
| 2.40 (-2.58, 7.29) | 1.34 (-2.11, 4.71) | 2.08 (-2.94, 7.03) | 2.94 (-1.25, 7.01) | 0.84 (-5.06, 6.77) | 3.52 (-1.40, 8.31) | 2.90 (-1.96, 7.63) | 3.20 (-1.52, 7.95) | 0.91 (-3.96, 5.79) | Multidomain Therapy | 0.49 (-3.98, 4.42) | 2.93 (0.26, 5.52) | 2.11 (-1.80, 5.90) | 0.73 (-2.58, 3.91) | -2.00 (-6.86, 2.75) | 2.87 (-1.95, 7.63) | 2.66 (-3.75, 9.01) | 2.34 (-3.27, 7.95) | 5.56 (-1.89, 12.96) |
| 1.88 (-3.11, 7.52) | 0.83 (-2.96, 5.09) | 1.55 (-3.53, 7.29) | 2.44 (-1.87, 7.31) | 0.35 (-5.66, 6.94) | 3.00 (-2.07, 8.60) | 2.36 (-2.53, 7.94) | 2.68 (-2.12, 8.28) | 0.39 (-4.57, 5.95) | -0.49 (-4.42, 3.98) | Music Therapy | 2.42 (-0.69, 6.02) | 1.59 (-2.57, 6.33) | 0.21 (-3.36, 4.36) | -2.52 (-7.41, 3.08) | 2.35 (-2.59, 7.85) | 2.13 (-4.12, 9.15) | 1.84 (-4.04, 8.25) | 5.06 (-2.36, 12.86) |
| -0.54 (-4.71, 3.60) | -1.59 (-3.78, 0.60) | -0.84 (-5.12, 3.47) | 0.04 (-3.15, 3.20) | -2.08 (-7.38, 3.24) | 0.57 (-3.52, 4.71) | -0.04 (-4.04, 4.00) | 0.29 (-3.68, 4.28) | -2.00 (-6.10, 2.03) | -2.93 (-5.52, -0.26) | -2.42 (-6.02, 0.69) | Placebo | -0.82 (-3.76, 2.05) | -2.20 (-4.12, -0.31) | -4.92 (-8.92, -0.90) | -0.05 | -0.26 (-6.02, 5.57) | -0.58 (-5.57, 4.46) | 2.64 (-4.19, 9.49) |
| 0.27 (-4.82, 5.37) | -0.77 (-4.51, 2.86) | -0.05 (-5.19, 5.20) | 0.84 (-3.42, 5.10) | -1.25 (-7.27, 4.84) | 1.41 (-3.58, 6.42) | 0.76 (-4.19, 5.76) | 1.10 (-3.73, 6.02) | -1.19 (-6.15, 3.77) | -2.11 (-5.90, 1.80) | -1.59 (-6.33, 2.57) | 0.82 (-2.05, 3.76) | Psychosocial Therapy | -1.38 (-4.85, 2.10) | -4.11 (-9.03, 0.86) | 0.75 (-4.15, 5.82) | 0.55 (-5.95, 7.02) | 0.21 (-5.57, 6.05) | 3.47 (-4.00, 10.91) |
| 1.67 (-2.91, 6.30) | 0.61 (-2.30, 3.53) | 1.34 (-3.27, 6.00) | 2.21 (-1.44, 5.93) | 0.11 (-5.55, 5.77) | 2.78 (-1.66, 7.36) | 2.16 (-2.20, 6.56) | 2.49 (-1.91, 6.94) | 0.19 (-4.21, 4.70) | -0.73 (-3.91, 2.58) | -0.21 (-4.36, 3.36) | 2.20 (0.31, 4.12) | 1.38 (-2.10, 4.85) | Reminiscence Therapy | -2.74 (-7.19, 1.86) | 2.15 (-2.22, 6.57) | 1.93 (-4.15, 8.06) | 1.65 (-3.75, 6.98) | 4.84 (-2.27, 11.94) |
| 4.39 (-1.37, 10.26) | 3.33 (-1.29, 7.92) | 4.07 (-1.87, 10.03) | 4.94 (-0.16, 10.10) | 2.85 (-3.80, 9.50) | 5.49 (-0.31, 11.28) | 4.88 (-0.72, 10.67) | 5.22 (-0.44, 10.93) | 2.91 (-2.76, 8.63) | 2.00 (-2.75, 6.86) | 2.52 (-3.08, 7.41) | 4.92 (0.90, 8.92) | 4.11 (-0.86, 9.03) | 2.74 (-1.86, 7.19) | Rivastigmine | 4.87 (-0.81, 10.57) | 4.64 (-2.36, 11.74) | 4.33 (-2.08, 10.76) | 7.58 (-0.40, 15.27) |
| -0.49 (-6.20, 5.28) | -1.54 (-6.14, 2.96) | -0.78 (-6.63, 5.05) | 0.07 (-5.03, 5.19) | -2.05 (-8.59, 4.58) | 0.65 (-5.02, 6.37) | 0.01 (-5.71, 5.67) | 0.35 (-5.26, 6.06) | -1.95 (-7.71, 3.74) | -2.87 (-7.63, 1.95) | -2.35 (-7.85, 2.59) | 0.05 (-3.95, 4.05) | -0.75 (-5.82, 4.15) | -2.15 (-6.57, 2.22) | -4.87 (-10.57, 0.81) | Sertraline | -0.23 (-7.19, 6.90) | -0.52 (-7.05, 5.85) | 2.72 (-3.04, 8.33) |
| -0.26 (-4.26, 3.75) | -1.32 (-7.57, 4.81) | -0.58 (-7.85, 6.50) | 0.28 (-6.33, 6.80) | -1.83 (-9.67, 6.01) | 0.85 (-6.37, 7.96) | 0.22 (-6.87, 7.29) | 0.58 (-6.39, 7.57) | -1.74 (-8.78, 5.21) | -2.66 (-9.01, 3.75) | -2.13 (-9.15, 4.12) | 0.26 (-5.57, 6.02) | -0.55 (-7.02, 5.95) | -1.93 (-8.06, 4.15) | -4.64 (-11.74, 2.36) | 0.23 (-6.90, 7.19) | Strength Exercise | -0.32 (-7.93, 7.42) | 2.92 (-6.25, 11.78) |
| 0.07 (-6.44, 6.57) | -1.01 (-6.52, 4.43) | -0.28 (-6.86, 6.37) | 0.59 (-5.24, 6.53) | -1.49 (-8.88, 5.92) | 1.17 (-5.25, 7.69) | 0.57 (-5.93, 6.88) | 0.88 (-5.55, 7.26) | -1.39 (-7.84, 4.95) | -2.34 (-7.95, 3.27) | -1.84 (-8.25, 4.04) | 0.58 (-4.46, 5.57) | -0.21 (-6.05, 5.57) | -1.65 (-6.98, 3.75) | -4.33 (-10.76, 2.08) | 0.52 (-5.85, 7.05) | 0.32 (-7.42, 7.93) | Tai Chi | 3.19 (-5.30, 11.75) |
| -3.17 (-11.30, 4.99) | -4.26 (-11.47, 3.02) | -3.52 (-11.43, 4.68) | -2.60 (-10.07, 4.99) | -4.71 (-13.19, 4.08) | -2.04 (-10.05, 5.84) | -2.68 (-10.61, 5.30) | -2.36 (-10.19, 5.69) | -4.62 (-12.59, 3.24) | -5.56 (-12.96, 1.89) | -5.06 (-12.86, 2.36) | -2.64 (-9.49, 4.19) | -3.47 (-10.91, 4.00) | -4.84 (-11.94, 2.27) | -7.58 (-15.27, 0.40) | -2.72 (-8.33, 3.04) | -2.92 (-11.78, 6.25) | -3.19 (-11.75, 5.30) | Venlafaxine |

## C. Hamilton Depression Rating Scale

| | | | | | | | | | | | | | | | |
|---|---|---|---|---|---|---|---|---|---|---|---|---|---|---|---|
| Amitriptyline | -12.58 (-20.16, -5.07) | -12.53 (-20.95, -4.13) | -12.99 (-21.17, -4.82) | -8.87 (-16.25, -1.36) | -10.66 (-18.03, -3.01) | -8.40 (-16.17, -1.04) | -5.42 (-9.79, -1.04) | -9.35 (-17.79, -1.07) | -7.07 (-14.55, 0.39) | -11.38 (-19.58, -3.25) | -19.19 (-28.52, -10.04) | -7.79 (-14.61, -0.96) | -10.63 (-18.59, -2.53) | -13.58 (-21.97, -5.09) | -11.26 (-20.43, -1.98) |
| 12.58 (5.07, 20.16) | Behavioral Training | 0.03 (-5.84, 5.97) | -0.42 (-5.94, 5.27) | 3.72 (-0.64, 8.14) | 1.94 (-2.50, 6.54) | 4.17 (-0.50, 8.50) | 7.16 (0.94, 13.38) | 3.19 (-2.74, 9.17) | 5.51 (0.97, 10.16) | 1.23 (-4.36, 6.59) | -6.63 (-13.65, 0.41) | 4.78 (1.51, 8.06) | 1.97 (-3.20, 7.18) | -0.99 (-6.90, 4.88) | 1.30 (-5.55, 8.25) |
| 12.53 (4.13, 20.95) | -0.03 (-5.97, 5.84) | Citalopram | -0.45 (-7.12, 6.33) | 3.70 (-2.08, 9.45) | 1.89 (-3.83, 7.93) | 4.15 (-1.83, 9.73) | 7.16 (-0.10, 14.25) | 3.18 (-3.87, 10.23) | 5.50 (-0.38, 11.23) | 1.18 (-5.43, 7.70) | -6.64 (-14.68, 1.23) | 4.75 (-0.22, 9.62) | 1.94 (-4.45, 8.25) | -1.02 (-7.93, 5.96) | 1.29 (-6.57, 9.26) |
| 12.99 (4.82, 21.17) | 0.42 (-5.27, 5.94) | 0.45 (-6.33, 7.12) | Cognitive Stimulated Training | 4.14 (-1.25, 9.46) | 2.35 (-3.08, 7.98) | 4.58 (-1.14, 9.86) | 7.55 (0.66, 14.65) | 3.61 (-3.14, 10.38) | 5.94 (0.40, 11.43) | 1.64 (-4.82, 7.94) | -6.24 (-13.86, 1.41) | 5.19 (0.63, 9.62) | 2.37 (-3.80, 8.52) | -0.57 (-7.24, 5.95) | 1.74 (-5.83, 9.20) |
| 8.87 (1.36, 16.25) | -3.72 (-8.14, 0.64) | -3.70 (-9.45, 2.08) | -4.14 (-9.46, 1.25) | Donepezil | -1.80 (-4.95, 1.54) | 0.46 (-3.98, 4.47) | 3.45 (-2.64, 9.42) | -0.04 (-5.21, 5.26) | 1.80 (-2.52, 6.05) | -2.51 (-7.79, 2.62) | -10.38 (-17.24, -3.48) | 1.05 (-1.81, 3.92) | -1.76 (-6.74, 3.16) | -4.73 (-10.39, 0.93) | -2.40 (-9.11, 4.38) |
| 10.66 (3.01, 18.03) | -1.94 (-6.54, 2.50) | -1.89 (-7.93, 3.83) | -2.35 (-7.98, 3.08) | 1.80 (-1.54, 4.95) | EGb 761 | 2.26 (-2.61, 6.34) | 5.26 (-1.02, 11.23) | 1.26 (-4.73, 7.01) | 3.60 (-0.99, 8.00) | -0.71 (-6.37, 4.54) | -8.59 (-15.49, -1.67) | 2.86 (-0.41, 5.90) | 0.04 (-5.26, 5.21) | -2.92 (-8.83, 2.88) | -0.60 (-7.52, 6.23) |
| 8.40 (1.04, 16.17) | -4.17 (-8.50, 0.50) | -4.15 (-9.73, 1.83) | -4.58 (-9.86, 1.14) | -0.46 (-4.47, 3.98) | -2.26 (-6.34, 2.61) | Exercise | 3.02 (-2.96, 9.38) | -0.98 (-6.60, 5.01) | 1.34 (-2.96, 6.05) | -2.95 (-7.33, 1.58) | -10.82 (-17.69, -3.57) | 0.61 (-2.29, 3.96) | -2.20 (-7.20, 3.11) | -5.12 (-10.84, 0.77) | -2.79 (-9.54, 4.15) |
| 5.42 (1.04, 9.79) | -7.16 (-13.38, -0.94) | -7.16 (-14.25, 0.10) | -7.55 (-14.65, -0.66) | -3.45 (-9.42, 2.64) | -5.26 (-11.23, 1.02) | -3.02 (-9.38, 2.96) | Fluoxetine | -3.96 (-11.23, 3.21) | -1.67 (-7.84, 4.57) | -5.97 (-12.91, 0.98) | -13.81 (-22.09, -5.55) | -2.40 (-7.68, 2.91) | -5.22 (-11.96, 1.50) | -8.16 (-15.45, -0.86) | -5.85 (-13.81, 2.33) |
| 9.35 (1.07, 17.79) | -3.19 (-9.17, 2.74) | -3.18 (-10.23, 3.87) | -3.61 (-10.38, 3.14) | 0.53 (-5.17, 6.33) | -1.26 (-7.01, 4.73) | 0.98 (-5.01, 6.60) | 3.96 (-3.21, 11.23) | Hormone Therapy | 2.32 (-3.55, 8.17) | -1.96 (-8.65, 4.58) | -9.83 (-17.77, -1.87) | 1.60 (-3.36, 6.60) | -1.22 (-7.64, 5.09) | -4.18 (-11.24, 2.76) | -1.83 (-9.83, 5.98) |
| 7.07 (-0.39, 14.55) | -5.51 (-10.16, -0.97) | -5.50 (-11.23, 0.38) | -5.94 (-11.43, -0.40) | -1.80 (-6.05, 2.52) | -3.60 (-8.00, 0.99) | -1.34 (-6.05, 2.96) | 1.67 (-4.57, 7.84) | -2.32 (-8.17, 3.55) | Imipramine | -4.29 (-9.82, 1.03) | -12.16 (-19.12, -5.27) | -0.75 (-3.91, 2.49) | -3.55 (-8.73, 1.64) | -6.56 (-12.32, -0.65) | -4.21 (-10.94, 2.71) |
| 11.38 (3.25, 19.58) | -1.23 (-6.59, 4.36) | -1.18 (-7.70, 5.43) | -1.64 (-7.94, 4.82) | 2.51 (-2.62, 7.79) | 0.71 (-4.54, 6.37) | 2.95 (-1.58, 7.33) | 5.97 (-0.98, 12.91) | 1.96 (-4.58, 8.65) | 4.29 (-1.03, 9.82) | Multidomain Therapy | -7.83 (-15.45, -0.13) | 3.55 (-0.84, 8.06) | 0.76 (-5.23, 6.81) | -2.21 (-8.69, 4.51) | 0.12 (-7.35, 7.81) |
| 19.19 (10.04, 28.52) | 6.63 (-0.41, 13.65) | 6.64 (-1.23, 14.68) | 6.24 (-1.41, 13.86) | 10.38 (3.48, 17.24) | 8.59 (1.67, 15.49) | 10.82 (3.57, 17.69) | 13.81 (5.55, 22.09) | 9.83 (1.87, 17.77) | 12.16 (5.27, 19.12) | 7.83 (0.13, 15.45) | Music Therapy | 11.43 (5.12, 17.66) | 8.61 (1.19, 16.03) | 5.65 (-2.44, 13.62) | 7.97 (-1.01, 16.86) |
| 7.79 (0.96, 14.61) | -4.78 (-8.06, -1.51) | -4.75 (-9.62, 0.22) | -5.19 (-9.62, -0.63) | -1.05 (-3.92, 1.81) | -2.86 (-5.90, 0.41) | -0.61 (-3.96, 2.29) | 2.40 (-2.91, 7.68) | -1.60 (-6.60, 3.36) | 0.75 (-2.49, 3.91) | -3.55 (-8.06, 0.84) | -11.43 (-17.66, -5.12) | Placebo | -2.80 (-6.92, 1.27) | -5.79 (-10.73, -0.89) | -3.46 (-9.49, 2.63) |
| 10.63 (2.53, 18.59) | -1.97 (-7.18, 3.20) | -1.94 (-8.25, 4.45) | -2.37 (-8.52, 3.80) | 1.76 (-3.16, 6.74) | -0.04 (-5.21, 5.26) | 2.20 (-3.11, 7.20) | 5.22 (-1.50, 11.96) | 1.22 (-5.09, 7.64) | 3.55 (-1.64, 8.73) | -0.76 (-6.81, 5.23) | -8.61 (-16.03, -1.19) | 2.80 (-1.27, 6.92) | Rivastigmine | -2.96 (-9.33, 3.45) | -0.61 (-7.87, 6.75) |
| 13.58 (5.09, 21.97) | 0.99 (-4.88, 6.90) | 1.02 (-5.96, 7.93) | 0.57 (-5.95, 7.24) | 4.73 (-0.93, 10.39) | 2.92 (-2.88, 8.83) | 5.12 (-0.77, 10.84) | 8.16 (0.86, 15.45) | 4.18 (-2.76, 11.24) | 6.56 (0.65, 12.32) | 2.21 (-4.51, 8.69) | -5.65 (-13.62, 2.44) | 5.79 (0.89, 10.73) | 2.96 (-3.45, 9.33) | Sertraline | 2.34 (-1.28, 6.00) |
| 11.26 (1.98, 20.43) | -1.30 (-8.25, 5.55) | -1.29 (-9.26, 6.57) | -1.74 (-9.20, 5.83) | 2.40 (-4.38, 9.11) | 0.60 (-6.23, 7.52) | 2.79 (-4.15, 9.54) | 5.85 (-2.33, 13.81) | 1.83 (-5.98, 9.83) | 4.21 (-2.71, 10.94) | -0.12 (-7.81, 7.35) | -7.97 (-16.86, 1.01) | 3.46 (-2.63, 9.49) | 0.61 (-6.75, 7.87) | -2.34 (-6.00, 1.28) | Venlafaxine |

## D. Mini-Mental State Examination

| | | | | | | | | | | | | | | | | | | | | | |
|---|---|---|---|---|---|---|---|---|---|---|---|---|---|---|---|---|---|---|---|---|---|
| Amitriptyline | -1.18 (-4.02, 1.70) | -0.93 (-3.97, 1.98) | 0.36 (-3.22, 3.88) | 0.61 (-2.19, 3.45) | 3.27 (-0.74, 7.38) | -0.37 (-3.39, 2.73) | -0.11 (-3.20, 2.93) | -1.99 (-5.54, 1.67) | -0.85 (-4.20, 2.51) | -1.41 (-5.09, 2.34) | -0.94 (-4.67, 2.79) | 0.27 (-3.57, 4.11) | -0.27 (-3.41, 2.71) | 2.06 (-1.98, 6.13) | 0.69 (-4.25, 5.58) | -0.79 (-3.47, 1.82) | -0.70 (-3.73, 2.41) | 0.51 (-2.43, 3.39) | 0.62 (-2.49, 3.67) | -2.34 (-5.98, 1.40) | -1.29 (-4.78, 2.18) |
| 1.18 (-1.70, 4.02) | Aripiprazole | 0.24 (-1.54, 1.92) | 1.53 (-1.02, 4.08) | 1.79 (0.39, 3.25) | 4.43 (1.23, 7.70) | 0.81 (-1.00, 2.66) | 1.05 (-0.86, 2.96) | -0.82 (-3.49, 1.84) | 0.32 (-2.04, 2.68) | -0.23 (-2.99, 2.60) | 0.23 (-2.57, 3.03) | 1.44 (-1.50, 4.41) | 0.89 (-1.00, 2.71) | 3.22 (0.01, 6.48) | 1.85 (-2.42, 6.08) | 0.36 (-0.70, 1.44) | 0.48 (-1.32, 2.33) | 1.68 (0.02, 3.31) | 1.79 (-0.03, 3.63) | -1.16 (-3.89, 1.56) | -0.11 (-2.65, 2.41) |
| 0.93 (-1.98, 3.97) | -0.24 (-1.92, 1.54) | Behavioral Training | 1.30 (-1.35, 4.01) | 1.55 (-0.02, 3.28) | 4.22 (0.88, 7.59) | 0.58 (-1.38, 2.66) | 0.82 (-1.22, 2.95) | -1.06 (-3.84, 1.80) | 0.09 (-2.41, 2.64) | -0.46 (-3.39, 2.55) | 0.00 (-2.86, 2.97) | 1.21 (-1.81, 4.37) | 0.66 (-1.38, 2.75) | 3.01 (-0.30, 6.42) | 1.61 (-2.73, 5.98) | 0.13 (-1.18, 1.52) | 0.24 (-1.75, 2.37) | 1.43 (-0.39, 3.34) | 1.56 (-0.43, 3.65) | -1.40 (-4.21, 1.44) | -0.35 (-2.96, 2.33) |
| -0.36 (-3.88, 3.22) | -1.53 (-4.08, 1.02) | -1.30 (-4.01, 1.35) | Citalopram | 0.26 (-2.25, 2.80) | 2.91 (-0.85, 6.69) | -0.71 (-3.43, 2.02) | -0.47 (-3.25, 2.34) | -2.36 (-5.73, 1.03) | -1.19 (-4.32, 1.91) | -1.76 (-5.19, 1.75) | -1.28 (-4.80, 2.13) | -0.08 (-3.68, 3.54) | -0.62 (-3.46, 2.12) | 1.71 (-2.11, 5.50) | 0.37 (-4.42, 5.04) | -1.16 (-3.49, 1.15) | -1.06 (-3.76, 1.74) | 0.15 (-2.49, 2.76) | 0.26 (-2.52, 3.04) | -2.70 (-6.07, 0.77) | -1.63 (-4.90, 1.57) |
| -0.61 (-3.45, 2.19) | -1.79 (-3.25, -0.39) | -1.55 (-3.28, 0.02) | -0.26 (-2.80, 2.25) | Cognitive Stimulated Training | 2.66 (-0.55, 5.82) | -0.97 (-2.76, 0.80) | -0.73 (-2.37, 0.85) | -2.61 (-5.30, 0.02) | -1.47 (-3.83, 0.82) | -2.04 (-4.73, 0.82) | -1.56 (-4.29, 1.21) | -0.36 (-3.28, 2.51) | -0.89 (-2.76, 0.84) | 1.44 (-1.70, 4.62) | 0.05 (-4.17, 4.29) | -1.42 (-2.39, -0.49) | -1.31 (-3.08, 0.45) | -0.12 (-1.71, 1.46) | 0.00 (-1.82, 1.77) | -2.94 (-5.62, -0.31) | -1.90 (-4.41, 0.56) |
| -3.27 (-7.38, 0.74) | -4.43 (-7.70, -1.23) | -4.22 (-7.59, -0.88) | -2.91 (-6.69, 0.85) | -2.66 (-5.82, 0.55) | Electrical Current Therapy | -3.62 (-7.03, -0.24) | -3.40 (-6.84, 0.03) | -5.27 (-9.16, -1.30) | -4.12 (-7.82, -0.42) | -4.69 (-8.65, -0.68) | -4.21 (-8.23, -0.21) | -3.02 (-7.10, 1.10) | -3.55 (-6.97, -0.22) | -1.21 (-5.51, 3.08) | -2.58 (-7.76, 2.44) | -4.08 (-7.11, -1.07) | -3.98 (-7.32, -0.56) | -2.77 (-6.08, 0.50) | -2.65 (-6.08, 0.71) | -5.58 (-9.58, -1.72) | -4.57 (-8.42, -0.77) |
| 0.37 (-2.73, 3.39) | -0.81 (-2.66, 1.00) | -0.58 (-2.66, 1.38) | 0.71 (-2.02, 3.43) | 0.97 (-0.80, 2.76) | 3.62 (0.24, 7.03) | EGb 761 | 0.23 (-1.93, 2.42) | -1.64 (-4.49, 1.23) | -0.50 (-3.08, 2.11) | -1.05 (-3.99, 1.97) | -0.58 (-3.56, 2.39) | 0.62 (-2.47, 3.74) | 0.08 (-2.11, 2.15) | 2.42 (-0.93, 5.79) | 1.03 (-3.37, 5.38) | -0.45 (-1.94, 1.04) | -0.34 (-2.43, 1.81) | 0.86 (-1.11, 2.81) | 0.98 (-0.94, 2.84) | -1.97 (-4.88, 0.91) | -0.93 (-3.67, 1.76) |
| 0.11 (-2.93, 3.20) | -1.05 (-2.96, 0.86) | -0.82 (-2.95, 1.22) | 0.47 (-2.34, 3.25) | 0.73 (-0.85, 2.37) | 3.40 (-0.03, 6.84) | -0.23 (-2.42, 1.93) | Exercise | -1.86 (-4.82, 1.08) | -0.73 (-3.32, 1.88) | -1.28 (-4.26, 1.76) | -0.81 (-3.86, 2.23) | 0.38 (-2.76, 3.61) | -0.17 (-2.33, 1.92) | 2.20 (-1.21, 5.59) | 0.79 (-3.59, 5.23) | -0.69 (-2.26, 0.88) | -0.58 (-2.72, 1.59) | 0.62 (-1.38, 2.60) | 0.74 (-1.42, 2.91) | -2.20 (-5.21, 0.71) | -1.17 (-3.99, 1.60) |
| 1.99 (-1.67, 5.54) | 0.82 (-1.84, 3.49) | 1.06 (-1.80, 3.84) | 2.36 (-1.03, 5.73) | 2.61 (-0.02, 5.30) | 5.27 (1.30, 9.16) | 1.64 (-1.23, 4.49) | 1.86 (-1.08, 4.82) | Fluoxetine | 1.15 (-2.08, 4.41) | 0.60 (-2.98, 4.17) | 1.04 (-2.51, 4.61) | 2.25 (-1.46, 5.96) | 1.71 (-1.23, 4.58) | 4.03 (0.12, 8.03) | 2.66 (-2.12, 7.54) | 1.19 (-1.27, 3.65) | 1.30 (-1.54, 4.18) | 2.49 (-0.29, 5.30) | 2.60 (-0.30, 5.52) | -0.35 (-3.79, 3.13) | 0.70 (-2.70, 4.02) |
| 0.85 (-2.51, 4.20) | -0.32 (-2.68, 2.04) | -0.09 (-2.64, 2.41) | 1.19 (-1.91, 4.32) | 1.47 (-0.82, 3.83) | 4.12 (0.42, 7.82) | 0.50 (-2.11, 3.08) | 0.73 (-1.88, 3.32) | -1.15 (-4.41, 2.08) | Hormone Therapy | -0.56 (-3.82, 2.79) | -0.07 (-3.46, 3.29) | 1.10 (-2.38, 4.68) | 0.57 (-2.04, 3.15) | 2.92 (-0.77, 6.65) | 1.51 (-3.08, 6.24) | 0.04 (-2.08, 2.17) | 0.15 (-2.41, 2.79) | 1.35 (-1.13, 3.85) | 1.48 (-1.13, 4.03) | -1.48 (-4.78, 1.80) | -0.44 (-3.56, 2.66) |
| 1.41 (-2.34, 5.09) | 0.23 (-2.60, 2.99) | 0.46 (-2.55, 3.39) | 1.76 (-1.75, 5.19) | 2.04 (-0.82, 4.73) | 4.69 (0.68, 8.65) | 1.05 (-1.97, 3.99) | 1.28 (-1.76, 4.26) | -0.60 (-4.17, 2.98) | 0.56 (-2.79, 3.82) | Imipramine | 0.46 (-3.19, 4.11) | 1.65 (-2.17, 5.45) | 1.12 (-1.94, 4.05) | 3.46 (-0.55, 7.43) | 2.10 (-2.90, 6.87) | 0.61 (-2.04, 3.16) | 0.72 (-2.35, 3.70) | 1.92 (-1.05, 4.77) | 2.03 (-0.99, 4.95) | -0.92 (-4.65, 2.63) | 0.12 (-3.40, 3.54) |
| 0.94 (-2.79, 4.67) | -0.23 (-3.03, 2.57) | -0.00 (-2.97, 2.86) | 1.28 (-2.13, 4.80) | 1.56 (-1.21, 4.29) | 4.21 (0.21, 8.23) | 0.58 (-2.39, 3.56) | 0.81 (-2.23, 3.86) | -1.04 (-4.61, 2.51) | 0.07 (-3.29, 3.46) | -0.46 (-4.11, 3.19) | Light therapy | 1.19 (-2.58, 5.05) | 0.65 (-2.31, 3.59) | 3.01 (-0.99, 6.96) | 1.63 (-3.31, 6.49) | 0.13 (-2.47, 2.71) | 0.23 (-2.74, 3.24) | 1.45 (-1.48, 4.32) | 1.56 (-1.43, 4.52) | -1.40 (-4.94, 2.17) | -0.34 (-3.80, 3.07) |
| -0.27 (-4.11, 3.57) | -1.44 (-4.41, 1.50) | -1.21 (-4.37, 1.81) | 0.08 (-3.54, 3.68) | 0.36 (-2.51, 3.28) | 3.02 (-1.10, 7.10) | -0.62 (-3.74, 2.47) | -0.38 (-3.61, 2.76) | -2.25 (-5.96, 1.46) | -1.10 (-4.68, 2.38) | -1.65 (-5.45, 2.17) | -1.19 (-5.05, 2.58) | Massage | -0.54 (-3.72, 2.59) | 1.80 (-2.31, 5.88) | 0.42 (-4.58, 5.34) | -1.07 (-3.85, 1.68) | -0.96 (-4.09, 2.19) | 0.25 (-2.82, 3.26) | 0.34 (-2.75, 3.49) | -2.61 (-6.33, 1.10) | -1.55 (-5.11, 1.97) |
| 0.27 (-2.71, 3.41) | -0.89 (-2.71, 1.00) | -0.66 (-2.75, 1.38) | 0.62 (-2.12, 3.46) | 0.89 (-0.84, 2.76) | 3.55 (0.22, 6.97) | -0.08 (-2.15, 2.11) | 0.17 (-1.92, 2.33) | -1.71 (-4.58, 1.23) | -0.57 (-3.15, 2.04) | -1.12 (-4.05, 1.94) | -0.65 (-3.59, 2.31) | 0.54 (-2.59, 3.72) | Multidomain Therapy | 2.33 (-1.03, 5.84) | 0.95 (-3.44, 5.39) | -0.53 (-2.02, 1.04) | -0.42 (-2.52, 1.78) | 0.78 (-1.17, 2.79) | 0.89 (-1.21, 3.10) | -2.05 (-4.95, 0.88) | -1.01 (-3.70, 1.76) |
| -2.06 (-6.13, 1.98) | -3.22 (-6.48, -0.01) | -3.01 (-6.42, 0.30) | -1.71 (-5.50, 2.11) | -1.44 (-4.62, 1.70) | 1.21 (-3.08, 5.51) | -2.42 (-5.79, 0.93) | -2.20 (-5.59, 1.21) | -4.03 (-8.03, -0.12) | -2.92 (-6.65, 0.77) | -3.46 (-7.43, 0.55) | -3.01 (-6.96, 0.99) | -1.80 (-5.88, 2.31) | -2.33 (-5.84, 1.03) | Music Therapy | -1.36 (-6.52, 3.66) | -2.87 (-5.95, 0.13) | -2.76 (-6.13, 0.59) | -1.55 (-4.94, 1.72) | -1.45 (-4.85, 1.93) | -4.40 (-8.40, -0.43) | -3.33 (-7.17, 0.36) |
| -0.69 (-5.58, 4.25) | -1.85 (-6.08, 2.42) | -1.61 (-5.98, 2.73) | -0.37 (-5.04, 4.42) | -0.05 (-4.29, 4.17) | 2.58 (-2.44, 7.76) | -1.03 (-5.38, 3.37) | -0.79 (-5.23, 3.59) | -2.66 (-7.54, 2.12) | -1.51 (-6.24, 3.08) | -2.10 (-6.87, 2.90) | -1.63 (-6.49, 3.31) | -0.42 (-5.34, 4.58) | -0.95 (-5.39, 3.44) | 1.36 (-3.66, 6.52) | Occupational Therapy | -1.50 (-5.59, 2.64) | -1.37 (-5.75, 3.08) | -0.19 (-4.48, 4.16) | -0.07 (-4.41, 4.33) | -3.00 (-7.90, 1.84) | -1.97 (-6.69, 2.73) |
| 0.79 (-1.82, 3.47) | -0.36 (-1.44, 0.70) | -0.13 (-1.52, 1.18) | 1.16 (-1.15, 3.49) | 1.42 (0.49, 2.39) | 4.08 (1.07, 7.11) | 0.45 (-1.04, 1.94) | 0.69 (-0.88, 2.26) | -1.19 (-3.65, 1.27) | -0.04 (-2.17, 2.08) | -0.61 (-3.16, 2.04) | -0.13 (-2.71, 2.47) | 1.07 (-1.68, 3.85) | 0.53 (-1.04, 2.02) | 2.87 (-0.13, 5.95) | 1.50 (-2.64, 5.59) | Placebo | 0.10 (-1.36, 1.64) | 1.31 (0.04, 2.56) | 1.43 (-0.06, 2.91) | -1.53 (-4.02, 0.96) | -0.47 (-2.79, 1.80) |
| 0.70 (-2.41, 3.73) | -0.48 (-2.33, 1.32) | -0.24 (-2.37, 1.75) | 1.06 (-1.74, 3.76) | 1.31 (-0.45, 3.08) | 3.98 (0.56, 7.32) | 0.34 (-1.81, 2.43) | 0.58 (-1.59, 2.72) | -1.30 (-4.18, 1.54) | -0.15 (-2.79, 2.41) | -0.72 (-3.70, 2.35) | -0.23 (-3.24, 2.74) | 0.96 (-2.19, 4.09) | 0.42 (-1.78, 2.52) | 2.76 (-0.59, 6.13) | 1.37 (-3.08, 5.75) | -0.10 (-1.64, 1.36) | Psychosocial Therapy | 1.20 (-0.76, 3.14) | 1.32 (-0.86, 3.40) | -1.64 (-4.58, 1.28) | -0.58 (-3.37, 2.12) |
| -0.51 (-3.39, 2.43) | -1.68 (-3.31, -0.02) | -1.43 (-3.34, 0.39) | -0.15 (-2.76, 2.49) | 0.12 (-1.46, 1.71) | 2.77 (-0.50, 6.08) | -0.86 (-2.81, 1.11) | -0.62 (-2.60, 1.38) | -2.49 (-5.30, 0.29) | -1.35 (-3.85, 1.13) | -1.92 (-4.77, 1.05) | -1.45 (-4.32, 1.48) | -0.25 (-3.26, 2.82) | -0.78 (-2.79, 1.17) | 1.55 (-1.72, 4.94) | 0.19 (-4.16, 4.48) | -1.31 (-2.56, -0.04) | -1.20 (-3.14, 0.76) | Reminiscence Therapy | 0.12 (-1.86, 2.11) | -2.84 (-5.61, -0.02) | -1.80 (-4.40, 0.80) |
| -0.62 (-3.67, 2.49) | -1.79 (-3.63, 0.03) | -1.56 (-3.65, 0.43) | -0.26 (-3.04, 2.52) | -0.00 (-1.77, 1.82) | 2.65 (-0.71, 6.08) | -0.98 (-2.84, 0.94) | -0.74 (-2.91, 1.42) | -2.60 (-5.52, 0.30) | -1.48 (-4.03, 1.13) | -2.03 (-4.95, 0.99) | -1.56 (-4.52, 1.43) | -0.34 (-3.49, 2.75) | -0.89 (-3.10, 1.21) | 1.45 (-1.93, 4.85) | 0.07 (-4.33, 4.41) | -1.43 (-2.91, 0.06) | -1.32 (-3.40, 0.86) | -0.12 (-2.11, 1.86) | Rivastigmine | -2.95 (-5.88, -0.05) | -1.89 (-4.66, 0.84) |
| 2.34 (-1.40, 5.98) | 1.16 (-1.56, 3.89) | 1.40 (-1.44, 4.21) | 2.70 (-0.77, 6.07) | 2.94 (0.31, 5.62) | 5.58 (1.72, 9.58) | 1.97 (-0.91, 4.88) | 2.20 (-0.71, 5.21) | 0.35 (-3.13, 3.79) | 1.48 (-1.80, 4.78) | 0.92 (-2.63, 4.65) | 1.40 (-2.17, 4.94) | 2.61 (-1.10, 6.33) | 2.05 (-0.88, 4.95) | 4.40 (0.43, 8.40) | 3.00 (-1.84, 7.90) | 1.53 (-0.96, 4.02) | 1.64 (-1.28, 4.58) | 2.84 (0.02, 5.61) | 2.95 (0.05, 5.88) | Sertraline | 1.05 (-2.31, 4.44) |
| 1.29 (-2.18, 4.78) | 0.11 (-2.41, 2.65) | 0.35 (-2.33, 2.96) | 1.63 (-1.57, 4.90) | 1.90 (-0.56, 4.41) | 4.57 (0.77, 8.42) | 0.93 (-1.76, 3.67) | 1.17 (-1.60, 3.99) | -0.70 (-4.02, 2.70) | 0.44 (-2.66, 3.56) | -0.12 (-3.54, 3.40) | 0.34 (-3.07, 3.80) | 1.55 (-1.97, 5.11) | 1.01 (-1.76, 3.70) | 3.33 (-0.36, 7.17) | 1.97 (-2.73, 6.69) | 0.47 (-1.80, 2.79) | 0.58 (-2.12, 3.37) | 1.80 (-0.80, 4.40) | 1.89 (-0.84, 4.66) | -1.05 (-4.44, 2.31) | Tai Chi |

## E. Neuropsychiatric Inventory

| | | | | | | | | | | | | | | | | | |
|---|---|---|---|---|---|---|---|---|---|---|---|---|---|---|---|---|---|
| Aerobic Exercise | 4.26 (-2.13, 8.02) | -2.96 (-10.82, 1.97) | -1.18 (-7.88, 3.64) | 0.69 (-5.74, 4.45) | 4.73 (-1.18, 10.00) | 6.89 (1.05, 14.94) | -3.50 (-10.97, 2.09) | -2.25 (-8.54, 1.18) | 5.14 (-1.69, 9.07) | 8.22 (-1.53, 15.77) | 5.85 (-0.94, 9.29) | 6.12 (-4.74, 12.91) | 6.69 (0.78, 9.97) | 3.00 (-5.35, 6.98) | 6.79 (-0.67, 11.25) | 2.42 (-6.79, 6.80) | 7.20 (0.62, 10.83) |
| -4.26 (-8.02, 2.13) | Aripiprazole | -7.13 (-12.20, -2.20) | -5.57 (-8.72, -1.36) | -3.53 (-6.36, -0.21) | 1.03 (-1.78, 5.52) | 3.95 (-0.83, 9.97) | -6.98 (-12.43, -2.82) | -6.43 (-8.96, -3.81) | 1.26 (-2.37, 4.00) | 3.86 (-3.76, 10.92) | 1.64 (-1.44, 4.15) | 2.53 (-6.45, 9.03) | 2.51 (0.61, 4.78) | -1.10 (-6.86, 2.73) | 2.62 (-1.50, 6.70) | -1.33 (-8.38, 3.01) | 2.93 (0.03, 6.40) |
| 2.96 (-1.97, 10.82) | 7.13 (2.20, 12.20) | Analgesic Treatment | 1.75 (-3.41, 7.35) | 3.60 (-1.26, 8.67) | 8.46 (3.77, 14.21) | 11.25 (5.25, 18.48) | 0.61 (-6.72, 6.09) | 0.85 (-4.11, 5.42) | 9.15 (2.76, 13.07) | 11.11 (2.55, 19.59) | 8.95 (3.58, 13.46) | 9.95 (-0.06, 17.35) | 9.68 (5.26, 14.21) | 5.98 (-0.64, 11.87) | 9.70 (4.39, 15.45) | 5.71 (-2.76, 12.24) | 10.19 (5.10, 15.19) |
| 1.18 (-3.64, 7.88) | 5.57 (1.36, 8.72) | -1.75 (-7.35, 3.41) | Behavioral Training | 2.02 (-1.94, 5.55) | 6.33 (2.78, 11.17) | 9.22 (3.98, 15.58) | -1.62 (-7.79, 2.79) | -0.89 (-4.67, 2.11) | 6.71 (2.01, 9.85) | 9.43 (1.39, 16.65) | 7.16 (2.64, 10.02) | 7.84 (-1.45, 14.18) | 8.03 (4.71, 10.80) | 4.34 (-2.01, 8.79) | 8.10 (3.59, 12.53) | 4.19 (-3.71, 8.62) | 8.46 (4.41, 12.02) |
| -0.69 (-4.45, 5.74) | 3.53 (0.21, 6.36) | -3.60 (-8.67, 1.26) | -2.02 (-5.55, 1.94) | Citalopram | 4.69 (1.50, 9.05) | 7.55 (2.54, 13.39) | -3.35 (-9.27, 0.64) | -2.82 (-5.90, -0.45) | 4.89 (0.80, 7.67) | 7.32 (-0.08, 14.45) | 5.34 (1.28, 7.65) | 6.17 (-3.12, 12.30) | 5.98 (3.64, 8.34) | 2.39 (-3.21, 6.35) | 6.08 (2.10, 10.25) | 2.12 (-5.05, 6.58) | 6.56 (3.12, 9.88) |
| -4.73 (-10.00, 1.18) | -1.03 (-5.52, 1.78) | -8.46 (-14.21, -3.77) | -6.33 (-11.17, -2.78) | -4.69 (-9.05, -1.50) | Cognitive Stimulated Training | 2.39 (-3.24, 8.65) | -8.61 (-14.88, -4.11) | -7.77 (-11.69, -4.91) | -0.16 (-5.04, 2.89) | 3.54 (-5.71, 9.45) | 0.30 (-4.29, 3.20) | 1.33 (-8.44, 7.34) | 1.25 (-2.14, 3.78) | -2.47 (-8.76, 2.21) | 1.62 (-3.62, 5.12) | -3.12 (-10.72, 2.49) | 1.73 (-2.71, 5.04) |
| -6.89 (-14.94, -1.05) | -3.95 (-9.97, 0.83) | -11.25 (-18.48, -5.25) | -9.22 (-15.58, -3.98) | -7.55 (-13.39, -2.54) | -2.39 (-8.65, 3.24) | Discontinuation of Antipsychotics | -11.75 (-19.24, -5.49) | -10.62 (-16.21, -5.86) | -3.03 (-9.34, 1.96) | 0.11 (-9.12, 8.08) | -2.59 (-8.71, 2.18) | -2.70 (-12.11, 5.49) | -1.56 (-6.91, 2.97) | -5.25 (-12.92, -0.03) | -1.26 (-7.86, 4.06) | -5.94 (-14.82, -0.27) | -1.08 (-7.11, 3.93) |
| 3.50 (-2.09, 10.97) | 6.98 (2.82, 12.43) | -0.61 (-6.09, 6.72) | 1.62 (-2.79, 7.79) | 3.35 (-0.64, 9.27) | 8.61 (4.11, 14.88) | 11.75 (5.49, 19.24) | Donepezil | 0.47 (-2.98, 5.26) | 8.10 (3.26, 13.95) | 10.87 (3.26, 19.80) | 8.45 (4.14, 13.86) | 8.81 (0.11, 17.82) | 9.41 (5.78, 14.59) | 5.57 (-0.64, 12.27) | 9.12 (4.88, 16.18) | 5.07 (-2.13, 11.96) | 9.73 (5.78, 15.68) |
| 2.25 (-1.18, 8.54) | 6.43 (3.81, 8.96) | -0.85 (-5.42, 4.11) | 0.89 (-2.11, 4.67) | 2.82 (0.45, 5.90) | 7.77 (4.91, 11.69) | 10.62 (5.86, 16.21) | -0.47 (-5.26, 2.98) | EGb 761 | 7.83 (4.44, 10.33) | 10.07 (3.23, 17.52) | 8.12 (5.07, 10.29) | 9.02 (0.17, 15.27) | 8.86 (7.59, 10.59) | 5.17 (0.07, 9.21) | 8.86 (5.49, 12.96) | 4.90 (-1.71, 9.41) | 9.34 (6.63, 12.42) |
| -5.14 (-9.07, 1.69) | -1.26 (-4.00, 2.37) | -9.15 (-13.07, -2.76) | -6.71 (-9.85, -2.01) | -4.89 (-7.67, -0.80) | 0.16 (-2.89, 5.04) | 3.03 (-1.96, 9.34) | -8.10 (-13.95, -3.26) | -7.83 (-10.33, -4.44) | Exercise | 2.49 (-4.58, 10.22) | 0.17 (-2.78, 3.64) | 0.72 (-7.55, 8.38) | 1.25 (-1.11, 4.24) | -2.72 (-7.50, 2.39) | 1.00 (-4.27, 6.39) | -3.42 (-9.42, 2.66) | 1.52 (-1.30, 5.76) |
| -8.22 (-15.77, 1.53) | -3.86 (-10.92, 3.76) | -11.11 (-19.59, -2.55) | -9.43 (-16.65, -1.39) | -7.32 (-14.45, 0.08) | -3.54 (-9.45, 5.71) | -0.11 (-8.08, 9.12) | -10.87 (-19.80, -3.26) | -10.07 (-17.52, -3.23) | -2.49 (-10.22, 4.58) | Light therapy | -2.01 (-9.69, 4.88) | -1.68 (-12.94, 7.92) | -1.33 (-8.09, 5.83) | -5.22 (-14.18, 2.73) | -1.21 (-8.52, 6.58) | -5.24 (-15.53, 2.23) | -0.74 (-8.22, 6.61) |
| -5.85 (-9.29, 0.94) | -1.64 (-4.15, 1.44) | -8.95 (-13.46, -3.58) | -7.16 (-10.02, -2.64) | -5.34 (-7.65, -1.28) | -0.30 (-3.20, 4.29) | 2.59 (-2.18, 8.71) | -8.45 (-13.86, -4.14) | -8.12 (-10.29, -5.07) | -0.17 (-3.64, 2.78) | 2.01 (-4.88, 9.69) | Multidomain Therapy | 1.02 (-7.88, 7.50) | 0.67 (-0.66, 3.60) | -2.94 (-7.99, 1.61) | 0.74 (-2.69, 5.49) | -3.21 (-9.70, 1.71) | 1.23 (-1.29, 5.09) |
| -6.12 (-12.91, 4.74) | -2.53 (-9.03, 6.45) | -9.95 (-17.35, 0.06) | -7.84 (-14.18, 1.45) | -6.17 (-12.30, 3.12) | -1.33 (-7.34, 8.44) | 2.70 (-5.49, 12.11) | -8.81 (-17.82, -0.11) | -9.02 (-15.27, -0.17) | -0.72 (-8.38, 7.55) | 1.68 (-7.92, 12.94) | -1.02 (-7.50, 7.88) | Music Therapy | -1.50 (-9.92, 6.81) | -3.98 (-11.26, 5.54) | -0.29 (-6.94, 10.25) | -4.19 (-13.16, 5.89) | 0.31 (-6.02, 9.69) |
| -6.69 (-9.97, -0.78) | -2.51 (-4.78, -0.61) | -9.68 (-14.21, -5.26) | -8.03 (-10.80, -4.71) | -5.98 (-8.34, -3.64) | -1.25 (-3.78, 2.14) | 1.56 (-2.97, 6.91) | -9.41 (-14.59, -5.78) | -8.86 (-10.59, -7.59) | -1.25 (-4.24, 1.11) | 1.33 (-5.83, 8.09) | -0.67 (-3.60, 0.66) | 1.50(-6.81, 9.92) | Placebo | -3.61 (-8.81, -0.06) | 0.10 (-3.35, 3.49) | -3.86 (-10.71, 0.79) | 0.51 (-1.96, 2.89) |
| -3.00 (-6.98, 5.35) | 1.10 (-2.73, 6.86) | -5.98 (-11.87, 0.64) | -4.34 (-8.79, 2.01) | -2.39 (-6.35, 3.21) | 2.47 (-2.21, 8.76) | 5.25 (0.03, 12.92) | -5.57 (-12.27, 0.64) | -5.17 (-9.21, -0.07) | 2.72 (-2.39, 7.50) | 5.22 (-2.73, 14.18) | 2.94 (-1.61, 7.99) | 3.98 (-5.54, 11.26) | 3.61 (0.06, 8.81) | Psychosocial Therapy | 3.69 (-1.76, 9.90) | -0.28 (-7.48, 6.36) | 4.18 (-0.36, 9.76) |
| -6.79 (-11.25, 0.67) | -2.62 (-6.70, 1.50) | -9.70 (-15.45, -4.39) | -8.10 (-12.53, -3.59) | -6.08 (-10.25, -2.10) | -1.62 (-5.12, 3.62) | 1.26 (-4.06, 7.86) | -9.12 (-16.18, -4.88) | -8.86 (-12.96, -5.49) | -1.00 (-6.39, 4.27) | 1.21 (-6.58, 8.52) | -0.74 (-5.49, 2.69) | 0.29 (-10.25, 6.94) | -0.10 (-3.49, 3.35) | -3.69 (-9.90, 1.76) | Reminiscence Therapy | -3.96 (-11.98, 1.12) | 0.49 (-3.81, 4.47) |
| -2.42 (-6.80, 6.79) | 1.33 (-3.01, 8.38) | -5.71 (-12.24, 2.76) | -4.19 (-8.62, 3.71) | -2.12 (-6.58, 5.05) | 3.12 (-2.49, 10.72) | 5.94 (0.27, 14.82) | -5.07 (-11.96, 2.13) | -4.90 (-9.41, 1.71) | 3.42 (-2.66, 9.42) | 5.24 (-2.23, 15.53) | 3.21 (-1.71, 9.70) | 4.19 (-5.89, 13.16) | 3.86 (-0.79, 10.71) | 0.28 (-6.36, 7.48) | 3.96 (-1.12, 11.98) | Sertraline | 4.44 (-0.36, 11.68) |
| -7.20 (-10.83, -0.62) | -2.93 (-6.40, -0.03) | -10.19 (-15.19, -5.10) | -8.46 (-12.02, -4.41) | -6.56 (-9.88, -3.12) | -1.73 (-5.04, 2.71) | 1.08 (-3.93, 7.11) | -9.73 (-15.68, -5.78) | -9.34 (-12.42, -6.63) | -1.52 (-5.76, 1.30) | 0.74 (-6.61, 8.22) | -1.23 (-5.09, 1.29) | -0.31 (-9.69, 6.02) | -0.51 (-2.89, 1.96) | -4.18 (-9.76, 0.36) | -0.49 (-4.47, 3.81) | -4.44 (-11.68, 0.36) | Tai Chi |

## F. Cohen-Mansfeld Agitation Inventory

| | | | | | | | | |
|---|---|---|---|---|---|---|---|---|
| Aripiprazole | 1.59 (-7.45, 10.80) | 1.30 (-7.14, 10.11) | 7.02 (-4.88, 18.48) | -0.17 (-8.70, 8.55) | -0.61 (-8.66, 7.89) | 4.20 (-1.03, 9.56) | -6.01 (-16.68, 4.22) | 6.79 (-19.84, 23.88) |
| -1.59 (-10.80, 7.45) | Analgesic Treatment | -0.31 (-11.04, 9.88) | 5.09 (-7.79, 18.15) | -2.07 (-12.16, 8.30) | -2.41 (-12.03, 7.91) | 2.52 (-5.04, 10.08) | -7.96 (-19.69, 4.08) | 4.76 (-22.26, 23.69) |
| -1.30 (-10.11, 7.14) | 0.31 (-9.88, 11.04) | Citalopram | 5.46 (-6.99, 18.16) | -1.60 (-11.26, 8.32) | -2.13 (-10.88, 7.47) | 2.85 (-4.02, 9.52) | -7.47 (-18.97, 3.59) | 5.10 (-21.39, 23.33) |
| -7.02 (-18.48, 4.88) | -5.09 (-18.15, 7.79) | -5.46 (-18.16, 6.99) | Fluoxetine | -7.23 (-19.94, 5.87) | -7.66 (-19.84, 4.90) | -2.65 (-13.38, 7.81) | -13.20 (-27.00, 0.51) | -0.53 (-29.39, 20.56) |
| 0.17 (-8.55, 8.70) | 2.07 (-8.30, 12.16) | 1.60 (-8.32, 11.26) | 7.23 (-5.87, 19.94) | Light therapy | -0.58 (-9.57, 9.19) | 4.41 (-2.84, 11.27) | -6.26 (-16.86, 5.82) | 7.29 (-20.72, 25.72) |
| 0.61 (-7.89, 8.66) | 2.41 (-7.91, 12.03) | 2.13 (-7.47, 10.88) | 7.66 (-4.90, 19.84) | 0.58 (-9.19, 9.57) | Massage | 5.02 (-1.80, 10.70) | -5.28 (-17.78, 5.26) | 7.40 (-20.15, 25.06) |
| -4.20 (-9.56, 1.03) | -2.52 (-10.08, 5.04) | -2.85 (-9.52, 4.02) | 2.65 (-7.81, 13.38) | -4.41 (-11.27, 2.84) | -5.02 (-10.70, 1.80) | Placebo | -10.39 (-19.59, -1.18) | 2.52 (-23.08, 18.51) |
| 6.01 (-4.22, 16.68) | 7.96 (-4.08, 19.69) | 7.47 (-3.59, 18.97) | 13.20 (-0.51, 27.00) | 6.26 (-5.82, 16.86) | 5.28 (-5.26, 17.78) | 10.39 (1.18, 19.59) | Pet/Robot-Assisted Therapy | 13.01 (-16.27, 32.49) |
| -6.79 (-23.88, 19.84) | -4.76 (-23.69, 22.26) | -5.10 (-23.33, 21.39) | 0.53 (-20.56, 29.39) | -7.29 (-25.72, 20.72) | -7.40 (-25.06, 20.15) | -2.52 (-18.51, 23.08) | -13.01 (-32.49, 16.27) | Sertraline |
