# Supplementary material for: Comparative efficacy and acceptability of treatments for depressive symptoms in cognitive impairment: A systematic review and Bayesian network meta-analysis
Source: Front Aging Neurosci. 2022 Dec 12;14:1037414. doi: 10.3389/fnagi.2022.1037414 (PMC9790988; doi:10.3389/fnagi.2022.1037414)
Supplement: Supplementary material 1 — PRISMA NMA checklist. [file Data_Sheet_1.zip › Supplementary Material Presentation/Supplementary_11.pdf]

A. Cornell Scale for Depression

Drug	Rank 1	Rank 2	Rank 3	Rank 4	Rank 5	Rank 6	Rank 7	Rank 8	Rank 9	Rank 10	Rank 11	Rank 12	Rank 13	Rank 14	Rank 15	Rank 16	Rank 17	Rank 18	Rank 19	Rank 20	Rank 21	Rank 22
Aerobic Exercise	0	0.48	0.23	0.1	0.05	0.03	0.02	0.02	0.01	0.01	0.01	0.01	0	0	0	0	0	0	0	0.01	0	0
Aripiprazole	0	0.01	0.03	0.06	0.1	0.12	0.14	0.13	0.11	0.08	0.06	0.04	0.03	0.02	0.02	0.01	0.01	0.01	0.01	0.01	0.01	0
Behavioral Training	0	0	0	0	0	0.02	0.04	0.08	0.13	0.16	0.17	0.14	0.1	0.07	0.04	0.02	0.01	0.01	0	0	0	0
Citalopram	0	0.01	0.02	0.05	0.06	0.08	0.08	0.08	0.07	0.07	0.05	0.05	0.04	0.04	0.04	0.03	0.03	0.03	0.03	0.05	0.08	0.01
Cognitive stimulated training	0	0	0	0	0	0	0	0	0.01	0.01	0.03	0.04	0.05	0.08	0.1	0.11	0.12	0.13	0.12	0.12	0.08	0
Discontinuation of antipsychotics	0	0	0.01	0.02	0.03	0.04	0.05	0.06	0.06	0.06	0.06	0.06	0.06	0.05	0.05	0.05	0.05	0.04	0.05	0.07	0.13	0.01
Electrical Current Therapy	0	0.19	0.16	0.23	0.14	0.08	0.05	0.04	0.03	0.02	0.01	0.01	0.01	0.01	0	0	0	0	0	0	0	0
Exercise	0	0	0	0	0	0	0.01	0.01	0.02	0.03	0.04	0.06	0.07	0.09	0.1	0.1	0.1	0.09	0.09	0.09	0.08	0
Light therapy	0	0	0	0.01	0.01	0.03	0.05	0.07	0.08	0.09	0.09	0.08	0.07	0.07	0.06	0.06	0.05	0.04	0.04	0.04	0.04	0
Massage	1	0	0	0	0	0	0	0	0	0	0	0	0	0	0	0	0	0	0	0	0	0
Mirtazapine	0	0	0	0	0.01	0.02	0.02	0.04	0.05	0.07	0.07	0.08	0.08	0.08	0.08	0.08	0.07	0.06	0.06	0.07	0.06	0
Multidomain Therapy	0	0	0	0	0	0	0	0.01	0.02	0.03	0.06	0.09	0.12	0.14	0.15	0.14	0.11	0.07	0.04	0.02	0.01	0
Music Therapy	0	0.06	0.1	0.16	0.18	0.14	0.11	0.08	0.05	0.03	0.02	0.02	0.01	0.01	0.01	0	0	0	0	0	0	0
Occupational Therapy	0	0	0	0.01	0.02	0.04	0.06	0.09	0.1	0.11	0.1	0.09	0.08	0.07	0.06	0.04	0.04	0.03	0.02	0.02	0.01	0
Placebo	0	0	0	0	0	0	0	0	0	0	0	0	0	0.01	0.03	0.08	0.15	0.23	0.25	0.18	0.06	0
Pet/Robot-Assisted Therapy	0	0.1	0.09	0.13	0.12	0.09	0.07	0.06	0.05	0.04	0.03	0.03	0.02	0.02	0.02	0.02	0.02	0.01	0.01	0.03	0.05	0.01
Psychosocial Therapy	0	0	0	0.01	0.02	0.03	0.05	0.07	0.09	0.09	0.08	0.08	0.07	0.07	0.06	0.05	0.04	0.04	0.04	0.04	0.05	0
Reminiscence Therapy	0	0.01	0.04	0.1	0.18	0.23	0.2	0.13	0.06	0.03	0.01	0.01	0	0	0	0	0	0	0	0	0	0
Sertraline	0	0	0	0	0	0	0.01	0.01	0.02	0.04	0.06	0.07	0.09	0.1	0.11	0.1	0.1	0.08	0.07	0.07	0.06	0
Strength Exercise	0	0.13	0.3	0.13	0.08	0.06	0.04	0.03	0.03	0.02	0.02	0.01	0.02	0.01	0.01	0.01	0.01	0.01	0.01	0.02	0.05	0.01
Tai Chi	0	0	0	0	0	0	0	0.01	0.01	0.01	0.02	0.03	0.04	0.05	0.06	0.08	0.09	0.11	0.12	0.16	0.2	0.01
Venlafaxine	0	0	0	0	0	0	0	0	0	0	0	0	0	0	0	0	0	0	0	0.01	0.03	0.93

B. Geriatric Depression Scale

Drug	Rank 1	Rank 2	Rank 3	Rank 4	Rank 5	Rank 6	Rank 7	Rank 8	Rank 9	Rank 10	Rank 11	Rank 12	Rank 13	Rank 14	Rank 15	Rank 16	Rank 17	Rank 18	Rank 19
Aerobic Exercise	0.01	0.03	0.03	0.04	0.04	0.05	0.05	0.06	0.06	0.07	0.07	0.06	0.06	0.07	0.07	0.08	0.08	0.06	0.02
Behavioral Training	0	0.02	0.04	0.07	0.09	0.11	0.12	0.12	0.11	0.09	0.07	0.04	0.04	0.02	0.02	0.01	0.01	0	0
CIT	0.02	0.04	0.05	0.05	0.05	0.06	0.06	0.06	0.07	0.06	0.06	0.05	0.05	0.05	0.06	0.06	0.06	0.05	0.03
Cognitive stimulated training	0	0.01	0.01	0.02	0.02	0.03	0.04	0.05	0.06	0.06	0.07	0.08	0.08	0.08	0.09	0.1	0.09	0.08	0.04
Electrical Current Therapy	0.11	0.12	0.09	0.07	0.06	0.06	0.05	0.05	0.05	0.04	0.04	0.03	0.03	0.03	0.03	0.04	0.03	0.03	0.02
EGb 761	0	0.01	0.01	0.02	0.02	0.02	0.03	0.04	0.04	0.04	0.05	0.06	0.06	0.07	0.08	0.1	0.12	0.14	0.1
Exercise	0.01	0.02	0.02	0.03	0.03	0.03	0.04	0.05	0.06	0.06	0.06	0.06	0.06	0.07	0.08	0.09	0.1	0.09	0.05
Hormone Therapy	0	0.01	0.01	0.02	0.02	0.03	0.03	0.04	0.04	0.05	0.06	0.07	0.07	0.08	0.09	0.1	0.11	0.12	0.07
Massage	0.06	0.1	0.1	0.09	0.08	0.08	0.07	0.07	0.06	0.05	0.05	0.03	0.03	0.03	0.03	0.02	0.02	0.01	0.01
Multidomain Therapy	0.06	0.18	0.17	0.14	0.11	0.09	0.07	0.05	0.04	0.03	0.02	0.01	0.01	0.01	0	0	0	0	0
Music Therapy	0.06	0.13	0.13	0.12	0.1	0.08	0.07	0.07	0.06	0.05	0.03	0.02	0.02	0.02	0.01	0.01	0.01	0	0
Placebo	0	0	0	0	0	0	0	0	0.02	0.05	0.11	0.21	0.21	0.19	0.14	0.07	0.03	0	0
Psychosocial Therapy	0	0.01	0.02	0.03	0.05	0.06	0.07	0.08	0.09	0.09	0.09	0.07	0.07	0.06	0.06	0.05	0.04	0.02	0.01
Reminiscence Therapy	0.01	0.04	0.1	0.14	0.15	0.14	0.13	0.1	0.07	0.05	0.03	0.01	0.01	0.01	0	0	0	0	0
Rivastigmine	0.56	0.17	0.08	0.05	0.03	0.02	0.02	0.01	0.01	0.01	0.01	0	0	0	0	0	0	0	0
Sertraline	0.01	0.01	0.02	0.03	0.03	0.04	0.04	0.05	0.06	0.06	0.07	0.07	0.07	0.07	0.08	0.1	0.1	0.09	0.02
Strength Exercise	0.03	0.04	0.04	0.04	0.04	0.04	0.04	0.04	0.05	0.05	0.05	0.05	0.05	0.05	0.06	0.07	0.08	0.1	0.09
Tai Chi	0.03	0.05	0.05	0.04	0.05	0.05	0.05	0.05	0.05	0.05	0.05	0.05	0.05	0.05	0.05	0.06	0.07	0.08	0.06
Venlafaxine	0.01	0.01	0.01	0.01	0.01	0.01	0.01	0.02	0.02	0.02	0.02	0.02	0.02	0.03	0.04	0.05	0.07	0.12	0.48

C. Hamilton Depression Rating Scale

Drug	Rank 1	Rank 2	Rank 3	Rank 4	Rank 5	Rank 6	Rank 7	Rank 8	Rank 9	Rank 10	Rank 11	Rank 12	Rank 13	Rank 14	Rank 15	Rank 16
Amitriptyline	0	0	0	0	0	0	0	0	0	0	0	0	0.01	0.01	0.03	0.94
Behavioral Training	0.01	0.11	0.17	0.19	0.18	0.13	0.09	0.05	0.03	0.02	0.01	0	0	0	0	0
Citalopram	0.03	0.17	0.14	0.14	0.12	0.1	0.08	0.06	0.05	0.03	0.02	0.01	0.01	0.01	0	0
Cognitive stimulated training	0.03	0.2	0.17	0.16	0.13	0.1	0.07	0.05	0.04	0.02	0.01	0.01	0	0	0	0
Donepezil	0	0	0	0.01	0.01	0.02	0.04	0.08	0.13	0.19	0.2	0.13	0.09	0.06	0.02	0
EGb 761	0	0.01	0.02	0.05	0.09	0.13	0.16	0.18	0.16	0.1	0.05	0.02	0.02	0.01	0.01	0
Exercise	0	0	0	0	0.01	0.02	0.03	0.06	0.09	0.14	0.18	0.17	0.15	0.12	0.04	0
Fluoxetine	0	0	0	0	0.01	0.01	0.01	0.02	0.02	0.03	0.04	0.05	0.07	0.14	0.61	0.01
Hormone Therapy	0	0.02	0.02	0.04	0.05	0.07	0.08	0.09	0.11	0.12	0.1	0.07	0.08	0.1	0.05	0.01
Imipramine	0	0	0	0	0	0	0.01	0.02	0.03	0.05	0.08	0.1	0.16	0.34	0.18	0.02
Multidomain Therapy	0.01	0.06	0.08	0.1	0.12	0.12	0.13	0.11	0.09	0.07	0.04	0.03	0.02	0.01	0.01	0
Music Therapy	0.85	0.07	0.03	0.02	0.01	0.01	0	0	0	0	0	0	0	0	0	0
Placebo	0	0	0	0	0	0	0	0	0.01	0.05	0.15	0.31	0.33	0.13	0.02	0
Rivastigmine	0	0.02	0.04	0.06	0.09	0.11	0.14	0.15	0.13	0.1	0.06	0.04	0.03	0.02	0.01	0
Sertraline	0.05	0.28	0.2	0.14	0.11	0.07	0.05	0.04	0.02	0.01	0.01	0.01	0	0	0	0

D. Mini-Mental State Examination

Drug	Rank 1	Rank 2	Rank 3	Rank 4	Rank 5	Rank 6	Rank 7	Rank 8	Rank 9	Rank 10	Rank 11	Rank 12	Rank 13	Rank 14	Rank 15	Rank 16	Rank 17	Rank 18	Rank 19	Rank 20	Rank 21	Rank 22
Amitriptyline	0.01	0.05	0.08	0.07	0.06	0.06	0.06	0.06	0.06	0.06	0.05	0.04	0.04	0.04	0.03	0.03	0.04	0.04	0.04	0.04	0.03	0.02
Aripiprazole	0	0	0	0	0	0	0	0.01	0.01	0.02	0.03	0.05	0.06	0.07	0.1	0.12	0.13	0.14	0.12	0.08	0.04	0.01
Behavioral Training	0	0	0	0	0.01	0.01	0.02	0.02	0.04	0.05	0.06	0.07	0.08	0.08	0.08	0.09	0.1	0.1	0.08	0.06	0.03	0.01
Citalopram	0.02	0.06	0.1	0.1	0.08	0.08	0.08	0.07	0.06	0.06	0.05	0.04	0.03	0.03	0.03	0.02	0.02	0.02	0.02	0.01	0.01	0
Cognitive stimulated training	0	0.02	0.07	0.13	0.17	0.17	0.15	0.12	0.08	0.04	0.03	0.01	0.01	0	0	0	0	0	0	0	0	0
Electrical Current Therapy	0.6	0.22	0.08	0.03	0.02	0.01	0.01	0.01	0	0	0	0	0	0	0	0	0	0	0	0	0	0
EGb 761	0	0	0.01	0.02	0.03	0.04	0.06	0.07	0.09	0.1	0.1	0.09	0.08	0.07	0.06	0.05	0.05	0.04	0.03	0.02	0.01	0
Exercise	0	0.01	0.02	0.04	0.05	0.06	0.08	0.09	0.09	0.1	0.09	0.08	0.06	0.05	0.04	0.04	0.04	0.03	0.02	0.01	0.01	0
Fluoxetine	0	0	0	0.01	0.01	0.01	0.01	0.01	0.01	0.02	0.02	0.03	0.03	0.03	0.03	0.04	0.05	0.07	0.09	0.13	0.18	0.23
Hormone Therapy	0	0.01	0.01	0.02	0.03	0.03	0.03	0.04	0.05	0.05	0.06	0.06	0.06	0.05	0.05	0.06	0.07	0.07	0.08	0.07	0.06	0.03
Imipramine	0	0.01	0.01	0.02	0.02	0.02	0.02	0.03	0.03	0.03	0.03	0.04	0.04	0.04	0.04	0.05	0.06	0.07	0.09	0.11	0.13	0.13
Light therapy	0	0.01	0.02	0.03	0.03	0.03	0.04	0.04	0.05	0.05	0.04	0.05	0.04	0.04	0.04	0.05	0.06	0.07	0.07	0.08	0.09	0.06
Massage	0.03	0.08	0.1	0.09	0.07	0.06	0.06	0.06	0.06	0.05	0.05	0.04	0.04	0.03	0.03	0.03	0.03	0.03	0.03	0.03	0.02	0.01
Multidomain Therapy	0	0.01	0.02	0.03	0.04	0.05	0.05	0.06	0.07	0.07	0.07	0.07	0.06	0.05	0.05	0.06	0.06	0.06	0.05	0.04	0.03	0.01
Music Therapy	0.22	0.31	0.16	0.07	0.05	0.03	0.03	0.02	0.02	0.02	0.01	0.01	0.01	0.01	0.01	0.01	0	0	0	0	0	0
Occupational Therapy	0.11	0.14	0.12	0.07	0.05	0.04	0.04	0.04	0.03	0.03	0.03	0.03	0.02	0.02	0.02	0.02	0.02	0.03	0.03	0.03	0.03	0.04
Placebo	0	0	0	0	0	0	0	0	0	0.01	0.04	0.1	0.17	0.22	0.21	0.14	0.07	0.03	0.01	0	0	0
Psychosocial Therapy	0	0	0	0.01	0.02	0.02	0.03	0.04	0.06	0.07	0.08	0.08	0.08	0.08	0.08	0.08	0.08	0.07	0.06	0.04	0.02	0.01
Reminiscence Therapy	0	0.02	0.07	0.11	0.13	0.13	0.12	0.11	0.09	0.07	0.05	0.03	0.02	0.01	0.01	0.01	0	0	0	0	0	0
Rivastigmine	0.01	0.05	0.11	0.14	0.13	0.12	0.1	0.09	0.07	0.06	0.04	0.03	0.02	0.01	0.01	0.01	0	0	0	0	0	0
Sertraline	0	0	0	0	0	0	0.01	0.01	0.01	0.01	0.01	0.02	0.02	0.02	0.02	0.03	0.04	0.06	0.08	0.12	0.2	0.34
Tai Chi	0	0	0.01	0.01	0.01	0.02	0.02	0.02	0.03	0.04	0.04	0.04	0.04	0.04	0.05	0.06	0.07	0.08	0.1	0.11	0.11	0.08

E. Neuropsychiatric Inventory

Drug	Rank 1	Rank 2	Rank 3	Rank 4	Rank 5	Rank 6	Rank 7	Rank 8	Rank 9	Rank 10	Rank 11	Rank 12	Rank 13	Rank 14	Rank 15	Rank 16	Rank 17	Rank 18
Aerobic Exercise	0.02	0.04	0.05	0.09	0.14	0.16	0.17	0.15	0.07	0.04	0.03	0.02	0.01	0.01	0	0	0	0
Aripiprazole	0	0	0	0	0	0.01	0.05	0.12	0.23	0.24	0.19	0.08	0.04	0.02	0.01	0	0	0
Analgesic Treatment	0.33	0.22	0.14	0.13	0.07	0.06	0.03	0.01	0.01	0	0	0	0	0	0	0	0	0
Behavioral Training	0.05	0.09	0.17	0.3	0.2	0.1	0.06	0.02	0.01	0	0	0	0	0	0	0	0	0
Citalopram	0	0.01	0.02	0.07	0.24	0.31	0.21	0.09	0.02	0.01	0	0	0	0	0	0	0	0
Cognitive stimulated training	0	0	0	0	0	0.01	0.01	0.04	0.06	0.1	0.12	0.14	0.14	0.1	0.09	0.1	0.07	0.02
Discontinuation of antipsychotics	0	0	0	0	0	0	0.01	0.01	0.02	0.02	0.04	0.04	0.05	0.04	0.06	0.12	0.24	0.35
Donepezil	0.48	0.23	0.12	0.08	0.05	0.02	0.01	0.01	0	0	0	0	0	0	0	0	0	0
EGb 761	0.07	0.33	0.39	0.16	0.04	0.01	0	0	0	0	0	0	0	0	0	0	0	0
Exercise	0	0	0	0	0	0.01	0.02	0.07	0.16	0.21	0.17	0.14	0.09	0.05	0.03	0.02	0.01	0
Light therapy	0	0	0	0.01	0.01	0.01	0.02	0.03	0.04	0.04	0.04	0.04	0.04	0.05	0.06	0.08	0.16	0.38
Multidomain Therapy	0	0	0	0	0	0	0.01	0.02	0.04	0.09	0.17	0.22	0.19	0.12	0.07	0.03	0.01	0
Music Therapy	0.01	0.02	0.02	0.02	0.03	0.04	0.09	0.08	0.09	0.06	0.05	0.05	0.04	0.03	0.04	0.07	0.11	0.11
Placebo	0	0	0	0	0	0	0	0	0	0	0.01	0.05	0.18	0.3	0.27	0.14	0.05	0
Psychosocial Therapy	0	0.01	0.02	0.05	0.08	0.12	0.16	0.21	0.13	0.08	0.05	0.04	0.02	0.01	0.01	0.01	0	0
Reminiscence Therapy	0	0	0	0	0	0	0	0.01	0.02	0.04	0.06	0.09	0.09	0.11	0.12	0.2	0.17	0.07
Sertraline	0.03	0.05	0.07	0.09	0.13	0.13	0.13	0.12	0.08	0.05	0.04	0.02	0.02	0.01	0.01	0.01	0.01	0
Tai Chi	0	0	0	0	0	0	0	0.01	0.01	0.02	0.03	0.05	0.08	0.14	0.22	0.21	0.16	0.06

.Cohen-Mansfeld Agitation Inventory

Drug	Rank 1	Rank 2	Rank 3	Rank 4	Rank 5	Rank 6	Rank 7	Rank 8	Rank 9
Aripiprazole	0.02	0.11	0.19	0.25	0.2	0.12	0.07	0.02	0.01
Analgesic Treatment	0.02	0.06	0.09	0.11	0.14	0.16	0.21	0.13	0.07
Citalopram	0.02	0.05	0.09	0.14	0.19	0.25	0.16	0.07	0.04
Fluoxetine	0.01	0.02	0.03	0.03	0.04	0.06	0.07	0.12	0.63
Light therapy	0.06	0.18	0.18	0.16	0.15	0.11	0.08	0.05	0.03
Massage	0.06	0.21	0.24	0.17	0.13	0.09	0.06	0.03	0.01
Placebo	0	0	0	0.01	0.02	0.08	0.23	0.49	0.17
Pet/Robot-Assisted Therapy	0.62	0.19	0.08	0.04	0.03	0.02	0.01	0.01	0
Sertraline	0.18	0.19	0.11	0.09	0.1	0.12	0.11	0.07	0.04
