# Supplementary material for: Comparative efficacy and acceptability of treatments for depressive symptoms in cognitive impairment: A systematic review and Bayesian network meta-analysis
Source: Front Aging Neurosci. 2022 Dec 12;14:1037414. doi: 10.3389/fnagi.2022.1037414 (PMC9790988; doi:10.3389/fnagi.2022.1037414)
Supplement: Supplementary material 1 — PRISMA NMA checklist. [file Data_Sheet_1.zip › Supplementary Material Presentation/Supplementary_12.pdf]

A. Total adverse events

Citalopram	1.12 (0.07, 16.60)	0.45 (0.09, 2.07)	0.79 (0.08, 6.41)	11.96 (0.49, 769.16)
0.89 (0.06, 13.75)	Hormone Therapy	0.39 (0.04, 3.72)	0.70 (0.04, 9.90)	10.92 (0.30, 960.36)
2.24 (0.48, 11.16)	2.54 (0.27, 23.71)	Placebo	1.78 (0.35, 7.61)	5.98 (1.72, 26.34)
1.26 (0.16, 13.13)	1.44 (0.10, 23.83)	0.56 (0.13, 2.88)	Sertraline	15.72 (0.70, 923.60)
0.08 (0.00, 2.06)	0.09 (0.00, 3.35)	0.17 (0.04, 0.58)	0.06 (0.00, 1.44)	Venlafaxine

B. Severe adverse events

Aripiprazole	0.45 (0.12, 1.50)	2.76 (0.14, 251.45)	0.78 (0.33, 1.65)	1.08 (0.30, 3.23)	4.65 (0.48, 39.95)
2.20 (0.67, 8.49)	EGb 761	5.97 (0.30, 536.45)	1.70 (0.66, 4.88)	2.34 (0.62, 8.70)	9.97 (1.04, 94.07)
0.36 (0.00, 7.34)	0.17 (0.00, 3.38)	Fluoxetine	0.28 (0.00, 5.27)	0.38 (0.00, 8.12)	1.63 (0.01, 56.54)
1.29 (0.61, 3.01)	0.59 (0.21, 1.53)	3.55 (0.19, 287.01)	Placebo	1.38 (0.55, 3.13)	5.90 (0.73, 42.97)
0.93 (0.31, 3.31)	0.43 (0.12, 1.61)	2.62 (0.12, 275.17)	0.72 (0.32, 1.81)	Sertraline	4.29 (0.66, 28.05)
0.22 (0.03, 2.09)	0.10 (0.01, 0.96)	0.61 (0.02, 92.43)	0.17 (0.02, 1.37)	0.23 (0.04, 1.51)	Venlafaxine

C.Diarrhea

Aripiprazole	0.76 (0.01, 42.08)	0.93 (0.12, 7.11)	0.79 (0.13, 4.51)	0.99 (0.12, 6.65)
1.31 (0.02, 86.02)	Citalopram	1.19 (0.03, 64.08)	1.01 (0.03, 46.42)	1.23 (0.03, 59.95)
1.08 (0.14, 8.58)	0.84 (0.02, 37.70)	EGb 761	0.85 (0.30, 2.35)	1.07 (0.22, 3.97)
1.26 (0.22, 7.44)	0.99 (0.02, 39.61)	1.17 (0.43, 3.33)	Placebo	1.25 (0.42, 3.11)
1.01 (0.15, 8.62)	0.81 (0.02, 38.54)	0.94 (0.25, 4.48)	0.80 (0.32, 2.41)	Sertraline

D. Headache

Aripiprazole	0.22 (0.03, 1.53)	0.96 (0.07, 12.01)	0.53 (0.08, 3.04)	0.46 (0.05, 3.47)	0.05 (0.00, 1.32)
4.48 (0.66, 36.70)	EGb 761	4.43 (0.58, 32.00)	2.36 (1.05, 5.49)	2.08 (0.55, 7.83)	0.24 (0.01, 3.90)
1.05 (0.08, 13.95)	0.23 (0.03, 1.71)	Hormone Therapy	0.53 (0.09, 3.36)	0.46 (0.06, 3.89)	0.05 (0.00, 1.42)
1.89 (0.33, 13.26)	0.42 (0.18, 0.96)	1.88 (0.30, 11.27)	Placebo	0.88 (0.31, 2.46)	0.10 (0.00, 1.49)
2.16 (0.29, 19.50)	0.48 (0.13, 1.82)	2.15 (0.26, 16.99)	1.14 (0.41, 3.20)	Sertraline	0.12 (0.00, 1.43)
18.55 (0.76, 1128.94)	4.10 (0.26, 149.70)	18.80 (0.70, 927.56)	9.74 (0.67, 329.46)	8.39 (0.70, 257.80)	Venlafaxine

E. Nausea

Citalopram	1.00 (0.08, 11.43)	3.82 (0.12, 245.63)	1.55 (0.22, 11.11)	1.11 (0.09, 13.90)	2.50 (0.19, 28.81)	6.90 (0.38, 131.62)
1.00 (0.09, 13.09)	EGb 761	3.90 (0.15, 218.38)	1.64 (0.36, 7.75)	1.15 (0.13, 10.59)	2.78 (0.29, 22.04)	7.12 (0.51, 100.01)
0.26 (0.00, 8.42)	0.26 (0.00, 6.87)	Hormone Therapy	0.42 (0.01, 7.08)	0.31 (0.01, 8.22)	0.68 (0.01, 17.44)	1.61 (0.03, 64.86)
0.64 (0.09, 4.57)	0.61 (0.13, 2.79)	2.36 (0.14, 95.87)	Placebo	0.71 (0.15, 3.33)	1.68 (0.33, 7.10)	4.31 (0.51, 39.08)
0.90 (0.07, 10.55)	0.87 (0.09, 7.44)	3.21 (0.12, 181.10)	1.41 (0.30, 6.67)	Rivastigmine	2.40 (0.24, 18.39)	5.93 (0.41, 87.20)
0.40 (0.03, 5.19)	0.36 (0.05, 3.39)	1.46 (0.06, 87.51)	0.60 (0.14, 3.03)	0.42 (0.05, 4.15)	Sertraline	2.61 (0.42, 19.69)
0.14 (0.01, 2.64)	0.14 (0.01, 1.97)	0.62 (0.02, 38.38)	0.23 (0.03, 1.97)	0.17 (0.01, 2.42)	0.38 (0.05, 2.39)	Venlafaxine
