# Supplementary material for: Comparative efficacy and acceptability of treatments for depressive symptoms in cognitive impairment: A systematic review and Bayesian network meta-analysis
Source: Front Aging Neurosci. 2022 Dec 12;14:1037414. doi: 10.3389/fnagi.2022.1037414 (PMC9790988; doi:10.3389/fnagi.2022.1037414)
Supplement: Supplementary material 1 — PRISMA NMA checklist. [file Data_Sheet_1.zip › Supplementary Material Presentation/Supplementary_2.pdf]

Searching strategies

1. *Therapeutics/OR *Drug Therapy/OR * Antidepressants/OR * Selective serotonin reuptake inhibitors /OR * Selective serotonin receptor agonists/OR * Tricyclic antidepressants/ OR * Serotonin antagonists/ OR * Cholinesterase inhibitors /OR *Antipsychotics/OR * Analgesics/ OR * Hormone / OR *Adrenergic beta-Antagonists/ OR *Calcium channel blockers/OR *Chinese medicine/OR * Cognitive therapy /OR * Noninvasive brain stimulation /OR * Psychological treatments / OR * Physiotherapy approach /OR * Exercise /OR * Multidomain interventions /
2. (Analgesic* OR Opioids: Morphine OR Tramadol OR Meperidine OR Acetaminophen OR “Lysine acetylsalicylic acid”).ab,ti
3. (Antipsychotics* OR Aripiprazole OR chlorpromazine OR clozapine OR haloperidol OR levomepromazine OR perphenazine OR prochlorperazine OR Olanzapine OR quetiapine OR risperidone) .ab,ti.
4. (Cholinesterase inhibitors* OR “ChEIs” OR Donepezil OR galantamine OR rivastigmine).ab,ti.
5. (N-Methyl-d-aspartate receptor antagonist * OR “NMDA” OR Memantine).ab,ti.
6. (Hormone* OR Progestin OR estradiol OR norethisterone OR Estrogen).ab,ti.
7. (Ergot alkaloid* OR Ergotamine OR Dihydroergotamine).ab,ti.
8. (NSAIDN* OR “non-steroidal anti-inflammatory drug” OR Aspirin OR Ibuprofen OR Ketorolac OR Diclofenac OR Naproxen OR “Lysine clonixinate” OR Indomethacin OR Piroxicam OR Salicylate OR Tolmetin).ab,ti.
9. (SSRI* OR “Selective serotonin reuptake inhibitor” OR Citalopram OR Dapoxetine OR Escitalopram OR Fluoxetine OR Fluvoxamine OR Indalpine OR Paroxetine OR Sertraline OR Vilazodone OR Zimelidine OR Venlafaxine OR Desvenlafaxine OR Duloxetine OR Milnacipran OR Levomilnacipran OR Sibutramine OR Bicifadine).ab,ti.
10. (SSRA* OR “Selective serotonin receptor agonist” OR Triptans OR “Intranasal sumatriptan” OR Almotriptan OR Eletriptan OR Frovatriptan OR Naratriptan OR Rizatriptan OR Sumatriptan OR Zolmitriptan).ab,ti.
11. (“Serotonin antagonist” OR Pizotifen OR Sandomigran).ab,ti.
12. (Tricyclic antidepressant* OR Amitriptyline OR Amoxapine OR Clomipramine OR Desipramine OR Dibenzepin OR Dothiepin OR Doxepin OR Imipramine OR Lofepramine OR Nortriptyline OR Opipramol OR Protriptyline OR Trimipramine).ab,ti.
13. (Chinese medicine OR Lithium OR Methylphenidate OR Melatonin OR “EGb761” OR ginkgo); OR Progestin).ab,ti.
14. (psychotherap* OR therap* OR (cognitive adj behavio* adj therapy) OR “cognitive therapy” OR behavio* adj therapy OR “problem solving therapy” OR “problem-solving therapy” OR “interpersonal
therapy” OR “inter-personal therapy” OR (family adj (therapy OR intervention)) OR psychodynamic OR psychoanalytic OR bibliotherapy OR mindful* OR (group adj (therapy OR intervention)) OR emotion-focused OR “emotion focused” OR reminiscen* OR “life review” OR life-review).ab,ti.

15. (Physio* OR Exercise OR (Manual*) OR (Soft-tissue techniques) OR (Strength and endurance training) OR Yoga OR (Tai Chi) OR (Spinal Manipulation) OR Masage).ab,ti.
16. ((*brain stimulation) OR (Transcranial magnetic stimulation) OR (Transcranial direct current stimulation) OR (*Oxygen therapy)).ab,ti.
17. (Multidomain interventions* OR therap* OR (multi* adj therapy) OR (Multi-faceted) OR (Collaborative Care*) OR (Multimodal Rehabilitative*) OR (comprehensive home-based*)).ab,ti.
18. (*Occupational therap*) OR (Music therap*) OR (Supplements, botanicals and diet alteration) OR (Mind-body therap*) OR (Robot-Assisted therap*) OR (Animal-Assisted therap*) OR (Ultrasound guided nerve pulsed radiofrequency) OR (Acupuncture) .ab,ti.
19. #1 OR #2 OR #3 OR #4 OR #5 OR #6 #7 OR #8 OR #9 OR #10 OR #11 OR #12 #13 OR #14 OR #15 OR #16 OR #17 OR #18
20. (*Cognirtve Disorders OR *Cognitive deficits OR OR *Cognitive impairment OR *dementia) AND (*depression OR (depressive symptoms)).ab,ti.
21. *Randomised Controlled Trial/OR (Randomised Controlled Trial).pt OR *Random Allocation/.
22. (Randomised OR randomised OR (random* adj (assigned OR allocated OR assignment OR allocation))).ab,ti.
23. #19 OR #20.
24. #21 AND #22 AND #23
