# Supplementary material for: Comparative efficacy and acceptability of treatments for depressive symptoms in cognitive impairment: A systematic review and Bayesian network meta-analysis
Source: Front Aging Neurosci. 2022 Dec 12;14:1037414. doi: 10.3389/fnagi.2022.1037414 (PMC9790988; doi:10.3389/fnagi.2022.1037414)
Supplement: Supplementary material 1 — PRISMA NMA checklist. [file Data_Sheet_1.zip › Supplementary Material Presentation/Supplementary_3.pdf]

Treatment	Study Number	Study	Diagnosis	Patients Number	Intervention	Completed (%)	Gender (Female%)	Age, Mean(SD)	Duration	Baseline			
										MMSE	CSDD	GDS	NPI
Analgesic Treatment	1	Husebo, Ballard et al. 2013[1]	Dementia	175	Pain treatment	89.10%	74.90%	84.9 (7.0)	8 weeks	7.5 (6.4)	-	-	34.8 (21.9)
				177	Placebo	80.20%	74.00%	86.5 (6.7)		8.4 (6.7)	-	-	31.4 (21.4)
	2	Husebo, Ballard et al. 2011[2]	Dementia	175	Pain treatment	81.10%	75.00%	85.0	12 weeks	7 (0-20)	-	-	32 (1-101)
				177	Placebo	85.90%	74.00%	87.0		8 (0-20)	-	-	29 (0-97)
Aripiprazole	3	Streim, Porsteinsson et al. 2008[3]	Dementia	131	Aripiprazole 2 mg	99.20%	56.40%	83.0	10 weeks	17.75 (9.725)	8.72 (4.71)	-	-
				125	Placebo	96.80%	62.40%	83.0		13.26 (4.44)	9.18 (4.4)	-	-
	4	Mintzer, Tune et al. 2007[4]	Dementia	118	Aripiprazole 2 mg	65.00%	68.64%	83.0	10 weeks	13.0 (8.0)	-	-	-
				122	Aripiprazole 5mg	60.00%	62.30%	82.4		12.4 (8.2)	-	-	-
				126	Aripiprazole 10mg	55.00%	60.30%	82.3		12.2 (8.0)	-	-	-
				121	Placebo	54.00%	67.77%	82.2		11.7 (8.2)	-	-	-
	5	De Deyn, Jeste et al. 2005[5]	Alzheimer's disease	106	Aripiprazole 10 mg	83.00%	72.00%	55-95	10 weeks	14.35	-	-	39.82

				102	Placebo	82.00%				14.13	-	-	40.08
Behavioral training	6	Travers 2016[6]	Dementia	10	Behavioral training	100.00%	80.00%	87.2 (7.7)	10 weeks	19.5 (3.87)	-	4 (4-7)	-
				8	Control care	100.00%	100.00%	85.5 (10.9)		16.13 (3.7)	-	5 (4.0-7.25)	-
	7	Linda, Logsdon et al. 1997[7]	Alzheimer's disease	23	Behavioral training -pleasant events	100.00%	70.00%	72.8 (8.2)	9 weeks	15.8 (7.8)	14.8 (4.2)	-	-
				19	Behavioral training -problem solving	100.00%	26.00%	78.5 (7.9)		15.7 (7.4)	15.1 (3.5)	-	-
				10	Typical control care	100.00%	60.00%	79.5 (6.9)		16.8 (5.4)	13.9 (4.6)	-	-
				20	Wait list control care	100.00%	75.00%	76.8 (8.2)		17.9 (7.9)	14.0 (4.2)	-	-
	8	Lichtenberg, Kemp-Havican et al[8]	Dementia	9	Behavioral training	100.00%	90.00%	84.8 (4.9)	12 weeks	-	13.1 (7.0)	3.6 (3.2)	-
				11	Control care	100.00%	90.00%	85.0 (5.1)		-	11.3 (5.8)	3.6 (3.2)	-
	9	Lam, Lui et al. 2010[9]	Dementia	37	Behavioral training	83.70%	67.60%	83.1(6.9)	8 weeks	13.6(4.0)	4.43 (3.1)	-	-
				37	Control care	70.30%	81.10%	83.8 (7.0)		13.9(4.8)	3.43 (4.24)	-	-
	10	Verkaik, Francke et al. 2011[10]	Dementia	62	Behavioral training	87.10%	83.90%	83.4 (7.2)	11 weeks	-	-	-	-

				35	Control care	80.00%	80.00%	84.1 (7.1)		-	-	-	-
	11	Tse, Lau et al. 2018[11]	Dementia	29	Behavioral training	100.00%	71.00%	83.3 (7.5)	8 weeks.	21.58 (2.75)	-	5.67 (2.05)	19.00 (4.32)
				24	Control care	100.00%	76.90%	87.6 (8.0)		19.49 (3.93)	-	8.40 (3.22)	17.38 (6.32)
	12	Todri, Todri et al. 2019[12]	Alzheimer's disease	45	Behavioral training	100.00%	62.20%	81.8 (4.8)	6 months	-	-	-	-
				45	Control care	100.00%	48.90%	80.4 (5.59)		-	-	-	-
	13	Teri, Gibbons et al. 2003[13]	Alzheimer’s disease	76	Behavioral training	59.00%	37.00%	78 (6)	3 months	17.6 (6.8)	5.8 (4.5)	-	-
				77	Control care	57.00%	45.00%	78 (8)		15.9 (7.4)	5.7 (3.9)	-	-
**Cognitive stimulation therapy**	14	Woods, Thorgrimsen et al. 2006[14]	Dementia	101	Cognitive stimulation therapy	100.00%	78.60%	85.3 (7.0)	8 weeks	14.4 (3.8)	5.5 (4.9)	-	-
				100	Control care	100.00%						-	-
	15	Yi-Xuan, Niu et al. 2010[15]	Alzheimer’s disease	16	Cognitive stimulation therapy	100.00%	12.50%	80.56 (4.23)	10 weeks	16.93 (3.02)	-	-	14.06 (5.69)
				16	Control care	100.00%	31.30%	79.13 (4.38)		17.31 (3.24)	-	-	13.69 (4.60)
	16	Buschert, Friese et al. 2011[16]	Cognitive impairment	12	Cognitive stimulation therapy	83.33%	50.00%	71.8 (8.6)	6 months	28.1 (1.5)	-	-	-

				12	Control care	100.00%	50.00%	70.7 (5.7)		26.8 (1.5)	-	-	-
			Alzheimer's disease	8	Cognitive stimulation therapy	100.00%	50.00%	77.3 (7.6)		24.5 (1.6)	-	-	-
				7	Control care	100.00%	57.10%	74.2 (9.0)		25.3 (1.5)	-	-	-
	17	Maci, Pira et al. 2012[17]	Alzheimer's disease	7	Cognitive stimulation therapy	100.00%	75.00%	75 (12.3)	3 months	17.5 (2.7)	9.0 (5.5)	-	-
				7	Control care	100.00%	75.00%	70.3 (5.8)	3 months	18.2 (2.9)	9.5 (2.9)	-	-
	18	Bergamaschi, Arcara et al. 2013[18]	Alzheimer's disease	16	Cognitive stimulation therapy	100.00%	-	78.19 (5.50)	1 year	20.25 (2.95)	-	-	-
				16	Control care	100.00%		77.72 (5.06)		21.94 (2.01)	-	-	-
	19	Jha, Jan et al. 2013[19]	Dementia	24	Cognitive stimulation therapy	100.00%	70.83%	78.47 (8)	6 months	21 (6)	6.4 (2.6)	-	-
				24	Control care	100.00%	62.50%	79 (7.6)		23 (5)	6.9 (3)	-	-
	20	Stanley, Calleo et al. 2013[20]	Dementia	16	Cognitive stimulation therapy	68.75%	62.50%	77.6 (10.54)	3 months	-	-	9.4 (7.19)	-

				16	Control care	93.75%	56.30%	79.6 (8.97)		-	-	10.7 (6.457)	-
	21	Orgeta, Leung et al. 2015[21]	Dementia	180	Cognitive stimulation therapy	75.00%	46.00%	78.4 (7.3)	26weeks	21.12 (4.48)	-	3.14 (2.64)	11.21 (13.96)
				176	Control care	79.00%	50.00%	78 (7.7)		21.33 (4.11)	-	3.16 (3.15)	10.99 (11.98)
	22	Amieva, Robert et al. 2015[22]	Alzheimer's disease	170	Cognitive stimulation therapy	88.80%	58.20%	78.5 (7.2)	3 months	21.5 (3.2)	-	-	16.3 (12.6)
				172	Reminiscence therapy	87.20%	62.80%	78.8 (6.9)		21.1 (3.1)	-	-	15.8 (12.9)
				157	Cognitive rehabilitation therapy	91.70%	58.60%	78.9 (6.2)		21.6 (3.0)	-	-	16.0 (16.1)
				154	Control care	91.60%	58.40%	78.7 (6.5)		21.6 (3.3)	-	-	17.5 (15.2)
	23	Capotosto, Belacchi et al. 2017[23]	Dementia	20	Cognitive stimulation therapy	100.00%	75.00%	88.25 (5.15)	7 weeks	18.30 (3.14)	2.90 (2.15)	-	22.1 (8.17)
				19	Control care	100.00%	63.20%	86.5 2(5.55)		18.20 (3.63)	2.63 (2.52)	-	19.32 (7.23)
	24	Churcher Clarke, Chan et al. 2017[24]	Dementia	20	Cognitive Mindfulness therapy	100.00%	60.00%	81.30 (9.29)	1week	-	6.80 (4.35)	-	-

				11	Control care	72.70%	27.30%	79.36 (9.91)		-	8.09 (6.06)	-	-
**Current Stimulation**	25	Khedr et al., 2019[25]	Alzheimer's disease	23	Transcranial Direct Current Stimulation	100.00%	43.50%	64.22 (3.64)	2 weeks	-	17.47 (4.75)	-	-
				23	Control care	91.30%	38.10%	65.23 (4.52)		-	16.90 (3.78)	-	-
	26	Luijpen, Swaab et al. 2004[26]	Cognitive Impairment	17	Transcutaneous Electrical Nerve Stimulation	100.00%	88.20%	88.06	4 weeks	20.24 (3.70)	-	7.94 (3.25)	-
				17	Control care	100.00%	64.70%	87.35		23.41 (2.35)	-	10.35 (5.68)	-
**Citalopram**	27	Nyth and Gottfries 1990[27]	Dementia	44	Citalopram 30mg/day	59.10%	77.50%	-	6 weeks	-	-	-	-
				45	Placebo	64.40%					-	-	-
	28	Nyth, Gottfries et al. 1992[28]	Dementia	88	Citalopram 10mg/day	68.10%	67.00%	76.14	6 weeks	-	-	-	-
				45	Placebo	71.10%	73.30%	77.7		-	-	-	-
	29	An, Choi et al. 2017[29]	Alzheimer's disease	41	Citalopram 15mg/day	65.90%	88.90%	74.33 (7.47)	12 weeks	19.04 (4.26)	11.11 (7.13)	9.56 (3.04)	-
				43	Placebo	76.70%	72.70%	75.85 (6.73)		18.70 (4.92)	11.67 (7.82)	9.2 1(2.99)	-
	30	Porsteinsson, Drye et al. 2014[30]	Alzheimer's disease	94	Citalopram 30mg/day	91.50%	47.00%	78 (9)	9 weeks	17.0 (6.2)	-	-	37.3 (17.5)

				92	Placebo	90.20%	45.00%	79 (8)		14.4 (6.9)	-	-	37.3 (17.7)
Donepezil	31	Devanand, Pelton et al. 2018[31]	Cognitive impairment	31	Donepezil	100.00%	53.33%	67.10 (7.66)	16 weeks	-	-	-	-
				30	Placebo	100.00%	48.39%	72.39 (8.90)		-	-	-	-
	32	Yancheva, Ihl et al. 2009[32]	Alzheimer's disease	31	EGB 761	96.80%	54.80%	69 (8)	22 weeks	-	-	-	18.9 (7.1)
				32	Donepezil	90.60%	84.40%	66 (8)		-	-	-	21.5 (9.7)
	33	Reynolds, Butters et al. 2011[33]	Cognitive impairment	67	Donepezil	62.90%	73.13%	73.1 (6.5)	16 weeks	28.5 (1.4)	-	-	-
				63	Placebo	77.80%	81.00%	73.9 (5.8)		28.4 (1.4)	-	-	-
EGB 761	34	Van Dongen, Van Rossum et al. 2000[34]	Dementia	84	EGB 761 160mg	100.00%	85.00%	82.8 (4.9)	24 weeks	18.3 (4.8)	-	2.5 (2.2)	-
				82	EGB 761 240mg	100.00%	85.00%	83.3 (5.3)		18.4 (4.2)	-	2.5 (2.4)	-
				48	Placebo	100.00%	81.00%	82.6 (5.7)		18.5 (4.5)	-	3.0 (2.0)	-
	35	Napryeyenko, Sonnik et al. 2009[35]	Alzheimer's disease	104	EGB 761 240mg	100.00%	67.00%	66 (8)	22 weeks	-	-	-	19.6 (8.4)
				110	Placebo	100.00%	71.00%	64 (8)		-	-	-	20.1 (8.6)
			Vascuar Disease	94	EGB 761 240mg	100.00%	78.00%	63 (8)		-	-	-	23.2 (10.4)

				87	Placebo	100.00%	74.00%	63 (9)		-	-	-	23.6 (11.2)
	36	Kanowski and Hoerr 2003[36]	Alzheimer's disease	106	EGB 761 240mg	74.50%	68.00%	72 (10)	24 weeks	21.6 (2.6)	-	-	-
				99	Placebo	79.80%	71.00%	72 (10)		21.5 (2.4)	-	-	-
	37	Napryeyenko and Borzenko 2007[37]	Dementia	198	EGB 761 240mg	72.00%	72.00%	65 (8)	22 weeks	-	-	-	21.3 (9.5)
				197	Placebo	72.00%	72.00%	63 (8)		-	-	-	21.6 (9.9)
**Exercise**	38	Liu, Lee et al. 2020[38]	Dementia	35	Strength-exercise	85.70%	20.00%	86.77 (6.99)	4 weeks	22.7 (4.28)	-	0.4 (0.68)	
				34	Aerobic-exercise	91.20%	16.13%	84.68 (6.74)		23.87 (4.65)	-	0.4 (0.68)	-
	39	Rolland, Pillard et al. 2007[39]	Alzheimer's disease	67	Combined Exercise	89.60%	71.70%	82.8 (7.8)	6 months	9.7 (6.8)	-	-	10.7 (6.9)
				67	Control	85.10%	79.10%	83.1 (7.0)		7.9 (6.4)	-	-	11.4(7.7)
	40	Bostrom, Conradsson et al. 2016[40]	Dementia	93	Combined Exercise	93.00%	80.60%	84.4 (6.2)	4 months	15.4 (3.4)	-	-	15.2 (15.8)
				93	Control	90.00%	81.70%	85.9 (7.8)		14.4 (3.5)	-	-	14.4 (12.6)
	41	Cancela, Ayan et al. 2016[41]	Dementia	73	Aerobic-exercise	70.00%	43.80%	80.63 (8.32)	15 months	-	6.31 (5.07)	-	9.70 (9.45)
				116	Control	54.30%	81.00%	82.90 (7.42)		-	6.71 (6.48)	-	11.32 (5.35)

	42	Hoffmann, Sobol et al. 2016[42]	Alzheimer's disease	107	Combined Exercise	95.30%	47.70%	69.8 (7.4)	16 weeks	23.8 (3.4)	-	-	10 (10.8)
				93	Control	94.62%	38.70%	71.3 (7.3)		24.1 (3.8)	-	-	9.4 (9.7)
	43	Chen, Kuo et al. 2017[43]	Dementia	75	Combined Exercise	86.70%	58.50%	80.7 (8.0)	15 months	12.1 (3.9)	3.2 (2.4)	-	-
				75	Control	82.70%	54.80%	81.6 (6.7)		11.0 (4.8)	2.6 (2.9)	-	-
	44	Morris, Vidoni et al. 2017[44]	Alzheimer's disease	39	Aerobic-exercise	94.90%	43.20%	74.4 (6.7)	26 weeks	25.8 (3.3)	8.6 (5.1)	-	-
				37	Strength-exercise	91.90%	57.70%	71.4 (8.4)		25.0 (3.2)	7.4 (3.8)	-	-
	45	Henskens, Nauta et al. 2018[45]	Dementia	21	Multidomain interventions	85.70%	90.50%	86.05 (5.86)	6 months	-	-	5.19 (0.84)	-
				22	Exercise	72.70%	77.30%	85.14 (4.64)		-	-	4.45 (1.71)	-
				22	Control	72.70%	77.30%	84.73 (4.55)		-	-	4.57 (2.04)	-
	46	Song and Yu 2019[46]	Cognitive impairment	60	Aerobic-exercise	80.00%	80.00%	76.22 (5.76)	16 weeks	-	-	5.33 (3.48)	-
				60	Control care	78.30%	70.00%	75.33 (6.78)		-	-	5.67 (3.70)	-
	47	Lam, Chan et al. 2015[47]	Cognitive impairment	145	Cognitive stimulation therapy	79.00%	79.30%	74.4(6.4)	12 months	25.7 (2.4)	0.6 (2.2)	-	-

				147	Physical Execise	78.00%	76.90%	75.5(6.7)		25.8 (2.3)	0.7 (2.6)	-	-
**Tai Chi**	48	Cheng, Chow et al. 2012[48]	Dementia	12	Tai Chi	100.00%	50.00%	81.0 (7.7)	6months	-	-	9.25 (2.14)	-
				12	Control care	100.00%	75.00%	82.5 (7.1)		-	-	9.08 (2.11)	-
	49	Lam, Chau et al. 2012[49]	Cognitive impairment	218	Tai Chi	77.52%	78.00%	78.3 (6.6)	12 months	24.3 (2.9)	0.8 (1.8)	-	1.3 (2.2)
				171	Exercise	53.80%	73.10%	77.2 (6.3)		24.7 (3.0)	0.8 (1.6)	-	1.2 (2.4)
**Hormone Therapy**	50	Wang, Liao et al. 2000[50]	Alzheimer's disease	25	Estrogen	96.00%	100.00%	72.6 (9.1)	12 weeks	16.1 (4.3)	-	-	-
				25	Placebo	92.00%	100.00%	71.0 (9.1)		16.2 (4.2)	-	-	-
	51	Valen-Sendstad, Engedal et al. 2010[51]	Alzheimer's disease	33	1mg estradiol; 0.5mg norethisterone	79.00%	100.00%	81.0 (5.7)	12 months	22.0 (4.3)	-	4.2 (0.6)	-
				32	Placebo	72.00%	100.00%	81.0 (4.5)		21.8 (3.9)	-	4.4 (0.6)	-
**Light therapy**	52	Dowling, Graf et al. 2007[52]	Alzheimer's disease	29	Morning light	-	81.40%	84 (10)	11 weeks	-	-	-	29.4 (20.7)
				24	Afternoon light					-	-	-	27.0 (15.7)
				17	Control care					-	-	-	24.1 (15.8)

	53	Riemersma-Van Der Lek, Swaab et al. 2008[53]	Dementia	49	Light therapy	95.9/87.8/67.3	91.80%	85.8 (5.5)	12 months	14.5 (6.2)	7.4 (6.9)	-	-
				45	Placebo	88.9/68.9/48.9	88.90%			14.3 (7)	7.6 (5.1)	-	-
	54	Burns, Allen et al. 2009[54]	Dementia	22	Light therapy	100.00%	73.00%	84.5 (1.7)	8 weeks	6.9 (5.3)	10.1 (5.2)	-	-
				40	Control care	87.50%				-	21.3 (3.4)	-	-
**Massage**	55	Yang, Wang et al. 2016[55]	Dementia	29	Massage (Aromatherapy)	93.10%	65.50%	83.34 (6.41)	9 weeks	-	12.89 (8.37)	-	-
				30	Control care	96.70%	56.70%	80.67 (7.44)		-	5.48 (4.37)	-	-
	56	Lanza, Centonze et al. 2018[56]	Alzheimer's disease	6	Massage (Shiatsu)	100.00%	83.30%	77 (8)	10 months	17.5 (0.5)	-	13(2)	-
				6	Control care	100.00%	100.00%	80 (7)		16.9 (0.7)	-	13 (1.0)	-
	57	Moyle, Cooke et al. 2014[57]	Dementia	26	Foot Massage	96.20%	66.30%	86.5 (7.13)	3 weeks	-	-	-	-
				29	Control care	96.60%				-	-	-	-
**Multidomain interventions**	58	Baldwin, Pratt et al. 2004[58]	Cognitive impairment	77	Multidomain intervention	76.60%	70.10%	80.6 (7.2)	8 weeks	18.2 (6.4)	-	14.4 (6.8)	-
				76	Control care	80.30%	57.90%	80.0 (7.5)		18.8 (6.9)	-	14.0 (6.6)	-
	59	Callahan, Boustani et al. 2006[59]	Alzheimer's disease	84	Collaborative Care	76.20%	46.40%	77.4 (5.9)	1 year	18.6 (5.9)	4.4 (4.9)	-	10.5 (15.3)

			69	Control care	89.90%	3.91%	77.7 (5.7)		17.5 (5.2)	5.4 (5.9)	-	13.4 (21.2)
60	Chapman and Toseland 2007[60]	Dementia	57	Advanced Illness Care	100.00%	94.70%	84.82 (6.8)	8 weeks	7.26 (7.575)	0.11 (0.14)	5.49 (0.869)	-
			61	Control care	100.00%	98.40%	88.0 (6.7)		6.8 (7.797)	0.08 (0.08)	5.66 (0.772)	-
61	Onor, Trevisiol et al. 2007[61]	Alzheimer's disease	8	Multimodal Rehabilitative Intervention	100.00%	37.50%	68.0 (6.5)	4 months	23.12 (4.15)	-	9.62 (3.73)	-
			8	Control care	100.00%	50.00%	72 (5.2)		20.00 (2.20)	-	12.87 (4.64)	-
62	Samus, Johnston et al. 2014[62]	Cognitive Impairment	110	Comprehensive home-based care intervention	100.00%	66.40%	84.0 (5.8)	18 months	19.0 (7.9)	6.5 (0.48)	-	6.9 (0.58)
			193	Control care	100.00%	62.20%	83.9 (5.9)		19.2 (7.7)	6.1 (0.37)	-	6.9 (0.44)
63	Eggermont, Knol et al. 2009[63]	Dementia	34	Hand movements group	88.23%	-	84.4 (5.2)	6 weeks	15.3 (3.2)	-	7.28 (5.71)	-
			32	Control care	96.87%		84.1 (4.8)		16.5 (4.7)	-	7.20 (5.52)	-
64	Hutson, Orrell et al. 2014[64]	Dementia	21	Sonas	95.23%	86.10%	86.6 (6.7)	7-8 weeks	-	5.10 (3.34)	-	17.20 (14.42)
			18	Control care	88.89%				-	5.44 (4.82)	-	10.06 (15.34)
65	Davison, Nayer et al. 2016[65]	Dementia	8	Memory Box intervention	100.00%	-	86.0 (5.2)	8 weeks	16.2 (4.6)	5.7 (3.1)	-	-

				8	Control care	100.00%					5.2 (5.9)	-	-
	66	Bae, Lee et al. 2019[66]	Cognitive Impairment	41	multicomponent intervention	-	43.90%	75.5 (6.0)	24 weeks	27.1 (2.1)	-	2.9 (2.2)	-
				42	Control care		52.40%	76.4 (5.1)		26.7 (2.0)	-	2.9 (2.7)	-
**Occupational therapy**	67	Graff, Vernooij-Dassen et al. 2007[67]	Dementia	68	Community Occupational Therapy	85.30%	57.40%	79.1 (6.2)	6 weeks	19.0 (5.7)	8.3 (6.2)	6.9 (3.0)	-
				67	Control care	83.60%	53.70%	77.1 (6.3)		19.0 (4.0)	8.1 (4.6)	7.5 (3.0)	-
	68	Lam, Lee et al. 2010[68]	Dementia	59	Case management by occupational therapist	89.80%	59.00%	78.6 (6.4)	4 months	17.6 (5.2)	3.0 (1.0, 6.0)	-	14.0 (5.0, 29.5)
				43	Control care	90.70%	56.00%	78.2 (5.4)		18.0 (5.1)	4.0 (1.0, 7.0)	-	17.0 (6.0, 35.0)
	69	Wenborn, Challis et al. 2013[69]	Dementia	104	Occupational therapy	76.00%	63.50%	84.2 (7.6)	12 weeks	5.8 (5.1)	7.6 (4.6)	-	-
				106	Control care	75.50%	70.80%	84.2 (7.6)		5.5 (4.6)	7.4 (4.9)	-	-
**Music therapy**	70	Guetin, Portet et al. 2009[70]	Alzheimer's Disease	15	Music therapy	93.30%	86.70%	85.2 (6)	16weeks	19.8 (4.4)	-	16.7 (6.2)	-
				15	Control care	80.00%	60.00%	86.9 (5.2)		20.7 (3.4)	-	11.8 (7.4)	-

	71	Cooke, Moyle et al. 2010[71]	Dementia	24	Music therapy	95.80%	70.20%	-	8 weeks	16.51 (6.737)	-	3.63 (2.25, 5.00)	-
				23	Control care	91.30%					-	3.96 (2.61, 5.30)	-
	72	Chu, Yang et al. 2014[72]	Dementia	52	Music therapy	94.20%	53.00%	82 (6.8)	6 weeks	12.80 (6.15)	17.39 (9.56)	-	-
				52	Control care	98.08%				13.76 (5.36)	15.70 (10.16)	-	-
	73	Raglio, Bellandi et al. 2015[73]	Dementia	40	Music therapy	77.50%	72.50%	81.0 (7.6)	10 weeks	11.1 (5.4)	-	-	33.1 (16.2)
				40	Control care	87.50%	82.50%	82.4 (6.8)		11.3 (5.3)	-	-	36.7 (19.2)
**Pet/robot-assisted therapy**	74	Joranson, Pedersen et al. 2015[74]	Dementia	30	Robot-Assisted therapy	90.00%	70.00%	83.9 (7.2)	12 weeks	9.0 (4.9)	-	-	-
				30	Control care	86.70%	63.30%	84.1 (6.7)		6.9 (4.7)	-	-	-
	75	Majic, Gutzmann et al. 2013[75]	Dementia	27	Pet-Assisted Therapy	100.00%	66.70%	81.33 (10.2)	10 weeks	6.37 (5.41)	-	-	-
				27	Control care	100.00%	74.10%	82.07 (8.65)		7.63 (5.94)	-	-	-
**Psychosoical therapy**	76	Kotynia-English, McGowan et al. 2005[76]	Cognitive impairment	53	Psychosoical therapy	86.80%	69.80%	82.9 (6.3)	12 months	16.9 (7.7)	-	4.9 (3.4)	14.2 (17.0)
				53	Control care	86.80%	64.10%	84.6 (8.1)		16.9 (7.7)	-	4.8 (3.4)	15.1 (16.0)

	77	de Rotrou, Cantegreil et al. 2011[77]	Alzheimer's disease	79	Psychosoical therapy	78.50%	63.30%	78.6 (6.1)	3 months	-	-	-	20.21 (17.67)
				78	Control care	79.50%	56.40%	78.7 (6.6)		-	-	-	17.72 (15.91)
	78	Waldorff, Buss et al. 2012[78]	Alzheimer's disease	163	Psychosoical therapy	80.36%	53.00%	76.5 (7.7)	6 months	-	-	4.74 (5.16)	3.90 (3.61)
				167	Control care	86.80%	55.00%	75.9 (6.6)		-	-	4.71 (5.02)	3.90 (3.65)
	79	Koivisto, Hallikainen et al. 2016[79]	Alzheimer's disease	84	Psychosoical therapy	64.00%	52.00%	75.8 (7.13)	36 months	21.8 (3.5)	-	-	8.5 (9.58)
				152	Control care	50.00%	50.00%	75.5 (6.19)		21.3 (3.4)	-	-	9.1 (9.75)
**Reminiscence therapy**	80	Wang 2007[80]	Dementia	51	Reminiscence therapy	86.30%	52.90%	79.76 (6.29)	8 months	14.33 (3.99)	7.37 (5.09)	7.09 (3.99)	-
				51	Control care	94.12%	49.00%	78.92 (7.64)		14.33 (4.05)	7.31 (4.98)	6.61 (3.48)	-
	81	Lee, Yip et al. 2013[81]	Alzheimer's disease	7	Computer-assisted memory training	100.00%	85.70%	77.7 (6.07)	6 weeks	17.00 (3.58)	-	2.17 (1.17)	-
				6	Therapist-led memory training	100.00%	50.00%			17.67 (4.76)	-	1.67 (2.73)	-
				6	Control care	100.00%	66.70%			15.29 (2.75)	-	2.57 (2.30)	-
	82	Van Bogaert, Van Grinsven et al. 2013[82]	Alzheimer's disease	41	Individual Reminiscence	100.00%	90.20%	83	4 weeks	18.39 (3.4)	5.71 (3.6)	6.90 (3.3)	8.22 (9.4)

				41	Control care	100.00%	75.60%	85		18.27 (4.5)	5.90 (4.8)	8.39 (3.9)	11.07 (5.6)
	83	Van Bogaert, Tolson et al. 2016[83]	Dementia	36	Individual Reminiscence	100.00%	82.80%	84	10 weeks	15 (11.48)	-	-	5 (32.14)
				36	Control care	100.00%	77.40%	84		18 (10.71)	-	-	3 (13.78)
	84	Duru Asiret and Kapucu 2016[84]	Alzheimer's disease	31	Reminiscence therapy	100.00%	67.70%	81.83 (4.87)	12 weeks	15.65 (2.49)	-	15.61 (3.06)	-
				31	Control care	100.00%	67.70%	82.26 (5.07)		14.16 (2.14)	-	15.93 (4.35)	-
	85	Lopes, Afonso et al. 2016[85]	Cognitive impairment	20	Reminiscence therapy	100.00%	75.00%	83.85 (6.991)	5 weeks	-	8.35 (8.119)	1.85 (1.387)	-
				21	Control care	95.23%	76.20%	83.62 (8.352)		-	9.86 (7.136)	2.52 (1.537)	-
	86	Lok, Bademli et al. 2019[86]	Alzheimer's disease	30	Reminiscence therapy	100.00%	60.00%	60+	8 weeks	18.26 (3.69)	10.66 (3.61)	-	-
				30	Control care	100.00%	53.40%			18.83 (3.00)	11.43 (3.78)	-	-
**Sertraline**	87	Munro, Longmire et al. 2012[87]	Alzheimer's disease	67	Sertraline 100 mg	94.02%	54.00%	79.0	24 weeks	21 (16.70)	-	-	-
				64	Placebo	95.31%				19.5 (16.8)	-	-	-
	88	Lyketsos, Sheppard et al. 2000[88]	Alzheimer's disease	12	Sertraline 150 mg	100.00%	59.00%	77 (8.4)	9 weeks	17.4 (6.5)	21.2 (6.4)	-	-
				10	Placebo	100.00%				14.2 (7.3)	17.5 (2.9)	-	-

	89	Lyketsos, Delcampo et al. 2003[89]	Alzheimer's disease	24	Sertraline 95 mg	87.50%	83.00%	75.5 (9.5)	12 weeks	17.5 (6.3)	18.1 (3.9)	-	36.8 (22.1)
				20	Placebo	75.00%		79.9 (5.2)		16.3 (6.8)	20.2 (5.4)	-	34.9 (14.8)
	90	Banerjee, Hellier et al. 2011[90]	Dementia	107	Sertraline 150 mg	63.50%	50.00%	80 (8.4)	39 weeks	18.5 (6.7)	12.8 (3.6)	-	26·9 (16·8)
				111	Placebo	73.90%	68.00%	79 (8.8)		18.2 (7.4)	13.6 (5.2)	-	30·2 (17·6)
	91	Mokhber, Abdollahian et al. 2014[91]	Alzheimer's disease	20	Sertraline 150 mg	100.00%	64.00%	67.3 (3.0)	12 weeks	15.05	-	-	-
				20	Venlafaxine 37.5 mg	100.00%	33.50%	67.9 (2.8)		14.7	-	-	-
	92	Zuidersma, Chua et al. 2019[92]	Alzheimer's disease	12	Mirtazapine 45g	83.33%	64.50%	78.0 (10.4)	13 weeks	-	19.0 (3.6)	-	-
				8	Sertraline 150mg	75.00%				-	19.3 (2.1)	-	-
				13	Placebo	84.60%				-	22.8 (7.2)	-	-
			Alzheimer's disease	28	Mirtazapine 45g	78.60%	62.80%	79.1 (7.9)		-	-	-	-
				29	Sertraline 150mg	86.20%				-	-	-	-
				28	Placebo	78.60%				-	-	-	-
			Alzheimer's disease	42	Mirtazapine 45g	78.60%	64.30%	79.5 (8.6)		-	-	-	-
				42	Sertraline 150mg	66.70%				-	-	-	-
				45	Placebo	88.90%				-	-	-	-

			Alzheimer's disease	24	Mirtazapine 45g	87.50%	80.50%	80.1 (98.1)		-	-	-	-
				27	Sertraline 150mg	70.40%				-	-	-	-
				24	Placebo	83.33%				-	-	-	-
	93	Oslin, Ten Have et al. 2003[93]	Dementia	25	Sertraline 100mg	100.00%	56.00%	83.8 (9.8)	10 weeks	21.9 (4.9)	20.9 (4.4)	16.9 (6.2)	-
				27	Venlafaxine 150mg	100.00%	33.00%	81.2 (10.8)		22.0 (5.4)	20.2 (4.1)	17.1 (5.8)	-
	94	Magai, Kennedy et al. 2000[94]	Alzheimer's disease	17	Sertraline 100mg	100.00%	100.00%	88.4 (6.1)	8 weeks	-	5.93 (1.94)	0.53 (0.92)	-
				14	Placebo	100.00%	100.00%	90.1 (6.5)		-	6.33 (2.31)	1.00 (0.95)	-
	95	Weintraub, Rosenberg et al. 2010[95]	Alzheimer's disease	67	Sertraline 100mg	95.50%	54.00%	79.0	24 weeks	21 (17-25)	-	-	-
				64	Placebo	93.80%				19.5 (15-23.25)	-	-	-
	96	Rosenberg, Drye et al. 2010[96]	Alzheimer's disease	67	Sertraline 100mg	98.50%	59.70%	76.5 (8.0)	-	-	-	-	-
				64	Placebo	98.40%	48.40%	78.2 (8.0)		-	-	-	-
**Venlafaxine**	97	de Vasconcelos Cunha, Lopes Rocha et al. 2007[97]	Dementia	14	Venlafaxine 75 mg	57.14%		77.6 (7.3)	6 weeks	20.6 (4.5)	13 (9, 19)	-	-
				17	Placebo	82.40%	64.70%	77.6 (5.8)		19.3 (4.8)	13 (9.5, 17)	-	-

**Imipramine**	98	REIFLER, BV et al. 1989[98]	Alzheimer's disease	13	Imipramine 25mg	100.00%	-	72 (8)	8 weeks	16.9 (4.6)	-	-	-
				15	Placebo	100.00%				18 (5.5)	-	-	-
			Alzheimer's disease	14	Imipramine 25mg	100.00%				13.4 (6.9)	-	-	-
				19	Placebo	100.00%				14.8 (15.1)	-	-	-
	99	Linda, Reifler et al. 1991[99]	Alzheimer's disease	13	Imipramine 25mg	100.00%	76.90%	76 (7)	8 weeks	-	-	-	-
				15	Placebo	100.00%	60.00%	71 (9)		-	-	-	-
			Alzheimer's disease	14	Imipramine 25mg	100.00%	50.00%	68 (7)		-	-	-	-
				19	Placebo	100.00%	52.60%	71 (8)		-	-	-	-
**Fluoxetine**	100	Taragano, Lyketsos et al. 1997[100]	Alzheimer's disease	18	Fluoxetine 10mg	100.00%	77.80%	71.7 (5.0)	45 days	20.0 (3.2)	-	-	-
				19	Amitriptyline 25mg	100.00%	78.90%	72.4 (4.9)		18.8 (4.2)	-	-	-
	101	Petracca, Chemerinski et al. 2001[101]	Alzheimer's disease	17	Fluoxetine 40mg	88.20%	47.00%	70.2 (6.3)	6 weeks	23.2 (4.5)	-	-	-
				24	Placebo	83.30%	71.00%	71.3 (6.9)		23.2 (5.3)	-	-	-
	102	Auchus and Bissey-Black 1997[102]	Alzheimer's disease	6	Fluoxetine 40mg	100.00%	66.70%	75.6 (7.5)	7 weeks	15.2 (4.6)	-	-	-
				6	Placebo	100.00%	66.70%				-	-	-
**Amitriptyline**	103	Reding, Young et al. 1983[103]	Alzheimer's disease	9	Amitriptyline 25mg	100.00%	63.60%	68.0	6 weeks	18 (3)	-	-	-

				8	Placebo	100.00%				16 (3)	-	-	-
**Rivastigmine**	104	Moretti, Torre et al. 2003[104]	Vascular dementia	104	Rivastigmine 3-6mg	100.00%	52.90%	75.67 (2.98)	12 months	19.75 (2.38)	-	13.46 (3.01)	-
				104	Placebo	100.00%				20.23 (2.45)	-	13.44 (2.31)	-
	105	Mowla, Mosavinasab et al. 2007[105]	Alzheimer's disease	41	Rivastigmine 6-12 mg	82.90%	53.50%	69.2	12 weeks	16.3 (4.1)	-	-	-
				40	Placebo	80.00%				16.5 (3.6)	-	-	-
	106	Nasab, Bahrammi et al. 2012[105]	Alzheimer's dementia	28	Rivastigmine 4.5 mg	76.00%	57.60%	65.72 (4.69)	24 weeks	-	-	-	-
				28	EGB 761 120 mg	80.00%	52.00%	66.03 (4.64)		-	-	-	-
**Discontinuation of antidepressants**	107	Bergh, Selbaek et al. 2012[106]	Dementia	63	Discontinued treatment	56.00%	78.00%	85.3 (8.2)	25 weeks	-	1.28 (1.71)	-	17.78 (16.75)
				65	Continued treatment	71.00%	72.00%	86.1 (6.7)		-	1.95 (2.40)	-	17.63 (14.09)

Reference

1. Husebo BS, Ballard C, Fritze F, Sandvik RK, Aarsland D. Efficacy of pain treatment on mood syndrome in patients with dementia: a randomized clinical trial. *International Journal of Geriatric Psychiatry.* 2013;29(8):828-836.
2. Husebo BS, Ballard C, Sandvik R, Nilsen OB, Aarsland D. Efficacy of treating pain to reduce behavioural disturbances in residents of nursing homes with

dementia: cluster randomised clinical trial. *BMJ.* 2011;343:d4065.

3. Streim JE, Porsteinsson AP, Breder CD, et al. A randomized, double-blind, placebo-controlled study of aripiprazole for the treatment of psychosis in nursing home patients with Alzheimer disease. *American Journal of Geriatric Psychiatry Official Journal of the American Association for Geriatric Psychiatry.* 2008;16(7):537.
4. Mintzer JE, Tune LE, Breder CD, et al. Aripiprazole for the treatment of psychoses in institutionalized patients with Alzheimer dementia: a multicenter, randomized, double-blind, placebo-controlled assessment of three fixed doses. *American Journal of Geriatric Psychiatry Official Journal of the American Association for Geriatric Psychiatry.* 2007;15(11):918-931.
5. De Deyn P, Jeste DV, Swanink R, et al. Aripiprazole for the treatment of psychosis in patients with Alzheimer's disease: a randomized, placebo-controlled study. *J Clin Psychopharmacol.* 2005;25(5):463-467.
6. Travers C. Increasing enjoyable activities to treat depression in nursing home residents with dementia: A pilot study. *Dementia.* 2016;16(2):204-218.
7. Linda T, Logsdon RG, Jay U, Mccurry SM. Behavioral Treatment of Depression in Dementia Patients: A Controlled Clinical Trial. *Journals of Gerontology.* 1997(4):P159-166.
8. Lichtenberg PA, Kemp-Havican J, Macneill SE, Schafer Johnson A. Pilot study of behavioral treatment in dementia care units. *Gerontologist.*45(3):406-410.
9. Lam LC, Lui VW, Luk DN, et al. Effectiveness of an individualized functional training program on affective disturbances and functional skills in mild and moderate dementia--a randomized control trial. *Int J Geriatr Psychiatry.* 2010;25(2):133-141.
10. Verkaik R, Francke AL, van Meijel B, Spreeuwenberg PM, Ribbe MW, Bensing JM. The effects of a nursing guideline on depression in psychogeriatric nursing home residents with dementia. *Int J Geriatr Psychiatry.* 2011;26(7):723-732.
11. Tse MMY, Lau JL, Kwan R, et al. Effects of play activities program for nursing home residents with dementia on pain and psychological well-being: Cluster randomized controlled trial. *Geriatr Gerontol Int.* 2018;18(10):1485-1490.
12. Todri J, Todri A, Lena O. Why Not a Global Postural Reeducation as an Alternative Therapy Applied to Alzheimer's Patients in Nursing Homes? A Pioneer Randomized Controlled Trial. *Dement Geriatr Cogn Disord.* 2019;48(3-4):172-179.
13. Teri L, Gibbons LE, McCurry SM, et al. Exercise plus behavioral management in patients with Alzheimer disease: a randomized controlled trial. *Jama.* 2003;290(15):2015-2022.
14. Woods B, Thorgrimsen L, Spector A, Royan L, Orrell M. Improved quality of life and cognitive stimulation therapy in dementia. *Aging & Mental Health.* 2006;10(3):219-226.
15. Yi-Xuan, Niu, Ji-Ping, et al. Cognitive stimulation therapy in the treatment of neuropsychiatric symptoms in Alzheimer's disease: a randomized controlled

trial. *Clinical Rehabilitation.* 2010;24(12):1102-1111.

16. Buschert VC, Friese U, Teipel SJ, et al. Effects of a newly developed cognitive intervention in amnestic mild cognitive impairment and mild Alzheimer's disease: a pilot study. *J Alzheimers Dis.* 2011;25(4):679-694.
17. Maci T, Pira FL, Quattrocchi G, Nuovo SD, Perciavalle V, Zappia M. Physical and cognitive stimulation in Alzheimer Disease. the GAIA Project: a pilot study. *Am J Alzheimers Dis Other Demen.* 2012;27(2):107-113.
18. Bergamaschi S, Arcara G, Calza A, Villani D, Orgeta V, Mondini S. One-year repeated cycles of cognitive training (CT) for Alzheimer's disease. *Aging Clin Exp Res.* 2013;25(4):421-426.
19. Jha A, Jan F, Gale T, Newman C. Effectiveness of a recovery-orientated psychiatric intervention package on the wellbeing of people with early dementia: a preliminary randomised controlled trial. *Int J Geriatr Psychiatry.* 2013;28(6):589-596.
20. Stanley MA, Calleo J, Bush AL, et al. The peaceful mind program: a pilot test of a cognitive-behavioral therapy-based intervention for anxious patients with dementia. *Am J Geriatr Psychiatry.* 2013;21(7):696-708.
21. Orgeta V, Leung P, Yates L, et al. Individual cognitive stimulation therapy for dementia: a clinical effectiveness and cost-effectiveness pragmatic, multicentre, randomised controlled trial. *Health Technol Assess.* 2015;19(64):1-108.
22. Amieva H, Robert PH, Grandoulier A-S, et al. Group and individual cognitive therapies in Alzheimer's disease: the ETNA3 randomized trial. *International Psychogeriatrics.* 2015;28(5):707-717.
23. Capotosto E, Belacchi C, Gardini S, et al. Cognitive stimulation therapy in the Italian context: its efficacy in cognitive and non-cognitive measures in older adults with dementia. *Int J Geriatr Psychiatry.* 2017;32(3):331-340.
24. Churcher Clarke A, Chan JMY, Stott J, Royan L, Spector A. An adapted mindfulness intervention for people with dementia in care homes: feasibility pilot study. *Int J Geriatr Psychiatry.* 2017;32(12):e123-e131.
25. Khedr EM, Salama RH, Abdel Hameed M, Abo Elfetoh N, Seif P. Therapeutic Role of Transcranial Direct Current Stimulation in Alzheimer Disease Patients: Double-Blind, Placebo-Controlled Clinical Trial. *Neurorehabil Neural Repair.* 2019;33(5):384-394.
26. Luijpen MW, Swaab DF, Sergeant JA, Scherder EJ. Effects of transcutaneous electrical nerve stimulation (TENS) on self-efficacy and mood in elderly with mild cognitive impairment. *Neurorehabil Neural Repair.* 2004;18(3):166-175.
27. Nyth AL, Gottfries CG. The clinical efficacy of citalopram in treatment of emotional disturbances in dementia disorders. A Nordic multicentre study. *British Journal of Psychiatry the Journal of Mental ence.* 1990;157(6):894.
28. Nyth AL, Gottfries CG, Lyby K, et al. A controlled multicenter clinical study of citalopram and placebo in elderly depressed patients with and without

concomitant dementia. *Acta Psychiatr Scand.* 1992;86(2):138-145.
29. An H, Choi B, Park KW, et al. The Effect of Escitalopram on Mood and Cognition in Depressive Alzheimer's Disease Subjects. *J Alzheimers Dis.* 2017;55(2):727-735.
30. Porsteinsson AP, Drye LT, Pollock BG, et al. Effect of citalopram on agitation in Alzheimer disease: the CitAD randomized clinical trial. *JAMA.* 2014;311(7):682-691.
31. Devanand DP, Pelton GH, D'Antonio K, et al. Donepezil Treatment in Patients With Depression and Cognitive Impairment on Stable Antidepressant Treatment: A Randomized Controlled Trial. *Am J Geriatr Psychiatry.* 2018;26(10):1050-1060.
32. Yancheva S, Ihl R, Nikolova G, et al. Ginkgo biloba extract EGb 761(R), donepezil or both combined in the treatment of Alzheimer's disease with neuropsychiatric features: a randomised, double-blind, exploratory trial. *Aging Ment Health.* 2009;13(2):183-190.
33. Reynolds CF, 3rd, Butters MA, Lopez O, et al. Maintenance treatment of depression in old age: a randomized, double-blind, placebo-controlled evaluation of the efficacy and safety of donepezil combined with antidepressant pharmacotherapy. *Arch Gen Psychiatry.* 2011;68(1):51-60.
34. Van Dongen MCJM, Van Rossum E, Kessels AGH, Sielhorst HJG, Knipschild PG. The efficacy of ginkgo for elderly people with dementia and age-associated memory impairment: new results of a randomized clinical trial. *Journal of the American Geriatrics Society.* 2000;48(10):1183–1194.
35. Napryeyenko O, Sonnik G, Tartakovsky I. Efficacy and tolerability of Ginkgo biloba extract EGb 761 by type of dementia: analyses of a randomised controlled trial. *J Neurol Sci.* 2009;283(1-2):224-229.
36. Kanowski S, Hoerr R. Ginkgo biloba extract EGb 761 in dementia: intent-to-treat analyses of a 24-week, multi-center, double-blind, placebo-controlled, randomized trial. *Pharmacopsychiatry.* 2003;36(6):297-303.
37. Napryeyenko O, Borzenko I. Ginkgo biloba special extract in dementia with neuropsychiatric features. A randomised, placebo-controlled, double-blind clinical trial. *Arzneimittelforschung.* 2007;57(1):4-11.
38. Liu IT, Lee WJ, Lin SY, Chang ST, Kao CL, Cheng YY. Therapeutic Effects of Exercise Training on Elderly Patients With Dementia: A Randomized Controlled Trial. *Arch Phys Med Rehabil.* 2020;101(5):762-769.
39. Rolland Y, Pillard F, Klapouszczak A, et al. Exercise program for nursing home residents with Alzheimer's disease: a 1-year randomized, controlled trial. *J Am Geriatr Soc.* 2007;55(2):158-165.
40. Bostrom G, Conradsson M, Hornsten C, et al. Effects of a high-intensity functional exercise program on depressive symptoms among people with dementia in residential care: a randomized controlled trial. *Int J Geriatr Psychiatry.* 2016;31(8):868-878.
41. Cancela JM, Ayan C, Varela S, Seijo M. Effects of a long-term aerobic exercise intervention on institutionalized patients with dementia. *J Sci Med Sport.*

2016;19(4):293-298.

42. Hoffmann K, Sobol NA, Frederiksen KS, et al. Moderate-to-High Intensity Physical Exercise in Patients with Alzheimer's Disease: A Randomized Controlled Trial. *J Alzheimers Dis.* 2016;50(2):443-453.
43. Chen KM, Kuo CC, Chang YH, Huang HT, Cheng YY. Resistance Band Exercises Reduce Depression and Behavioral Problems of Wheelchair-Bound Older Adults with Dementia: A Cluster-Randomized Controlled Trial. *J Am Geriatr Soc.* 2017;65(2):356-363.
44. Morris JK, Vidoni ED, Johnson DK, et al. Aerobic exercise for Alzheimer's disease: A randomized controlled pilot trial. *PLoS One.* 2017;12(2):e0170547.
45. Henskens M, Nauta IM, van Eekeren MCA, Scherder EJA. Effects of Physical Activity in Nursing Home Residents with Dementia: A Randomized Controlled Trial. *Dement Geriatr Cogn Disord.* 2018;46(1-2):60-80.
46. Song D, Yu DSF. Effects of a moderate-intensity aerobic exercise programme on the cognitive function and quality of life of community-dwelling elderly people with mild cognitive impairment: A randomised controlled trial. *Int J Nurs Stud.* 2019;93:97-105.
47. Lam LC, Chan WC, Leung T, Fung AW, Leung EM. Would older adults with mild cognitive impairment adhere to and benefit from a structured lifestyle activity intervention to enhance cognition?: a cluster randomized controlled trial. *PLoS One.* 2015;10(3):e0118173.
48. Cheng ST, Chow PK, Yu EC, Chan AC. Leisure activities alleviate depressive symptoms in nursing home residents with very mild or mild dementia. *Am J Geriatr Psychiatry.* 2012;20(10):904-908.
49. Lam LC, Chau RC, Wong BM, et al. A 1-year randomized controlled trial comparing mind body exercise (Tai Chi) with stretching and toning exercise on cognitive function in older Chinese adults at risk of cognitive decline. *J Am Med Dir Assoc.* 2012;13(6):568 e515-520.
50. Wang P, Liao S, Liu R, et al. Effects of estrogen on cognition, mood, and cerebral blood flow in AD: a controlled study. *Neurology.* 2000;54(11):2061-2066.
51. Valen-Sendstad A, Engedal K, Stray-Pedersen B, et al. Effects of hormone therapy on depressive symptoms and cognitive functions in women with Alzheimer disease: a 12 month randomized, double-blind, placebo-controlled study of low-dose estradiol and norethisterone. *The American Journal of Geriatric Psychiatry.* 2010;18(1):11-20.
52. Dowling GA, Graf CL, Hubbard EM, Luxenberg JS. Light treatment for neuropsychiatric behaviors in Alzheimer's disease. *Western journal of nursing research.* 2007;29(8):961-975.
53. Riemersma-Van Der Lek RF, Swaab DF, Twisk J, Hol EM, Hoogendijk WJ, Van Someren EJ. Effect of bright light and melatonin on cognitive and noncognitive function in elderly residents of group care facilities: a randomized controlled trial. *Jama.* 2008;299(22):2642-2655.
54. Burns A, Allen H, Tomenson B, Duignan D, Byrne J. Bright light therapy for agitation in dementia: a randomized controlled trial. *Int Psychogeriatr.* 2009;21(4):711-721.

55. Yang YP, Wang CJ, Wang JJ. Effect of aromatherapy massage on agitation and depressive mood in individuals with dementia. *Journal of gerontological nursing.* 2016;42(9):38-46.
56. Lanza G, Centonze SS, Destro G, et al. Shiatsu as an adjuvant therapy for depression in patients with Alzheimer's disease: A pilot study. *Complement Ther Med.* 2018;38:74-78.
57. Moyle W, Cooke ML, Beattie E, Shum DH, O'Dwyer ST, Barrett S. Foot massage versus quiet presence on agitation and mood in people with dementia: a randomised controlled trial. *Int J Nurs Stud.* 2014;51(6):856-864.
58. Baldwin R, Pratt H, Goring H, Marriott A, Roberts C. Does a nurse-led mental health liaison service for older people reduce psychiatric morbidity in acute general medical wards? A randomised controlled trial. *Age Ageing.* 2004;33(5):472-478.
59. Callahan CM, Boustani MA, Unverzagt FW, et al. Effectiveness of collaborative care for older adults with Alzheimer disease in primary care: a randomized controlled trial. *Jama.* 2006;295(18):2148-2157.
60. Chapman DG, Toseland RW. Effectiveness of advanced illness care teams for nursing home residents with dementia. *Social Work.* 2007;52(4):321-329.
61. Onor ML, Trevisiol M, Negro C, Signorini A, Saina M, Aguglia E. Impact of a multimodal rehabilitative intervention on demented patients and their caregivers. *Am J Alzheimers Dis Other Demen.* 2007;22(4):261-272.
62. Samus QM, Johnston D, Black BS, et al. A multidimensional home-based care coordination intervention for elders with memory disorders: the maximizing independence at home (MIND) pilot randomized trial. *Am J Geriatr Psychiatry.* 2014;22(4):398-414.
63. Eggermont LH, Knol DL, Hol EM, Swaab DF, Scherder EJ. Hand motor activity, cognition, mood, and the rest-activity rhythm in dementia: a clustered RCT. *Behav Brain Res.* 2009;196(2):271-278.
64. Hutson C, Orrell M, Dugmore O, Spector A. Sonas: a pilot study investigating the effectiveness of an intervention for people with moderate to severe dementia. *Am J Alzheimers Dis Other Demen.* 2014;29(8):696-703.
65. Davison TE, Nayer K, Coxon S, et al. A personalized multimedia device to treat agitated behavior and improve mood in people with dementia: A pilot study. *Geriatr Nurs.* 2016;37(1):25-29.
66. Bae S, Lee S, Lee S, et al. The effect of a multicomponent intervention to promote community activity on cognitive function in older adults with mild cognitive impairment: A randomized controlled trial. *Complement Ther Med.* 2019;42:164-169.
67. Graff MJ, Vernooij-Dassen MJ, Thijssen M, Dekker J, Hoefnagels WH, OldeRikkert MG. Effects of community occupational therapy on quality of life, mood, and health status in dementia patients and their caregivers: a randomized controlled trial. *The Journals of Gerontology Series A: Biological Sciences and Medical Sciences.* 2007;62(9):1002-1009.

68. Lam LC, Lee JS, Chung JC, Lau A, Woo J, Kwok TC. A randomized controlled trial to examine the effectiveness of case management model for community dwelling older persons with mild dementia in Hong Kong. *Int J Geriatr Psychiatry.* 2010;25(4):395-402.
69. Wenborn J, Challis D, Head J, et al. Providing activity for people with dementia in care homes: a cluster randomised controlled trial. *Int J Geriatr Psychiatry.* 2013;28(12):1296-1304.
70. Guetin S, Portet F, Picot MC, et al. Effect of music therapy on anxiety and depression in patients with Alzheimer's type dementia: randomised, controlled study. *Dement Geriatr Cogn Disord.* 2009;28(1):36-46.
71. Cooke M, Moyle W, Shum D, Harrison S, Murfield J. A randomized controlled trial exploring the effect of music on quality of life and depression in older people with dementia. *J Health Psychol.* 2010;15(5):765-776.
72. Chu H, Yang CY, Lin Y, et al. The impact of group music therapy on depression and cognition in elderly persons with dementia: a randomized controlled study. *Biol Res Nurs.* 2014;16(2):209-217.
73. Raglio A, Bellandi D, Baiardi P, et al. Effect of Active Music Therapy and Individualized Listening to Music on Dementia: A Multicenter Randomized Controlled Trial. *J Am Geriatr Soc.* 2015;63(8):1534-1539.
74. Joranson N, Pedersen I, Rokstad AM, Ihlebaek C. Effects on Symptoms of Agitation and Depression in Persons With Dementia Participating in Robot-Assisted Activity: A Cluster-Randomized Controlled Trial. *J Am Med Dir Assoc.* 2015;16(10):867-873.
75. Majic T, Gutzmann H, Heinz A, Lang UE, Rapp MA. Animal-assisted therapy and agitation and depression in nursing home residents with dementia: a matched case-control trial. *Am J Geriatr Psychiatry.* 2013;21(11):1052-1059.
76. Kotynia-English R, McGowan H, Almeida OP. A randomized trial of early psychiatric intervention in residential care: impact on health outcomes. *Int Psychogeriatr.* 2005;17(3):475-485.
77. de Rotrou J, Cantegreil I, Faucounau V, et al. Do patients diagnosed with Alzheimer's disease benefit from a psycho-educational programme for family caregivers? A randomised controlled study. *Int J Geriatr Psychiatry.* 2011;26(8):833-842.
78. Waldorff FB, Buss DV, Eckermann A, et al. Efficacy of psychosocial intervention in patients with mild Alzheimer's disease: the multicentre, rater blinded, randomised Danish Alzheimer Intervention Study (DAISY). *BMJ.* 2012;345:e4693.
79. Koivisto AM, Hallikainen I, Valimaki T, et al. Early psychosocial intervention does not delay institutionalization in persons with mild Alzheimer disease and has impact on neither disease progression nor caregivers' well-being: ALSOVA 3-year follow-up. *Int J Geriatr Psychiatry.* 2016;31(3):273-283.
80. Wang JJ. Group reminiscence therapy for cognitive and affective function of demented elderly in Taiwan. *Int J Geriatr Psychiatry.* 2007;22(12):1235-1240.
81. Lee GY, Yip CC, Yu EC, Man DW. Evaluation of a computer-assisted errorless learning-based memory training program for patients with early Alzheimer's

disease in Hong Kong: a pilot study. *Clin Interv Aging.* 2013;8:623-633.
82. Van Bogaert P, Van Grinsven R, Tolson D, Wouters K, Engelborghs S, Van der Mussele S. Effects of SolCos model-based individual reminiscence on older adults with mild to moderate dementia due to Alzheimer disease: a pilot study. *J Am Med Dir Assoc.* 2013;14(7):528 e529-513.
83. Van Bogaert P, Tolson D, Eerlingen R, et al. SolCos model-based individual reminiscence for older adults with mild to moderate dementia in nursing homes: a randomized controlled intervention study. *J Psychiatr Ment Health Nurs.* 2016;23(9-10):568-575.
84. Duru Asiret G, Kapucu S. The Effect of Reminiscence Therapy on Cognition, Depression, and Activities of Daily Living for Patients With Alzheimer Disease. *J Geriatr Psychiatry Neurol.* 2016;29(1):31-37.
85. Lopes TS, Afonso RM, Ribeiro OM. A quasi-experimental study of a reminiscence program focused on autobiographical memory in institutionalized older adults with cognitive impairment. *Arch Gerontol Geriatr.* 2016;66:183-192.
86. Lok N, Bademli K, Selcuk-Tosun A. The effect of reminiscence therapy on cognitive functions, depression, and quality of life in Alzheimer patients: Randomized controlled trial. *Int J Geriatr Psychiatry.* 2019;34(1):47-53.
87. Munro CA, Longmire CF, Drye LT, et al. Cognitive outcomes after sertaline treatment in patients with depression of Alzheimer disease. *Am J Geriatr Psychiatry.* 2012;20(12):1036-1044.
88. Lyketsos CG, Sheppard JME, Steele CD, et al. Randomized, placebo-controlled, double-blind clinical trial of sertraline in the treatment of depression complicating Alzheimer's disease: initial results from the Depression in Alzheimer's Disease study. *American Journal of Psychiatry.* 2000;157(10):1686-1689.
89. Lyketsos CG, Delcampo L, Steinberg M, et al. Treating Depression in Alzheimer Disease. *Archives of General Psychiatry.* 2003;60(7):737-746.
90. Banerjee S, Hellier J, Dewey M, et al. Sertraline or mirtazapine for depression in dementia (HTA-SADD): a randomised, multicentre, double-blind, placebo-controlled trial. *The Lancet.* 2011;378(9789):403-411.
91. Mokhber N, Abdollahian E, Soltanifar A, et al. Comparison of sertraline, venlafaxine and desipramine effects on depression, cognition and the daily living activities in Alzheimer patients. *Pharmacopsychiatry.* 2014;47(4-5):131-140.
92. Zuidersma M, Chua KC, Hellier J, Voshaar RO, Banerjee S, Group H-SI. Sertraline and Mirtazapine Versus Placebo in Subgroups of Depression in Dementia: Findings From the HTA-SADD Randomized Controlled Trial. *Am J Geriatr Psychiatry.* 2019;27(9):920-931.
93. Oslin DW, Ten Have TR, Streim JE, et al. Probing the safety of medications in the frail elderly: evidence from a randomized clinical trial of sertraline and venlafaxine in depressed nursing home residents. *J Clin Psychiatry.* 2003;64(8):875-882.
94. Magai C, Kennedy G, Cohen CI, Gomberg D. A controlled clinical trial of sertraline in the treatment of depression in nursing home patients with late-stage Alzheimer's disease. *Am J Geriatr Psychiatry.* 2000;8(1):66-74.

95. Weintraub D, Rosenberg PB, Drye LT, et al. Sertraline for the treatment of depression in Alzheimer disease: week-24 outcomes. *Am J Geriatr Psychiatry.* 2010;18(4):332-340.
96. Rosenberg PB, Drye LT, Martin BK, et al. Sertraline for the treatment of depression in Alzheimer disease. *Am J Geriatr Psychiatry.* 2010;18(2):136-145.
97. de Vasconcelos Cunha UG, Lopes Rocha F, Avila de Melo R, et al. A placebo-controlled double-blind randomized study of venlafaxine in the treatment of depression in dementia. *Dement Geriatr Cogn Disord.* 2007;24(1):36-41.
98. Reifler B. Double-blind trial of imipramine in Alzheimer's disease patients with and without depression. *American Journal of Psychiatry.* 1989;146.
99. Linda T, Reifler BV, Veith RC, et al. Imipramine in the treatment of depressed Alzheimer's patients: impact on cognition. *Journal of Gerontology.* (6):372-377.
100. Taragano FE, Lyketsos CG, Mangone CA, Allegri RF, Comesaña-Diaz E. A Double-Blind, Randomized, Fixed-Dose Trial of Fluoxetine vs. Amitriptyline in the Treatment of Major Depression Complicating Alzheimer's Disease. *Psychosomatics.* 1997;38(3):246-252.
101. Petracca GM, Chemerinski E, Starkstein SE. A double-blind, placebo-controlled study of fluoxetine in depressed patients with Alzheimer's disease. *International Psychogeriatrics.* 2001;13(2):233-240.
102. Auchus AP, Bissey-Black C. Pilot study of haloperidol, fluoxetine, and placebo for agitation in Alzheimer's disease. *The Journal of neuropsychiatry and clinical neurosciences.* 1997;9(4):591-593.
103. Reding MJ, Young R, Diponte P. Amitriptyline in Alzheimer's disease. *Neurology.* 1983;33(4):522-523.
104. Moretti R, Torre P, Antonello RM, Cazzato G, Bava A. Rivastigmine in subcortical vascular dementia: a randomized, controlled, open 12-month study in 208 patients. *Am J Alzheimers Dis Other Demen.* 2003;18(5):265-272.
105. Mowla A, Mosavinasab M, Haghshenas H, Borhani Haghighi A. Does serotonin augmentation have any effect on cognition and activities of daily living in Alzheimer's dementia? A double-blind, placebo-controlled clinical trial. *J Clin Psychopharmacol.* 2007;27(5):484-487.
106. Bergh S, Selbaek G, Engedal K. Discontinuation of antidepressants in people with dementia and neuropsychiatric symptoms (DESEP study): double blind, randomised, parallel group, placebo controlled trial. *BMJ.* 2012;344:e1566.
