# Supplementary material for: Comparative efficacy and acceptability of treatments for depressive symptoms in cognitive impairment: A systematic review and Bayesian network meta-analysis
Source: Front Aging Neurosci. 2022 Dec 12;14:1037414. doi: 10.3389/fnagi.2022.1037414 (PMC9790988; doi:10.3389/fnagi.2022.1037414)
Supplement: Supplementary material 1 — PRISMA NMA checklist. [file Data_Sheet_1.zip › Supplementary Material Presentation/Supplementary_4.pdf]

A. Cornell Scale for Depression

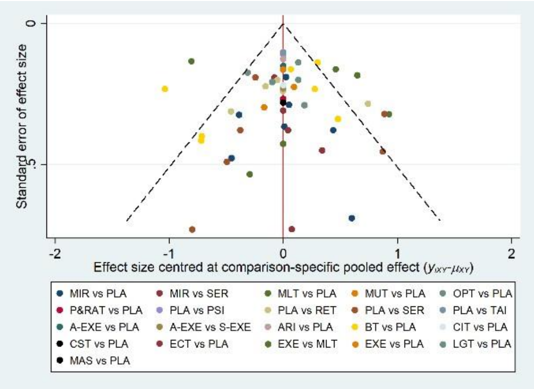

B. Geriatric Depression Scale

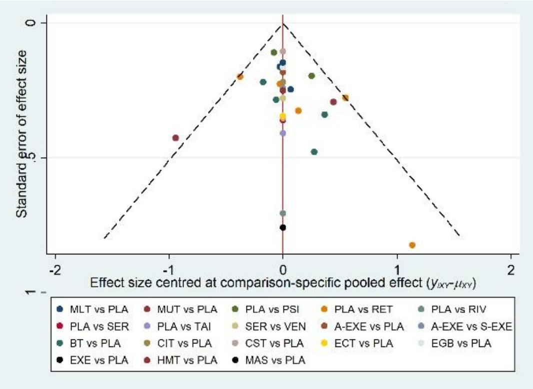

C. Hamilton Depression Rating Scale

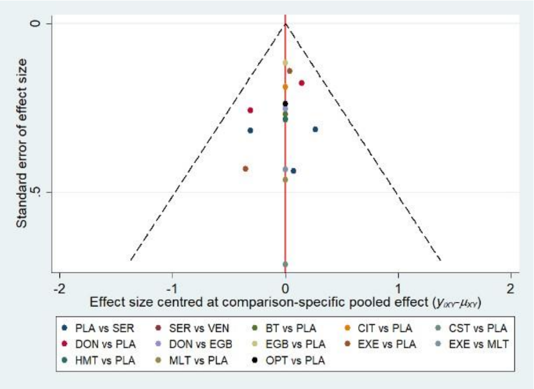

D. Mini-Mental State Examination

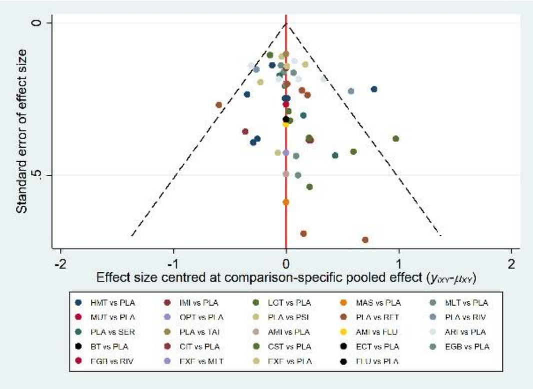

E. Neuropsychiatric Inventory

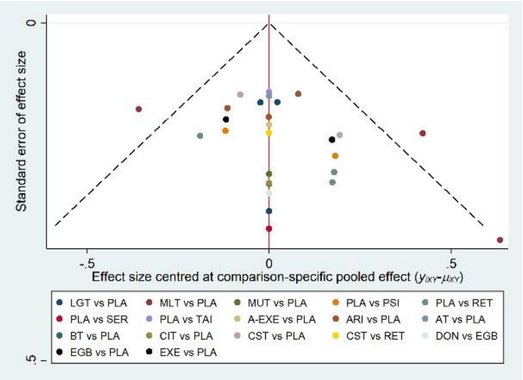

F. Cohen-Mansfeld Agitation Inventory

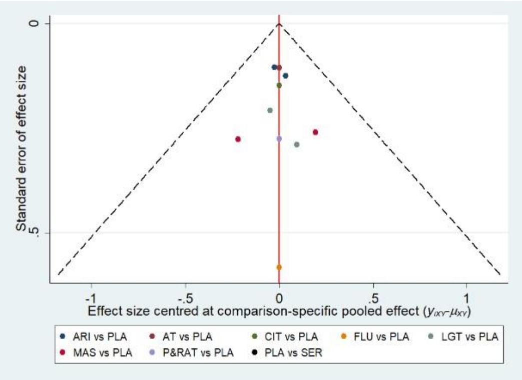

Abbreviations		
AEXE = Aerobic Exercise;	EGB = EGb 761;	OPT = Occupational Therapy;
AMI = Amitriptyline;	EXE = Exercise;	P/RAT = Pet/Robot-Assisted Therapy;
ARI = Aripiprazole;	FLU = Fluoxetine;	PLA = Placebo；
AT = Analgesic Treatment;	HMT = Hormone Therapy;	PST = Psychosocial Therapy;
BT = Behavioral Training;	IMI = Imipramine;	RET = Reminiscence Therapy;
CIT = Citalopram;	LGT = Light therapy;	RIV = Rivastigmie;
CST = Cognitive Stimulated Training;	MAS = Massage;	SEXE = Strength Exercise;
DIS = Discontinuation of Antipsychotics;	MIR = Mirtazapine;	SER = Sertraline;
DON = Donepezil;	MLT = Multidomain Therapy	TAI = Tai Chi;
ECT = Electrical Current Therapy；	MUT = Music Therapy;	VEN = Venlafaxine
