# Supplementary material for: Comparative efficacy and acceptability of treatments for depressive symptoms in cognitive impairment: A systematic review and Bayesian network meta-analysis
Source: Front Aging Neurosci. 2022 Dec 12;14:1037414. doi: 10.3389/fnagi.2022.1037414 (PMC9790988; doi:10.3389/fnagi.2022.1037414)
Supplement: Supplementary material 1 — PRISMA NMA checklist. [file Data_Sheet_1.zip › Supplementary Material Presentation/Supplementary_5.pdf]

Sensitivity and Heterogeneity Analysis						
	Model	Dbar	pD	DIC	Ratio	I^2
A. Cornell Scale for Depression	Random-Effect Model	104.6006	70.16961	174.7702	1.113	11%
	Fixed-Effect Model	108.7975	64.99007	173.7875	1.157	15%
B. Geriatric Depression Scale	Random-Effect Model	32.2721	30.64489	62.91699	1.009	4%
	Fixed-Effect Model	40.48003	27.00095	67.48098	1.265	23%
C. Hamilton Depression Rating Scale	Random-Effect Model	25.11853	25.0149	50.13343	0.9661	1%
	Fixed-Effect Model	24.64237	24.00841	48.65077	0.9478	0%
D. Mini-Mental State Examination	Random-Effect Model	114.8771	100.7861	215.6632	0.9416	0%
	Fixed-Effect Model	160.7242	82.01038	242.7346	1.317	25%
E. Neuropsychiatric Inventory	Random-Effect Model	60.40249	51.13602	111.5385	0.9742	0%
	Fixed-Effect Model	62.60487	48.07208	110.677	1.01	3%
F. Cohen-Mansfeld Agitation Inventory	Random-Effect Model	19.73363	18.98012	38.71375	0.897	0%
	Fixed-Effect Model	20.38407	20.02735	40.41142	0.9265	0%
