# Supplementary material for: Comparative efficacy and acceptability of treatments for depressive symptoms in cognitive impairment: A systematic review and Bayesian network meta-analysis
Source: Front Aging Neurosci. 2022 Dec 12;14:1037414. doi: 10.3389/fnagi.2022.1037414 (PMC9790988; doi:10.3389/fnagi.2022.1037414)
Supplement: Supplementary material 1 — PRISMA NMA checklist. [file Data_Sheet_1.zip › Supplementary Material Presentation/Supplementary_6.pdf]

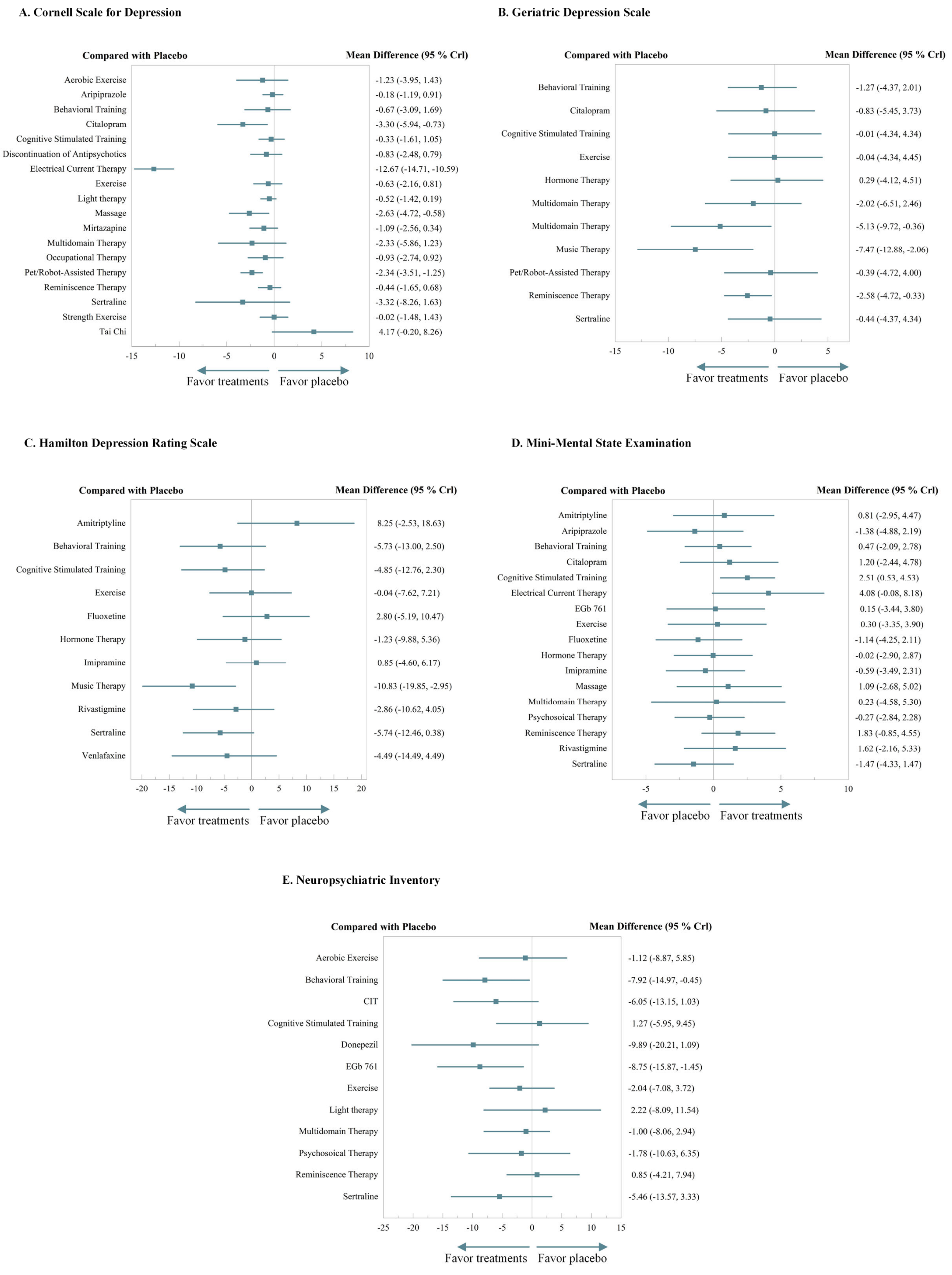
A. Cornell Scale for Depression
Compared with Placebo
Mean Difference (95 % CrI)
Aerobic Exercise -1.23 (-3.95, 1.43)
Aripiprazole -0.18 (-1.19, 0.91)
Behavioral Training -0.67 (-3.09, 1.69)
Citalopram -3.30 (-5.94, -0.73)
Cognitive Stimulated Training -0.33 (-1.61, 1.05)
Discontinuation of Antipsychotics -0.83 (-2.48, 0.79)
Electrical Current Therapy -12.67 (-14.71, -10.59)
Exercise -0.63 (-2.16, 0.81)
Light therapy -0.52 (-1.42, 0.19)
Massage -2.63 (-4.72, -0.58)
Mirtazapine -1.09 (-2.56, 0.34)
Multidomain Therapy -2.33 (-5.86, 1.23)
Occupational Therapy -0.93 (-2.74, 0.92)
Pet/Robot-Assisted Therapy -2.34 (-3.51, -1.25)
Reminiscence Therapy -0.44 (-1.65, 0.68)
Sertraline -3.32 (-8.26, 1.63)
Strength Exercise -0.02 (-1.48, 1.43)
Tai Chi 4.17 (-0.20, 8.26)
-15 -10 -5 0 5 10
Favor treatments
Favor placebo
B. Geriatric Depression Scale
Compared with Placebo
Mean Difference (95 % CrI)
Behavioral Training -1.27 (-4.37, 2.01)
Citalopram -0.83 (-5.45, 3.73)
Cognitive Stimulated Training -0.01 (-4.34, 4.34)
Exercise -0.04 (-4.34, 4.45)
Hormone Therapy 0.29 (-4.12, 4.51)
Multidomain Therapy -2.02 (-6.51, 2.46)
Multidomain Therapy -5.13 (-9.72, -0.36)
Music Therapy -7.47 (-12.88, -2.06)
Pet/Robot-Assisted Therapy -0.39 (-4.72, 4.00)
Reminiscence Therapy -2.58 (-4.72, -0.33)
Sertraline -0.44 (-4.37, 4.34)
-15 -10 -5 0 5
Favor treatments
Favor placebo
C. Hamilton Depression Rating Scale
Compared with Placebo
Mean Difference (95 % CrI)
Amitriptyline 8.25 (-2.53, 18.63)
Behavioral Training -5.73 (-13.00, 2.50)
Cognitive Stimulated Training -4.85 (-12.76, 2.30)
Exercise -0.04 (-7.62, 7.21)
Fluoxetine 2.80 (-5.19, 10.47)
Hormone Therapy -1.23 (-9.88, 5.36)
Imipramine 0.85 (-4.60, 6.17)
Music Therapy -10.83 (-19.85, -2.95)
Rivastigmine -2.86 (-10.62, 4.05)
Sertraline -5.74 (-12.46, 0.38)
Venlafaxine -4.49 (-14.49, 4.49)
-20 -15 -10 -5 0 5 10 15 20
Favor treatments
Favor placebo
D. Mini-Mental State Examination
Compared with Placebo
Mean Difference (95 % CrI)
Amitriptyline 0.81 (-2.95, 4.47)
Aripiprazole -1.38 (-4.88, 2.19)
Behavioral Training 0.47 (-2.09, 2.78)
Citalopram 1.20 (-2.44, 4.78)
Cognitive Stimulated Training 2.51 (0.53, 4.53)
Electrical Current Therapy 4.08 (-0.08, 8.18)
EGb 761 0.15 (-3.44, 3.80)
Exercise 0.30 (-3.35, 3.90)
Fluoxetine -1.14 (-4.25, 2.11)
Hormone Therapy -0.02 (-2.90, 2.87)
Imipramine -0.59 (-3.49, 2.31)
Massage 1.09 (-2.68, 5.02)
Multidomain Therapy 0.23 (-4.58, 5.30)
Psychosoical Therapy -0.27 (-2.84, 2.28)
Reminiscence Therapy 1.83 (-0.85, 4.55)
Rivastigmine 1.62 (-2.16, 5.33)
Sertraline -1.47 (-4.33, 1.47)
-5 0 5 10
Favor placebo
Favor treatments
E. Neuropsychiatric Inventory
Compared with Placebo
Mean Difference (95 % CrI)
Aerobic Exercise -1.12 (-8.87, 5.85)
Behavioral Training -7.92 (-14.97, -0.45)
CIT -6.05 (-13.15, 1.03)
Cognitive Stimulated Training 1.27 (-5.95, 9.45)
Donepezil -9.89 (-20.21, 1.09)
EGb 761 -8.75 (-15.87, -1.45)
Exercise -2.04 (-7.08, 3.72)
Light therapy 2.22 (-8.09, 11.54)
Multidomain Therapy -1.00 (-8.06, 2.94)
Psychosoical Therapy -1.78 (-10.63, 6.35)
Reminiscence Therapy 0.85 (-4.21, 7.94)
Sertraline -5.46 (-13.57, 3.33)
-25 -20 -15 -10 -5 0 5 10 15
Favor treatments
Favor placebo
