# Supplementary material for: Comparative efficacy and acceptability of treatments for depressive symptoms in cognitive impairment: A systematic review and Bayesian network meta-analysis
Source: Front Aging Neurosci. 2022 Dec 12;14:1037414. doi: 10.3389/fnagi.2022.1037414 (PMC9790988; doi:10.3389/fnagi.2022.1037414)
Supplement: Supplementary material 1 — PRISMA NMA checklist. [file Data_Sheet_1.zip › Supplementary Material Presentation/Supplementary_7.pdf]

A. Cornell Scale for Depression

Aerobic Exercise	3.36 (-1.62, 7.71)	3.84 (-1.03, 7.89)	3.98 (-1.20, 8.30)	4.88 (0.06, 9.10)	4.35 (-0.72, 8.66)	1.95 (-3.31, 6.45)	4.73 (-0.13, 9.03)	4.30 (-0.82, 8.49)	-7.43 (-12.57, -2.89)	4.45 (-0.42, 8.58)	4.50 (-0.45, 8.32)	4.50 (-1.86, 9.90)	5.11 (0.40, 9.17)	4.68 (-0.13, 8.72)	2.91 (-1.90, 7.45)	4.69 (-0.25, 8.74)	1.17 (-1.31, 3.80)	5.11 (0.17, 9.20)
-3.36 (-7.71, 1.62)	Aripiprazole	0.48 (-1.62, 2.40)	0.67 (-2.46, 3.71)	1.54 (-0.31, 3.64)	0.98 (-1.70, 4.18)	-1.43 (-4.68, 1.72)	1.39 (-0.64, 3.70)	0.95 (-1.42, 3.32)	-10.80 (-13.49, -8.13)	1.06 (-1.08, 3.34)	1.10 (-1.13, 3.41)	0.94 (-3.12, 5.72)	1.73 (0.11, 3.58)	1.27 (-0.79, 3.47)	-0.40 (-2.54, 1.91)	1.30 (-0.77, 3.43)	-1.98 (-6.59, 3.36)	1.71 (-0.41, 4.00)
-3.84 (-7.89, 1.03)	-0.48 (-2.40, 1.62)	Behavioral Training	0.20 (-2.46, 2.91)	1.07 (-0.18, 2.63)	0.51 (-1.75, 3.24)	-1.89 (-4.73, 0.75)	0.95 (-0.50, 2.67)	0.50 (-1.29, 2.32)	-11.32 (-13.39, -9.06)	0.55 (-0.92, 2.33)	0.64 (-1.06, 2.50)	0.49 (-3.44, 4.81)	1.24 (0.55, 2.34)	0.75 (-0.54, 2.50)	-0.87 (-2.29, 0.90)	0.80 (-0.44, 2.30)	-2.50 (-6.86, 2.77)	1.21 (-0.23, 3.15)
-3.98 (-8.30, 1.20)	-0.67 (-3.71, 2.46)	-0.20 (-2.91, 2.46)	Citalopram	0.90 (-1.89, 3.72)	0.33 (-3.04, 3.97)	-2.11 (-5.67, 1.64)	0.76 (-2.07, 3.75)	0.28 (-2.66, 3.30)	-11.47 (-14.67, -8.22)	0.49 (-2.47, 3.40)	0.32 (-2.42, 3.50)	0.31 (-4.17, 5.34)	1.08 (-1.39, 3.72)	0.64 (-2.19, 3.60)	-1.05 (-3.86, 1.92)	0.67 (-2.18, 3.40)	-2.70 (-7.66, 2.96)	1.04 (-1.71, 4.07)
-4.88 (-9.10, -0.06)	-1.54 (-3.64, 0.31)	-1.07 (-2.63, 0.18)	-0.90 (-3.72, 1.89)	Cognitive Stimulated Training	-0.50 (-3.12, 2.11)	-3.02 (-5.79, -0.14)	-0.12 (-1.34, 0.96)	-0.62 (-2.58, 1.24)	-12.38 (-14.72, -9.95)	-0.49 (-2.19, 1.24)	-0.49 (-2.25, 1.49)	-0.68 (-4.51, 3.68)	0.20 (-0.84, 1.16)	-0.32 (-1.88, 1.27)	-1.92 (-3.62, -0.28)	-0.26 (-1.80, 1.14)	-3.50 (-8.13, 1.51)	0.19 (-1.61, 1.85)
-4.35 (-8.66, 0.72)	-0.98 (-4.18, 1.70)	-0.51 (-3.24, 1.75)	-0.33 (-3.97, 3.04)	0.50 (-2.11, 3.12)	Discontinuation of Antipsychotics	-2.46 (-6.00, 0.91)	0.36 (-2.24, 3.01)	-0.07 (-3.07, 2.70)	-11.91 (-15.11, -8.72)	0.01 (-2.61, 2.66)	0.03 (-2.72, 2.90)	0.04 (-4.44, 4.68)	0.72 (-1.70, 2.96)	0.22 (-2.43, 2.82)	-1.40 (-4.20, 1.15)	0.27 (-2.39, 2.76)	-3.10 (-8.05, 2.52)	0.71 (-2.00, 3.35)
-1.95 (-6.45, 3.31)	1.43 (-1.72, 4.68)	1.89 (-0.75, 4.73)	2.11 (-1.64, 5.67)	3.02 (0.14, 5.79)	2.46 (-0.91, 6.00)	Electrical Current Therapy	2.87 (-0.09, 5.72)	2.36 (-0.89, 5.53)	-9.49 (-12.43, -5.78)	2.49 (-0.36, 5.62)	2.56 (-0.50, 5.99)	2.38 (-3.02, 7.28)	3.14 (0.61, 5.95)	2.60 (0.03, 5.72)	1.04 (-2.07, 4.20)	2.71 (-0.02, 5.58)	-0.76 (-5.43, 5.05)	3.10 (0.31, 6.27)
-4.73 (-9.03, 0.13)	-1.39 (-3.70, 0.64)	-0.95 (-2.67, 0.50)	-0.76 (-3.75, 2.07)	0.12 (-0.96, 1.34)	-0.36 (-3.01, 2.24)	-2.87 (-5.72, 0.09)	Exercise	-0.51 (-2.55, 1.54)	-12.25 (-14.65, -9.70)	-0.37 (-2.19, 1.54)	-0.36 (-2.17, 1.70)	-0.55 (-4.31, 3.86)	0.32 (-0.95, 1.55)	-0.20 (-1.92, 1.58)	-1.80 (-3.63, 0.01)	-0.13 (-1.83, 1.46)	-3.36 (-8.00, 1.74)	0.30 (-1.60, 2.14)
-4.30 (-8.49, 0.82)	-0.95 (-3.32, 1.42)	-0.50 (-2.32, 1.29)	-0.28 (-3.30, 2.66)	0.62 (-1.24, 2.58)	0.07 (-2.70, 3.07)	-2.36 (-5.53, 0.89)	0.51 (-1.54, 2.55)	Light therapy	-11.78 (-14.31, -9.07)	0.10 (-1.93, 2.29)	0.20 (-2.03, 2.41)	-0.06 (-4.03, 4.58)	0.75 (-0.77, 2.48)	0.25 (-1.61, 2.41)	-1.31 (-3.32, 0.87)	0.35 (-1.55, 2.28)	-3.01 (-7.48, 2.64)	0.74 (-1.26, 2.95)
7.43 (2.89, 12.57)	10.80 (8.13, 13.49)	11.32 (9.06, 13.39)	11.47 (8.22, 14.67)	12.38 (9.95, 14.72)	11.91 (8.72, 15.11)	9.49 (5.78, 12.43)	12.25 (9.70, 14.65)	11.78 (9.07, 14.31)	Massage	11.90 (9.53, 14.32)	11.95 (9.48, 14.43)	11.84 (7.34, 16.46)	12.58 (10.61, 14.64)	12.10 (9.85, 14.40)	10.46 (8.13, 12.88)	12.10 (9.90, 14.41)	8.74 (4.14, 14.31)	12.57 (10.15, 15.04)
-4.45 (-8.58, 0.42)	-1.06 (-3.34, 1.08)	-0.55 (-2.33, 0.92)	-0.49 (-3.40, 2.47)	0.49 (-1.24, 2.19)	-0.01 (-2.66, 2.61)	-2.49 (-5.62, 0.36)	0.37 (-1.54, 2.19)	-0.10 (-2.29, 1.93)	-11.90 (-14.32, -9.53)	Mirtazapine	0.01 (-2.14, 2.11)	-0.08 (-4.11, 4.17)	0.68 (-0.75, 1.99)	0.18 (-1.66, 2.03)	-1.42 (-3.36, 0.39)	0.21 (-1.18, 1.48)	-3.13 (-7.61, 2.16)	0.65 (-1.29, 2.63)
-4.50 (-8.32, 0.45)	-1.10 (-3.41, 1.13)	-0.64 (-2.50, 1.06)	-0.32 (-3.50, 2.42)	0.49 (-1.49, 2.25)	-0.03 (-2.90, 2.72)	-2.56 (-5.99, 0.50)	0.36 (-1.70, 2.17)	-0.20 (-2.41, 2.03)	-11.95 (-14.43, -9.48)	-0.01 (-2.11, 2.14)	Multidomain Therapy	-0.19 (-4.17, 4.25)	0.66 (-0.89, 2.16)	0.16 (-1.72, 2.19)	-1.50 (-3.53, 0.52)	0.21 (-1.77, 2.01)	-3.24 (-7.83, 2.17)	0.64 (-1.46, 2.69)
-4.50 (-9.90, 1.86)	-0.94 (-5.72, 3.12)	-0.49 (-4.81, 3.44)	-0.31 (-5.34, 4.17)	0.68 (-3.68, 4.51)	-0.04 (-4.68, 4.44)	-2.38 (-7.28, 3.02)	0.55 (-3.86, 4.31)	0.06 (-4.58, 4.03)	-11.84 (-16.46, -7.34)	0.08 (-4.17, 4.11)	0.19 (-4.25, 4.17)	Occupational Therapy	0.75 (-3.35, 4.60)	0.26 (-4.05, 4.45)	-1.33 (-5.62, 2.75)	0.31 (-3.95, 4.24)	-3.25 (-9.08, 3.55)	0.74 (-3.66, 4.82)
-5.11 (-9.17, -0.40)	-1.73 (-3.58, -0.11)	-1.24 (-2.34, -0.55)	-1.08 (-3.72, 1.39)	-0.20 (-1.16, 0.84)	-0.72 (-2.96, 1.70)	-3.14 (-5.95, -0.61)	-0.32 (-1.55, 0.95)	-0.75 (-2.48, 0.77)	-12.58 (-14.64, -10.61)	-0.68 (-1.99, 0.75)	-0.66 (-2.16, 0.89)	-0.75 (-4.60, 3.35)	Placebo	-0.51 (-1.68, 0.78)	-2.11 (-3.46, -0.80)	-0.44 (-1.56, 0.61)	-3.75 (-8.06, 1.30)	-0.02 (-1.46, 1.43)
-4.68 (-8.72, 0.13)	-1.27 (-3.47, 0.79)	-0.75 (-2.50, 0.54)	-0.64 (-3.60, 2.19)	0.32 (-1.27, 1.88)	-0.22 (-2.82, 2.43)	-2.60 (-5.72, -0.03)	0.20 (-1.58, 1.92)	-0.25 (-2.41, 1.61)	-12.10 (-14.40, -9.85)	-0.18 (-2.03, 1.66)	-0.16 (-2.19, 1.72)	-0.26 (-4.45, 4.05)	0.51 (-0.78, 1.68)	Pet/Robot-Assisted Therapy	-1.60 (-3.45, 0.08)	0.09 (-1.64, 1.59)	-3.33 (-7.73, 1.94)	0.50 (-1.43, 2.28)
-2.91 (-7.45, 1.90)	0.40 (-1.91, 2.54)	0.87 (-0.90, 2.29)	1.05 (-1.92, 3.86)	1.92 (0.28, 3.62)	1.40 (-1.15, 4.20)	-1.04 (-4.20, 2.07)	1.80 (-0.01, 3.63)	1.31 (-0.87, 3.32)	-10.46 (-12.88, -8.13)	1.42 (-0.39, 3.36)	1.50 (-0.52, 3.53)	1.33 (-2.75, 5.62)	2.11 (0.80, 3.46)	1.60 (-0.08, 3.45)	Reminiscence Therapy	1.65 (-0.01, 3.28)	-1.67 (-6.16, 3.65)	2.08 (0.21, 4.07)
-4.69 (-8.74, 0.25)	-1.30 (-3.43, 0.77)	-0.80 (-2.30, 0.44)	-0.67 (-3.40, 2.18)	0.26 (-1.14, 1.80)	-0.27 (-2.76, 2.39)	-2.71 (-5.58, 0.02)	0.13 (-1.46, 1.83)	-0.35 (-2.28, 1.55)	-12.10 (-14.41, -9.90)	-0.21 (-1.48, 1.18)	-0.21 (-2.01, 1.77)	-0.31 (-4.24, 3.95)	0.44 (-0.61, 1.56)	-0.09 (-1.59, 1.64)	-1.65 (-3.28, 0.01)	Sertraline	-3.35 (-7.73, 1.97)	0.41 (-1.30, 2.30)
-1.17 (-3.80, 1.31)	1.98 (-3.36, 6.59)	2.50 (-2.77, 6.86)	2.70 (-2.96, 7.66)	3.50 (-1.51, 8.13)	3.10 (-2.52, 8.05)	0.76 (-5.05, 5.43)	3.36 (-1.74, 8.00)	3.01 (-2.64, 7.48)	-8.74 (-14.31, -4.14)	3.13 (-2.16, 7.61)	3.24 (-2.17, 7.83)	3.25 (-3.55, 9.08)	3.75 (-1.30, 8.06)	3.33 (-1.94, 7.73)	1.67 (-3.65, 6.16)	3.35 (-1.97, 7.73)	Strength Exercise	3.76 (-1.61, 8.23)
-5.11 (-9.20, -0.17)	-1.71 (-4.00, 0.41)	-1.21 (-3.15, 0.23)	-1.04 (-4.07, 1.71)	-0.19 (-1.85, 1.61)	-0.71 (-3.35, 2.00)	-3.10 (-6.27, -0.31)	-0.30 (-2.14, 1.60)	-0.74 (-2.95, 1.26)	-12.57 (-15.04, -10.15)	-0.65 (-2.63, 1.29)	-0.64 (-2.69, 1.46)	-0.74 (-4.82, 3.66)	0.02 (-1.43, 1.46)	-0.50 (-2.28, 1.43)	-2.08 (-4.07, -0.21)	-0.41 (-2.30, 1.30)	-3.76 (-8.23, 1.61)	Tai Chi

B. Geriatric Depression Scale

Behavioral Training	0.42 (-5.16, 5.99)	1.29 (-4.14, 6.72)	1.23 (-4.21, 6.57)	1.54 (-3.91, 6.77)	-0.75 (-6.31, 4.74)	-3.87 (-9.46, 1.87)	-6.19 (-12.49, -0.10)	1.27 (-2.01, 4.37)	0.86 (-4.49, 6.29)	-1.35 (-5.17, 2.45)	1.23 (-4.27, 6.62)
-0.42 (-5.99, 5.16)	Citalopram	0.84 (-5.38, 7.08)	0.80 (-5.54, 7.21)	1.13 (-5.11, 7.38)	-1.17 (-7.57, 5.21)	-4.25 (-10.78, 2.39)	-6.58 (-13.87, 0.40)	0.83 (-3.73, 5.45)	0.45 (-5.75, 6.85)	-1.74 (-6.78, 3.42)	0.80 (-5.55, 7.19)
-1.29 (-6.72, 4.14)	-0.84 (-7.08, 5.38)	Cognitive Stimulated Training	-0.06 (-6.10, 6.14)	0.28 (-6.04, 6.33)	-2.04 (-8.32, 4.33)	-5.15 (-11.44, 1.31)	-7.50 (-14.45, -0.72)	0.01 (-4.34, 4.34)	-0.42 (-6.58, 5.80)	-2.61 (-7.50, 2.30)	-0.06 (-6.36, 6.33)
-1.23 (-6.57, 4.21)	-0.80 (-7.21, 5.54)	0.06 (-6.14, 6.10)	Exercise	0.31 (-5.95, 6.25)	-1.97 (-8.27, 4.21)	-5.10 (-11.42, 1.29)	-7.44 (-14.40, -0.54)	0.04 (-4.45, 4.34)	-0.34 (-6.49, 5.70)	-2.58 (-7.45, 2.34)	-0.02 (-6.10, 6.27)
-1.54 (-6.77, 3.91)	-1.13 (-7.38, 5.11)	-0.28 (-6.33, 6.04)	-0.31 (-6.25, 5.95)	Hormone Therapy	-2.32 (-8.43, 4.07)	-5.39 (-11.66, 0.96)	-7.73 (-14.60, -0.90)	-0.29 (-4.51, 4.12)	-0.68 (-6.82, 5.51)	-2.88 (-7.57, 2.10)	-0.32 (-6.37, 5.96)
0.75 (-4.74, 6.31)	1.17 (-5.21, 7.57)	2.04 (-4.33, 8.32)	1.97 (-4.21, 8.27)	2.32 (-4.07, 8.43)	Multidomain Therapy	-3.14 (-9.47, 3.49)	-5.44 (-12.40, 1.51)	2.02 (-2.46, 6.51)	1.63 (-4.48, 7.92)	-0.57 (-5.55, 4.53)	1.99 (-4.17, 8.40)
3.87 (-1.87, 9.46)	4.25 (-2.39, 10.78)	5.15 (-1.31, 11.44)	5.10 (-1.29, 11.42)	5.39 (-0.96, 11.66)	3.14 (-3.49, 9.47)	Multidomain Therapy	-2.31 (-9.53, 4.77)	5.13 (0.36, 9.72)	4.74 (-1.62, 11.13)	2.54 (-2.60, 7.64)	5.06 (-1.35, 11.49)
6.19 (0.10, 12.49)	6.58 (-0.40, 13.87)	7.50 (0.72, 14.45)	7.44 (0.54, 14.40)	7.73 (0.90, 14.60)	5.44 (-1.51, 12.40)	2.31 (-4.77, 9.53)	Music Therapy	7.47 (2.06, 12.88)	7.10 (0.13, 14.08)	4.88 (-0.88, 10.85)	7.41 (0.60, 14.53)
-1.27 (-4.37, 2.01)	-0.83 (-5.45, 3.73)	-0.01 (-4.34, 4.34)	-0.04 (-4.34, 4.45)	0.29 (-4.12, 4.51)	-2.02 (-6.51, 2.46)	-5.13 (-9.72, -0.36)	-7.47 (-12.88, -2.06)	Placebo	-0.39 (-4.72, 4.00)	-2.58 (-4.72, -0.33)	-0.04 (-4.37, 4.34)
-0.86 (-6.29, 4.49)	-0.45 (-6.85, 5.75)	0.42 (-5.80, 6.58)	0.34 (-5.70, 6.49)	0.68 (-5.51, 6.82)	-1.63 (-7.92, 4.48)	-4.74 (-11.13, 1.62)	-7.10 (-14.08, -0.13)	0.39 (-4.00, 4.72)	Pet/Robot-Assisted Therapy	-2.22 (-6.98, 2.67)	0.34 (-5.86, 6.63)
1.35 (-2.45, 5.17)	1.74 (-3.42, 6.78)	2.61 (-2.30, 7.50)	2.58 (-2.34, 7.45)	2.88 (-2.10, 7.57)	0.57 (-4.53, 5.55)	-2.54 (-7.64, 2.60)	-4.88 (-10.85, 0.88)	2.58 (0.33, 4.72)	2.22 (-2.67, 6.98)	Reminiscence Therapy	2.55 (-2.38, 7.47)
-1.23 (-6.62, 4.27)	-0.80 (-7.19, 5.55)	0.06 (-6.33, 6.36)	0.02 (-6.27, 6.10)	0.32 (-5.96, 6.37)	-1.99 (-8.40, 4.17)	-5.06 (-11.49, 1.35)	-7.41 (-14.53, -0.60)	0.04 (-4.34, 4.37)	-0.34 (-6.63, 5.86)	-2.55 (-7.47, 2.38)	Sertraline

C. Hamilton Depression Rating Scale

Amitriptyline	-14.30 (-27.25, 0.11)	-13.37 (-26.62, -0.42)	-8.29 (-21.13, 4.30)	-5.62 (-12.59, 1.63)	-9.52 (-24.13, 2.28)	-7.51 (-19.34, 4.60)	-19.42 (-32.17, -5.82)	-8.25 (-18.63, 2.53)	-11.13 (-24.56, 1.19)	-14.28 (-26.48, -2.44)	-13.17 (-27.87, 0.84)
14.30 (-0.11, 27.25)	Behavioral Training	0.84 (-11.28, 10.85)	5.77 (-5.92, 15.73)	8.66 (-3.03, 19.33)	4.44 (-7.71, 13.94)	6.57 (-3.39, 15.38)	-5.07 (-16.64, 5.77)	5.73 (-2.50, 13.00)	2.85 (-8.64, 12.74)	-0.07 (-11.00, 9.41)	1.19 (-12.66, 13.17)
13.37 (0.42, 26.62)	-0.84 (-10.85, 11.28)	Cognitive Stimulated Training	4.83 (-5.38, 15.86)	7.88 (-2.91, 19.11)	3.67 (-7.35, 13.94)	5.69 (-3.14, 15.32)	-5.93 (-16.28, 5.09)	4.85 (-2.30, 12.76)	2.01 (-8.16, 12.67)	-0.86 (-10.51, 9.19)	0.61 (-12.29, 12.54)
8.29 (-4.30, 21.13)	-5.77 (-15.73, 5.92)	-4.83 (-15.86, 5.38)	Exercise	2.78 (-7.70, 13.71)	-1.23 (-12.35, 8.58)	0.86 (-8.30, 10.22)	-10.79 (-21.81, 0.72)	0.04 (-7.21, 7.62)	-2.83 (-13.49, 7.42)	-5.74 (-15.01, 3.52)	-4.39 (-16.77, 7.35)
5.62 (-1.63, 12.59)	-8.66 (-19.33, 3.03)	-7.88 (-19.11, 2.91)	-2.78 (-13.71, 7.70)	Fluoxetine	-4.09 (-16.67, 5.68)	-1.96 (-11.55, 7.36)	-13.86 (-25.30, -2.38)	-2.80 (-10.47, 5.19)	-5.66 (-16.79, 4.65)	-8.77 (-19.06, 1.21)	-7.65 (-20.91, 4.64)
9.52 (-2.28, 24.13)	-4.44 (-13.94, 7.71)	-3.67 (-13.94, 7.35)	1.23 (-8.58, 12.35)	4.09 (-5.68, 16.67)	Hormone Therapy	2.10 (-6.04, 12.30)	-9.57 (-20.21, 2.59)	1.23 (-5.36, 9.88)	-1.63 (-10.99, 9.58)	-4.50 (-14.05, 5.91)	-3.18 (-15.27, 9.36)
7.51 (-4.60, 19.34)	-6.57 (-15.38, 3.39)	-5.69 (-15.32, 3.14)	-0.86 (-10.22, 8.30)	1.96 (-7.36, 11.55)	-2.10 (-12.30, 6.04)	Imipramine	-11.61 (-21.84, -1.83)	-0.85 (-6.17, 4.60)	-3.70 (-12.73, 4.94)	-6.53 (-15.33, 1.53)	-5.26 (-16.84, 5.35)
19.42 (5.82, 32.17)	5.07 (-5.77, 16.64)	5.93 (-5.09, 16.28)	10.79 (-0.72, 21.81)	13.86 (2.38, 25.30)	9.57 (-2.59, 20.21)	11.61 (1.83, 21.84)	Music Therapy	10.83 (2.95, 19.85)	7.91 (-3.39, 18.81)	4.99 (-5.66, 15.31)	6.41 (-7.12, 18.90)
8.25 (-2.53, 18.63)	-5.73 (-13.00, 2.50)	-4.85 (-12.76, 2.30)	-0.04 (-7.62, 7.21)	2.80 (-5.19, 10.47)	-1.23 (-9.88, 5.36)	0.85 (-4.60, 6.17)	-10.83 (-19.85, -2.95)	Placebo	-2.86 (-10.62, 4.05)	-5.74 (-12.46, 0.38)	-4.49 (-14.94, 4.92)
11.13 (-1.19, 24.56)	-2.85 (-12.74, 8.64)	-2.01 (-12.67, 8.16)	2.83 (-7.42, 13.49)	5.66 (-4.65, 16.79)	1.63 (-9.58, 10.99)	3.70 (-4.94, 12.73)	-7.91 (-18.81, 3.39)	2.86 (-4.05, 10.62)	Rivastigmine	-2.90 (-12.90, 6.86)	-1.55 (-14.31, 10.29)
14.28 (2.44, 26.48)	0.07 (-9.41, 11.00)	0.86 (-9.19, 10.51)	5.74 (-3.52, 15.01)	8.77 (-1.21, 19.06)	4.50 (-5.91, 14.05)	6.53 (-1.53, 15.33)	-4.99 (-15.31, 5.66)	5.74 (-0.38, 12.46)	2.90 (-6.86, 12.90)	Sertraline	1.29 (-6.58, 8.90)
13.17 (-0.84, 27.87)	-1.19 (-13.17, 12.66)	-0.61 (-12.54, 12.29)	4.39 (-7.35, 16.77)	7.65 (-4.64, 20.91)	3.18 (-9.36, 15.27)	5.26 (-5.35, 16.84)	-6.41 (-18.90, 7.12)	4.49 (-4.92, 14.94)	1.55 (-10.29, 14.31)	-1.29 (-8.90, 6.58)	Venlafaxine

D. Mini-Mental State Examination

Amitriptyline	-2.13 (-7.22, 2.91)	-0.30 (-4.88, 3.89)	0.42 (-4.79, 5.47)	1.72 (-2.46, 5.95)	3.31 (-2.31, 8.81)	-0.63 (-5.82, 4.48)	-0.50 (-5.60, 4.72)	-1.92 (-6.92, 3.03)	-0.81 (-5.55, 3.86)	-1.39 (-6.03, 3.38)	0.33 (-5.00, 5.76)	-0.80 (-6.27, 4.87)	-0.81 (-4.47, 2.95)	-1.06 (-5.55, 3.47)	1.05 (-3.54, 5.62)	0.82 (-4.49, 6.11)	-2.23 (-6.95, 2.50)
2.13 (-2.91, 7.22)	Aripiprazole	1.86 (-2.55, 5.97)	2.58 (-2.56, 7.74)	3.91 (-0.16, 7.90)	5.44 (-0.01, 10.73)	1.52 (-3.48, 6.45)	1.67 (-3.43, 6.74)	0.22 (-4.53, 4.99)	1.35 (-3.24, 5.90)	0.76 (-3.72, 5.32)	2.45 (-2.90, 7.85)	1.36 (-4.16, 6.76)	1.38 (-2.19, 4.88)	1.09 (-3.36, 5.53)	3.19 (-1.26, 7.67)	3.00 (-2.32, 8.02)	-0.10 (-4.71, 4.50)
0.30 (-3.89, 4.88)	-1.86 (-5.97, 2.55)	Behavioral Training	0.72 (-3.39, 5.26)	2.02 (-1.00, 5.34)	3.62 (-1.08, 8.56)	-0.32 (-4.56, 4.18)	-0.20 (-4.32, 4.29)	-1.61 (-5.40, 2.50)	-0.51 (-4.15, 3.44)	-1.04 (-4.81, 2.83)	0.63 (-3.73, 5.34)	-0.51 (-5.13, 4.48)	-0.47 (-2.78, 2.09)	-0.73 (-4.06, 2.92)	1.34 (-2.15, 5.13)	1.16 (-3.25, 5.69)	-1.93 (-5.56, 1.91)
-0.42 (-5.47, 4.79)	-2.58 (-7.74, 2.56)	-0.72 (-5.26, 3.39)	Citalopram	1.31 (-2.82, 5.40)	2.88 (-2.53, 8.30)	-1.02 (-6.08, 3.95)	-0.95 (-5.89, 4.26)	-2.34 (-7.09, 2.54)	-1.25 (-5.93, 3.38)	-1.84 (-6.44, 2.91)	-0.09 (-5.39, 5.10)	-1.23 (-6.74, 4.51)	-1.20 (-4.78, 2.44)	-1.48 (-5.95, 2.95)	0.63 (-3.90, 5.15)	0.43 (-4.79, 5.62)	-2.67 (-7.23, 1.93)
-1.72 (-5.95, 2.46)	-3.91 (-7.90, 0.16)	-2.02 (-5.34, 1.00)	-1.31 (-5.40, 2.82)	Cognitive Stimulated Training	1.56 (-3.09, 6.19)	-2.36 (-6.45, 1.73)	-2.21 (-6.29, 1.95)	-3.63 (-7.38, 0.21)	-2.54 (-6.09, 0.97)	-3.10 (-6.60, 0.45)	-1.40 (-5.71, 2.97)	-2.53 (-7.18, 2.19)	-2.51 (-4.53, -0.53)	-2.78 (-6.07, 0.60)	-0.68 (-4.03, 2.63)	-0.89 (-5.20, 3.32)	-3.99 (-7.47, -0.45)
-3.31 (-8.81, 2.31)	-5.44 (-10.73, 0.01)	-3.62 (-8.56, 1.08)	-2.88 (-8.30, 2.53)	-1.56 (-6.19, 3.09)	Electrical Current Therapy	-3.91 (-9.29, 1.43)	-3.78 (-9.15, 1.69)	-5.23 (-10.36, 0.09)	-4.12 (-9.29, 1.02)	-4.62 (-9.79, 0.44)	-2.97 (-8.63, 2.73)	-4.06 (-9.95, 1.73)	-4.08 (-8.18, 0.08)	-4.36 (-9.15, 0.55)	-2.25 (-7.13, 2.71)	-2.46 (-8.09, 3.04)	-5.52 (-10.55, -0.58)
0.63 (-4.48, 5.82)	-1.52 (-6.45, 3.48)	0.32 (-4.18, 4.56)	1.02 (-3.95, 6.08)	2.36 (-1.73, 6.45)	3.91 (-1.43, 9.29)	EGb 761	0.15 (-4.96, 5.19)	-1.28 (-5.87, 3.64)	-0.16 (-4.80, 4.39)	-0.75 (-5.31, 3.96)	0.95 (-4.35, 6.21)	-0.18 (-5.71, 5.41)	-0.15 (-3.80, 3.44)	-0.41 (-4.81, 4.04)	1.69 (-2.90, 6.27)	1.45 (-3.86, 6.73)	-1.63 (-6.08, 3.05)
0.50 (-4.72, 5.60)	-1.67 (-6.74, 3.43)	0.20 (-4.29, 4.32)	0.95 (-4.26, 5.89)	2.21 (-1.95, 6.29)	3.78 (-1.69, 9.15)	-0.15 (-5.19, 4.96)	Exercise	-1.43 (-6.16, 3.41)	-0.32 (-5.01, 4.20)	-0.92 (-5.52, 3.72)	0.81 (-4.48, 6.03)	-0.30 (-5.92, 5.32)	-0.30 (-3.90, 3.35)	-0.55 (-5.02, 3.93)	1.57 (-2.98, 5.98)	1.29 (-4.08, 6.57)	-1.75 (-6.38, 2.94)
1.92 (-3.03, 6.92)	-0.22 (-4.99, 4.53)	1.61 (-2.50, 5.40)	2.34 (-2.54, 7.09)	3.63 (-0.21, 7.38)	5.23 (-0.09, 10.36)	1.28 (-3.64, 5.87)	1.43 (-3.41, 6.16)	Fluoxetine	1.11 (-3.21, 5.32)	0.54 (-3.74, 4.75)	2.25 (-2.74, 7.11)	1.09 (-4.26, 6.48)	1.14 (-2.11, 4.25)	0.86 (-3.28, 4.88)	2.96 (-1.27, 7.11)	2.74 (-2.21, 7.48)	-0.33 (-4.64, 3.98)
0.81 (-3.86, 5.55)	-1.35 (-5.90, 3.24)	0.51 (-3.44, 4.15)	1.25 (-3.38, 5.93)	2.54 (-0.97, 6.09)	4.12 (-1.02, 9.29)	0.16 (-4.39, 4.80)	0.32 (-4.20, 5.01)	-1.11 (-5.32, 3.21)	Hormone Therapy	-0.56 (-4.66, 3.53)	1.14 (-3.67, 6.06)	-0.01 (-4.98, 5.10)	0.02 (-2.87, 2.90)	-0.22 (-4.24, 3.61)	1.88 (-2.03, 5.78)	1.65 (-3.07, 6.31)	-1.44 (-5.60, 2.76)
1.39 (-3.38, 6.03)	-0.76 (-5.32, 3.72)	1.04 (-2.83, 4.81)	1.84 (-2.91, 6.44)	3.10 (-0.45, 6.60)	4.62 (-0.44, 9.79)	0.75 (-3.96, 5.31)	0.92 (-3.72, 5.52)	-0.54 (-4.75, 3.74)	0.56 (-3.53, 4.66)	Imipramine	1.70 (-3.05, 6.64)	0.58 (-4.44, 5.67)	0.59 (-2.31, 3.49)	0.34 (-3.65, 4.21)	2.43 (-1.50, 6.33)	2.21 (-2.70, 6.89)	-0.87 (-4.94, 3.26)
-0.33 (-5.76, 5.00)	-2.45 (-7.85, 2.90)	-0.63 (-5.34, 3.73)	0.09 (-5.10, 5.39)	1.40 (-2.97, 5.71)	2.97 (-2.73, 8.63)	-0.95 (-6.21, 4.35)	-0.81 (-6.03, 4.48)	-2.25 (-7.11, 2.74)	-1.14 (-6.06, 3.67)	-1.70 (-6.64, 3.05)	Massage	-1.12 (-6.84, 4.65)	-1.09 (-5.02, 2.68)	-1.39 (-6.00, 3.32)	0.74 (-3.84, 5.40)	0.52 (-4.96, 5.87)	-2.55 (-7.36, 2.19)
0.80 (-4.87, 6.27)	-1.36 (-6.76, 4.16)	0.51 (-4.48, 5.13)	1.23 (-4.51, 6.74)	2.53 (-2.19, 7.18)	4.06 (-1.73, 9.95)	0.18 (-5.41, 5.71)	0.30 (-5.32, 5.92)	-1.09 (-6.48, 4.26)	0.01 (-5.10, 4.98)	-0.58 (-5.67, 4.44)	1.12 (-4.65, 6.84)	Multidomain Therapy	0.02 (-4.28, 4.18)	-0.23 (-5.30, 4.58)	1.84 (-3.20, 6.77)	1.64 (-4.08, 7.25)	-1.45 (-6.58, 3.67)
0.81 (-2.95, 4.47)	-1.38 (-4.88, 2.19)	0.47 (-2.09, 2.78)	1.20 (-2.44, 4.78)	2.51 (0.53, 4.53)	4.08 (-0.08, 8.18)	0.15 (-3.44, 3.80)	0.30 (-3.35, 3.90)	-1.14 (-4.25, 2.11)	-0.02 (-2.90, 2.87)	-0.59 (-3.49, 2.31)	1.09 (-2.68, 5.02)	-0.02 (-4.18, 4.28)	Placebo	-0.27 (-2.84, 2.28)	1.83 (-0.85, 4.55)	1.62 (-2.16, 5.33)	-1.47 (-4.33, 1.47)
1.06 (-3.47, 5.55)	-1.09 (-5.53, 3.36)	0.73 (-2.92, 4.06)	1.48 (-2.95, 5.95)	2.78 (-0.60, 6.07)	4.36 (-0.55, 9.15)	0.41 (-4.04, 4.81)	0.55 (-3.93, 5.02)	-0.86 (-4.88, 3.28)	0.22 (-3.61, 4.24)	-0.34 (-4.21, 3.65)	1.39 (-3.32, 6.00)	0.23 (-4.58, 5.30)	0.27 (-2.28, 2.84)	Psychosoical Therapy	2.12 (-1.65, 5.76)	1.91 (-2.74, 6.43)	-1.21 (-5.00, 2.68)
-1.05 (-5.62, 3.54)	-3.19 (-7.67, 1.26)	-1.34 (-5.13, 2.15)	-0.63 (-5.15, 3.90)	0.68 (-2.63, 4.03)	2.25 (-2.71, 7.13)	-1.69 (-6.27, 2.90)	-1.57 (-5.98, 2.98)	-2.96 (-7.11, 1.27)	-1.88 (-5.78, 2.03)	-2.43 (-6.33, 1.50)	-0.74 (-5.40, 3.84)	-1.84 (-6.77, 3.20)	-1.83 (-4.55, 0.85)	-2.12 (-5.76, 1.65)	Reminiscence Therapy	-0.24 (-4.89, 4.41)	-3.29 (-7.23, 0.68)
-0.82 (-6.11, 4.49)	-3.00 (-8.02, 2.32)	-1.16 (-5.69, 3.25)	-0.43 (-5.62, 4.79)	0.89 (-3.32, 5.20)	2.46 (-3.04, 8.09)	-1.45 (-6.73, 3.86)	-1.29 (-6.57, 4.08)	-2.74 (-7.48, 2.21)	-1.65 (-6.31, 3.07)	-2.21 (-6.89, 2.70)	-0.52 (-5.87, 4.96)	-1.64 (-7.25, 4.08)	-1.62 (-5.33, 2.16)	-1.91 (-6.43, 2.74)	0.24 (-4.41, 4.89)	Rivastigmine	-3.09 (-7.77, 1.74)
2.23 (-2.50, 6.95)	0.10 (-4.50, 4.71)	1.93 (-1.91, 5.56)	2.67 (-1.93, 7.23)	3.99 (0.45, 7.47)	5.52 (0.58, 10.55)	1.63 (-3.05, 6.08)	1.75 (-2.94, 6.38)	0.33 (-3.98, 4.64)	1.44 (-2.76, 5.60)	0.87 (-3.26, 4.94)	2.55 (-2.19, 7.36)	1.45 (-3.67, 6.58)	1.47 (-1.47, 4.33)	1.21 (-2.68, 5.00)	3.29 (-0.68, 7.23)	3.09 (-1.74, 7.77)	Sertraline

E. Neuropsychiatric Inventory

Aerobic Exercise	-6.65 (-16.71, 4.36)	-4.92 (-14.92, 5.55)	2.52 (-7.30, 13.81)	-8.73 (-21.39, 4.65)	-7.51 (-17.68, 3.02)	-0.56 (-9.32, 9.02)	3.65 (-8.92, 14.74)	0.18 (-10.48, 7.93)	1.12 (-5.85, 8.87)	-0.49 (-11.88, 10.59)	1.94 (-5.84, 12.97)	-4.07 (-15.04, 7.58)
6.65 (-4.36, 16.71)	Behavioral Training	1.94 (-8.50, 11.86)	9.63 (-0.91, 19.89)	-1.48 (-15.24, 10.93)	-0.59 (-11.10, 9.42)	6.34 (-3.12, 14.94)	10.11 (-2.55, 21.11)	6.78 (-3.93, 14.21)	7.92 (0.45, 14.97)	5.79 (-5.31, 16.69)	8.83 (0.52, 18.99)	2.17 (-8.33, 13.85)
4.92 (-5.55, 14.92)	-1.94 (-11.86, 8.50)	CIT	7.25 (-2.13, 18.40)	-3.79 (-16.32, 9.32)	-2.66 (-12.45, 7.29)	4.04 (-4.63, 13.51)	8.44 (-4.21, 19.31)	5.12 (-5.11, 12.30)	6.05 (-1.03, 13.15)	4.31 (-6.98, 14.65)	6.76 (-0.91, 17.33)	0.56 (-10.06, 11.59)
-2.52 (-13.81, 7.30)	-9.63 (-19.89, 0.91)	-7.25 (-18.40, 2.13)	Cognitive Stimulated Training	-10.96 (-24.66, 1.85)	-9.83 (-21.03, -0.00)	-3.05 (-12.77, 5.92)	0.56 (-12.79, 12.68)	-2.39 (-13.86, 5.42)	-1.27 (-9.45, 5.95)	-3.25 (-15.57, 7.34)	-0.71 (-8.08, 8.08)	-7.06 (-18.48, 4.72)
8.73 (-4.65, 21.39)	1.48 (-10.93, 15.24)	3.79 (-9.32, 16.32)	10.96 (-1.85, 24.66)	Donepezil	1.14 (-6.65, 8.75)	7.83 (-3.35, 19.53)	12.31 (-3.19, 25.32)	8.69 (-4.57, 19.06)	9.89 (-1.09, 20.21)	8.34 (-6.01, 21.23)	10.09 (-0.26, 23.65)	3.76 (-9.12, 18.18)
7.51 (-3.02, 17.68)	0.59 (-9.42, 11.10)	2.66 (-7.29, 12.45)	9.83 (0.00, 21.03)	-1.14 (-8.75, 6.65)	EGb 761	6.72 (-1.90, 15.88)	11.05 (-1.80, 21.90)	7.65 (-2.90, 15.22)	8.75 (1.45, 15.87)	6.88 (-4.69, 17.52)	9.24 (1.20, 20.10)	3.16 (-7.72, 14.63)
0.56 (-9.02, 9.32)	-6.34 (-14.94, 3.12)	-4.04 (-13.51, 4.63)	3.05 (-5.92, 12.77)	-7.83 (-19.53, 3.35)	-6.72 (-15.88, 1.90)	Exercise	4.09 (-7.76, 14.23)	0.73 (-8.50, 6.75)	2.04 (-3.72, 7.08)	-0.03 (-10.33, 9.71)	2.38 (-4.32, 11.78)	-3.72 (-13.34, 6.64)
-3.65 (-14.74, 8.92)	-10.11 (-21.11, 2.55)	-8.44 (-19.31, 4.21)	-0.56 (-12.68, 12.79)	-12.31 (-25.32, 3.19)	-11.05 (-21.90, 1.80)	-4.09 (-14.23, 7.76)	Light therapy	-3.62 (-14.87, 7.22)	-2.22 (-11.54, 8.09)	-4.06 (-16.57, 9.06)	-0.99 (-12.13, 11.51)	-7.54 (-19.74, 6.00)
-0.18 (-7.93, 10.48)	-6.78 (-14.21, 3.93)	-5.12 (-12.30, 5.11)	2.39 (-5.42, 13.86)	-8.69 (-19.06, 4.57)	-7.65 (-15.22, 2.90)	-0.73 (-6.75, 8.50)	3.62 (-7.22, 14.87)	Multidomain Therapy	1.00 (-2.94, 8.06)	-0.52 (-9.94, 10.34)	2.03 (-3.98, 12.62)	-4.23 (-12.91, 7.26)
-1.12 (-8.87, 5.85)	-7.92 (-14.97, -0.45)	-6.05 (-13.15, 1.03)	1.27 (-5.95, 9.45)	-9.89 (-20.21, 1.09)	-8.75 (-15.87, -1.45)	-2.04 (-7.08, 3.72)	2.22 (-8.09, 11.54)	-1.00 (-8.06, 2.94)	Placebo	-1.78 (-10.63, 6.35)	0.85 (-4.21, 7.94)	-5.46 (-13.57, 3.33)
0.49 (-10.59, 11.88)	-5.79 (-16.69, 5.31)	-4.31 (-14.65, 6.98)	3.25 (-7.34, 15.57)	-8.34 (-21.23, 6.01)	-6.88 (-17.52, 4.69)	0.03 (-9.71, 10.33)	4.06 (-9.06, 16.57)	0.52 (-10.34, 9.94)	1.78 (-6.35, 10.63)	Psychosoical Therapy	2.92 (-6.44, 14.40)	-3.46 (-14.92, 9.49)
-1.94 (-12.97, 5.84)	-8.83 (-18.99, -0.52)	-6.76 (-17.33, 0.91)	0.71 (-8.08, 8.08)	-10.09 (-23.65, 0.26)	-9.24 (-20.10, -1.20)	-2.38 (-11.78, 4.32)	0.99 (-11.51, 12.13)	-2.03 (-12.62, 3.98)	-0.85 (-7.94, 4.21)	-2.92 (-14.40, 6.44)	Reminiscence Therapy	-6.63 (-17.63, 4.09)
4.07 (-7.58, 15.04)	-2.17 (-13.85, 8.33)	-0.56 (-11.59, 10.06)	7.06 (-4.72, 18.48)	-3.76 (-18.18, 9.12)	-3.16 (-14.63, 7.72)	3.72 (-6.64, 13.34)	7.54 (-6.00, 19.74)	4.23 (-7.26, 12.91)	5.46 (-3.33, 13.57)	3.46 (-9.49, 14.92)	6.63 (-4.09, 17.63)	Sertraline
