# Supplementary material for: Comparative efficacy and acceptability of treatments for depressive symptoms in cognitive impairment: A systematic review and Bayesian network meta-analysis
Source: Front Aging Neurosci. 2022 Dec 12;14:1037414. doi: 10.3389/fnagi.2022.1037414 (PMC9790988; doi:10.3389/fnagi.2022.1037414)
Supplement: Supplementary material 1 — PRISMA NMA checklist. [file Data_Sheet_1.zip › Supplementary Material Presentation/Supplementary_8.pdf]

A. Cornell Scale for Depression

| Drug | Rank 1 | Rank 2 | Rank 3 | Rank 4 | Rank 5 | Rank 6 | Rank 7 | Rank 8 | Rank 9 | Rank 10 | Rank 11 | Rank 12 | Rank 13 | Rank 14 | Rank 15 | Rank 16 | Rank 17 | Rank 18 | Rank 19 |
|---|---|---|---|---|---|---|---|---|---|---|---|---|---|---|---|---|---|---|---|
| Aerobic Exercise | 0 | 0.61 | 0.22 | 0.06 | 0.03 | 0.02 | 0.01 | 0.01 | 0.01 | 0.01 | 0 | 0 | 0 | 0 | 0 | 0 | 0.01 | 0.01 | 0 |
| Aripiprazole | 0 | 0.01 | 0.04 | 0.09 | 0.16 | 0.18 | 0.15 | 0.11 | 0.07 | 0.05 | 0.04 | 0.04 | 0.02 | 0.01 | 0.01 | 0.01 | 0.01 | 0.01 | 0 |
| Behavioral Training | 0 | 0 | 0 | 0.01 | 0.04 | 0.09 | 0.17 | 0.21 | 0.2 | 0.12 | 0.07 | 0.04 | 0.02 | 0.01 | 0 | 0 | 0 | 0 | 0 |
| Citalopram | 0 | 0.02 | 0.03 | 0.07 | 0.1 | 0.09 | 0.09 | 0.07 | 0.05 | 0.05 | 0.05 | 0.04 | 0.04 | 0.05 | 0.04 | 0.04 | 0.05 | 0.05 | 0.08 |
| Cognitive stimulated training | 0 | 0 | 0 | 0 | 0 | 0 | 0 | 0.01 | 0.02 | 0.04 | 0.06 | 0.08 | 0.11 | 0.12 | 0.14 | 0.13 | 0.11 | 0.09 | 0.08 |
| Discontinuation of antipsychotics | 0 | 0 | 0.01 | 0.02 | 0.05 | 0.07 | 0.08 | 0.08 | 0.07 | 0.06 | 0.06 | 0.06 | 0.05 | 0.04 | 0.04 | 0.05 | 0.05 | 0.09 | 0.12 |
| Electrical Current Therapy | 0 | 0.2 | 0.15 | 0.35 | 0.11 | 0.07 | 0.04 | 0.02 | 0.02 | 0.01 | 0.02 | 0.01 | 0.01 | 0 | 0 | 0 | 0 | 0 | 0 |
| Exercise | 0 | 0 | 0 | 0 | 0 | 0.01 | 0.02 | 0.03 | 0.04 | 0.07 | 0.08 | 0.11 | 0.11 | 0.12 | 0.1 | 0.1 | 0.08 | 0.1 | 0.04 |
| Light therapy | 0 | 0 | 0.01 | 0.02 | 0.03 | 0.05 | 0.06 | 0.07 | 0.09 | 0.1 | 0.11 | 0.1 | 0.08 | 0.06 | 0.05 | 0.04 | 0.04 | 0.04 | 0.04 |
| Massage | 1 | 0 | 0 | 0 | 0 | 0 | 0 | 0 | 0 | 0 | 0 | 0 | 0 | 0 | 0 | 0 | 0 | 0 | 0 |
| Mirtazapine | 0 | 0 | 0 | 0.01 | 0.01 | 0.04 | 0.05 | 0.07 | 0.09 | 0.11 | 0.12 | 0.1 | 0.08 | 0.08 | 0.06 | 0.05 | 0.05 | 0.05 | 0.03 |
| Multidomain Therapy | 0 | 0 | 0 | 0.01 | 0.02 | 0.04 | 0.07 | 0.08 | 0.1 | 0.08 | 0.09 | 0.07 | 0.06 | 0.06 | 0.05 | 0.06 | 0.07 | 0.07 | 0.06 |
| Occupational Therapy | 0 | 0.03 | 0.06 | 0.07 | 0.07 | 0.07 | 0.05 | 0.05 | 0.05 | 0.04 | 0.04 | 0.03 | 0.03 | 0.02 | 0.02 | 0.03 | 0.03 | 0.06 | 0.25 |
| Placebo | 0 | 0 | 0 | 0 | 0 | 0 | 0 | 0 | 0 | 0 | 0 | 0.02 | 0.05 | 0.1 | 0.18 | 0.23 | 0.23 | 0.14 | 0.05 |
| Psychosoical Therapy | 0 | 0 | 0 | 0 | 0.01 | 0.01 | 0.03 | 0.05 | 0.08 | 0.09 | 0.11 | 0.1 | 0.12 | 0.1 | 0.08 | 0.07 | 0.06 | 0.06 | 0.04 |
| Reminiscence Therapy | 0 | 0.02 | 0.06 | 0.14 | 0.3 | 0.2 | 0.12 | 0.08 | 0.04 | 0.02 | 0.01 | 0.01 | 0.01 | 0 | 0 | 0 | 0 | 0 | 0 |
| Sertraline | 0 | 0 | 0 | 0 | 0 | 0.01 | 0.02 | 0.03 | 0.05 | 0.08 | 0.11 | 0.13 | 0.15 | 0.11 | 0.11 | 0.07 | 0.07 | 0.05 | 0.02 |
| Strength Exercise | 0 | 0.11 | 0.42 | 0.15 | 0.07 | 0.05 | 0.03 | 0.02 | 0.02 | 0.01 | 0.01 | 0.01 | 0.01 | 0.01 | 0.01 | 0.01 | 0.01 | 0.02 | 0.03 |
| Tai Chi | 0 | 0 | 0 | 0 | 0 | 0.01 | 0.01 | 0.01 | 0.02 | 0.03 | 0.03 | 0.04 | 0.07 | 0.09 | 0.1 | 0.13 | 0.15 | 0.18 | 0.13 |

B. Geriatric Depression Scale

| Drug | Rank 1 | Rank 2 | Rank 3 | Rank 4 | Rank 5 | Rank 6 | Rank 7 | Rank 8 | Rank 9 | Rank 10 | Rank 11 | Rank 12 |
|---|---|---|---|---|---|---|---|---|---|---|---|---|
| Behavioral Training | 0 | 0.02 | 0.06 | 0.13 | 0.21 | 0.18 | 0.13 | 0.09 | 0.06 | 0.05 | 0.04 | 0.02 |
| Citalopram | 0.01 | 0.03 | 0.08 | 0.1 | 0.12 | 0.13 | 0.12 | 0.09 | 0.07 | 0.08 | 0.09 | 0.08 |
| Cognitive stimulated training | 0 | 0.01 | 0.03 | 0.04 | 0.06 | 0.08 | 0.1 | 0.11 | 0.11 | 0.12 | 0.15 | 0.17 |
| Exercise | 0 | 0.01 | 0.03 | 0.05 | 0.07 | 0.09 | 0.11 | 0.1 | 0.1 | 0.12 | 0.14 | 0.17 |
| Hormone Therapy | 0 | 0.01 | 0.02 | 0.04 | 0.05 | 0.07 | 0.08 | 0.09 | 0.1 | 0.12 | 0.17 | 0.24 |
| Massage | 0.02 | 0.07 | 0.21 | 0.21 | 0.14 | 0.1 | 0.07 | 0.05 | 0.04 | 0.03 | 0.03 | 0.02 |
| Multidomain Therapy | 0.2 | 0.56 | 0.1 | 0.05 | 0.03 | 0.02 | 0.01 | 0.01 | 0.01 | 0.01 | 0 | 0 |
| Music Therapy | 0.74 | 0.18 | 0.04 | 0.02 | 0.01 | 0.01 | 0 | 0 | 0 | 0 | 0 | 0 |
| Placebo | 0 | 0 | 0 | 0 | 0.01 | 0.03 | 0.1 | 0.22 | 0.29 | 0.23 | 0.1 | 0.02 |
| Psychosoical Therapy | 0.01 | 0.02 | 0.04 | 0.06 | 0.09 | 0.12 | 0.12 | 0.11 | 0.1 | 0.1 | 0.12 | 0.12 |
| Reminiscence Therapy | 0.01 | 0.07 | 0.36 | 0.26 | 0.14 | 0.08 | 0.04 | 0.02 | 0.01 | 0.01 | 0 | 0 |
| Sertraline | 0 | 0.01 | 0.03 | 0.05 | 0.07 | 0.09 | 0.1 | 0.11 | 0.1 | 0.13 | 0.15 | 0.16 |

C. Hamilton Depression Rating Scale

| Drug | Rank 1 | Rank 2 | Rank 3 | Rank 4 | Rank 5 | Rank 6 | Rank 7 | Rank 8 | Rank 9 | Rank 10 | Rank 11 | Rank 12 |
|---|---|---|---|---|---|---|---|---|---|---|---|---|
| Amitriptyline | 0 | 0 | 0 | 0 | 0 | 0.01 | 0.01 | 0.01 | 0.01 | 0.02 | 0.05 | 0.88 |
| Behavioral Training | 0.05 | 0.29 | 0.19 | 0.2 | 0.13 | 0.06 | 0.03 | 0.02 | 0.01 | 0.01 | 0.01 | 0.01 |
| Cognitive stimulated training | 0.04 | 0.15 | 0.19 | 0.24 | 0.2 | 0.08 | 0.04 | 0.02 | 0.01 | 0.01 | 0.01 | 0.01 |
| Exercise | 0 | 0.01 | 0.02 | 0.02 | 0.04 | 0.06 | 0.15 | 0.25 | 0.2 | 0.16 | 0.07 | 0.03 |
| Fluoxetine | 0 | 0 | 0.01 | 0.01 | 0.02 | 0.02 | 0.04 | 0.06 | 0.07 | 0.14 | 0.6 | 0.01 |
| Hormone Therapy | 0.01 | 0.02 | 0.04 | 0.06 | 0.08 | 0.14 | 0.3 | 0.09 | 0.1 | 0.11 | 0.05 | 0.01 |
| Imipramine | 0 | 0 | 0.01 | 0.01 | 0.02 | 0.04 | 0.07 | 0.12 | 0.18 | 0.38 | 0.15 | 0.03 |
| Music Therapy | 0.76 | 0.1 | 0.05 | 0.03 | 0.02 | 0.01 | 0.01 | 0.01 | 0 | 0 | 0 | 0 |
| Placebo | 0 | 0 | 0 | 0 | 0.01 | 0.04 | 0.14 | 0.34 | 0.35 | 0.1 | 0.02 | 0 |
| Rivastigmine | 0.02 | 0.03 | 0.05 | 0.08 | 0.19 | 0.37 | 0.13 | 0.05 | 0.03 | 0.03 | 0.02 | 0.01 |
| Sertraline | 0.04 | 0.23 | 0.31 | 0.2 | 0.11 | 0.05 | 0.03 | 0.01 | 0.01 | 0.01 | 0 | 0 |
| Venlafaxine | 0.08 | 0.16 | 0.13 | 0.13 | 0.18 | 0.13 | 0.06 | 0.03 | 0.03 | 0.03 | 0.02 | 0.01 |

D. Mini-Mental State Examination

| Drug | Rank 1 | Rank 2 | Rank 3 | Rank 4 | Rank 5 | Rank 6 | Rank 7 | Rank 8 | Rank 9 | Rank 10 | Rank 11 | Rank 12 | Rank 13 | Rank 14 | Rank 15 | Rank 16 | Rank 17 | Rank 18 |
|---|---|---|---|---|---|---|---|---|---|---|---|---|---|---|---|---|---|---|
| Amitriptyline | 0.03 | 0.06 | 0.06 | 0.08 | 0.08 | 0.08 | 0.08 | 0.07 | 0.06 | 0.05 | 0.05 | 0.05 | 0.05 | 0.05 | 0.04 | 0.04 | 0.03 | 0.03 |
| Aripiprazole | 0 | 0.01 | 0.01 | 0.01 | 0.02 | 0.02 | 0.02 | 0.03 | 0.03 | 0.04 | 0.04 | 0.05 | 0.06 | 0.08 | 0.09 | 0.12 | 0.16 | 0.22 |
| Behavioral Training | 0 | 0.02 | 0.03 | 0.05 | 0.07 | 0.09 | 0.1 | 0.1 | 0.1 | 0.09 | 0.07 | 0.07 | 0.07 | 0.06 | 0.04 | 0.04 | 0.02 | 0.01 |
| Citalopram | 0.05 | 0.08 | 0.08 | 0.09 | 0.09 | 0.09 | 0.08 | 0.08 | 0.06 | 0.05 | 0.04 | 0.04 | 0.03 | 0.03 | 0.03 | 0.02 | 0.02 | 0.01 |
| Cognitive stimulated training | 0.09 | 0.23 | 0.22 | 0.16 | 0.11 | 0.07 | 0.05 | 0.03 | 0.02 | 0.01 | 0.01 | 0 | 0 | 0 | 0 | 0 | 0 | 0 |
| Electrical Current Therapy | 0.58 | 0.14 | 0.08 | 0.05 | 0.04 | 0.03 | 0.02 | 0.01 | 0.01 | 0.01 | 0.01 | 0.01 | 0 | 0 | 0 | 0 | 0 | 0 |
| EGb 761 | 0.01 | 0.03 | 0.04 | 0.05 | 0.06 | 0.07 | 0.06 | 0.07 | 0.07 | 0.06 | 0.06 | 0.06 | 0.06 | 0.07 | 0.07 | 0.06 | 0.06 | 0.05 |
| Exercise | 0.02 | 0.03 | 0.05 | 0.05 | 0.06 | 0.06 | 0.07 | 0.07 | 0.07 | 0.06 | 0.06 | 0.06 | 0.06 | 0.06 | 0.06 | 0.06 | 0.05 | 0.04 |
| Fluoxetine | 0 | 0.01 | 0.01 | 0.01 | 0.02 | 0.02 | 0.03 | 0.04 | 0.04 | 0.04 | 0.05 | 0.05 | 0.07 | 0.08 | 0.11 | 0.13 | 0.15 | 0.16 |
| Hormone Therapy | 0 | 0.01 | 0.02 | 0.03 | 0.04 | 0.06 | 0.07 | 0.07 | 0.07 | 0.07 | 0.07 | 0.07 | 0.08 | 0.08 | 0.08 | 0.07 | 0.05 | 0.04 |
| Imipramine | 0 | 0.01 | 0.01 | 0.02 | 0.02 | 0.04 | 0.05 | 0.05 | 0.06 | 0.05 | 0.06 | 0.07 | 0.08 | 0.09 | 0.1 | 0.11 | 0.1 | 0.08 |
| Massage | 0.05 | 0.09 | 0.08 | 0.09 | 0.08 | 0.08 | 0.07 | 0.07 | 0.06 | 0.05 | 0.04 | 0.04 | 0.04 | 0.04 | 0.04 | 0.03 | 0.03 | 0.02 |
| Multidomain Therapy | 0.02 | 0.04 | 0.04 | 0.05 | 0.05 | 0.06 | 0.06 | 0.05 | 0.05 | 0.05 | 0.05 | 0.05 | 0.06 | 0.06 | 0.07 | 0.07 | 0.08 | 0.09 |
| Placebo | 0 | 0 | 0 | 0 | 0 | 0 | 0.02 | 0.05 | 0.12 | 0.19 | 0.22 | 0.21 | 0.12 | 0.05 | 0.02 | 0 | 0 | 0 |
| Psychosoical Therapy | 0 | 0.01 | 0.01 | 0.02 | 0.03 | 0.04 | 0.05 | 0.06 | 0.07 | 0.07 | 0.08 | 0.09 | 0.1 | 0.11 | 0.09 | 0.08 | 0.06 | 0.03 |
| Reminiscence Therapy | 0.05 | 0.12 | 0.14 | 0.13 | 0.13 | 0.11 | 0.08 | 0.06 | 0.05 | 0.03 | 0.02 | 0.02 | 0.02 | 0.01 | 0.01 | 0 | 0 | 0 |
| Rivastigmine | 0.08 | 0.13 | 0.1 | 0.1 | 0.09 | 0.08 | 0.07 | 0.06 | 0.05 | 0.04 | 0.03 | 0.03 | 0.03 | 0.03 | 0.03 | 0.02 | 0.02 | 0.01 |
| Sertraline | 0 | 0 | 0 | 0.01 | 0.01 | 0.01 | 0.02 | 0.03 | 0.03 | 0.03 | 0.04 | 0.05 | 0.06 | 0.08 | 0.11 | 0.13 | 0.17 | 0.21 |

E. Neuropsychiatric Inventory

| Drug | Rank 1 | Rank 2 | Rank 3 | Rank 4 | Rank 5 | Rank 6 | Rank 7 | Rank 8 | Rank 9 | Rank 10 | Rank 11 | Rank 12 | Rank 13 |
|---|---|---|---|---|---|---|---|---|---|---|---|---|---|
| Aripiprazole | 0.01 | 0.01 | 0.02 | 0.03 | 0.05 | 0.11 | 0.14 | 0.18 | 0.17 | 0.1 | 0.07 | 0.06 | 0.04 |
| Behavioral Training | 0.17 | 0.24 | 0.26 | 0.15 | 0.08 | 0.04 | 0.02 | 0.01 | 0.01 | 0.01 | 0 | 0 | 0 |
| Citalopram | 0.05 | 0.07 | 0.13 | 0.29 | 0.29 | 0.07 | 0.04 | 0.02 | 0.02 | 0.01 | 0.01 | 0.01 | 0 |
| Cognitive stimulated training | 0 | 0 | 0.01 | 0.01 | 0.03 | 0.05 | 0.05 | 0.06 | 0.06 | 0.07 | 0.12 | 0.29 | 0.26 |
| Donepezil | 0.51 | 0.18 | 0.13 | 0.06 | 0.04 | 0.02 | 0.02 | 0.01 | 0.01 | 0.01 | 0.01 | 0.01 | 0.01 |
| EGb 761 | 0.16 | 0.37 | 0.2 | 0.16 | 0.05 | 0.02 | 0.01 | 0.01 | 0.01 | 0.01 | 0 | 0 | 0 |
| Exercise | 0 | 0.01 | 0.01 | 0.03 | 0.06 | 0.23 | 0.22 | 0.14 | 0.11 | 0.09 | 0.05 | 0.04 | 0.02 |
| Light therapy | 0.01 | 0.01 | 0.02 | 0.02 | 0.04 | 0.05 | 0.06 | 0.05 | 0.05 | 0.07 | 0.1 | 0.13 | 0.4 |
| Multidomain Therapy | 0 | 0.01 | 0.02 | 0.03 | 0.06 | 0.1 | 0.13 | 0.17 | 0.2 | 0.17 | 0.07 | 0.03 | 0.01 |
| Placebo | 0 | 0 | 0 | 0 | 0 | 0.01 | 0.03 | 0.09 | 0.18 | 0.28 | 0.32 | 0.08 | 0.01 |
| Psychosoical Therapy | 0.02 | 0.03 | 0.04 | 0.06 | 0.09 | 0.17 | 0.15 | 0.09 | 0.08 | 0.07 | 0.06 | 0.08 | 0.07 |
| Reminiscence Therapy | 0 | 0 | 0 | 0.01 | 0.02 | 0.03 | 0.06 | 0.13 | 0.09 | 0.1 | 0.16 | 0.25 | 0.15 |
| Sertraline | 0.07 | 0.08 | 0.17 | 0.15 | 0.21 | 0.09 | 0.06 | 0.05 | 0.03 | 0.03 | 0.02 | 0.02 | 0.02 |
