# Supplementary material for: Comparative efficacy and acceptability of treatments for depressive symptoms in cognitive impairment: A systematic review and Bayesian network meta-analysis
Source: Front Aging Neurosci. 2022 Dec 12;14:1037414. doi: 10.3389/fnagi.2022.1037414 (PMC9790988; doi:10.3389/fnagi.2022.1037414)
Supplement: Supplementary material 1 — PRISMA NMA checklist. [file Data_Sheet_1.zip › Supplementary Material Presentation/Supplementary_9.pdf]

Cornell Scale for Depression

| Comparison | Number of studies | Within-study bias | Reporting bias | Indirectness | Imprecision | Heterogeneity | Incoherence | Confidence rating |
|---|---|---|---|---|---|---|---|---|
| AEXE:PLA | 1 | Some concerns | Low risk | No concerns | No concerns | No concerns | No concerns | High |
| AEXE:SEXE | 1 | Some concerns | Low risk | No concerns | No concerns | No concerns | No concerns | High |
| ARI:PLA | 1 | Some concerns | Low risk | No concerns | No concerns | No concerns | No concerns | High |
| BT:PLA | 6 | Some concerns | Low risk | No concerns | No concerns | No concerns | No concerns | High |
| CIT:PLA | 1 | Some concerns | Low risk | No concerns | No concerns | Some concerns | No concerns | High |
| CST:EXE | 1 | Some concerns | Low risk | No concerns | No concerns | No concerns | No concerns | High |
| CST:PLA | 4 | Some concerns | Low risk | No concerns | No concerns | No concerns | No concerns | High |
| DIS:PLA | 1 | Some concerns | Low risk | No concerns | No concerns | No concerns | No concerns | High |
| ECT:PLA | 1 | Some concerns | Low risk | No concerns | No concerns | No concerns | No concerns | High |
| EXE:MLT | 1 | Some concerns | Low risk | No concerns | No concerns | No concerns | No concerns | High |
| EXE:PLA | 1 | Some concerns | Low risk | No concerns | No concerns | No concerns | No concerns | High |
| LGT:PLA | 2 | Some concerns | Low risk | No concerns | No concerns | No concerns | No concerns | High |
| MAS:PLA | 1 | Some concerns | Low risk | No concerns | No concerns | No concerns | No concerns | High |
| MIR:PLA | 5 | Major concerns | Low risk | Some concerns | No concerns | No concerns | No concerns | High |
| MIR:SER | 5 | Major concerns | Low risk | Some concerns | No concerns | No concerns | Some concerns | High |
| MLT:PLA | 5 | Some concerns | Low risk | No concerns | No concerns | No concerns | No concerns | High |
| MUT:PLA | 2 | Some concerns | Low risk | No concerns | No concerns | No concerns | No concerns | High |
| OPT:PLA | 3 | Some concerns | Low risk | No concerns | No concerns | No concerns | No concerns | High |
| PLA:PRAT | 1 | Some concerns | Low risk | No concerns | Some concerns | No concerns | No concerns | High |
| PLA:PSI | 1 | Some concerns | Low risk | No concerns | No concerns | No concerns | No concerns | High |
| PLA:RET | 5 | Some concerns | Low risk | No concerns | No concerns | No concerns | No concerns | High |
| PLA:SER | 8 | Major concerns | Low risk | Some concerns | No concerns | No concerns | No concerns | High |
| PLA:TAI | 1 | Some concerns | Low risk | No concerns | No concerns | No concerns | No concerns | High |
| SER:VEN | 1 | Some concerns | Low risk | No concerns | No concerns | No concerns | No concerns | High |
| AEXE:ARI | 0 | Some concerns | Low risk | No concerns | Some concerns | No concerns | No concerns | High |
| AEXE:BT | 0 | Some concerns | Low risk | No concerns | Some concerns | No concerns | No concerns | High |
| AEXE:CIT | 0 | Some concerns | Low risk | No concerns | Some concerns | No concerns | No concerns | High |
| AEXE:CST | 0 | Some concerns | Low risk | No concerns | No concerns | No concerns | No concerns | High |
| AEXE:DIS | 0 | Some concerns | Low risk | No concerns | Some concerns | No concerns | No concerns | High |
| AEXE:ECT | 0 | Some concerns | Low risk | No concerns | Some concerns | No concerns | No concerns | High |
| AEXE:EXE | 0 | Some concerns | Low risk | No concerns | No concerns | Some concerns | No concerns | High |
| AEXE:LGT | 0 | Some concerns | Low risk | No concerns | Some concerns | No concerns | No concerns | High |
| AEXE:MAS | 0 | Some concerns | Low risk | No concerns | No concerns | No concerns | No concerns | High |
| AEXE:MIR | 0 | Major concerns | Low risk | Some concerns | Some concerns | No concerns | No concerns | High |
| AEXE:MLT | 0 | Some concerns | Low risk | No concerns | No concerns | Some concerns | No concerns | High |
| AEXE:MUT | 0 | Some concerns | Low risk | No concerns | Some concerns | No concerns | No concerns | High |
| AEXE:OPT | 0 | Some concerns | Low risk | No concerns | Some concerns | No concerns | No concerns | High |
| AEXE:PRAT | 0 | Some concerns | Low risk | No concerns | Some concerns | No concerns | No concerns | High |
| AEXE:PSI | 0 | Some concerns | Low risk | No concerns | Some concerns | No concerns | No concerns | High |
| AEXE:RET | 0 | Some concerns | Low risk | No concerns | Some concerns | No concerns | No concerns | High |
| AEXE:SER | 0 | Some concerns | Low risk | No concerns | No concerns | Some concerns | No concerns | High |
| AEXE:TAI | 0 | Some concerns | Low risk | No concerns | No concerns | No concerns | No concerns | High |
| AEXE:VEN | 0 | Some concerns | Low risk | No concerns | No concerns | No concerns | No concerns | High |
| ARI:BT | 0 | Some concerns | Low risk | No concerns | No concerns | No concerns | No concerns | High |
| ARI:CIT | 0 | Some concerns | Low risk | No concerns | No concerns | No concerns | No concerns | High |
| ARI:CST | 0 | Some concerns | Low risk | No concerns | No concerns | No concerns | No concerns | High |
| ARI:DIS | 0 | Some concerns | Low risk | No concerns | No concerns | Some concerns | No concerns | High |
| ARI:ECT | 0 | Some concerns | Low risk | No concerns | Some concerns | No concerns | No concerns | High |
| ARI:EXE | 0 | Some concerns | Low risk | No concerns | No concerns | No concerns | No concerns | High |
| ARI:LGT | 0 | Some concerns | Low risk | No concerns | No concerns | No concerns | No concerns | High |
| ARI:MAS | 0 | Some concerns | Low risk | No concerns | No concerns | No concerns | No concerns | High |
| ARI:MIR | 0 | Major concerns | Low risk | Some concerns | No concerns | No concerns | No concerns | High |
| ARI:MLT | 0 | Some concerns | Low risk | No concerns | No concerns | No concerns | No concerns | High |
| ARI:MUT | 0 | Some concerns | Low risk | No concerns | No concerns | No concerns | No concerns | High |
| ARI:OPT | 0 | Some concerns | Low risk | No concerns | No concerns | No concerns | No concerns | High |
| ARI:PRAT | 0 | Some concerns | Low risk | No concerns | Some concerns | No concerns | No concerns | High |
| ARI:PSI | 0 | Some concerns | Low risk | No concerns | No concerns | No concerns | No concerns | High |
| ARI:RET | 0 | Some concerns | Low risk | No concerns | No concerns | No concerns | No concerns | High |
| ARI:SER | 0 | Some concerns | Low risk | No concerns | No concerns | No concerns | No concerns | High |
| ARI:SEXE | 0 | Some concerns | Low risk | No concerns | Some concerns | No concerns | No concerns | High |
| ARI:TAI | 0 | Some concerns | Low risk | No concerns | No concerns | Some concerns | No concerns | High |
| ARI:VEN | 0 | Some concerns | Low risk | No concerns | No concerns | No concerns | No concerns | High |
| BT:CIT | 0 | Some concerns | Low risk | No concerns | No concerns | No concerns | No concerns | High |
| BT:CST | 0 | Some concerns | Low risk | No concerns | No concerns | No concerns | No concerns | High |
| BT:DIS | 0 | Some concerns | Low risk | No concerns | No concerns | No concerns | No concerns | High |
| BT:ECT | 0 | Some concerns | Low risk | No concerns | Some concerns | No concerns | No concerns | High |
| BT:EXE | 0 | Some concerns | Low risk | No concerns | No concerns | No concerns | No concerns | High |
| BT:LGT | 0 | Some concerns | Low risk | No concerns | No concerns | No concerns | No concerns | High |
| BT:MAS | 0 | Some concerns | Low risk | No concerns | No concerns | No concerns | No concerns | High |
| BT:MIR | 0 | Major concerns | Low risk | Some concerns | No concerns | No concerns | No concerns | High |
| BT:MLT | 0 | Some concerns | Low risk | No concerns | No concerns | No concerns | No concerns | High |
| BT:MUT | 0 | Some concerns | Low risk | No concerns | No concerns | Some concerns | No concerns | High |
| BT:OPT | 0 | Some concerns | Low risk | No concerns | No concerns | No concerns | No concerns | High |
| BT:PRAT | 0 | Some concerns | Low risk | No concerns | Some concerns | No concerns | No concerns | High |
| BT:PSI | 0 | Some concerns | Low risk | No concerns | No concerns | No concerns | No concerns | High |
| BT:RET | 0 | Some concerns | Low risk | No concerns | No concerns | No concerns | No concerns | High |
| BT:SER | 0 | Some concerns | Low risk | No concerns | No concerns | No concerns | No concerns | High |
| BT:SEXE | 0 | Some concerns | Low risk | No concerns | Some concerns | No concerns | No concerns | High |
| BT:TAI | 0 | Some concerns | Low risk | No concerns | No concerns | No concerns | No concerns | High |
| BT:VEN | 0 | Some concerns | Low risk | No concerns | No concerns | No concerns | No concerns | High |
| CIT:CST | 0 | Some concerns | Low risk | No concerns | No concerns | Some concerns | No concerns | High |
| CIT:DIS | 0 | Some concerns | Low risk | No concerns | No concerns | Some concerns | No concerns | High |
| CIT:ECT | 0 | Some concerns | Low risk | No concerns | Some concerns | No concerns | No concerns | High |
| CIT:EXE | 0 | Some concerns | Low risk | No concerns | No concerns | No concerns | No concerns | High |
| CIT:LGT | 0 | Some concerns | Low risk | No concerns | No concerns | No concerns | No concerns | High |
| CIT:MAS | 0 | Some concerns | Low risk | No concerns | No concerns | No concerns | No concerns | High |
| CIT:MIR | 0 | Major concerns | Low risk | Some concerns | No concerns | No concerns | No concerns | High |
| CIT:MLT | 0 | Some concerns | Low risk | No concerns | No concerns | No concerns | No concerns | High |
| CIT:MUT | 0 | Some concerns | Low risk | No concerns | Some concerns | No concerns | No concerns | High |

| | | | | | | | | |
|---|---|---|---|---|---|---|---|---|
| CIT:OPT | 0 | Some concerns | Low risk | No concerns | No concerns | No concerns | No concerns | High |
| CIT:PRAT | 0 | Some concerns | Low risk | No concerns | Some concerns | No concerns | No concerns | High |
| CIT:PSI | 0 | Some concerns | Low risk | No concerns | No concerns | No concerns | No concerns | High |
| CIT:RET | 0 | Some concerns | Low risk | No concerns | No concerns | Some concerns | No concerns | High |
| CIT:SER | 0 | Some concerns | Low risk | No concerns | No concerns | No concerns | No concerns | High |
| CIT:SEXE | 0 | Some concerns | Low risk | No concerns | Some concerns | No concerns | No concerns | High |
| CIT:TAI | 0 | Some concerns | Low risk | No concerns | No concerns | Some concerns | No concerns | High |
| CIT:VEN | 0 | Some concerns | Low risk | No concerns | No concerns | No concerns | No concerns | High |
| CST:DIS | 0 | Some concerns | Low risk | No concerns | No concerns | No concerns | No concerns | High |
| CST:ECT | 0 | Some concerns | Low risk | No concerns | No concerns | No concerns | No concerns | High |
| CST:LGT | 0 | Some concerns | Low risk | No concerns | No concerns | No concerns | No concerns | High |
| CST:MAS | 0 | Some concerns | Low risk | No concerns | No concerns | No concerns | No concerns | High |
| CST:MIR | 0 | Some concerns | Low risk | No concerns | No concerns | No concerns | No concerns | High |
| CST:MLT | 0 | Some concerns | Low risk | No concerns | No concerns | No concerns | No concerns | High |
| CST:MUT | 0 | Some concerns | Low risk | No concerns | No concerns | Some concerns | No concerns | High |
| CST:OPT | 0 | Some concerns | Low risk | No concerns | No concerns | No concerns | No concerns | High |
| CST:PRAT | 0 | Some concerns | Low risk | No concerns | Some concerns | No concerns | No concerns | High |
| CST:PSI | 0 | Some concerns | Low risk | No concerns | No concerns | No concerns | No concerns | High |
| CST:RET | 0 | Some concerns | Low risk | No concerns | No concerns | No concerns | No concerns | High |
| CST:SER | 0 | Some concerns | Low risk | No concerns | No concerns | No concerns | No concerns | High |
| CST:SEXE | 0 | Some concerns | Low risk | No concerns | Some concerns | No concerns | No concerns | High |
| CST:TAI | 0 | Some concerns | Low risk | No concerns | No concerns | No concerns | No concerns | High |
| CST:VEN | 0 | Some concerns | Low risk | No concerns | No concerns | No concerns | No concerns | High |
| DIS:ECT | 0 | Some concerns | Low risk | No concerns | Some concerns | No concerns | No concerns | High |
| DIS:EXE | 0 | Some concerns | Low risk | No concerns | No concerns | No concerns | No concerns | High |
| DIS:LGT | 0 | Some concerns | Low risk | No concerns | No concerns | No concerns | No concerns | High |
| DIS:MAS | 0 | Some concerns | Low risk | No concerns | No concerns | No concerns | No concerns | High |
| DIS:MIR | 0 | Major concerns | Low risk | Some concerns | No concerns | No concerns | No concerns | High |
| DIS:MLT | 0 | Some concerns | Low risk | No concerns | No concerns | No concerns | No concerns | High |
| DIS:MUT | 0 | Some concerns | Low risk | No concerns | Some concerns | No concerns | No concerns | High |
| DIS:OPT | 0 | Some concerns | Low risk | No concerns | No concerns | No concerns | No concerns | High |
| DIS:PRAT | 0 | Some concerns | Low risk | No concerns | Some concerns | No concerns | No concerns | High |
| DIS:PSI | 0 | Some concerns | Low risk | No concerns | No concerns | No concerns | No concerns | High |
| DIS:RET | 0 | Some concerns | Low risk | No concerns | Some concerns | No concerns | No concerns | High |
| DIS:SER | 0 | Some concerns | Low risk | No concerns | No concerns | No concerns | No concerns | High |
| DIS:SEXE | 0 | Some concerns | Low risk | No concerns | Some concerns | No concerns | No concerns | High |
| DIS:TAI | 0 | Some concerns | Low risk | No concerns | No concerns | No concerns | No concerns | High |
| DIS:VEN | 0 | Some concerns | Low risk | No concerns | No concerns | No concerns | No concerns | High |
| ECT:EXE | 0 | Some concerns | Low risk | No concerns | No concerns | No concerns | No concerns | High |
| ECT:LGT | 0 | Some concerns | Low risk | No concerns | Some concerns | No concerns | No concerns | High |
| ECT:MAS | 0 | Some concerns | Low risk | No concerns | No concerns | No concerns | No concerns | High |
| ECT:MIR | 0 | Major concerns | Low risk | Some concerns | No concerns | Some concerns | No concerns | High |
| ECT:MLT | 0 | Some concerns | Low risk | No concerns | No concerns | Some concerns | No concerns | High |
| ECT:MUT | 0 | Some concerns | Low risk | No concerns | No concerns | Some concerns | No concerns | High |
| ECT:OPT | 0 | Some concerns | Low risk | No concerns | Some concerns | No concerns | No concerns | High |
| ECT:PRAT | 0 | Some concerns | Low risk | No concerns | Some concerns | No concerns | No concerns | High |
| ECT:PSI | 0 | Some concerns | Low risk | No concerns | Some concerns | No concerns | No concerns | High |
| ECT:RET | 0 | Some concerns | Low risk | No concerns | No concerns | No concerns | No concerns | High |
| ECT:SER | 0 | Some concerns | Low risk | No concerns | No concerns | No concerns | No concerns | High |
| ECT:SEXE | 0 | Some concerns | Low risk | No concerns | Major concerns | No concerns | No concerns | High |
| ECT:TAI | 0 | Some concerns | Low risk | No concerns | No concerns | No concerns | No concerns | High |
| ECT:VEN | 0 | Some concerns | Low risk | No concerns | No concerns | No concerns | No concerns | High |
| EXE:LGT | 0 | Some concerns | Low risk | No concerns | No concerns | No concerns | No concerns | High |
| EXE:MAS | 0 | Some concerns | Low risk | No concerns | No concerns | No concerns | No concerns | High |
| EXE:MIR | 0 | Some concerns | Low risk | No concerns | No concerns | No concerns | No concerns | High |
| EXE:MUT | 0 | Some concerns | Low risk | No concerns | No concerns | Some concerns | No concerns | High |
| EXE:OPT | 0 | Some concerns | Low risk | No concerns | No concerns | No concerns | No concerns | High |
| EXE:PRAT | 0 | Some concerns | Low risk | No concerns | Some concerns | No concerns | No concerns | High |
| EXE:PSI | 0 | Some concerns | Low risk | No concerns | No concerns | No concerns | No concerns | High |
| EXE:RET | 0 | Some concerns | Low risk | No concerns | No concerns | No concerns | No concerns | High |
| EXE:SER | 0 | Some concerns | Low risk | No concerns | No concerns | No concerns | No concerns | High |
| EXE:SEXE | 0 | Some concerns | Low risk | No concerns | Some concerns | No concerns | No concerns | High |
| EXE:TAI | 0 | Some concerns | Low risk | No concerns | No concerns | No concerns | No concerns | High |
| EXE:VEN | 0 | Some concerns | Low risk | No concerns | No concerns | No concerns | No concerns | High |
| LGT:MAS | 0 | Some concerns | Low risk | No concerns | No concerns | No concerns | No concerns | High |
| LGT:MIR | 0 | Major concerns | Low risk | Some concerns | No concerns | No concerns | No concerns | High |
| LGT:MLT | 0 | Some concerns | Low risk | No concerns | No concerns | No concerns | No concerns | High |
| LGT:MUT | 0 | Some concerns | Low risk | No concerns | Some concerns | No concerns | No concerns | High |
| LGT:OPT | 0 | Some concerns | Low risk | No concerns | No concerns | No concerns | No concerns | High |
| LGT:PRAT | 0 | Some concerns | Low risk | No concerns | Some concerns | No concerns | No concerns | High |
| LGT:PSI | 0 | Some concerns | Low risk | No concerns | No concerns | No concerns | No concerns | High |
| LGT:RET | 0 | Some concerns | Low risk | No concerns | No concerns | No concerns | No concerns | High |
| LGT:SER | 0 | Some concerns | Low risk | No concerns | No concerns | No concerns | No concerns | High |
| LGT:SEXE | 0 | Some concerns | Low risk | No concerns | Some concerns | No concerns | No concerns | High |
| LGT:TAI | 0 | Some concerns | Low risk | No concerns | No concerns | No concerns | No concerns | High |
| LGT:VEN | 0 | Some concerns | Low risk | No concerns | No concerns | No concerns | No concerns | High |
| MAS:MIR | 0 | Major concerns | Low risk | Some concerns | No concerns | No concerns | No concerns | High |
| MAS:MLT | 0 | Some concerns | Low risk | No concerns | No concerns | No concerns | No concerns | High |
| MAS:MUT | 0 | Some concerns | Low risk | No concerns | No concerns | No concerns | No concerns | High |
| MAS:OPT | 0 | Some concerns | Low risk | No concerns | No concerns | No concerns | No concerns | High |
| MAS:PRAT | 0 | Some concerns | Low risk | No concerns | No concerns | No concerns | No concerns | High |
| MAS:PSI | 0 | Some concerns | Low risk | No concerns | No concerns | No concerns | No concerns | High |
| MAS:RET | 0 | Some concerns | Low risk | No concerns | No concerns | No concerns | No concerns | High |
| MAS:SER | 0 | Some concerns | Low risk | No concerns | No concerns | No concerns | No concerns | High |
| MAS:SEXE | 0 | Some concerns | Low risk | No concerns | No concerns | No concerns | No concerns | High |
| MAS:TAI | 0 | Some concerns | Low risk | No concerns | No concerns | No concerns | No concerns | High |
| MAS:VEN | 0 | Some concerns | Low risk | No concerns | No concerns | No concerns | No concerns | High |
| MIR:MLT | 0 | Some concerns | Low risk | No concerns | No concerns | No concerns | No concerns | High |
| MIR:MUT | 0 | Some concerns | Low risk | Some concerns | Some concerns | No concerns | No concerns | High |
| MIR:OPT | 0 | Major concerns | Low risk | Some concerns | No concerns | No concerns | No concerns | High |
| MIR:PRAT | 0 | Some concerns | Low risk | Some concerns | Some concerns | No concerns | No concerns | High |

| | | | | | | | | |
|---|---|---|---|---|---|---|---|---|
| MIR:PSI | 0 | Some concerns | Low risk | Some concerns | No concerns | No concerns | No concerns | High |
| MIR:RET | 0 | Major concerns | Low risk | Some concerns | No concerns | No concerns | No concerns | High |
| MIR:SEXE | 0 | Some concerns | Low risk | No concerns | Some concerns | No concerns | No concerns | High |
| MIR:TAI | 0 | Major concerns | Low risk | Some concerns | No concerns | No concerns | No concerns | High |
| MIR:VEN | 0 | Major concerns | Low risk | Some concerns | No concerns | No concerns | No concerns | High |
| MLT:MUT | 0 | Some concerns | Low risk | No concerns | No concerns | Some concerns | No concerns | High |
| MLT:OPT | 0 | Some concerns | Low risk | No concerns | No concerns | No concerns | No concerns | High |
| MLT:PRAT | 0 | Some concerns | Low risk | No concerns | Some concerns | No concerns | No concerns | High |
| MLT:PSI | 0 | Some concerns | Low risk | No concerns | No concerns | No concerns | No concerns | High |
| MLT:RET | 0 | Some concerns | Low risk | No concerns | No concerns | No concerns | No concerns | High |
| MLT:SER | 0 | Some concerns | Low risk | No concerns | No concerns | No concerns | No concerns | High |
| MLT:SEXE | 0 | Some concerns | Low risk | No concerns | Some concerns | No concerns | No concerns | High |
| MLT:TAI | 0 | Some concerns | Low risk | No concerns | No concerns | No concerns | No concerns | High |
| MLT:VEN | 0 | Some concerns | Low risk | No concerns | No concerns | No concerns | No concerns | High |
| MUT:OPT | 0 | Some concerns | Low risk | No concerns | No concerns | Some concerns | No concerns | High |
| MUT:PRAT | 0 | Some concerns | Low risk | No concerns | Some concerns | No concerns | No concerns | High |
| MUT:PSI | 0 | Some concerns | Low risk | No concerns | Some concerns | No concerns | No concerns | High |
| MUT:RET | 0 | Some concerns | Low risk | No concerns | No concerns | No concerns | No concerns | High |
| MUT:SER | 0 | Some concerns | Low risk | No concerns | No concerns | Some concerns | No concerns | High |
| MUT:SEXE | 0 | Some concerns | Low risk | No concerns | Major concerns | No concerns | No concerns | High |
| MUT:TAI | 0 | Some concerns | Low risk | No concerns | No concerns | No concerns | No concerns | High |
| MUT:VEN | 0 | Some concerns | Low risk | No concerns | No concerns | No concerns | No concerns | High |
| OPT:PRAT | 0 | Some concerns | Low risk | No concerns | Some concerns | No concerns | No concerns | High |
| OPT:PSI | 0 | Some concerns | Low risk | No concerns | No concerns | No concerns | No concerns | High |
| OPT:RET | 0 | Some concerns | Low risk | No concerns | No concerns | No concerns | No concerns | High |
| OPT:SER | 0 | Some concerns | Low risk | No concerns | No concerns | No concerns | No concerns | High |
| OPT:SEXE | 0 | Some concerns | Low risk | No concerns | Some concerns | No concerns | No concerns | High |
| OPT:TAI | 0 | Some concerns | Low risk | No concerns | No concerns | No concerns | No concerns | High |
| OPT:VEN | 0 | Some concerns | Low risk | No concerns | No concerns | No concerns | No concerns | High |
| PLA:SEXE | 0 | Some concerns | Low risk | No concerns | Some concerns | No concerns | No concerns | High |
| PLA:VEN | 0 | Some concerns | Low risk | No concerns | No concerns | No concerns | No concerns | High |
| PRAT:PSI | 0 | Some concerns | Low risk | No concerns | Some concerns | No concerns | No concerns | High |
| PRAT:RET | 0 | Some concerns | Low risk | No concerns | No concerns | No concerns | No concerns | High |
| PRAT:SER | 0 | Some concerns | Low risk | No concerns | Some concerns | No concerns | No concerns | High |
| PRAT:SEXE | 0 | Some concerns | Low risk | No concerns | Major concerns | No concerns | No concerns | High |
| PRAT:TAI | 0 | Some concerns | Low risk | No concerns | Some concerns | No concerns | No concerns | High |
| PRAT:VEN | 0 | Some concerns | Low risk | No concerns | No concerns | No concerns | No concerns | High |
| PSI:RET | 0 | Some concerns | Low risk | No concerns | No concerns | No concerns | No concerns | High |
| PSI:SER | 0 | Some concerns | Low risk | No concerns | No concerns | No concerns | No concerns | High |
| PSI:SEXE | 0 | Some concerns | Low risk | No concerns | Some concerns | No concerns | No concerns | High |
| PSI:TAI | 0 | Some concerns | Low risk | No concerns | No concerns | No concerns | No concerns | High |
| PSI:VEN | 0 | Some concerns | Low risk | No concerns | No concerns | No concerns | No concerns | High |
| RET:SER | 0 | Some concerns | Low risk | No concerns | No concerns | No concerns | No concerns | High |
| RET:SEXE | 0 | Some concerns | Low risk | No concerns | Some concerns | Some concerns | No concerns | High |
| RET:TAI | 0 | Some concerns | Low risk | No concerns | No concerns | Some concerns | No concerns | High |
| RET:VEN | 0 | Some concerns | Low risk | No concerns | No concerns | No concerns | No concerns | High |
| SER:SEXE | 0 | Some concerns | Low risk | No concerns | Some concerns | No concerns | No concerns | High |
| SER:TAI | 0 | Some concerns | Low risk | No concerns | No concerns | No concerns | No concerns | High |
| SEXE:TAI | 0 | Some concerns | Low risk | No concerns | Some concerns | No concerns | No concerns | High |
| SEXE:VEN | 0 | Some concerns | Low risk | No concerns | No concerns | No concerns | No concerns | High |
| TAI:VEN | 0 | Some concerns | Low risk | No concerns | No concerns | Some concerns | No concerns | High |

Geriatric Depression Scale

| Comparison | Number of studies | Within-study bias | Reporting bias | Indirectness | Imprecision | Heterogeneity | Incoherence | Confidence rating |
|---|---|---|---|---|---|---|---|---|
| AEXE:PLA | 1 | Some concerns | Low risk | No concerns | No concerns | No concerns | Some concerns | High |
| AEXE:SEXE | 1 | Some concerns | Low risk | No concerns | No concerns | No concerns | Some concerns | High |
| BT:PLA | 4 | Some concerns | Low risk | No concerns | No concerns | No concerns | Some concerns | High |
| CIT:PLA | 1 | Some concerns | Low risk | No concerns | No concerns | No concerns | Some concerns | High |
| CST:PLA | 2 | Some concerns | Low risk | No concerns | No concerns | No concerns | Some concerns | High |
| ECT:PLA | 1 | Some concerns | Low risk | No concerns | No concerns | Some concerns | Some concerns | High |
| EGB:PLA | 1 | Some concerns | Low risk | No concerns | No concerns | No concerns | Some concerns | High |
| EXE:PLA | 1 | Some concerns | Low risk | No concerns | No concerns | No concerns | Some concerns | High |
| HMT:PLA | 1 | Some concerns | Low risk | No concerns | No concerns | No concerns | Some concerns | High |
| MAS:PLA | 1 | Some concerns | Low risk | No concerns | No concerns | No concerns | Some concerns | High |
| MLT:PLA | 3 | Some concerns | Low risk | No concerns | No concerns | No concerns | Some concerns | High |
| MUT:PLA | 2 | Some concerns | Low risk | No concerns | No concerns | No concerns | Some concerns | High |
| PLA:PSI | 2 | Some concerns | Low risk | No concerns | No concerns | No concerns | Some concerns | High |
| PLA:RET | 5 | Some concerns | Low risk | No concerns | No concerns | No concerns | Some concerns | High |
| PLA:RIV | 1 | Some concerns | Low risk | No concerns | No concerns | No concerns | Some concerns | High |
| PLA:SER | 1 | Some concerns | Low risk | No concerns | No concerns | No concerns | Some concerns | High |
| PLA:TAI | 1 | Some concerns | Low risk | No concerns | No concerns | No concerns | Some concerns | High |
| SER:VEN | 1 | Some concerns | Low risk | No concerns | Some concerns | No concerns | Some concerns | High |
| AEXE:BT | 0 | Some concerns | Low risk | No concerns | No concerns | No concerns | Some concerns | High |
| AEXE:CIT | 0 | Some concerns | Low risk | No concerns | No concerns | No concerns | Some concerns | High |
| AEXE:CST | 0 | Some concerns | Low risk | No concerns | No concerns | No concerns | Some concerns | High |
| AEXE:ECT | 0 | Some concerns | Low risk | No concerns | Some concerns | No concerns | Some concerns | High |
| AEXE:EGB | 0 | Some concerns | Low risk | No concerns | No concerns | No concerns | Some concerns | High |
| AEXE:EXE | 0 | Some concerns | Low risk | No concerns | No concerns | No concerns | Some concerns | High |
| AEXE:HMT | 0 | Some concerns | Low risk | No concerns | No concerns | No concerns | Some concerns | High |
| AEXE:MAS | 0 | Some concerns | Low risk | No concerns | No concerns | Some concerns | Some concerns | High |
| AEXE:MLT | 0 | Some concerns | Low risk | No concerns | No concerns | Some concerns | Some concerns | High |
| AEXE:MUT | 0 | Some concerns | Low risk | No concerns | No concerns | Some concerns | Some concerns | High |
| AEXE:PSI | 0 | Some concerns | Low risk | No concerns | No concerns | No concerns | Some concerns | High |
| AEXE:RET | 0 | Some concerns | Low risk | No concerns | No concerns | No concerns | Some concerns | High |
| AEXE:RIV | 0 | Some concerns | Low risk | No concerns | No concerns | Some concerns | Some concerns | High |
| AEXE:SER | 0 | Some concerns | Low risk | No concerns | No concerns | No concerns | Some concerns | High |
| AEXE:TAI | 0 | Some concerns | Low risk | No concerns | No concerns | No concerns | Some concerns | High |
| AEXE:VEN | 0 | Some concerns | Low risk | No concerns | Some concerns | No concerns | Some concerns | High |
| BT:CIT | 0 | Some concerns | Low risk | No concerns | No concerns | No concerns | Some concerns | High |
| BT:CST | 0 | Some concerns | Low risk | No concerns | No concerns | No concerns | Some concerns | High |
| BT:ECT | 0 | Some concerns | Low risk | No concerns | No concerns | No concerns | Some concerns | High |
| BT:EGB | 0 | Some concerns | Low risk | No concerns | No concerns | Some concerns | Some concerns | High |
| BT:EXE | 0 | Some concerns | Low risk | No concerns | No concerns | No concerns | Some concerns | High |
| BT:HMT | 0 | Some concerns | Low risk | No concerns | No concerns | No concerns | Some concerns | High |
| BT:MAS | 0 | Some concerns | Low risk | No concerns | No concerns | No concerns | Some concerns | High |
| BT:MLT | 0 | Some concerns | Low risk | No concerns | No concerns | No concerns | Some concerns | High |
| BT:MUT | 0 | Some concerns | Low risk | No concerns | No concerns | No concerns | Some concerns | High |
| BT:PSI | 0 | Some concerns | Low risk | No concerns | No concerns | No concerns | Some concerns | High |
| BT:RET | 0 | Some concerns | Low risk | No concerns | No concerns | No concerns | Some concerns | High |
| BT:RIV | 0 | Some concerns | Low risk | No concerns | No concerns | Some concerns | Some concerns | High |
| BT:SER | 0 | Some concerns | Low risk | No concerns | No concerns | No concerns | Some concerns | High |
| BT:SEXE | 0 | Some concerns | Low risk | No concerns | No concerns | Some concerns | Some concerns | High |
| BT:TAI | 0 | Some concerns | Low risk | No concerns | No concerns | No concerns | Some concerns | High |
| BT:VEN | 0 | Some concerns | Low risk | No concerns | Some concerns | No concerns | Some concerns | High |
| CIT:CST | 0 | Some concerns | Low risk | No concerns | No concerns | No concerns | Some concerns | High |
| CIT:ECT | 0 | Some concerns | Low risk | No concerns | No concerns | Some concerns | Some concerns | High |
| CIT:EGB | 0 | Some concerns | Low risk | No concerns | No concerns | Some concerns | Some concerns | High |
| CIT:EXE | 0 | Some concerns | Low risk | No concerns | No concerns | No concerns | Some concerns | High |
| CIT:HMT | 0 | Some concerns | Low risk | No concerns | No concerns | No concerns | Some concerns | High |
| CIT:MAS | 0 | Some concerns | Low risk | No concerns | No concerns | No concerns | Some concerns | High |
| CIT:MLT | 0 | Some concerns | Low risk | No concerns | No concerns | Some concerns | Some concerns | High |
| CIT:MUT | 0 | Some concerns | Low risk | No concerns | No concerns | No concerns | Some concerns | High |
| CIT:PSI | 0 | Some concerns | Low risk | No concerns | No concerns | No concerns | Some concerns | High |
| CIT:RET | 0 | Some concerns | Low risk | No concerns | No concerns | No concerns | Some concerns | High |
| CIT:RIV | 0 | Some concerns | Low risk | No concerns | Some concerns | No concerns | Some concerns | High |
| CIT:SER | 0 | Some concerns | Low risk | No concerns | No concerns | No concerns | Some concerns | High |
| CIT:SEXE | 0 | Some concerns | Low risk | No concerns | No concerns | Some concerns | Some concerns | High |
| CIT:TAI | 0 | Some concerns | Low risk | No concerns | No concerns | Some concerns | Some concerns | High |
| CIT:VEN | 0 | Some concerns | Low risk | No concerns | Some concerns | No concerns | Some concerns | High |
| CST:ECT | 0 | Some concerns | Low risk | No concerns | Some concerns | No concerns | Some concerns | High |
| CST:EGB | 0 | Some concerns | Low risk | No concerns | No concerns | No concerns | Some concerns | High |
| CST:EXE | 0 | Some concerns | Low risk | No concerns | No concerns | No concerns | Some concerns | High |
| CST:HMT | 0 | Some concerns | Low risk | No concerns | No concerns | No concerns | Some concerns | High |
| CST:MAS | 0 | Some concerns | Low risk | No concerns | No concerns | Some concerns | Some concerns | High |
| CST:MLT | 0 | Some concerns | Low risk | No concerns | No concerns | Some concerns | Some concerns | High |
| CST:MUT | 0 | Some concerns | Low risk | No concerns | No concerns | Some concerns | Some concerns | High |
| CST:PSI | 0 | Some concerns | Low risk | No concerns | No concerns | No concerns | Some concerns | High |
| CST:RET | 0 | Some concerns | Low risk | No concerns | No concerns | No concerns | Some concerns | High |
| CST:RIV | 0 | Some concerns | Low risk | No concerns | No concerns | Some concerns | Some concerns | High |
| CST:SER | 0 | Some concerns | Low risk | No concerns | No concerns | No concerns | Some concerns | High |
| CST:SEXE | 0 | Some concerns | Low risk | No concerns | No concerns | No concerns | Some concerns | High |
| CST:TAI | 0 | Some concerns | Low risk | No concerns | No concerns | No concerns | Some concerns | High |
| CST:VEN | 0 | Some concerns | Low risk | No concerns | Some concerns | No concerns | Some concerns | High |
| ECT:EGB | 0 | Some concerns | Low risk | No concerns | Some concerns | No concerns | Some concerns | High |
| ECT:EXE | 0 | Some concerns | Low risk | No concerns | Some concerns | No concerns | Some concerns | High |
| ECT:HMT | 0 | Some concerns | Low risk | No concerns | Some concerns | No concerns | Some concerns | High |
| ECT:MAS | 0 | Some concerns | Low risk | No concerns | No concerns | No concerns | Some concerns | High |
| ECT:MLT | 0 | Some concerns | Low risk | No concerns | No concerns | Some concerns | Some concerns | High |
| ECT:MUT | 0 | Some concerns | Low risk | No concerns | No concerns | No concerns | Some concerns | High |
| ECT:PSI | 0 | Some concerns | Low risk | No concerns | No concerns | Some concerns | Some concerns | High |
| ECT:RET | 0 | Some concerns | Low risk | No concerns | No concerns | No concerns | Some concerns | High |
| ECT:RIV | 0 | Some concerns | Low risk | No concerns | Some concerns | No concerns | Some concerns | High |
| ECT:SER | 0 | Some concerns | Low risk | No concerns | Some concerns | No concerns | Some concerns | High |

| | | | | | | | | |
|---|---|---|---|---|---|---|---|---|
| ECT:SEXE | 0 | Some concerns | Low risk | No concerns | Some concerns | No concerns | Some concerns | High |
| ECT:TAI | 0 | Some concerns | Low risk | No concerns | Some concerns | No concerns | Some concerns | High |
| ECT:VEN | 0 | Some concerns | Low risk | No concerns | Some concerns | No concerns | Some concerns | High |
| EGB:EXE | 0 | Some concerns | Low risk | No concerns | No concerns | No concerns | Some concerns | High |
| EGB:HMT | 0 | Some concerns | Low risk | No concerns | No concerns | No concerns | Some concerns | High |
| EGB:MAS | 0 | Some concerns | Low risk | No concerns | No concerns | Some concerns | Some concerns | High |
| EGB:MLT | 0 | Some concerns | Low risk | No concerns | Some concerns | No concerns | Some concerns | High |
| EGB:MUT | 0 | Some concerns | Low risk | No concerns | No concerns | Some concerns | Some concerns | High |
| EGB:PSI | 0 | Some concerns | Low risk | No concerns | No concerns | No concerns | Some concerns | High |
| EGB:RET | 0 | Some concerns | Low risk | No concerns | No concerns | Some concerns | Some concerns | High |
| EGB:RIV | 0 | Some concerns | Low risk | No concerns | No concerns | Some concerns | Some concerns | High |
| EGB:SER | 0 | Some concerns | Low risk | No concerns | No concerns | No concerns | Some concerns | High |
| EGB:SEXE | 0 | Some concerns | Low risk | No concerns | No concerns | Some concerns | Some concerns | High |
| EGB:TAI | 0 | Some concerns | Low risk | No concerns | No concerns | Some concerns | Some concerns | High |
| EGB:VEN | 0 | Some concerns | Low risk | No concerns | Some concerns | No concerns | Some concerns | High |
| EXE:HMT | 0 | Some concerns | Low risk | No concerns | No concerns | No concerns | Some concerns | High |
| EXE:MAS | 0 | Some concerns | Low risk | No concerns | No concerns | Some concerns | Some concerns | High |
| EXE:MLT | 0 | Some concerns | Low risk | No concerns | No concerns | Some concerns | Some concerns | High |
| EXE:MUT | 0 | Some concerns | Low risk | No concerns | No concerns | Some concerns | Some concerns | High |
| EXE:PSI | 0 | Some concerns | Low risk | No concerns | No concerns | No concerns | Some concerns | High |
| EXE:RET | 0 | Some concerns | Low risk | No concerns | No concerns | Some concerns | Some concerns | High |
| EXE:RIV | 0 | Some concerns | Low risk | No concerns | No concerns | Some concerns | Some concerns | High |
| EXE:SER | 0 | Some concerns | Low risk | No concerns | No concerns | No concerns | Some concerns | High |
| EXE:SEXE | 0 | Some concerns | Low risk | No concerns | No concerns | No concerns | Some concerns | High |
| EXE:TAI | 0 | Some concerns | Low risk | No concerns | No concerns | Some concerns | Some concerns | High |
| EXE:VEN | 0 | Some concerns | Low risk | No concerns | Some concerns | No concerns | Some concerns | High |
| HMT:MAS | 0 | Some concerns | Low risk | No concerns | No concerns | Some concerns | Some concerns | High |
| HMT:MLT | 0 | Some concerns | Low risk | No concerns | No concerns | Some concerns | Some concerns | High |
| HMT:MUT | 0 | Some concerns | Low risk | No concerns | No concerns | Some concerns | Some concerns | High |
| HMT:PSI | 0 | Some concerns | Low risk | No concerns | No concerns | No concerns | Some concerns | High |
| HMT:RET | 0 | Some concerns | Low risk | No concerns | No concerns | Some concerns | Some concerns | High |
| HMT:RIV | 0 | Some concerns | Low risk | No concerns | No concerns | Some concerns | Some concerns | High |
| HMT:SER | 0 | Some concerns | Low risk | No concerns | No concerns | No concerns | Some concerns | High |
| HMT:SEXE | 0 | Some concerns | Low risk | No concerns | No concerns | No concerns | Some concerns | High |
| HMT:TAI | 0 | Some concerns | Low risk | No concerns | No concerns | Some concerns | Some concerns | High |
| HMT:VEN | 0 | Some concerns | Low risk | No concerns | Some concerns | No concerns | Some concerns | High |
| MAS:MLT | 0 | Some concerns | Low risk | No concerns | No concerns | No concerns | Some concerns | High |
| MAS:MUT | 0 | Some concerns | Low risk | No concerns | No concerns | No concerns | Some concerns | High |
| MAS:PSI | 0 | Some concerns | Low risk | No concerns | No concerns | No concerns | Some concerns | High |
| MAS:RET | 0 | Some concerns | Low risk | No concerns | No concerns | No concerns | Some concerns | High |
| MAS:RIV | 0 | Some concerns | Low risk | No concerns | Some concerns | No concerns | Some concerns | High |
| MAS:SER | 0 | Some concerns | Low risk | No concerns | No concerns | Some concerns | Some concerns | High |
| MAS:SEXE | 0 | Some concerns | Low risk | No concerns | No concerns | Some concerns | Some concerns | High |
| MAS:TAI | 0 | Some concerns | Low risk | No concerns | No concerns | Some concerns | Some concerns | High |
| MAS:VEN | 0 | Some concerns | Low risk | No concerns | Some concerns | No concerns | Some concerns | High |
| MLT:MUT | 0 | Some concerns | Low risk | No concerns | No concerns | No concerns | Some concerns | High |
| MLT:PSI | 0 | Some concerns | Low risk | No concerns | No concerns | No concerns | Some concerns | High |
| MLT:RET | 0 | Some concerns | Low risk | No concerns | No concerns | No concerns | Some concerns | High |
| MLT:RIV | 0 | Some concerns | Low risk | No concerns | No concerns | Some concerns | Some concerns | High |
| MLT:SER | 0 | Some concerns | Low risk | No concerns | No concerns | Some concerns | Some concerns | High |
| MLT:SEXE | 0 | Some concerns | Low risk | No concerns | Some concerns | No concerns | Some concerns | High |
| MLT:TAI | 0 | Some concerns | Low risk | No concerns | Some concerns | No concerns | Some concerns | High |
| MLT:VEN | 0 | Some concerns | Low risk | No concerns | Some concerns | No concerns | Some concerns | High |
| MUT:PSI | 0 | Some concerns | Low risk | No concerns | No concerns | No concerns | Some concerns | High |
| MUT:RET | 0 | Some concerns | Low risk | No concerns | No concerns | No concerns | Some concerns | High |
| MUT:RIV | 0 | Some concerns | Low risk | No concerns | No concerns | Some concerns | Some concerns | High |
| MUT:SER | 0 | Some concerns | Low risk | No concerns | No concerns | Some concerns | Some concerns | High |
| MUT:SEXE | 0 | Some concerns | Low risk | No concerns | No concerns | Some concerns | Some concerns | High |
| MUT:TAI | 0 | Some concerns | Low risk | No concerns | No concerns | Some concerns | Some concerns | High |
| MUT:VEN | 0 | Some concerns | Low risk | No concerns | Some concerns | No concerns | Some concerns | High |
| PLA:SEXE | 0 | Some concerns | Low risk | No concerns | No concerns | No concerns | Some concerns | High |
| PLA:VEN | 0 | Some concerns | Low risk | No concerns | Some concerns | No concerns | Some concerns | High |
| PSI:RET | 0 | Some concerns | Low risk | No concerns | No concerns | No concerns | Some concerns | High |
| PSI:RIV | 0 | Some concerns | Low risk | No concerns | No concerns | Some concerns | Some concerns | High |
| PSI:SER | 0 | Some concerns | Low risk | No concerns | No concerns | No concerns | Some concerns | High |
| PSI:SEXE | 0 | Some concerns | Low risk | No concerns | No concerns | No concerns | Some concerns | High |
| PSI:TAI | 0 | Some concerns | Low risk | No concerns | No concerns | No concerns | Some concerns | High |
| PSI:VEN | 0 | Some concerns | Low risk | No concerns | Some concerns | No concerns | Some concerns | High |
| RET:RIV | 0 | Some concerns | Low risk | No concerns | No concerns | Some concerns | Some concerns | High |
| RET:SER | 0 | Some concerns | Low risk | No concerns | No concerns | Some concerns | Some concerns | High |
| RET:SEXE | 0 | Some concerns | Low risk | No concerns | No concerns | Some concerns | Some concerns | High |
| RET:TAI | 0 | Some concerns | Low risk | No concerns | No concerns | Some concerns | Some concerns | High |
| RET:VEN | 0 | Some concerns | Low risk | No concerns | Some concerns | No concerns | Some concerns | High |
| RIV:SER | 0 | Some concerns | Low risk | No concerns | No concerns | Some concerns | Some concerns | High |
| RIV:SEXE | 0 | Some concerns | Low risk | No concerns | Some concerns | No concerns | Some concerns | High |
| RIV:TAI | 0 | Some concerns | Low risk | No concerns | Some concerns | No concerns | Some concerns | High |
| RIV:VEN | 0 | Some concerns | Low risk | No concerns | No concerns | Some concerns | Some concerns | High |
| SER:SEXE | 0 | Some concerns | Low risk | No concerns | No concerns | No concerns | Some concerns | High |
| SER:TAI | 0 | Some concerns | Low risk | No concerns | No concerns | No concerns | Some concerns | High |
| SEXE:TAI | 0 | Some concerns | Low risk | No concerns | No concerns | Some concerns | Some concerns | High |
| SEXE:VEN | 0 | Some concerns | Low risk | No concerns | Some concerns | No concerns | Some concerns | High |
| TAI:VEN | 0 | Some concerns | Low risk | No concerns | Some concerns | No concerns | Some concerns | High |

Hamilton Depression Rating Scale

| Comparison | Number of studies | Within-study bias | Reporting bias | Indirectness | Imprecision | Heterogeneity | Incoherence | Confidence rating |
|---|---|---|---|---|---|---|---|---|
| AMI:FLU | 1 | Some concerns | Low risk | No concerns | No concerns | No concerns | Some concerns | High |
| BT:PLA | 2 | Some concerns | Low risk | No concerns | No concerns | No concerns | Some concerns | High |
| CIT:PLA | 1 | Some concerns | Low risk | No concerns | No concerns | No concerns | Some concerns | High |
| CST:PLA | 1 | Some concerns | Low risk | No concerns | No concerns | No concerns | Some concerns | High |
| DON:EGB | 1 | Some concerns | Low risk | No concerns | No concerns | No concerns | No concerns | High |
| DON:PLA | 2 | Some concerns | Low risk | No concerns | No concerns | No concerns | No concerns | High |
| EGB:PLA | 1 | Some concerns | Low risk | No concerns | No concerns | No concerns | No concerns | High |
| EXE:MLT | 1 | Major concerns | Low risk | Some concerns | No concerns | No concerns | No concerns | High |
| EXE:PLA | 2 | Some concerns | Low risk | No concerns | No concerns | No concerns | Some concerns | High |
| FLU:PLA | 1 | Some concerns | Low risk | No concerns | No concerns | No concerns | Some concerns | High |
| HMT:PLA | 1 | Some concerns | Low risk | No concerns | No concerns | No concerns | Some concerns | High |
| IMI:PLA | 1 | Some concerns | Low risk | No concerns | No concerns | No concerns | Some concerns | High |
| MLT:PLA | 1 | Major concerns | Low risk | Some concerns | No concerns | No concerns | Some concerns | High |
| MUT:PLA | 1 | Some concerns | Low risk | No concerns | No concerns | No concerns | Some concerns | High |
| PLA:RIV | 1 | Some concerns | Low risk | No concerns | No concerns | No concerns | Some concerns | High |
| PLA:SER | 2 | Some concerns | Low risk | No concerns | No concerns | Some concerns | Some concerns | High |
| SER:VEN | 1 | Some concerns | Low risk | No concerns | No concerns | No concerns | Some concerns | High |
| AMI:BT | 0 | Some concerns | Low risk | No concerns | No concerns | No concerns | Some concerns | High |
| AMI:CIT | 0 | Some concerns | Low risk | No concerns | No concerns | Some concerns | Some concerns | High |
| AMI:CST | 0 | Some concerns | Low risk | No concerns | No concerns | Some concerns | Some concerns | High |
| AMI:DON | 0 | Some concerns | Low risk | No concerns | No concerns | Some concerns | Some concerns | High |
| AMI:EGB | 0 | Some concerns | Low risk | No concerns | No concerns | Some concerns | Some concerns | High |
| AMI:EXE | 0 | Some concerns | Low risk | No concerns | No concerns | Some concerns | Some concerns | High |
| AMI:HMT | 0 | Some concerns | Low risk | No concerns | No concerns | Some concerns | Some concerns | High |
| AMI:IMI | 0 | Some concerns | Low risk | No concerns | No concerns | Some concerns | Some concerns | High |
| AMI:MLT | 0 | Some concerns | Low risk | No concerns | No concerns | Some concerns | Some concerns | High |
| AMI:MUT | 0 | Some concerns | Low risk | No concerns | No concerns | No concerns | Some concerns | High |
| AMI:PLA | 0 | Some concerns | Low risk | No concerns | No concerns | Some concerns | Some concerns | High |
| AMI:RIV | 0 | Some concerns | Low risk | No concerns | No concerns | Some concerns | Some concerns | High |
| AMI:SER | 0 | Some concerns | Low risk | No concerns | No concerns | Some concerns | Some concerns | High |
| AMI:VEN | 0 | Some concerns | Low risk | No concerns | No concerns | Some concerns | Some concerns | High |
| BT:CIT | 0 | Some concerns | Low risk | No concerns | No concerns | No concerns | Some concerns | High |
| BT:CST | 0 | Some concerns | Low risk | No concerns | No concerns | No concerns | Some concerns | High |
| BT:DON | 0 | Some concerns | Low risk | No concerns | No concerns | No concerns | Some concerns | High |
| BT:EGB | 0 | Some concerns | Low risk | No concerns | No concerns | No concerns | Some concerns | High |
| BT:EXE | 0 | Some concerns | Low risk | No concerns | No concerns | No concerns | Some concerns | High |
| BT:FLU | 0 | Some concerns | Low risk | No concerns | No concerns | Some concerns | Some concerns | High |
| BT:HMT | 0 | Some concerns | Low risk | No concerns | No concerns | No concerns | Some concerns | High |
| BT:IMI | 0 | Some concerns | Low risk | No concerns | No concerns | Some concerns | Major concerns | High |
| BT:MLT | 0 | Some concerns | Low risk | No concerns | No concerns | No concerns | Some concerns | High |
| BT:MUT | 0 | Some concerns | Low risk | No concerns | No concerns | Some concerns | Some concerns | High |
| BT:RIV | 0 | Some concerns | Low risk | No concerns | No concerns | No concerns | Some concerns | High |
| BT:SER | 0 | Some concerns | Low risk | No concerns | No concerns | No concerns | Some concerns | High |
| BT:VEN | 0 | Some concerns | Low risk | No concerns | No concerns | Some concerns | Some concerns | High |
| CIT:CST | 0 | Some concerns | Low risk | No concerns | No concerns | No concerns | Some concerns | High |
| CIT:DON | 0 | Some concerns | Low risk | No concerns | No concerns | No concerns | Some concerns | High |
| CIT:EGB | 0 | Some concerns | Low risk | No concerns | No concerns | No concerns | Some concerns | High |
| CIT:EXE | 0 | Some concerns | Low risk | No concerns | No concerns | No concerns | Some concerns | High |
| CIT:FLU | 0 | Some concerns | Low risk | No concerns | No concerns | Some concerns | Some concerns | High |
| CIT:HMT | 0 | Some concerns | Low risk | No concerns | No concerns | No concerns | Major concerns | High |
| CIT:IMI | 0 | Some concerns | Low risk | No concerns | No concerns | Some concerns | Some concerns | High |
| CIT:MLT | 0 | Some concerns | Low risk | No concerns | No concerns | No concerns | Some concerns | High |
| CIT:MUT | 0 | Some concerns | Low risk | No concerns | No concerns | Some concerns | Some concerns | High |
| CIT:RIV | 0 | Some concerns | Low risk | No concerns | No concerns | No concerns | Some concerns | High |
| CIT:SER | 0 | Some concerns | Low risk | No concerns | No concerns | No concerns | Some concerns | High |
| CIT:VEN | 0 | Some concerns | Low risk | No concerns | No concerns | Some concerns | Some concerns | High |
| CST:DON | 0 | Some concerns | Low risk | No concerns | No concerns | No concerns | No concerns | High |
| CST:EGB | 0 | Some concerns | Low risk | No concerns | No concerns | No concerns | Some concerns | High |
| CST:EXE | 0 | Some concerns | Low risk | No concerns | No concerns | No concerns | Some concerns | High |
| CST:FLU | 0 | Some concerns | Low risk | No concerns | No concerns | Some concerns | Some concerns | High |
| CST:HMT | 0 | Some concerns | Low risk | No concerns | No concerns | Some concerns | Some concerns | High |
| CST:IMI | 0 | Some concerns | Low risk | No concerns | No concerns | Some concerns | Some concerns | High |
| CST:MLT | 0 | Some concerns | Low risk | No concerns | No concerns | No concerns | Some concerns | High |
| CST:MUT | 0 | Some concerns | Low risk | No concerns | No concerns | Some concerns | Some concerns | High |
| CST:RIV | 0 | Some concerns | Low risk | No concerns | No concerns | No concerns | Major concerns | High |
| CST:SER | 0 | Some concerns | Low risk | No concerns | No concerns | No concerns | Some concerns | High |
| CST:VEN | 0 | Some concerns | Low risk | No concerns | No concerns | Some concerns | Some concerns | High |
| DON:EXE | 0 | Some concerns | Low risk | No concerns | No concerns | No concerns | Some concerns | High |
| DON:FLU | 0 | Some concerns | Low risk | No concerns | No concerns | No concerns | Some concerns | High |
| DON:HMT | 0 | Some concerns | Low risk | No concerns | No concerns | No concerns | Some concerns | High |
| DON:IMI | 0 | Some concerns | Low risk | No concerns | No concerns | No concerns | Some concerns | High |
| DON:MLT | 0 | Some concerns | Low risk | No concerns | No concerns | No concerns | Some concerns | High |
| DON:MUT | 0 | Some concerns | Low risk | No concerns | No concerns | Some concerns | Some concerns | High |
| DON:RIV | 0 | Some concerns | Low risk | No concerns | No concerns | No concerns | Major concerns | High |
| DON:SER | 0 | Some concerns | Low risk | No concerns | No concerns | No concerns | Major concerns | High |
| DON:VEN | 0 | Some concerns | Low risk | No concerns | No concerns | No concerns | Some concerns | High |
| EGB:EXE | 0 | Some concerns | Low risk | No concerns | No concerns | No concerns | Some concerns | High |
| EGB:FLU | 0 | Some concerns | Low risk | No concerns | No concerns | Some concerns | Some concerns | High |
| EGB:HMT | 0 | Some concerns | Low risk | No concerns | No concerns | No concerns | Some concerns | High |
| EGB:IMI | 0 | Some concerns | Low risk | No concerns | No concerns | No concerns | Some concerns | High |
| EGB:MLT | 0 | Some concerns | Low risk | No concerns | No concerns | No concerns | Some concerns | High |
| EGB:MUT | 0 | Some concerns | Low risk | No concerns | No concerns | Some concerns | Some concerns | High |
| EGB:RIV | 0 | Some concerns | Low risk | No concerns | No concerns | No concerns | Some concerns | High |
| EGB:SER | 0 | Some concerns | Low risk | No concerns | No concerns | No concerns | Some concerns | High |
| EGB:VEN | 0 | Some concerns | Low risk | No concerns | No concerns | No concerns | Some concerns | High |
| EXE:FLU | 0 | Some concerns | Low risk | No concerns | No concerns | No concerns | Some concerns | High |
| EXE:HMT | 0 | Some concerns | Low risk | No concerns | No concerns | No concerns | Some concerns | High |
| EXE:IMI | 0 | Some concerns | Low risk | No concerns | No concerns | No concerns | Some concerns | High |
| EXE:MUT | 0 | Some concerns | Low risk | No concerns | No concerns | Some concerns | Some concerns | High |

| | | | | | | | | |
|---|---|---|---|---|---|---|---|---|
| **EXE:RIV** | 0 | Some concerns | Low risk | No concerns | No concerns | No concerns | Some concerns | High |
| **EXE:SER** | 0 | Some concerns | Low risk | No concerns | No concerns | Some concerns | Some concerns | High |
| **EXE:VEN** | 0 | Some concerns | Low risk | No concerns | No concerns | No concerns | Some concerns | High |
| **FLU:HMT** | 0 | Some concerns | Low risk | No concerns | No concerns | Some concerns | Some concerns | High |
| **FLU:IMI** | 0 | Some concerns | Low risk | No concerns | No concerns | No concerns | Some concerns | High |
| **FLU:MLT** | 0 | Some concerns | Low risk | No concerns | No concerns | Some concerns | Some concerns | High |
| **FLU:MUT** | 0 | Some concerns | Low risk | No concerns | No concerns | No concerns | Some concerns | High |
| **FLU:RIV** | 0 | Some concerns | Low risk | No concerns | No concerns | Some concerns | Some concerns | High |
| **FLU:SER** | 0 | Some concerns | Low risk | No concerns | No concerns | Some concerns | Major concerns | High |
| **FLU:VEN** | 0 | Some concerns | Low risk | No concerns | No concerns | Some concerns | Some concerns | High |
| **HMT:IMI** | 0 | Some concerns | Low risk | No concerns | No concerns | No concerns | Some concerns | High |
| **HMT:MLT** | 0 | Some concerns | Low risk | No concerns | No concerns | No concerns | Some concerns | High |
| **HMT:MUT** | 0 | Some concerns | Low risk | No concerns | No concerns | Some concerns | Some concerns | High |
| **HMT:RIV** | 0 | Some concerns | Low risk | No concerns | No concerns | No concerns | Some concerns | High |
| **HMT:SER** | 0 | Some concerns | Low risk | No concerns | No concerns | Some concerns | Some concerns | High |
| **HMT:VEN** | 0 | Some concerns | Low risk | No concerns | No concerns | No concerns | Major concerns | High |
| **IMI:MLT** | 0 | Some concerns | Low risk | No concerns | No concerns | Some concerns | Some concerns | High |
| **IMI:MUT** | 0 | Some concerns | Low risk | No concerns | No concerns | No concerns | Some concerns | High |
| **IMI:RIV** | 0 | Some concerns | Low risk | No concerns | No concerns | No concerns | Some concerns | High |
| **IMI:SER** | 0 | Some concerns | Low risk | No concerns | No concerns | Some concerns | Some concerns | High |
| **IMI:VEN** | 0 | Some concerns | Low risk | No concerns | No concerns | Some concerns | Some concerns | High |
| **MLT:MUT** | 0 | Some concerns | Low risk | No concerns | Some concerns | No concerns | Major concerns | High |
| **MLT:RIV** | 0 | Some concerns | Low risk | No concerns | No concerns | No concerns | Some concerns | High |
| **MLT:SER** | 0 | Some concerns | Low risk | No concerns | No concerns | No concerns | Some concerns | High |
| **MLT:VEN** | 0 | Some concerns | Low risk | No concerns | No concerns | No concerns | Some concerns | High |
| **MUT:RIV** | 0 | Some concerns | Low risk | No concerns | No concerns | Some concerns | Some concerns | High |
| **MUT:SER** | 0 | Some concerns | Low risk | No concerns | No concerns | Some concerns | Some concerns | High |
| **MUT:VEN** | 0 | Some concerns | Low risk | No concerns | Some concerns | No concerns | Some concerns | High |
| **PLA:VEN** | 0 | Some concerns | Low risk | No concerns | No concerns | No concerns | Major concerns | High |
| **RIV:SER** | 0 | Some concerns | Low risk | No concerns | No concerns | No concerns | Some concerns | High |
| **RIV:VEN** | 0 | Some concerns | Low risk | No concerns | No concerns | No concerns | Some concerns | High |

## Mini-Mental State Examination

| Comparison | Number of | Within-study bias | Reporting bias | Indirectness | Imprecision | Heterogeneity | Incoherence | Confidence rating |
|---|---|---|---|---|---|---|---|---|
| AMI:PLA | 1 | Some concerns | Low risk | No concerns | No concerns | No concerns | No concerns | High |
| ARI:PLA | 3 | Some concerns | Low risk | No concerns | No concerns | No concerns | No concerns | High |
| BT:PLA | 4 | Some concerns | Low risk | No concerns | No concerns | No concerns | No concerns | High |
| CIT:PLA | 1 | Some concerns | Low risk | No concerns | No concerns | No concerns | No concerns | High |
| CST:EXE | 1 | Some concerns | Low risk | No concerns | No concerns | No concerns | No concerns | High |
| CST:PLA | 7 | Some concerns | Low risk | No concerns | No concerns | No concerns | No concerns | High |
| ECT:PLA | 1 | Some concerns | Low risk | No concerns | No concerns | No concerns | No concerns | High |
| EGB:PLA | 2 | Some concerns | Low risk | No concerns | No concerns | No concerns | No concerns | High |
| EGB:RIV | 1 | Some concerns | Low risk | No concerns | No concerns | No concerns | No concerns | High |
| EXE:MLT | 1 | Some concerns | Low risk | No concerns | No concerns | Some concerns | No concerns | High |
| EXE:PLA | 2 | Some concerns | Low risk | No concerns | No concerns | No concerns | No concerns | High |
| FLU:PLA | 2 | Some concerns | Low risk | No concerns | No concerns | Some concerns | No concerns | High |
| HMT:PLA | 2 | Some concerns | Low risk | No concerns | No concerns | No concerns | No concerns | High |
| IMI:PLA | 2 | Some concerns | Low risk | No concerns | No concerns | Some concerns | No concerns | High |
| LGT:PLA | 2 | Some concerns | Low risk | No concerns | No concerns | No concerns | No concerns | High |
| MAS:PLA | 1 | Some concerns | Low risk | No concerns | No concerns | Some concerns | No concerns | High |
| MLT:PLA | 4 | Some concerns | Low risk | No concerns | No concerns | No concerns | No concerns | High |
| MUT:PLA | 1 | Some concerns | Low risk | No concerns | No concerns | Some concerns | No concerns | High |
| OPT:PLA | 1 | Some concerns | Low risk | No concerns | Some concerns | No concerns | No concerns | High |
| PLA:PSI | 3 | Some concerns | Low risk | No concerns | No concerns | No concerns | No concerns | High |
| PLA:RET | 5 | Some concerns | Low risk | No concerns | No concerns | No concerns | No concerns | High |
| PLA:RIV | 2 | Some concerns | Low risk | No concerns | No concerns | No concerns | No concerns | High |
| PLA:SER | 4 | Some concerns | Low risk | No concerns | No concerns | Some concerns | No concerns | High |
| PLA:TAI | 1 | Some concerns | Low risk | No concerns | No concerns | No concerns | No concerns | High |
| AMI:ARI | 0 | Some concerns | Low risk | No concerns | No concerns | Some concerns | No concerns | High |
| AMI:BT | 0 | Some concerns | Low risk | No concerns | No concerns | Some concerns | No concerns | High |
| AMI:CIT | 0 | Some concerns | Low risk | No concerns | No concerns | Some concerns | No concerns | High |
| AMI:CST | 0 | Some concerns | Low risk | No concerns | No concerns | No concerns | No concerns | High |
| AMI:ECT | 0 | Some concerns | Low risk | No concerns | Some concerns | No concerns | No concerns | High |
| AMI:EGB | 0 | Some concerns | Low risk | No concerns | No concerns | No concerns | No concerns | High |
| AMI:EXE | 0 | Some concerns | Low risk | No concerns | No concerns | No concerns | No concerns | High |
| AMI:FLU | 0 | Some concerns | Low risk | No concerns | Some concerns | No concerns | No concerns | High |
| AMI:HMT | 0 | Some concerns | Low risk | No concerns | No concerns | Some concerns | No concerns | High |
| AMI:IMI | 0 | Some concerns | Low risk | No concerns | Some concerns | Some concerns | No concerns | High |
| AMI:LGT | 0 | Some concerns | Low risk | No concerns | Some concerns | No concerns | No concerns | High |
| AMI:MAS | 0 | Some concerns | Low risk | No concerns | No concerns | Some concerns | No concerns | High |
| AMI:MLT | 0 | Some concerns | Low risk | No concerns | Some concerns | No concerns | No concerns | High |
| AMI:MUT | 0 | Some concerns | Low risk | No concerns | Some concerns | No concerns | No concerns | High |
| AMI:OPT | 0 | Some concerns | Low risk | No concerns | Some concerns | Some concerns | No concerns | High |
| AMI:PSI | 0 | Some concerns | Low risk | No concerns | No concerns | Some concerns | No concerns | High |
| AMI:RET | 0 | Some concerns | Low risk | No concerns | No concerns | Some concerns | No concerns | High |
| AMI:RIV | 0 | Some concerns | Low risk | No concerns | No concerns | Some concerns | No concerns | High |
| AMI:SER | 0 | Some concerns | Low risk | No concerns | Some concerns | No concerns | No concerns | High |
| AMI:TAI | 0 | Some concerns | Low risk | No concerns | Some concerns | No concerns | No concerns | High |
| ARI:BT | 0 | Some concerns | Low risk | No concerns | No concerns | No concerns | No concerns | High |
| ARI:CIT | 0 | Some concerns | Low risk | No concerns | No concerns | Some concerns | No concerns | High |
| ARI:CST | 0 | Some concerns | Low risk | No concerns | No concerns | Some concerns | No concerns | High |
| ARI:ECT | 0 | Some concerns | Low risk | No concerns | No concerns | No concerns | No concerns | High |
| ARI:EGB | 0 | Some concerns | Low risk | No concerns | No concerns | No concerns | No concerns | High |
| ARI:EXE | 0 | Some concerns | Low risk | No concerns | No concerns | No concerns | No concerns | High |
| ARI:FLU | 0 | Some concerns | Low risk | No concerns | No concerns | Some concerns | No concerns | High |
| ARI:HMT | 0 | Some concerns | Low risk | No concerns | No concerns | No concerns | No concerns | High |
| ARI:IMI | 0 | Some concerns | Low risk | No concerns | Some concerns | No concerns | No concerns | High |
| ARI:LGT | 0 | Some concerns | Low risk | No concerns | No concerns | No concerns | No concerns | High |
| ARI:MAS | 0 | Some concerns | Low risk | No concerns | Some concerns | No concerns | No concerns | High |
| ARI:MLT | 0 | Some concerns | Low risk | No concerns | No concerns | No concerns | No concerns | High |
| ARI:MUT | 0 | Some concerns | Low risk | No concerns | No concerns | Some concerns | No concerns | High |
| ARI:OPT | 0 | Some concerns | Low risk | No concerns | Some concerns | No concerns | No concerns | High |
| ARI:PSI | 0 | Some concerns | Low risk | No concerns | No concerns | No concerns | No concerns | High |
| ARI:RET | 0 | Some concerns | Low risk | No concerns | No concerns | Some concerns | No concerns | High |
| ARI:RIV | 0 | Some concerns | Low risk | No concerns | No concerns | Some concerns | No concerns | High |
| ARI:SER | 0 | Some concerns | Low risk | No concerns | No concerns | Some concerns | No concerns | High |
| ARI:TAI | 0 | Some concerns | Low risk | No concerns | No concerns | No concerns | No concerns | High |
| BT:CIT | 0 | Some concerns | Low risk | No concerns | No concerns | Some concerns | No concerns | High |
| BT:CST | 0 | Some concerns | Low risk | No concerns | No concerns | No concerns | No concerns | High |
| BT:ECT | 0 | Some concerns | Low risk | No concerns | No concerns | No concerns | No concerns | High |
| BT:EGB | 0 | Some concerns | Low risk | No concerns | No concerns | No concerns | No concerns | High |
| BT:EXE | 0 | Some concerns | Low risk | No concerns | No concerns | No concerns | No concerns | High |
| BT:FLU | 0 | Some concerns | Low risk | No concerns | No concerns | Some concerns | No concerns | High |
| BT:HMT | 0 | Some concerns | Low risk | No concerns | No concerns | No concerns | No concerns | High |
| BT:IMI | 0 | Some concerns | Low risk | No concerns | Some concerns | No concerns | No concerns | High |
| BT:LGT | 0 | Some concerns | Low risk | No concerns | No concerns | No concerns | No concerns | High |
| BT:MAS | 0 | Some concerns | Low risk | No concerns | No concerns | Some concerns | No concerns | High |
| BT:MLT | 0 | Some concerns | Low risk | No concerns | No concerns | No concerns | No concerns | High |
| BT:MUT | 0 | Some concerns | Low risk | No concerns | Some concerns | No concerns | No concerns | High |
| BT:OPT | 0 | Some concerns | Low risk | No concerns | Some concerns | No concerns | No concerns | High |
| BT:PSI | 0 | Some concerns | Low risk | No concerns | No concerns | No concerns | No concerns | High |
| BT:RET | 0 | Some concerns | Low risk | No concerns | No concerns | Some concerns | No concerns | High |
| BT:RIV | 0 | Some concerns | Low risk | No concerns | No concerns | Some concerns | No concerns | High |

| | | | | | | | | |
|---|---|---|---|---|---|---|---|---|
| BT:SER | 0 | Some concerns | Low risk | No concerns | Some concerns | No concerns | No concerns | High |
| BT:TAI | 0 | Some concerns | Low risk | No concerns | No concerns | No concerns | No concerns | High |
| CIT:CST | 0 | Some concerns | Low risk | No concerns | No concerns | No concerns | No concerns | High |
| CIT:ECT | 0 | Some concerns | Low risk | No concerns | Some concerns | No concerns | No concerns | High |
| CIT:EGB | 0 | Some concerns | Low risk | No concerns | No concerns | No concerns | No concerns | High |
| CIT:EXE | 0 | Some concerns | Low risk | No concerns | No concerns | No concerns | No concerns | High |
| CIT:FLU | 0 | Some concerns | Low risk | No concerns | Some concerns | No concerns | No concerns | High |
| CIT:HMT | 0 | Some concerns | Low risk | No concerns | Some concerns | No concerns | No concerns | High |
| CIT:IMI | 0 | Some concerns | Low risk | No concerns | Some concerns | No concerns | No concerns | High |
| CIT:LGT | 0 | Some concerns | Low risk | No concerns | Some concerns | No concerns | No concerns | High |
| CIT:MAS | 0 | Some concerns | Low risk | No concerns | No concerns | No concerns | No concerns | High |
| CIT:MLT | 0 | Some concerns | Low risk | No concerns | Some concerns | No concerns | No concerns | High |
| CIT:MUT | 0 | Some concerns | Low risk | No concerns | Some concerns | No concerns | No concerns | High |
| CIT:OPT | 0 | Some concerns | Low risk | No concerns | Major concerns | No concerns | No concerns | High |
| CIT:PSI | 0 | Some concerns | Low risk | No concerns | No concerns | Some concerns | No concerns | High |
| CIT:RET | 0 | Some concerns | Low risk | No concerns | No concerns | No concerns | No concerns | High |
| CIT:RIV | 0 | Some concerns | Low risk | No concerns | No concerns | No concerns | No concerns | High |
| CIT:SER | 0 | Some concerns | Low risk | No concerns | Some concerns | No concerns | No concerns | High |
| CIT:TAI | 0 | Some concerns | Low risk | No concerns | Some concerns | No concerns | No concerns | High |
| CST:ECT | 0 | Some concerns | Low risk | No concerns | Some concerns | No concerns | No concerns | High |
| CST:EGB | 0 | Some concerns | Low risk | No concerns | No concerns | No concerns | No concerns | High |
| CST:FLU | 0 | Some concerns | Low risk | No concerns | No concerns | Some concerns | No concerns | High |
| CST:HMT | 0 | Some concerns | Low risk | No concerns | No concerns | Some concerns | No concerns | High |
| CST:IMI | 0 | Some concerns | Low risk | No concerns | Some concerns | No concerns | No concerns | High |
| CST:LGT | 0 | Some concerns | Low risk | No concerns | Some concerns | No concerns | No concerns | High |
| CST:MAS | 0 | Some concerns | Low risk | No concerns | No concerns | No concerns | No concerns | High |
| CST:MLT | 0 | Some concerns | Low risk | No concerns | No concerns | Some concerns | No concerns | High |
| CST:MUT | 0 | Some concerns | Low risk | No concerns | Some concerns | No concerns | No concerns | High |
| CST:OPT | 0 | Some concerns | Low risk | No concerns | Some concerns | Some concerns | No concerns | High |
| CST:PSI | 0 | Some concerns | Low risk | No concerns | No concerns | No concerns | No concerns | High |
| CST:RET | 0 | Some concerns | Low risk | No concerns | No concerns | No concerns | No concerns | High |
| CST:RIV | 0 | Some concerns | Low risk | No concerns | No concerns | No concerns | No concerns | High |
| CST:SER | 0 | Some concerns | Low risk | No concerns | No concerns | Some concerns | No concerns | High |
| CST:TAI | 0 | Some concerns | Low risk | No concerns | Some concerns | No concerns | No concerns | High |
| ECT:EGB | 0 | Some concerns | Low risk | No concerns | No concerns | Some concerns | No concerns | High |
| ECT:EXE | 0 | Some concerns | Low risk | No concerns | No concerns | Some concerns | No concerns | High |
| ECT:FLU | 0 | Some concerns | Low risk | No concerns | No concerns | No concerns | No concerns | High |
| ECT:HMT | 0 | Some concerns | Low risk | No concerns | No concerns | No concerns | No concerns | High |
| ECT:IMI | 0 | Some concerns | Low risk | No concerns | Some concerns | No concerns | No concerns | High |
| ECT:LGT | 0 | Some concerns | Low risk | No concerns | No concerns | Some concerns | No concerns | High |
| ECT:MAS | 0 | Some concerns | Low risk | No concerns | Some concerns | No concerns | No concerns | High |
| ECT:MLT | 0 | Some concerns | Low risk | No concerns | No concerns | No concerns | No concerns | High |
| ECT:MUT | 0 | Some concerns | Low risk | No concerns | Some concerns | No concerns | No concerns | High |
| ECT:OPT | 0 | Some concerns | Low risk | No concerns | Some concerns | No concerns | No concerns | High |
| ECT:PSI | 0 | Some concerns | Low risk | No concerns | No concerns | No concerns | No concerns | High |
| ECT:RET | 0 | Some concerns | Low risk | No concerns | Some concerns | No concerns | No concerns | High |
| ECT:RIV | 0 | Some concerns | Low risk | No concerns | Some concerns | No concerns | No concerns | High |
| ECT:SER | 0 | Some concerns | Low risk | No concerns | No concerns | No concerns | No concerns | High |
| ECT:TAI | 0 | Some concerns | Low risk | No concerns | No concerns | No concerns | No concerns | High |
| EGB:EXE | 0 | Some concerns | Low risk | No concerns | No concerns | No concerns | No concerns | High |
| EGB:FLU | 0 | Some concerns | Low risk | No concerns | Some concerns | No concerns | No concerns | High |
| EGB:HMT | 0 | Some concerns | Low risk | No concerns | No concerns | No concerns | No concerns | High |
| EGB:IMI | 0 | Some concerns | Low risk | No concerns | No concerns | Major concerns | No concerns | High |
| EGB:LGT | 0 | Some concerns | Low risk | No concerns | No concerns | Some concerns | No concerns | High |
| EGB:MAS | 0 | Some concerns | Low risk | No concerns | No concerns | Some concerns | No concerns | High |
| EGB:MLT | 0 | Some concerns | Low risk | No concerns | No concerns | No concerns | No concerns | High |
| EGB:MUT | 0 | Some concerns | Low risk | No concerns | Some concerns | No concerns | No concerns | High |
| EGB:OPT | 0 | Some concerns | Low risk | No concerns | Some concerns | No concerns | No concerns | High |
| EGB:PSI | 0 | Some concerns | Low risk | No concerns | No concerns | No concerns | No concerns | High |
| EGB:RET | 0 | Some concerns | Low risk | No concerns | No concerns | No concerns | No concerns | High |
| EGB:SER | 0 | Some concerns | Low risk | No concerns | Some concerns | No concerns | No concerns | High |
| EGB:TAI | 0 | Some concerns | Low risk | No concerns | No concerns | Some concerns | No concerns | High |
| EXE:FLU | 0 | Some concerns | Low risk | No concerns | Some concerns | No concerns | No concerns | High |
| EXE:HMT | 0 | Some concerns | Low risk | No concerns | No concerns | No concerns | No concerns | High |
| EXE:IMI | 0 | Some concerns | Low risk | No concerns | No concerns | Major concerns | No concerns | High |
| EXE:LGT | 0 | Some concerns | Low risk | No concerns | No concerns | Some concerns | No concerns | High |
| EXE:MAS | 0 | Some concerns | Low risk | No concerns | No concerns | No concerns | No concerns | High |
| EXE:MUT | 0 | Some concerns | Low risk | No concerns | Some concerns | No concerns | No concerns | High |
| EXE:OPT | 0 | Some concerns | Low risk | No concerns | Some concerns | No concerns | No concerns | High |
| EXE:PSI | 0 | Some concerns | Low risk | No concerns | No concerns | No concerns | No concerns | High |
| EXE:RET | 0 | Some concerns | Low risk | No concerns | No concerns | No concerns | No concerns | High |
| EXE:RIV | 0 | Some concerns | Low risk | No concerns | No concerns | No concerns | No concerns | High |
| EXE:SER | 0 | Some concerns | Low risk | No concerns | Some concerns | No concerns | No concerns | High |
| EXE:TAI | 0 | Some concerns | Low risk | No concerns | No concerns | Some concerns | No concerns | High |
| FLU:HMT | 0 | Some concerns | Low risk | No concerns | Some concerns | No concerns | No concerns | High |
| FLU:IMI | 0 | Some concerns | Low risk | No concerns | Some concerns | No concerns | No concerns | High |
| FLU:LGT | 0 | Some concerns | Low risk | No concerns | Some concerns | No concerns | No concerns | High |
| FLU:MAS | 0 | Some concerns | Low risk | No concerns | Some concerns | No concerns | No concerns | High |
| FLU:MLT | 0 | Some concerns | Low risk | No concerns | No concerns | Some concerns | No concerns | High |
| FLU:MUT | 0 | Some concerns | Low risk | No concerns | No concerns | Some concerns | No concerns | High |
| FLU:OPT | 0 | Some concerns | Low risk | No concerns | Some concerns | No concerns | No concerns | High |
| FLU:PSI | 0 | Some concerns | Low risk | No concerns | No concerns | Some concerns | No concerns | High |

| | | | | | | | | |
|---|---|---|---|---|---|---|---|---|
| FLU:RET | 0 | Some concerns | Low risk | No concerns | Some concerns | No concerns | No concerns | High |
| FLU:RIV | 0 | Some concerns | Low risk | No concerns | Some concerns | No concerns | No concerns | High |
| FLU:SER | 0 | Some concerns | Low risk | No concerns | No concerns | Some concerns | No concerns | High |
| FLU:TAI | 0 | Some concerns | Low risk | No concerns | No concerns | Some concerns | No concerns | High |
| HMT:IMI | 0 | Some concerns | Low risk | No concerns | Some concerns | No concerns | No concerns | High |
| HMT:LGT | 0 | Some concerns | Low risk | No concerns | No concerns | No concerns | No concerns | High |
| HMT:MAS | 0 | Some concerns | Low risk | No concerns | Some concerns | No concerns | No concerns | High |
| HMT:MLT | 0 | Some concerns | Low risk | No concerns | No concerns | No concerns | No concerns | High |
| HMT:MUT | 0 | Some concerns | Low risk | No concerns | Some concerns | No concerns | No concerns | High |
| HMT:OPT | 0 | Some concerns | Low risk | No concerns | Some concerns | No concerns | No concerns | High |
| HMT:PSI | 0 | Some concerns | Low risk | No concerns | No concerns | No concerns | No concerns | High |
| HMT:RET | 0 | Some concerns | Low risk | No concerns | No concerns | Some concerns | No concerns | High |
| HMT:RIV | 0 | Some concerns | Low risk | No concerns | No concerns | Some concerns | No concerns | High |
| HMT:SER | 0 | Some concerns | Low risk | No concerns | Some concerns | No concerns | No concerns | High |
| HMT:TAI | 0 | Some concerns | Low risk | No concerns | No concerns | No concerns | No concerns | High |
| IMI:LGT | 0 | Some concerns | Low risk | No concerns | Some concerns | Some concerns | No concerns | High |
| IMI:MAS | 0 | Some concerns | Low risk | No concerns | Some concerns | Some concerns | No concerns | High |
| IMI:MLT | 0 | Some concerns | Low risk | No concerns | Some concerns | No concerns | No concerns | High |
| IMI:MUT | 0 | Some concerns | Low risk | No concerns | Some concerns | No concerns | No concerns | High |
| IMI:OPT | 0 | Some concerns | Low risk | No concerns | Major concerns | No concerns | No concerns | High |
| IMI:PSI | 0 | Some concerns | Low risk | No concerns | Some concerns | No concerns | No concerns | High |
| IMI:RET | 0 | Some concerns | Low risk | No concerns | Some concerns | No concerns | No concerns | High |
| IMI:RIV | 0 | Some concerns | Low risk | No concerns | Some concerns | No concerns | No concerns | High |
| IMI:SER | 0 | Some concerns | Low risk | No concerns | Some concerns | No concerns | No concerns | High |
| IMI:TAI | 0 | Some concerns | Low risk | No concerns | Some concerns | No concerns | No concerns | High |
| LGT:MAS | 0 | Some concerns | Low risk | No concerns | Some concerns | No concerns | No concerns | High |
| LGT:MLT | 0 | Some concerns | Low risk | No concerns | No concerns | No concerns | No concerns | High |
| LGT:MUT | 0 | Some concerns | Low risk | No concerns | Some concerns | No concerns | No concerns | High |
| LGT:OPT | 0 | Some concerns | Low risk | No concerns | Some concerns | No concerns | No concerns | High |
| LGT:PSI | 0 | Some concerns | Low risk | No concerns | No concerns | No concerns | No concerns | High |
| LGT:RET | 0 | Some concerns | Low risk | No concerns | Some concerns | No concerns | No concerns | High |
| LGT:RIV | 0 | Some concerns | Low risk | No concerns | Some concerns | No concerns | No concerns | High |
| LGT:SER | 0 | Some concerns | Low risk | No concerns | Some concerns | No concerns | No concerns | High |
| LGT:TAI | 0 | Some concerns | Low risk | No concerns | No concerns | Some concerns | No concerns | High |
| MAS:MLT | 0 | Some concerns | Low risk | No concerns | Some concerns | No concerns | No concerns | High |
| MAS:MUT | 0 | Some concerns | Low risk | No concerns | Some concerns | No concerns | No concerns | High |
| MAS:OPT | 0 | Some concerns | Low risk | No concerns | Major concerns | No concerns | No concerns | High |
| MAS:PSI | 0 | Some concerns | Low risk | No concerns | No concerns | Some concerns | No concerns | High |
| MAS:RET | 0 | Some concerns | Low risk | No concerns | No concerns | No concerns | No concerns | High |
| MAS:RIV | 0 | Some concerns | Low risk | No concerns | No concerns | No concerns | No concerns | High |
| MAS:SER | 0 | Some concerns | Low risk | No concerns | Some concerns | No concerns | No concerns | High |
| MAS:TAI | 0 | Some concerns | Low risk | No concerns | Some concerns | No concerns | No concerns | High |
| MLT:MUT | 0 | Some concerns | Low risk | No concerns | Some concerns | No concerns | No concerns | High |
| MLT:OPT | 0 | Some concerns | Low risk | No concerns | Some concerns | No concerns | No concerns | High |
| MLT:PSI | 0 | Some concerns | Low risk | No concerns | No concerns | No concerns | No concerns | High |
| MLT:RET | 0 | Some concerns | Low risk | No concerns | No concerns | Some concerns | No concerns | High |
| MLT:RIV | 0 | Some concerns | Low risk | No concerns | Some concerns | No concerns | No concerns | High |
| MLT:SER | 0 | Some concerns | Low risk | No concerns | Some concerns | No concerns | No concerns | High |
| MLT:TAI | 0 | Some concerns | Low risk | No concerns | No concerns | No concerns | No concerns | High |
| MUT:OPT | 0 | Some concerns | Low risk | No concerns | Some concerns | Some concerns | No concerns | High |
| MUT:PSI | 0 | Some concerns | Low risk | No concerns | Some concerns | No concerns | No concerns | High |
| MUT:RET | 0 | Some concerns | Low risk | No concerns | Some concerns | No concerns | No concerns | High |
| MUT:RIV | 0 | Some concerns | Low risk | No concerns | Some concerns | No concerns | No concerns | High |
| MUT:SER | 0 | Some concerns | Low risk | No concerns | No concerns | No concerns | No concerns | High |
| MUT:TAI | 0 | Some concerns | Low risk | No concerns | Some concerns | No concerns | No concerns | High |
| OPT:PSI | 0 | Some concerns | Low risk | No concerns | Some concerns | No concerns | No concerns | High |
| OPT:RET | 0 | Some concerns | Low risk | No concerns | Major concerns | No concerns | No concerns | High |
| OPT:RIV | 0 | Some concerns | Low risk | No concerns | Major concerns | No concerns | No concerns | High |
| OPT:SER | 0 | Some concerns | Low risk | No concerns | Some concerns | No concerns | No concerns | High |
| OPT:TAI | 0 | Some concerns | Low risk | No concerns | Some concerns | No concerns | No concerns | High |
| PSI:RET | 0 | Some concerns | Low risk | No concerns | No concerns | No concerns | No concerns | High |
| PSI:RIV | 0 | Some concerns | Low risk | No concerns | No concerns | Some concerns | No concerns | High |
| PSI:SER | 0 | Some concerns | Low risk | No concerns | Some concerns | No concerns | No concerns | High |
| PSI:TAI | 0 | Some concerns | Low risk | No concerns | No concerns | No concerns | No concerns | High |
| RET:RIV | 0 | Some concerns | Low risk | No concerns | No concerns | No concerns | No concerns | High |
| RET:SER | 0 | Some concerns | Low risk | No concerns | No concerns | Some concerns | No concerns | High |
| RET:TAI | 0 | Some concerns | Low risk | No concerns | Some concerns | No concerns | No concerns | High |
| RIV:SER | 0 | Some concerns | Low risk | No concerns | No concerns | Some concerns | No concerns | High |
| RIV:TAI | 0 | Some concerns | Low risk | No concerns | Some concerns | No concerns | No concerns | High |
| SER:TAI | 0 | Some concerns | Low risk | No concerns | Some concerns | No concerns | No concerns | High |

Neuropsychiatric Inventory

| Comparison | Number of studies | Within-study bias | Reporting bias | Indirectness | Imprecision | Heterogeneity | Incoherence | Confidence rating |
|---|---|---|---|---|---|---|---|---|
| AEXE:PLA | 1 | Some concerns | Low risk | No concerns | No concerns | No concerns | No concerns | High |
| ARI:PLA | 3 | Some concerns | Low risk | No concerns | No concerns | No concerns | No concerns | High |
| AT:PLA | 1 | Some concerns | Low risk | No concerns | No concerns | No concerns | No concerns | High |
| BT:PLA | 1 | Some concerns | Low risk | No concerns | No concerns | No concerns | No concerns | High |
| CIT:PLA | 1 | Some concerns | Low risk | No concerns | No concerns | No concerns | No concerns | High |
| CST:PLA | 2 | Some concerns | Low risk | No concerns | No concerns | No concerns | No concerns | High |
| CST:RET | 1 | Some concerns | Low risk | No concerns | No concerns | No concerns | No concerns | High |
| DIS:PLA | 1 | Some concerns | Low risk | No concerns | No concerns | No concerns | No concerns | High |
| DON:EGB | 1 | Some concerns | Low risk | No concerns | No concerns | No concerns | No concerns | High |
| EGB:PLA | 2 | Some concerns | Low risk | No concerns | No concerns | No concerns | No concerns | High |
| EXE:PLA | 2 | Some concerns | Low risk | No concerns | No concerns | No concerns | No concerns | High |
| LGT:PLA | 1 | Some concerns | Low risk | No concerns | No concerns | No concerns | No concerns | High |
| MLT:PLA | 3 | Some concerns | Low risk | No concerns | No concerns | No concerns | No concerns | High |
| MUT:PLA | 1 | Some concerns | Low risk | No concerns | No concerns | No concerns | No concerns | High |
| PLA:PSI | 2 | Some concerns | Low risk | No concerns | No concerns | No concerns | No concerns | High |
| PLA:RET | 3 | Some concerns | Low risk | No concerns | No concerns | No concerns | No concerns | High |
| PLA:SER | 1 | Some concerns | Low risk | No concerns | No concerns | No concerns | No concerns | High |
| PLA:TAI | 1 | Some concerns | Low risk | No concerns | No concerns | No concerns | No concerns | High |
| AEXE:ARI | 0 | Some concerns | Low risk | No concerns | No concerns | No concerns | No concerns | High |
| AEXE:AT | 0 | Some concerns | Low risk | No concerns | No concerns | No concerns | No concerns | High |
| AEXE:BT | 0 | Some concerns | Low risk | No concerns | No concerns | No concerns | No concerns | High |
| AEXE:CIT | 0 | Some concerns | Low risk | No concerns | No concerns | No concerns | No concerns | High |
| AEXE:CST | 0 | Some concerns | Low risk | No concerns | No concerns | No concerns | No concerns | High |
| AEXE:DIS | 0 | Some concerns | Low risk | No concerns | No concerns | No concerns | No concerns | High |
| AEXE:DON | 0 | Some concerns | Low risk | No concerns | No concerns | No concerns | No concerns | High |
| AEXE:EGB | 0 | Some concerns | Low risk | No concerns | No concerns | No concerns | No concerns | High |
| AEXE:EXE | 0 | Some concerns | Low risk | No concerns | No concerns | No concerns | No concerns | High |
| AEXE:LGT | 0 | Some concerns | Low risk | No concerns | No concerns | No concerns | No concerns | High |
| AEXE:MLT | 0 | Some concerns | Low risk | No concerns | No concerns | No concerns | No concerns | High |
| AEXE:MUT | 0 | Some concerns | Low risk | No concerns | No concerns | No concerns | No concerns | High |
| AEXE:PSI | 0 | Some concerns | Low risk | No concerns | No concerns | No concerns | No concerns | High |
| AEXE:RET | 0 | Some concerns | Low risk | No concerns | No concerns | No concerns | No concerns | High |
| AEXE:SER | 0 | Some concerns | Low risk | No concerns | No concerns | No concerns | No concerns | High |
| AEXE:TAI | 0 | Some concerns | Low risk | No concerns | No concerns | No concerns | No concerns | High |
| ARI:AT | 0 | Some concerns | Low risk | No concerns | No concerns | No concerns | No concerns | High |
| ARI:BT | 0 | Some concerns | Low risk | No concerns | No concerns | No concerns | No concerns | High |
| ARI:CIT | 0 | Some concerns | Low risk | No concerns | No concerns | No concerns | No concerns | High |
| ARI:CST | 0 | Some concerns | Low risk | No concerns | No concerns | No concerns | No concerns | High |
| ARI:DIS | 0 | Some concerns | Low risk | No concerns | No concerns | No concerns | No concerns | High |
| ARI:DON | 0 | Some concerns | Low risk | No concerns | No concerns | No concerns | No concerns | High |
| ARI:EGB | 0 | Some concerns | Low risk | No concerns | No concerns | No concerns | No concerns | High |
| ARI:EXE | 0 | Some concerns | Low risk | No concerns | No concerns | No concerns | No concerns | High |
| ARI:LGT | 0 | Some concerns | Low risk | No concerns | No concerns | No concerns | No concerns | High |
| ARI:MLT | 0 | Some concerns | Low risk | No concerns | No concerns | No concerns | No concerns | High |
| ARI:MUT | 0 | Some concerns | Low risk | No concerns | No concerns | No concerns | No concerns | High |
| ARI:PSI | 0 | Some concerns | Low risk | No concerns | No concerns | No concerns | No concerns | High |
| ARI:RET | 0 | Some concerns | Low risk | No concerns | No concerns | No concerns | No concerns | High |
| ARI:SER | 0 | Some concerns | Low risk | No concerns | No concerns | No concerns | No concerns | High |
| ARI:TAI | 0 | Some concerns | Low risk | No concerns | No concerns | No concerns | No concerns | High |
| AT:BT | 0 | Some concerns | Low risk | No concerns | No concerns | No concerns | No concerns | High |
| AT:CIT | 0 | Some concerns | Low risk | No concerns | No concerns | No concerns | No concerns | High |
| AT:CST | 0 | Some concerns | Low risk | No concerns | No concerns | No concerns | No concerns | High |
| AT:DIS | 0 | Some concerns | Low risk | No concerns | No concerns | No concerns | No concerns | High |
| AT:DON | 0 | Some concerns | Low risk | No concerns | No concerns | No concerns | No concerns | High |
| AT:EGB | 0 | Some concerns | Low risk | No concerns | No concerns | No concerns | No concerns | High |
| AT:EXE | 0 | Some concerns | Low risk | No concerns | No concerns | No concerns | No concerns | High |
| AT:LGT | 0 | Some concerns | Low risk | No concerns | No concerns | Some concerns | No concerns | High |
| AT:MLT | 0 | Some concerns | Low risk | No concerns | No concerns | No concerns | No concerns | High |
| AT:MUT | 0 | Some concerns | Low risk | No concerns | No concerns | No concerns | No concerns | High |
| AT:PSI | 0 | Some concerns | Low risk | No concerns | No concerns | No concerns | No concerns | High |
| AT:RET | 0 | Some concerns | Low risk | No concerns | No concerns | No concerns | No concerns | High |
| AT:SER | 0 | Some concerns | Low risk | No concerns | No concerns | No concerns | No concerns | High |
| AT:TAI | 0 | Some concerns | Low risk | No concerns | No concerns | No concerns | No concerns | High |
| BT:CIT | 0 | Some concerns | Low risk | No concerns | No concerns | No concerns | No concerns | High |
| BT:CST | 0 | Some concerns | Low risk | No concerns | No concerns | No concerns | No concerns | High |
| BT:DIS | 0 | Some concerns | Low risk | No concerns | No concerns | No concerns | No concerns | High |
| BT:DON | 0 | Some concerns | Low risk | No concerns | No concerns | No concerns | No concerns | High |
| BT:EGB | 0 | Some concerns | Low risk | No concerns | No concerns | No concerns | No concerns | High |
| BT:EXE | 0 | Some concerns | Low risk | No concerns | No concerns | No concerns | No concerns | High |
| BT:LGT | 0 | Some concerns | Low risk | No concerns | No concerns | No concerns | No concerns | High |
| BT:MLT | 0 | Some concerns | Low risk | No concerns | No concerns | No concerns | No concerns | High |
| BT:MUT | 0 | Some concerns | Low risk | No concerns | No concerns | No concerns | No concerns | High |
| BT:PSI | 0 | Some concerns | Low risk | No concerns | No concerns | No concerns | No concerns | High |
| BT:RET | 0 | Some concerns | Low risk | No concerns | No concerns | No concerns | No concerns | High |
| BT:SER | 0 | Some concerns | Low risk | No concerns | No concerns | No concerns | No concerns | High |
| BT:TAI | 0 | Some concerns | Low risk | No concerns | No concerns | No concerns | No concerns | High |
| CIT:CST | 0 | Some concerns | Low risk | No concerns | No concerns | No concerns | No concerns | High |
| CIT:DIS | 0 | Some concerns | Low risk | No concerns | No concerns | No concerns | No concerns | High |
| CIT:DON | 0 | Some concerns | Low risk | No concerns | No concerns | No concerns | No concerns | High |
| CIT:EGB | 0 | Some concerns | Low risk | No concerns | No concerns | No concerns | No concerns | High |
| CIT:EXE | 0 | Some concerns | Low risk | No concerns | No concerns | No concerns | No concerns | High |
| CIT:LGT | 0 | Some concerns | Low risk | No concerns | No concerns | No concerns | No concerns | High |
| CIT:MLT | 0 | Some concerns | Low risk | No concerns | No concerns | No concerns | No concerns | High |
| CIT:MUT | 0 | Some concerns | Low risk | No concerns | No concerns | No concerns | No concerns | High |
| CIT:PSI | 0 | Some concerns | Low risk | No concerns | No concerns | No concerns | No concerns | High |
| CIT:RET | 0 | Some concerns | Low risk | No concerns | No concerns | No concerns | No concerns | High |
| CIT:SER | 0 | Some concerns | Low risk | No concerns | No concerns | No concerns | No concerns | High |
| CIT:TAI | 0 | Some concerns | Low risk | No concerns | No concerns | No concerns | No concerns | High |
| CST:DIS | 0 | Some concerns | Low risk | No concerns | No concerns | No concerns | No concerns | High |

| | | | | | | | | |
|---|---|---|---|---|---|---|---|---|
| **CST:DON** | 0 | Some concerns | Low risk | No concerns | No concerns | No concerns | No concerns | High |
| **CST:EGB** | 0 | Some concerns | Low risk | No concerns | No concerns | No concerns | No concerns | High |
| **CST:EXE** | 0 | Some concerns | Low risk | No concerns | No concerns | No concerns | No concerns | High |
| **CST:LGT** | 0 | Some concerns | Low risk | No concerns | No concerns | No concerns | No concerns | High |
| **CST:MLT** | 0 | Some concerns | Low risk | No concerns | No concerns | No concerns | No concerns | High |
| **CST:MUT** | 0 | Some concerns | Low risk | No concerns | No concerns | No concerns | No concerns | High |
| **CST:PSI** | 0 | Some concerns | Low risk | No concerns | No concerns | No concerns | No concerns | High |
| **CST:SER** | 0 | Some concerns | Low risk | No concerns | No concerns | No concerns | No concerns | High |
| **CST:TAI** | 0 | Some concerns | Low risk | No concerns | No concerns | No concerns | No concerns | High |
| **DIS:DON** | 0 | Some concerns | Low risk | No concerns | No concerns | Some concerns | No concerns | High |
| **DIS:EGB** | 0 | Some concerns | Low risk | No concerns | No concerns | No concerns | No concerns | High |
| **DIS:EXE** | 0 | Some concerns | Low risk | No concerns | No concerns | No concerns | No concerns | High |
| **DIS:LGT** | 0 | Some concerns | Low risk | No concerns | No concerns | No concerns | No concerns | High |
| **DIS:MLT** | 0 | Some concerns | Low risk | No concerns | No concerns | No concerns | No concerns | High |
| **DIS:MUT** | 0 | Some concerns | Low risk | No concerns | No concerns | No concerns | No concerns | High |
| **DIS:PSI** | 0 | Some concerns | Low risk | No concerns | No concerns | No concerns | No concerns | High |
| **DIS:RET** | 0 | Some concerns | Low risk | No concerns | No concerns | No concerns | No concerns | High |
| **DIS:SER** | 0 | Some concerns | Low risk | No concerns | No concerns | No concerns | No concerns | High |
| **DIS:TAI** | 0 | Some concerns | Low risk | No concerns | No concerns | No concerns | No concerns | High |
| **DON:EXE** | 0 | Some concerns | Low risk | No concerns | No concerns | No concerns | No concerns | High |
| **DON:LGT** | 0 | Some concerns | Low risk | No concerns | No concerns | No concerns | No concerns | High |
| **DON:MLT** | 0 | Some concerns | Low risk | No concerns | No concerns | No concerns | No concerns | High |
| **DON:MUT** | 0 | Some concerns | Low risk | No concerns | No concerns | No concerns | No concerns | High |
| **DON:PLA** | 0 | Some concerns | Low risk | No concerns | No concerns | No concerns | No concerns | High |
| **DON:PSI** | 0 | Some concerns | Low risk | No concerns | No concerns | No concerns | No concerns | High |
| **DON:RET** | 0 | Some concerns | Low risk | No concerns | No concerns | No concerns | No concerns | High |
| **DON:SER** | 0 | Some concerns | Low risk | No concerns | No concerns | No concerns | No concerns | High |
| **DON:TAI** | 0 | Some concerns | Low risk | No concerns | No concerns | No concerns | No concerns | High |
| **EGB:EXE** | 0 | Some concerns | Low risk | No concerns | No concerns | No concerns | No concerns | High |
| **EGB:LGT** | 0 | Some concerns | Low risk | No concerns | No concerns | No concerns | No concerns | High |
| **EGB:MLT** | 0 | Some concerns | Low risk | No concerns | No concerns | No concerns | No concerns | High |
| **EGB:MUT** | 0 | Some concerns | Low risk | No concerns | No concerns | No concerns | No concerns | High |
| **EGB:PSI** | 0 | Some concerns | Low risk | No concerns | No concerns | No concerns | No concerns | High |
| **EGB:RET** | 0 | Some concerns | Low risk | No concerns | No concerns | No concerns | No concerns | High |
| **EGB:SER** | 0 | Some concerns | Low risk | No concerns | No concerns | No concerns | No concerns | High |
| **EGB:TAI** | 0 | Some concerns | Low risk | No concerns | No concerns | No concerns | No concerns | High |
| **EXE:LGT** | 0 | Some concerns | Low risk | No concerns | No concerns | No concerns | No concerns | High |
| **EXE:MLT** | 0 | Some concerns | Low risk | No concerns | No concerns | No concerns | No concerns | High |
| **EXE:MUT** | 0 | Some concerns | Low risk | No concerns | No concerns | No concerns | No concerns | High |
| **EXE:PSI** | 0 | Some concerns | Low risk | No concerns | No concerns | No concerns | No concerns | High |
| **EXE:RET** | 0 | Some concerns | Low risk | No concerns | No concerns | No concerns | No concerns | High |
| **EXE:SER** | 0 | Some concerns | Low risk | No concerns | No concerns | No concerns | No concerns | High |
| **EXE:TAI** | 0 | Some concerns | Low risk | No concerns | No concerns | No concerns | No concerns | High |
| **LGT:MLT** | 0 | Some concerns | Low risk | No concerns | No concerns | No concerns | No concerns | High |
| **LGT:MUT** | 0 | Some concerns | Low risk | No concerns | No concerns | No concerns | No concerns | High |
| **LGT:PSI** | 0 | Some concerns | Low risk | No concerns | No concerns | No concerns | No concerns | High |
| **LGT:RET** | 0 | Some concerns | Low risk | No concerns | No concerns | No concerns | No concerns | High |
| **LGT:SER** | 0 | Some concerns | Low risk | No concerns | No concerns | No concerns | No concerns | High |
| **LGT:TAI** | 0 | Some concerns | Low risk | No concerns | No concerns | No concerns | No concerns | High |
| **MLT:MUT** | 0 | Some concerns | Low risk | No concerns | No concerns | No concerns | No concerns | High |
| **MLT:PSI** | 0 | Some concerns | Low risk | No concerns | No concerns | No concerns | No concerns | High |
| **MLT:RET** | 0 | Some concerns | Low risk | No concerns | No concerns | No concerns | No concerns | High |
| **MLT:SER** | 0 | Some concerns | Low risk | No concerns | No concerns | No concerns | No concerns | High |
| **MLT:TAI** | 0 | Some concerns | Low risk | No concerns | No concerns | No concerns | No concerns | High |
| **MUT:PSI** | 0 | Some concerns | Low risk | No concerns | No concerns | No concerns | No concerns | High |
| **MUT:RET** | 0 | Some concerns | Low risk | No concerns | No concerns | No concerns | No concerns | High |
| **MUT:SER** | 0 | Some concerns | Low risk | No concerns | No concerns | No concerns | No concerns | High |
| **MUT:TAI** | 0 | Some concerns | Low risk | No concerns | No concerns | No concerns | No concerns | High |
| **PSI:RET** | 0 | Some concerns | Low risk | No concerns | No concerns | No concerns | No concerns | High |
| **PSI:SER** | 0 | Some concerns | Low risk | No concerns | No concerns | No concerns | No concerns | High |
| **PSI:TAI** | 0 | Some concerns | Low risk | No concerns | No concerns | No concerns | No concerns | High |
| **RET:SER** | 0 | Some concerns | Low risk | No concerns | No concerns | No concerns | No concerns | High |
| **RET:TAI** | 0 | Some concerns | Low risk | No concerns | No concerns | No concerns | No concerns | High |
| **SER:TAI** | 0 | Some concerns | Low risk | No concerns | No concerns | No concerns | No concerns | High |

Cohen-Mansfeld Agitation Inventory

| Comparison | Number of studies | Within-study bias | Reporting bias | Indirectness | Imprecision | Heterogeneity | Incoherence | Confidence rating |
|---|---|---|---|---|---|---|---|---|
| ARI:PLA | 2 | Some concerns | Low risk | No concerns | No concerns | No concerns | No concerns | High |
| AT:PLA | 1 | Some concerns | Low risk | No concerns | No concerns | No concerns | No concerns | High |
| CIT:PLA | 1 | Some concerns | Low risk | No concerns | No concerns | No concerns | No concerns | High |
| FLU:PLA | 1 | Some concerns | Low risk | No concerns | No concerns | Some concerns | Some concerns | High |
| LGT:PLA | 2 | Some concerns | Low risk | No concerns | No concerns | No concerns | No concerns | High |
| MAS:PLA | 2 | Some concerns | Low risk | No concerns | No concerns | No concerns | No concerns | High |
| PLA:PRAT | 1 | Some concerns | Low risk | No concerns | No concerns | Some concerns | Some concerns | High |
| ARI:AT | 0 | Some concerns | Low risk | No concerns | No concerns | No concerns | Some concerns | High |
| ARI:CIT | 0 | Some concerns | Low risk | No concerns | No concerns | No concerns | Some concerns | High |
| ARI:FLU | 0 | Some concerns | Low risk | No concerns | No concerns | Some concerns | Some concerns | High |
| ARI:LGT | 0 | Some concerns | Low risk | No concerns | No concerns | No concerns | Some concerns | High |
| ARI:MAS | 0 | Some concerns | Low risk | No concerns | No concerns | No concerns | Some concerns | High |
| ARI:PRAT | 0 | Some concerns | Low risk | No concerns | No concerns | Some concerns | Some concerns | High |
| AT:CIT | 0 | Some concerns | Low risk | No concerns | No concerns | No concerns | Some concerns | High |
| AT:FLU | 0 | Some concerns | Low risk | No concerns | No concerns | Some concerns | Some concerns | High |
| AT:LGT | 0 | Some concerns | Low risk | No concerns | No concerns | No concerns | Some concerns | High |
| AT:MAS | 0 | Some concerns | Low risk | No concerns | No concerns | No concerns | Some concerns | High |
| AT:PRAT | 0 | Some concerns | Low risk | No concerns | No concerns | Some concerns | Some concerns | High |
| CIT:FLU | 0 | Some concerns | Low risk | No concerns | No concerns | Some concerns | Some concerns | High |
| CIT:LGT | 0 | Some concerns | Low risk | No concerns | No concerns | No concerns | Some concerns | High |
| CIT:MAS | 0 | Some concerns | Low risk | No concerns | No concerns | No concerns | Some concerns | High |
| CIT:PRAT | 0 | Some concerns | Low risk | No concerns | No concerns | Some concerns | Some concerns | High |
| FLU:LGT | 0 | Some concerns | Low risk | No concerns | No concerns | Some concerns | Some concerns | High |
| FLU:MAS | 0 | Some concerns | Low risk | No concerns | No concerns | Some concerns | Some concerns | High |
| FLU:PRAT | 0 | Some concerns | Low risk | No concerns | No concerns | Some concerns | Some concerns | High |
| LGT:MAS | 0 | Some concerns | Low risk | No concerns | No concerns | No concerns | Some concerns | High |
| LGT:PRAT | 0 | Some concerns | Low risk | No concerns | No concerns | Some concerns | Some concerns | High |
| MAS:PRAT | 0 | Some concerns | Low risk | No concerns | No concerns | Some concerns | Some concerns | High |
